# Global trends and projections of high BMI burden and its independent impact on atrial fibrillation and flutter

**DOI:** 10.1093/inthealth/ihaf005

**Published:** 2025-02-04

**Authors:** YuBin He, YaPing Ren, YaYun Zhang, Zixiong Zhu, Xuewen Li

**Affiliations:** Department of Cardiovascular Medicine, Third Hospital of Shanxi Medical University, Shanxi Bethune Hospital, Shanxi Academy of Medical Sciences, No. 99 Longcheng Street, Taiyuan, Shanxi 030032, China; Department of Cardiovascular Medicine, Taiyuan Central Hospital, Taiyuan, Shanxi 030032, China; Department of Cardiovascular Medicine, Third Hospital of Shanxi Medical University, Shanxi Bethune Hospital, Shanxi Academy of Medical Sciences, No. 99 Longcheng Street, Taiyuan, Shanxi 030032, China; Department of Cardiovascular Medicine, Third Hospital of Shanxi Medical University, Shanxi Bethune Hospital, Shanxi Academy of Medical Sciences, No. 99 Longcheng Street, Taiyuan, Shanxi 030032, China; Department of Cardiovascular Medicine, Third Hospital of Shanxi Medical University, Shanxi Bethune Hospital, Shanxi Academy of Medical Sciences, No. 99 Longcheng Street, Taiyuan, Shanxi 030032, China; Department of Cardiovascular Medicine, Third Hospital of Shanxi Medical University, Shanxi Bethune Hospital, Shanxi Academy of Medical Sciences, No. 99 Longcheng Street, Taiyuan, Shanxi 030032, China

**Keywords:** age-standardized DALYs rate, age-standardized mortality rate, atrial fibrillation and flutter, decomposition and frontier analysis, high body mass index

## Abstract

**Background:**

This study evaluates changes in the burden of high body mass index (BMI) and its impact on atrial fibrillation and flutter (AF/AFL) using the 2021 Global Burden of Disease database.

**Methods:**

Mortality and disability-adjusted life years rates were analysed globally, considering age, sex, geography and the Socio-demographic Index (SDI). Decomposition and frontier analyses assessed the impact of epidemiological drivers and SDI on the burden. The nordpred model validated the predictions.

**Results:**

The burden of high BMI is now 2.5 times greater than 30 y ago and will continue to increase over the next 20 y, heavily impacting cardiovascular and metabolic diseases. High BMI–related AF/AFL also poses a significant burden, especially in developed regions. Men's burden grows faster than women's. Decomposition analysis shows epidemiological changes mainly drive burden increases, while in women, population growth is more significant. Frontier analysis indicates that the gap between actual burden and theoretical optimal value has widened with increasing SDI, except in countries such as Bulgaria and the Czech Republic.

**Conclusions:**

Over the past 30 y, the overall burden of high BMI and its impact on AF/AFL have increased significantly, highlighting the need for targeted public health strategies.

## Introduction

Atrial fibrillation and flutter (AF/AFL), the most common rapid cardiac arrhythmias, are globally prevalent. Their incidence and prevalence are increasing due to longer average lifespans and an increase in chronic disease patients, positioning them as significant challenges in 21st-century cardiovascular disease (CVD) management.^1^ According to the Global Burden of Disease (GBD) study, the number of AF/AFL patients reached approximately 46.3 million in 2016, marking a threefold increase over the past 50 y.^2^ Despite advances in treating AF/AFL, its high readmission rate and serious complications such as myocardial infarction, heart failure, stroke and death remain major global public health challenges.^3^

In recent decades, the global prevalence of overweight and obesity has surged. By 2015, an estimated 107.7 million children and 603.7 million adults were diagnosed as obese.^4^ Despite advancements in understanding and managing overweight and obesity, their incidence continues to increase.^5^ Epidemiological studies show that a higher body mass index (BMI) increases the risk of chronic diseases such as CVDs, diabetes, chronic kidney disease, multiple cancers and musculoskeletal disorders.^6,7^ This trend poses a serious global public health challenge that urgently requires effective measures.

In the aetiology of AF/AFL, obesity is a recognized risk factor, with BMI being the most common measure. High BMI is a major independent predictor of AF/AFL, accounting for 12–18% of the population-attributable risk.^8^ Subgroup analysis indicates that obese patients have a 20–30% higher risk of AF compared with those with a normal BMI.^9^ Given the significant impact of AF/AFL on global health and its link to high BMI, this study examines the changes in the overall burden of high BMI from 1990 to 2021 and its contribution to AF/AFL trends globally. We analysed 2021 GBD data from various countries and regions to evaluate disability-adjusted life years (DALYs) and mortality rates, informing targeted health policies and resource distribution. Decomposition and frontier analyses elucidated the epidemiological contributors and disparities between theoretical and actual burdens. These insights enhance evidence-based policy and resource optimization.

## Methods

### Data sources and collection

Data on overall high BMI and its associated AF/AFL deaths and DALYs from 1990 to 2021 were obtained from the Global Health Data Exchange query tool (https://ghdx.healthdata.org). We selected ‘all countries and regions’ or ‘GBD regions’ as locations, ‘all causes’ and ‘atrial fibrillation and flutter’ as causes, ‘high body mass index’ as the risk factor, ‘DALYs’ and ‘deaths’ as metrics, measured by ‘number’ and ‘rate’. Gender categories included ‘male,’ ‘female’ and ‘both’, with age grouped in 5-y intervals.

### Assessment of burden

Data on high BMI exposure were obtained from a systematic review and report covering 2022 studies across 190 countries.^10^ To assess the impact of high BMI on the burden of AF/AFL, the comparative risk assessment framework was used to calculate population attributable fractions. Additionally, Bayesian meta-regression and spatiotemporal Gaussian process regression were applied to evaluate relative risks and exposure levels.^11,12^

### Statistical analyses

Age-standardized rate (ASR) and estimated annual percentage change (EAPC) are used to quantify trends in DALYs and mortality rates for AF/AFL. The ASR was calculated as ASR=Σ_i_Aa_i_w_i_/Σ_i_Aw_i_×100 000, where A is the total number of age groups, a_i_ is the age-specific rate for age group i and w_i_ is the standard population weight for age group i. The EAPC, a measure of ASR trend over time, was determined by fitting a regression line to the natural logarithm of the ASR (y=ln[ASR]) against the calendar year (x), with the equation y=α+βx+e. The EAPC was calculated as 100×(exp[β]−1), with 95% uncertainty intervals (UIs) obtained from the linear regression model. We used Das Gupta's decomposition method to break down changes in ASRs for AF/AFL into the effects of aging structure, population growth and epidemiological changes^13^ and used frontier analysis to identify the gap between actual ASRs and theoretical optimal values at different development levels.^14^ The Nordpred model package were used to validate the prediction results. All statistical analyses and graphical visualizations were performed using R version 4.3.2 (R foundation for Statistical Computing, Vienna, Austria).

## Results

### Analysis of the overall impact and trends of high BMI across countries, GBD regions, SDI, gender and age

**Figure 1.**
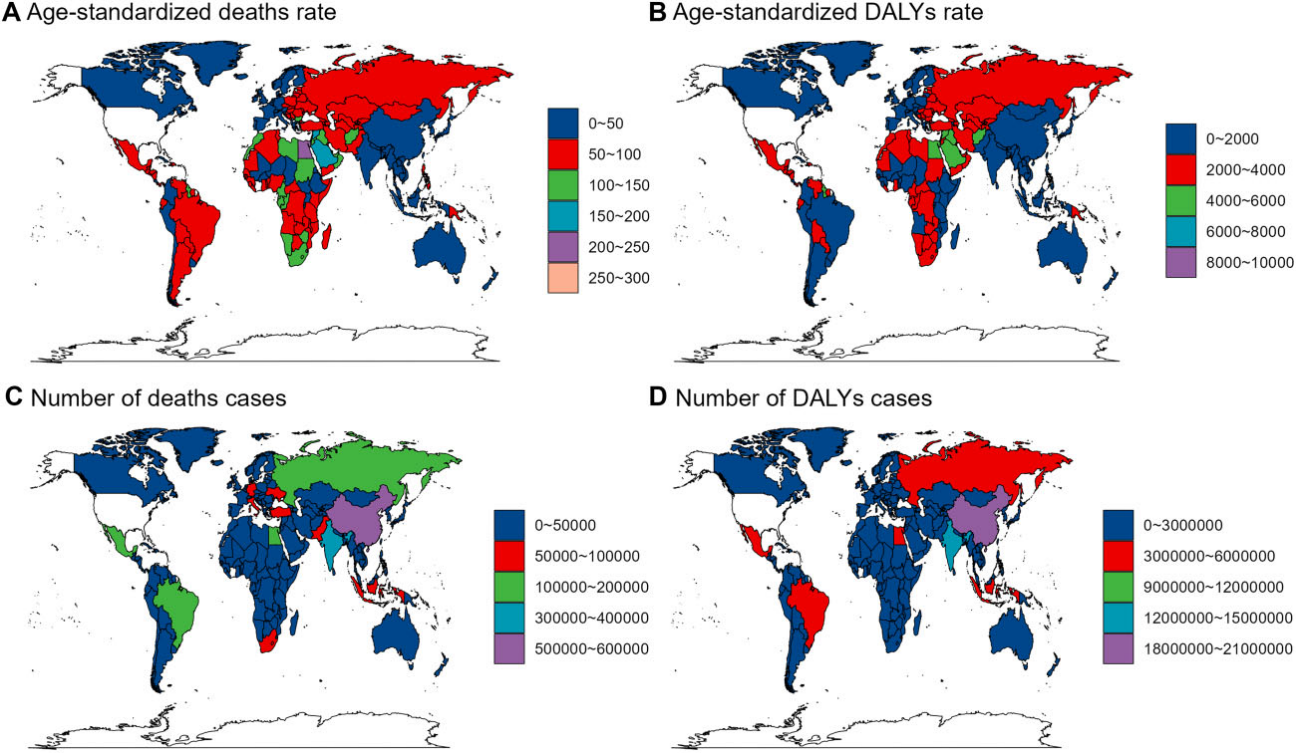
Overall burden attributable to high BMI across 204 countries and territories in 2021. **(A)** ASMR in 2021, **(B)** ASDR in 2021, **(C)** number of deaths in 2021 and **(D)** number of DALYs in 2021.

In 2021, global deaths and DALYs due to high BMI were 3 709 063 and 12 852 083, respectively, a 2.6-fold increase since 1990. The age-standardized mortality rate (ASMR) and DALYs rate (ASDR) were 44.23 per 100 000 (95% UI 22.01 to 67.64) and 1493.24 per 100 000 (95% UI 648.2 to 2350.72), reflecting a steady 30-y increase (Supplementary Figure S1 and Tables S1 and S2). Among 204 countries, Nauru and Fiji had the highest ASMRs and ASDRs in 2021, at 296.35 (95% UI 135.41 to 454.58) and 291.64 (95% UI 137.36 to 433.79) per 100 000 for ASMR and 9922.73 (95% UI 4581.83 to 14 859.93) and 8343.95 (95% UI 4069.55 to 12 130.29) per 100 000 for ASDR. Japan recorded the lowest rates, with an ASMR of 9.71 (95% UI 4.84 to 16.08) and an ASDR of 588.23 (95% UI 219.54 to 995.7) per 100 000 (Figure 1; Supplementary Tables S1 and S2). Zimbabwe had the largest increase in estimated annual percentage change (EAPC), at 3.98% (95% UI 3.34 to 4.61) for ASMR and 3.95% (95% UI 3.33 to 4.57) for ASDR, while Norway (−2.17% [95% UI −2.25 to −2.1]) and Ethiopia (−1.81% [95% UI −2.02 to −1.6]) showed the most significant declines (Supplementary Figure S2 and Tables S1 and S2).

**Figure 2.**
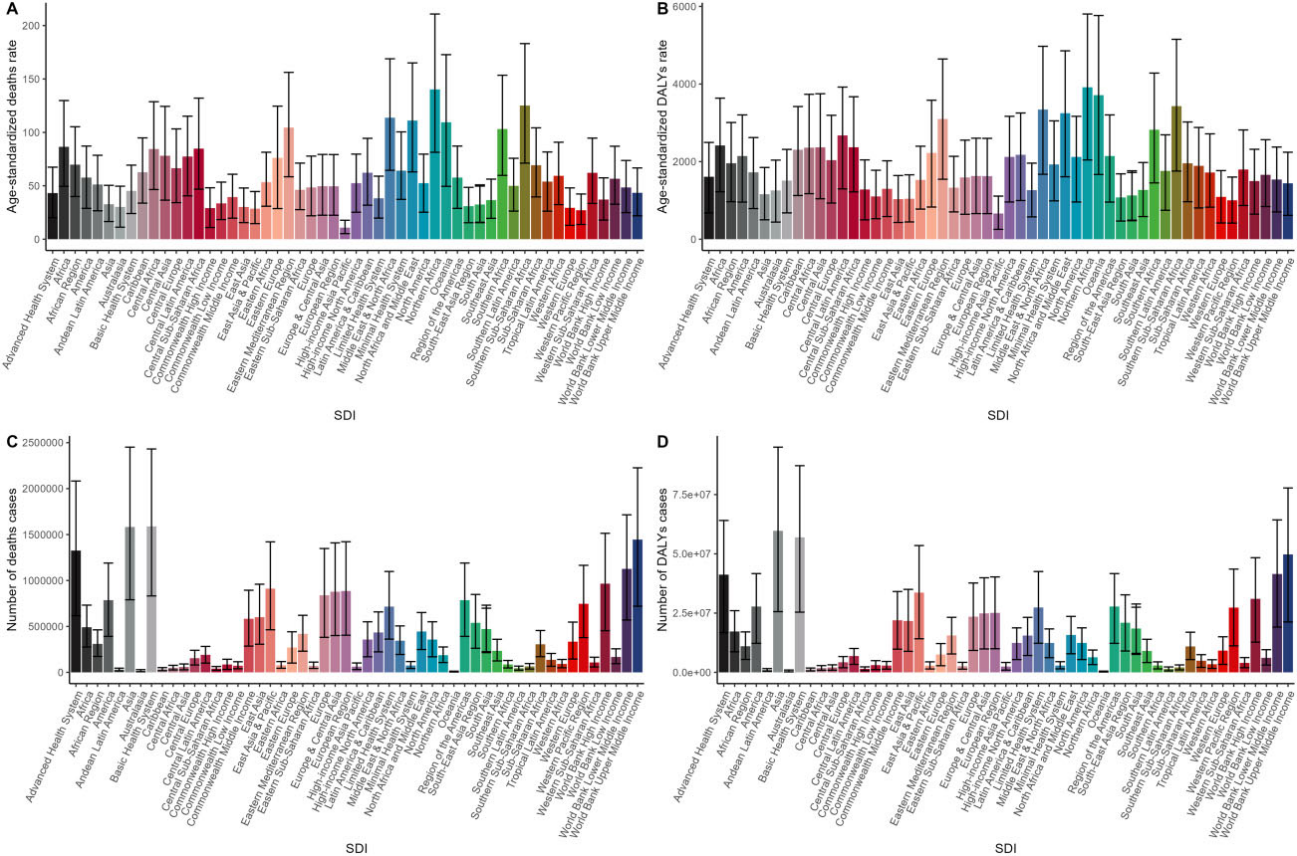
Overall burden attributable to high BMI across GBD regions in 2021. **(A)** ASMR in 2021, **(B)** ASDR in 2021, **(C)** number of deaths in 2021 and **(D)** number of DALYs in 2021.

In the GBD regional classification, Northern Africa had the highest ASMR and ASDR in 2021, at 140.23 (95% UI 81.34 to 210.83) and 3916.41 (95% UI 2043.36 to 5804.41) per 100 000. The high-income Asia-Pacific region showed the best performance, with ASMR at 10.83 (95% UI 5.23 to 17.74), ASDR at 662.64 (95% UI 253.69–1110.48) per 100 000 and EAPC at −1.25% (95% UI −1.42 to −1.09) for ASMR. The Western Pacific Region ranked second, with ASMR at 27.11 (95% UI 13.78 to 42.32) and ASDR at 1002.14 (95% UI 415.71 to 1601.08) per 100 000 (Figure 2; Supplementary Tables S1 and S2). Regions with the highest EAPC increases in ASMR and ASDR were East Asia and Pacific (2.78%) and South Asia (2.35%) (Supplementary Figure S3 and Tables S1 and S2). In 2021, ASMR and ASDR peaked in the low-middle SDI category, with the largest 30-y increases. The lowest ASMR was in the high SDI category, showing a marked decline, while the lowest ASDR was in the low SDI category (Supplementary Figures S4 and S5 and Tables S1 and S2).

**Figure 3.**
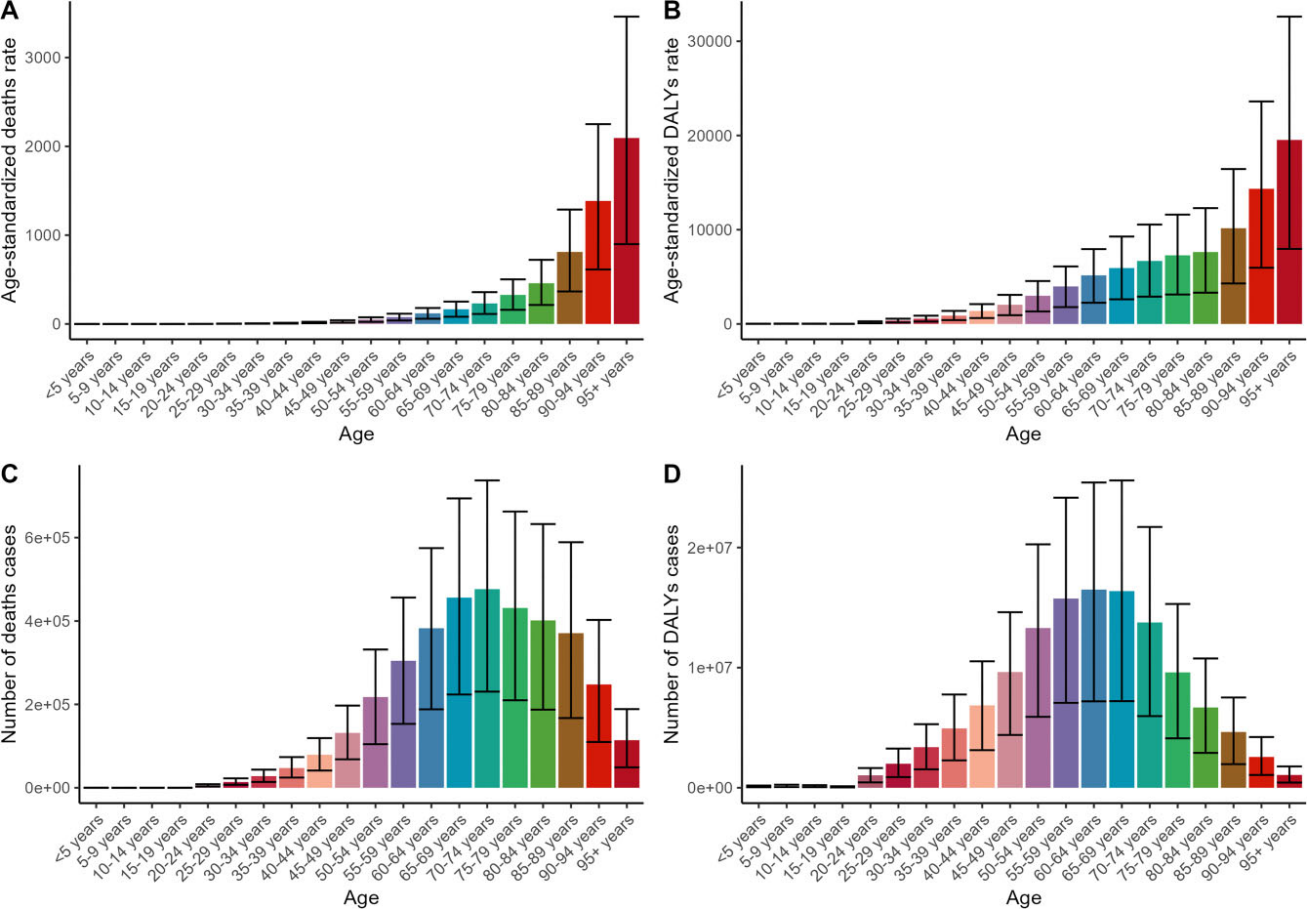
Overall burden attributable to high BMI by different age groups in 2021. **(A)** ASMR in 2021, **(B)** ASDR in 2021, **(C)** number of deaths in 2021 and **(D)** number of DALYs in 2021.

Overall, the relationship between gender and high BMI in 2021 showed that, in terms of deaths and DALYs, females outnumber males. However, in age-standardized rates (ASR), males have slightly higher rates than females, with a significantly greater increase in ASR for males over the past 30 y. Notably, the increase in ASMR for males was nearly 40 times that for females (Supplementary Figures S6 and S7 and Tables S1 and S2). In terms of age distribution, deaths and DALYs were primarily concentrated in the 60- to 80-y age group, where the case count peaked. ASR changes have shown a steady upward trend, reaching the highest levels in the ≥95-y age group (Figure 3), with the most substantial increases observed in the 20- to 30-y age group (Supplementary Figure S8 and Tables S1 and S2).

### The impact of high BMI on overall disease

**Figure 4.**
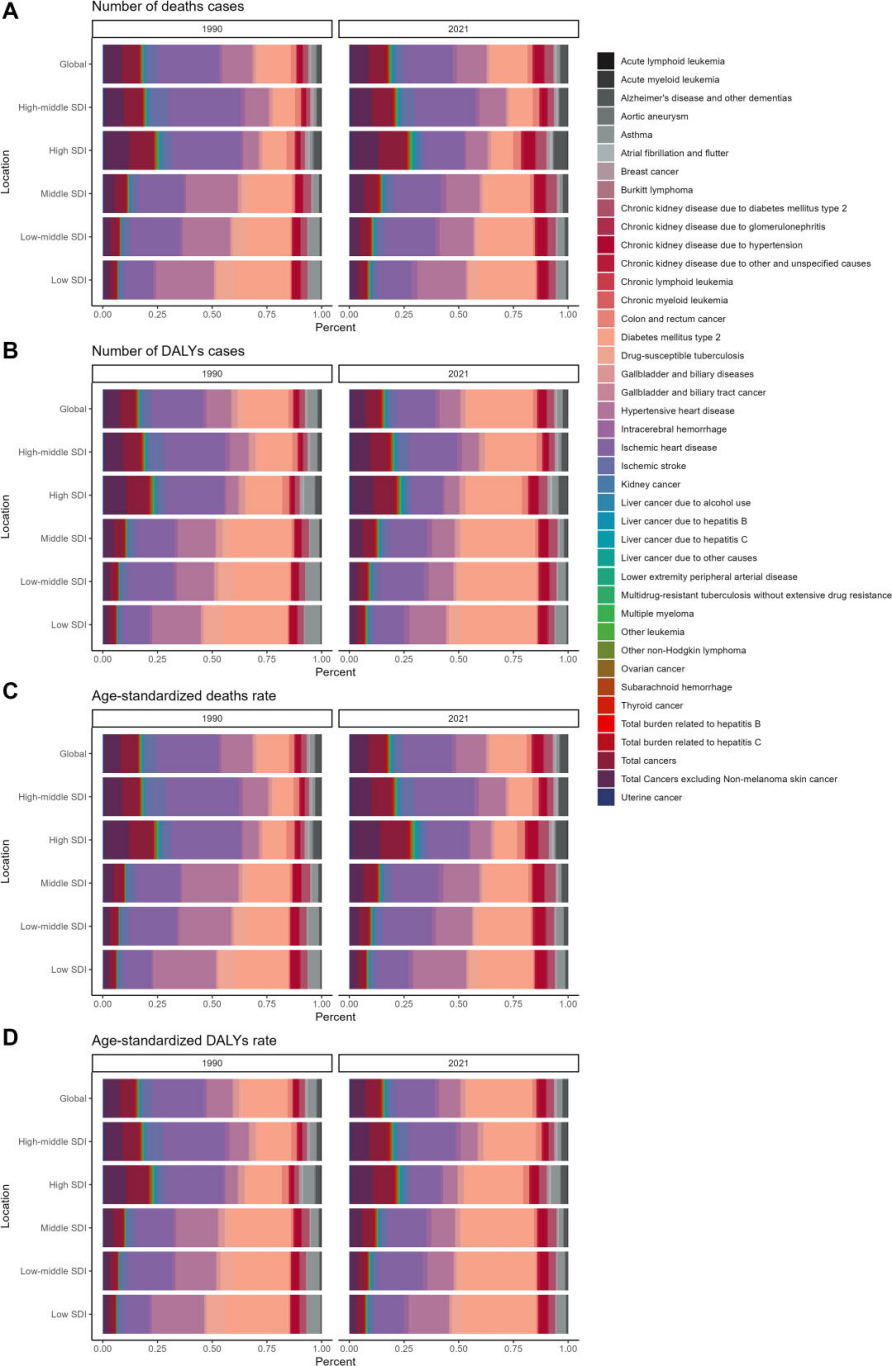
Percentage composition of the impact of high BMI on disease. **(A)** The number of deaths globally and in five SDI regions. **(B)** The number of DALYs cases in global and five SDI regions. (**C**) The ASMR in global and five SDI regions. **(D)** The ASDR globally and five SDI regions.

The 2021 analysis of the impact of high BMI on global disease burden shows that ischaemic heart disease accounted for the highest proportion of BMI-related deaths and mortality rate, at 22.15% and 22.17%, respectively. For DALYs, type 2 diabetes mellitus was the largest contributor, representing 29.25% of cases and 29.48% of the rate. Other significantly affected diseases included hypertensive heart disease, chronic kidney disease due to hypertension, chronic kidney disease due to type 2 diabetes mellitus, ischaemic stroke, Alzheimer's disease and other dementias, colon and rectal cancer and intracerebral haemorrhage, with proportions ranging from 3% to 20%. In contrast, diseases such as Burkitt lymphoma, multidrug-resistant tuberculosis without extensive drug resistance and chronic myeloid leukaemia were minimally impacted, each contributing <0.01%. When categorized by SDI, the diseases most affected by high BMI—from low to high SDI regions—closely followed global trends, predominantly affecting cardiovascular and cerebrovascular diseases, diabetes and its renal complications and certain cancers such as colon, rectal and breast cancer. Among all disease categories, blood disorders were least affected by high BMI, with an overall share of <1 in 10 000 (Figure 4; Supplementary Table S3).

### Changes in the global burden of AF/AFL attributed to high BMI from 1990 to 2021

**Figure 5.**
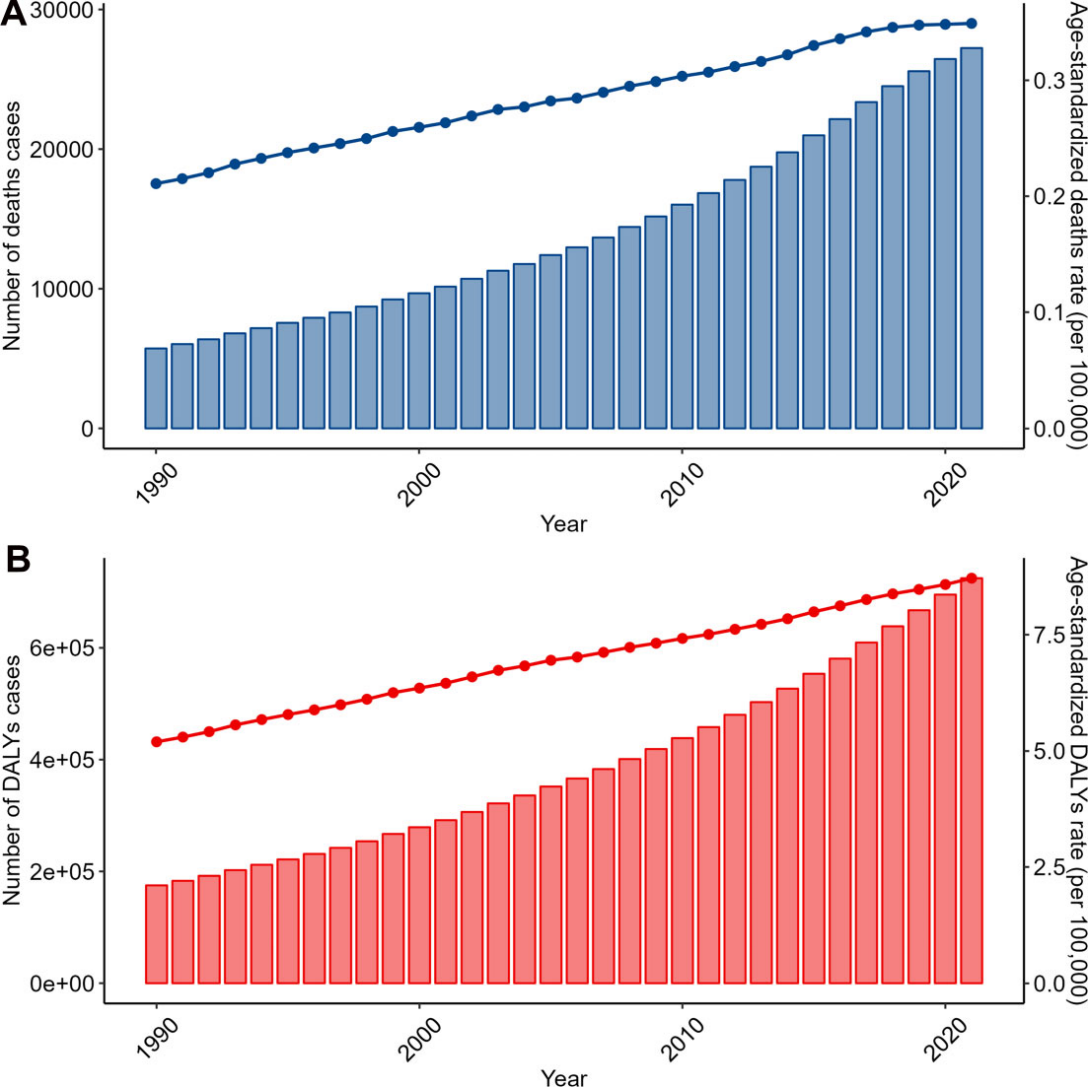
Trends in the global burden of AF/AFL attributed to high BMI from 1990 to 2021. **(A)** Changes in the number of deaths and ASMR. **(B)** Changes in the number of DALYs and ASDR.

In 2021, there were 27 237 deaths from AF/AFL due to high BMI, a 376% increase from 1990. The ASMR rose from 0.21 to 0.35 per 100 000, with an EAPC of 1.65% (Figure 5A, Table 1). DALYs reached 724 574, up 314% from 1990, and the ASDR was 8.71 per 100 000, with an EAPC of 1.64% (Figure 5B, Table 2).

**Table 1.** Cases and age-standardized values for AF/AFL deaths and their EAPCs from 1990 to 2021 at the global and regional levels

Region	1990, n (95% UI	ASMR 1990, (95% UI)	2021, n (95% UI)	ASMR 2021 (95% UI)	EAPC, % (95% UI)
Global	5722 (2352 to 9912)	0.21 (0.09 to 0.36)	27 237 (11 747 to 46 605)	0.35 (0.15 to 0.6)	1.65 (1.6 to 1.71)
Andean Latin America	20 (8 to 38)	0.12 (0.04 to 0.22)	182 (73 to 330)	0.33 (0.13 to 0.6)	3.31 (3.15 to 3.48)
Australasia	97 (38 to 182)	0.46 (0.18 to 0.87)	563 (233 to 1041)	0.86 (0.36 to 1.59)	2.13 (1.89 to 2.38)
Caribbean	38 (16 to 62)	0.18 (0.08 to 0.3)	222 (99 to 363)	0.39 (0.18 to 0.65)	2.42 (2.32 to 2.52)
Central Asia	60 (25 to 102)	0.16 (0.07 to 0.28)	163 (71 to 276)	0.27 (0.12 to 0.46)	1.47 (1.24 to 1.7)
Central Europe	570 (246 to 977)	0.48 (0.21 to 0.82)	1462 (651 to 2582)	0.6 (0.27 to 1.06)	0.86 (0.62 to 1.09)
Central Latin America	156 (63 to 286)	0.26 (0.1 to 0.47)	1244 (551 to 2211)	0.54 (0.24 to 0.96)	2.39 (2.3 to 2.49)
Central SSA	5 (2 to 11)	0.03 (0.01 to 0.08)	67 (25 to 136)	0.21 (0.07 to 0.42)	5.88 (5.69 to 6.06)
East Asia	58 (13 to 133)	0.01 (0 to 0.03)	2544 (995 to 4446)	0.16 (0.06 to 0.28)	7.96 (7.7 to 8.21)
Eastern Europe	784 (331 to 1304)	0.36 (0.16 to 0.6)	2283 (980 to 3917)	0.63 (0.27 to 1.09)	1.69 (1.52 to 1.87)
Eastern SSA	8 (2 to 18)	0.02 (0 to 0.04)	91 (34 to 171)	0.08 (0.03 to 0.15)	5.25 (5.13 to 5.36)
High-income Asia-Pacific	31 (9 to 69)	0.02 (0 to 0.04)	342 (122 to 652)	0.05 (0.02 to 0.1)	2.83 (2.5 to 3.15)
High-income North America	1240 (503 to 2412)	0.34 (0.14 to 0.66)	5732 (2425 to 9904)	0.76 (0.32 to 1.31)	2.5 (2.35 to 2.66)
North Africa and Middle East	249 (99 to 441)	0.24 (0.09 to 0.43)	1660 (703 to 2811)	0.56 (0.24 to 0.96)	2.91 (2.68 to 3.15)
Oceania	3 (1 to 6)	0.16 (0.06 to 0.35)	14 (6 to 25)	0.27 (0.12 to 0.49)	1.52 (1.44 to 1.59)
South Asia	30 (8 to 66)	0.01 (0 to 0.02)	823 (345 to 1507)	0.08 (0.03 to 0.15)	8.07 (7.86 to 8.29)
Southeast Asia	22 (4 to 47)	0.01 (0 to 0.03)	508 (194 to 902)	0.11 (0.04 to 0.2)	7.61 (7.32 to 7.9)
Southern Latin America	96 (40 to 177)	0.26 (0.1 to 0.47)	417 (176 to 758)	0.45 (0.19 to 0.81)	2.65 (2.23 to 3.07)
Southern SSA	36 (16 to 63)	0.19 (0.08 to 0.34)	207 (94 to 342)	0.57 (0.26 to 0.94)	3.51 (3.11 to 3.91)
Tropical Latin America	115 (44 to 208)	0.19 (0.07 to 0.35)	1266 (509 to 2201)	0.53 (0.21 to 0.92)	3.45 (3.19 to 3.7)
Western Europe	2049 (862 to 3497)	0.35 (0.15 to 0.6)	7145 (2949 to 12 840)	0.55 (0.23 to 1)	1.68 (1.58 to 1.77)
Western SSA	50 (18 to 93)	0.1 (0.04 to 0.19)	305 (127 to 522)	0.3 (0.12 to 0.51)	3.08 (2.92 to 3.23)

**Table 2.** Cases and age-standardized values for AF/AFL DALYs and their EAPCs from 1990 to 2021 at the global and regional levels

Region	1990, n (95% UI)	ASDR 1990, n (95% UI)	2019, n (95% UI)	ASDR 2019, n (95% UI)	EAPC, % (95% UI)
Global	615 332 (288 803 to 1 068 931)	17.8 (8.46 to 31.18)	1 768 682 (934 503 to 2 911 771)	22.46 (11.86 to 37.08)	0.92 (0.88 to 0.97)
Andean Latin America	1880 (867 to 3271)	10.33 (4.63 to 18.3)	8707 (4827 to 13 932)	16.21 (8.96 to 26.02)	1.8 (1.72 to 1.88)
Australasia	9732 (4928 to 16 437)	42.13 (21.33 to 71.11)	26 757 (15 003 to 42 263)	50.79 (28.4 to 80.54)	0.61 (0.55 to 0.67)
Caribbean	3176 (1575 to 5446)	13.22 (6.53 to 22.91)	10 003 (5485 to 16 320)	19.25 (10.56 to 31.41)	1.49 (1.37 to 1.6)
Central Asia	10 147 (4946 to 17 413)	23.8 (11.67 to 41.02)	23 001 (12 539 to 37 370)	37.98 (20.78 to 61.11)	1.69 (1.6 to 1.77)
Central Europe	50 291 (26 687 to 82 492)	35.44 (18.8 to 57.27)	97 650 (55 294 to 154 077)	44 (24.88 to 69.18)	0.8 (0.74 to 0.87)
Central Latin America	11 042 (5400 to 18 806)	15.22 (7.39 to 26.14)	48 606 (26 401 to 77 893)	21.51 (11.62 to 34.55)	1.12 (1.06 to 1.19)
Central SSA	1728 (692 to 3414)	9.51 (3.61 to 19.53)	5075 (2303 to 9220)	11.81 (5.22 to 22.03)	0.31 (−0.11 to 0.73)
East Asia	42 365 (9706 to 101 657)	6.1 (1.34 to 14.86)	235 350 (90 584 to 458 430)	12.24 (4.71 to 24.03)	2.5 (2.41 to 2.59)
Eastern Europe	82 371 (42359 to 135 143)	31.07 (16.15 to 50.81)	155 996 (87276 to 247 371)	44.24 (24.74 to 70.04)	1.27 (1.19 to 1.35)
Eastern SSA	2760 (888 to 5969)	4.67 (1.45 to 10.43)	12 512 (5745 to 22 476)	9.5 (4.24 to 17.54)	2.72 (2.47 to 2.97)
High-income Asia-Pacific	12 001 (3712 to 24 865)	6.29 (1.93 to 13.06)	29 469 (10510 to 58 115)	6.2 (2.25 to 12.07)	−0.48 (−0.64 to 0.32)
High-income North America	122 854 (60 936 to 209 385)	33.91 (16.8 to 57.47)	335 889 (188 185 to 527 631)	51.22 (28.61 to 80.31)	1.89 (1.72 to 2.06)
North Africa and Middle East	23 052 (11 938 to 38 593)	16.48 (8.44 to 27.81)	88 927 (51 035 to 139 689)	24.34 (14.14 to 37.99)	1.29 (1.25 to 1.32)
Oceania	391 (172 to 715)	15.09 (6.12 to 28.78)	1161 (553 to 2063)	18.53 (8.41 to 33.55)	0.57 (0.45 to 0.69)
South Asia	20 853 (6266 to 46 234)	4.72 (1.36 to 10.59)	158 452 (75634 to 277 778)	12.77 (6.06 to 22.67)	3.54 (3.35 to 3.72)
Southeast Asia	10 935 (3209 to 24 729)	4.95 (1.4 to 11.52)	74 145 (34360 to 131 308)	13.56 (6.19 to 24.26)	3.69 (3.64 to 3.74)
Southern Latin America	7340 (3346 to 12 893)	17.15 (7.79 to 30.26)	22 466 (11700 to 36 724)	26.24 (13.62 to 42.91)	1.41 (1.28 to 1.54)
Southern SSA	3863 (2130 to 6100)	16.14 (8.72 to 25.44)	11 342 (6740 to 16 886)	23.94 (14.14 to 35.68)	1.52 (1.41 to 1.64)
Tropical Latin America	12 860 (5936 to 22 853)	16.63 (7.54 to 29.97)	65 194 (36989 to 102 970)	28.23 (15.96 to 44.55)	2.34 (2.16 to 2.52)
Western Europe	180 660 (89 291 to 309 329)	30.44 (14.9 to 52.04)	337 415 (177 853 to 554 851)	33.76 (17.74 to 55.38)	0.46 (0.39 to 0.53)
Western SSA	5031 (1895 to 9754)	7.19 (2.62 to 14.21)	20 566 (10 325 to 34 207)	14.05 (6.99 to 23.7)	2.25 (2.22 to 2.29)

In 2021, among 21 regions analysed in the GBD study, Australasia had the highest ASMR for AF/AFL due to high BMI (0.86 [95% UI 0.36 to 1.59]), followed by high-income North America (0.76 [95% UI 0.32 to 1.31]), Eastern Europe (0.63 [95% UI 0.27 to 1.09]) and Central Europe (0.6 [95% UI 0.27 to 1.06]) (Table 1). High-income North America recorded the highest ASDR (23.85 [95% UI 10.09 to 40.69]), followed by Australasia (21.4 [95% UI 9.07 to 37.72]) and Eastern Europe (16.43 [95% UI 6.97 to 28.51]) (Table 2). The lowest ASMR and ASDR were in the high-income Asia-Pacific region, at 0.05 and 1.77 per 100 000, respectively (Tables 1 and 2). From 1990 to 2021, Southeast Asia saw the largest ASMR increase (8.07% [95% UI 7.86 to 8.29]) and East Asia the largest ASDR increase (8.24% [95% UI 8.07 to 8.41]), while Central Europe had the lowest ASMR and ASDR (0.86 [95% UI 0.62 to 1.09] and 0.85 [95% UI 0.70 to 1.00], respectively) (Tables 1 and 2).

**Figure 6.**
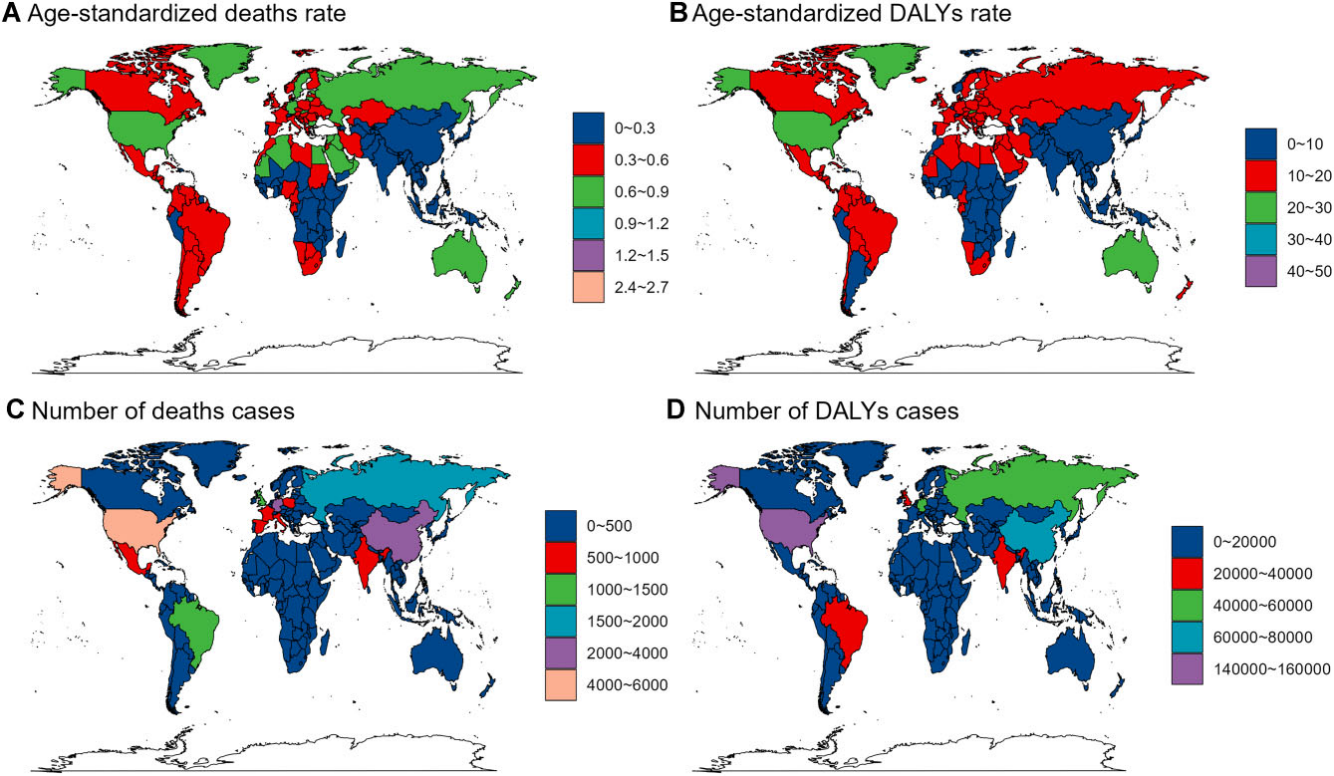
Burden of AF/AFL attributed to high BMI across 204 countries and territories in 2021. **(A)** ASMR in 2021, **(B)** ASDR in 2021, **(C)** number of deaths in 2021 and **(D)** number of DALYs in 2021.

**Figure 7.**
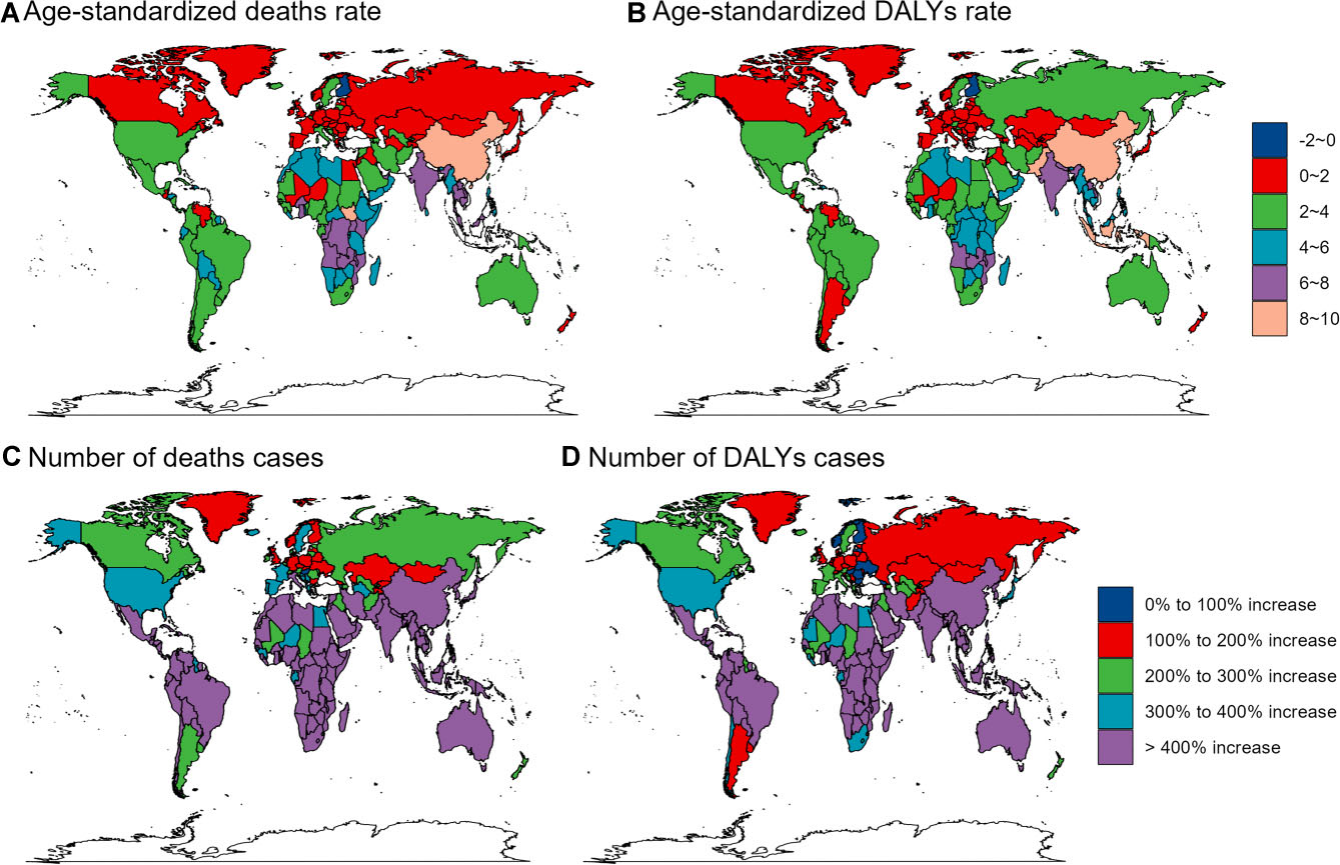
Trends in the burden of AF/AFL attributed to high BMI across 204 countries and territories from 1990 to 2021. **(A)** The ASMR between 1990 and 2021. **(B)** The ASDR between 1990 and 2021. **(C)** The number of deaths between 1990 and 2021. **(D)** The number of DALYs between 1990 and 2021.

In 2021, Montenegro had the highest ASMR and ASDR for AF/AFL due to high BMI (2.72 [95% UI 1.14 to 4.89] and 44.18 [95% UI 18.82 to 77.58], respectively), while Timor-Leste had the lowest (0 [95% UI 0 to 0.01] and 0.25 [95% UI 0 to 0.59], respectively) (Figure 6A, B; Supplementary Tables S4 and S5). The United States (deaths: 5278 [95% UI 2228 to 9082], DALYs: 152 786 [95% UI 64 970 to 258 787]) and China (deaths: 2378 [95% UI 942 to 4212, DALYs: 75 252 [95% UI 29 280 to 129 907]) had the highest death and DALY counts from high BMI–related AF/AFL (Figure 6C, D; Supplementary Tables S4 and S5). From 1990 to 2021, Timor-Leste saw the greatest percentage increase in AF/AFL burden (ASMR 84.6% [95% UI 41.8 to 140.31], ASDR 28.88% [95% UI 21.69 to 36.5]), while Guam (−1.16% [95% UI −1.63 to 0.69]) and Finland (−0.23% [95% UI −0.44 to −0.02]) had the largest decreases in ASRs (Figure 7A, B; Supplementary Tables S4 and S5). Over the past 30 y, China, India, Southeast Asia, much of Africa and Brazil showed the largest increases in burden of AF/AFL, exceeding 300% and reaching >400%, while the smallest increases occurred mostly in Europe (Figure 7C, D; Supplementary Tables S4 and S5).

At the SDI level, results show that high-SDI regions recorded the highest numbers of deaths, ASMR, DALYs and ASDR, while low-SDI regions had the lowest figures (Supplementary Figure S9 and Tables S4 and S5). From 1990 to 2021, the low-SDI regions had the largest increases in both ASMR (EAPC 4.49%) and ASDR (EAPC 4.22%), while the high-middle SDI regions showed the smallest growth in ASRs, with EAPCs of 1.47% and 1.28%, respectively (Supplementary Figure S10 and Tables S4 and S5). Furthermore, in the upper mid-range of SDI (0.5–0.9), certain countries and regions—such as Montenegro, Nauru, American Samoa and Dominica—had ASDR and ASMR values much higher than expected, ranking them among the highest globally. Conversely, countries such as Japan, Singapore and the Republic of Korea had ASDR and ASMR values significantly lower than expected (Supplementary Figure S11).

A gender-based analysis showed that although females had higher deaths, DALYs and ASRs than males (Supplementary Figure S12), the burden rose more sharply for males (Supplementary Figure S13). Male deaths increased nearly 500%, 1.51 times the increase observed in females, with an EAPC of 2.53%, which is 1.84 times that of females (0.77%). For DALYs, male cases increased by 382%, 1.39 times the increase in females, with an EAPC of 2.32%, nearly 1.75 times that of females (1.32%) (Supplementary Tables S4 and S5). By age, global ASMR and ASDR for AF/AFL due to high BMI peaked in the 85- to 89-y age group for deaths and the 70- to 74-y age group for DALYs (Supplementary Figure S14). From 1990 to 2021, the 30- to 34-y age group had the highest EAPC in ASRs at 3.23% and 3.08%. The largest increases in deaths and DALYs were in the ≥95-y age group, rising about 800% and 760%, respectively, while the slowest growth occurred in the 75- to 79-y and 70- to 74-y age groups (Supplementary Figure S15 and Tables S4 and S5).

### Epidemiological drivers: the contribution of aging, population growth and epidemiological changes on the burden of AF/AFL caused by high BMI

**Figure 8.**
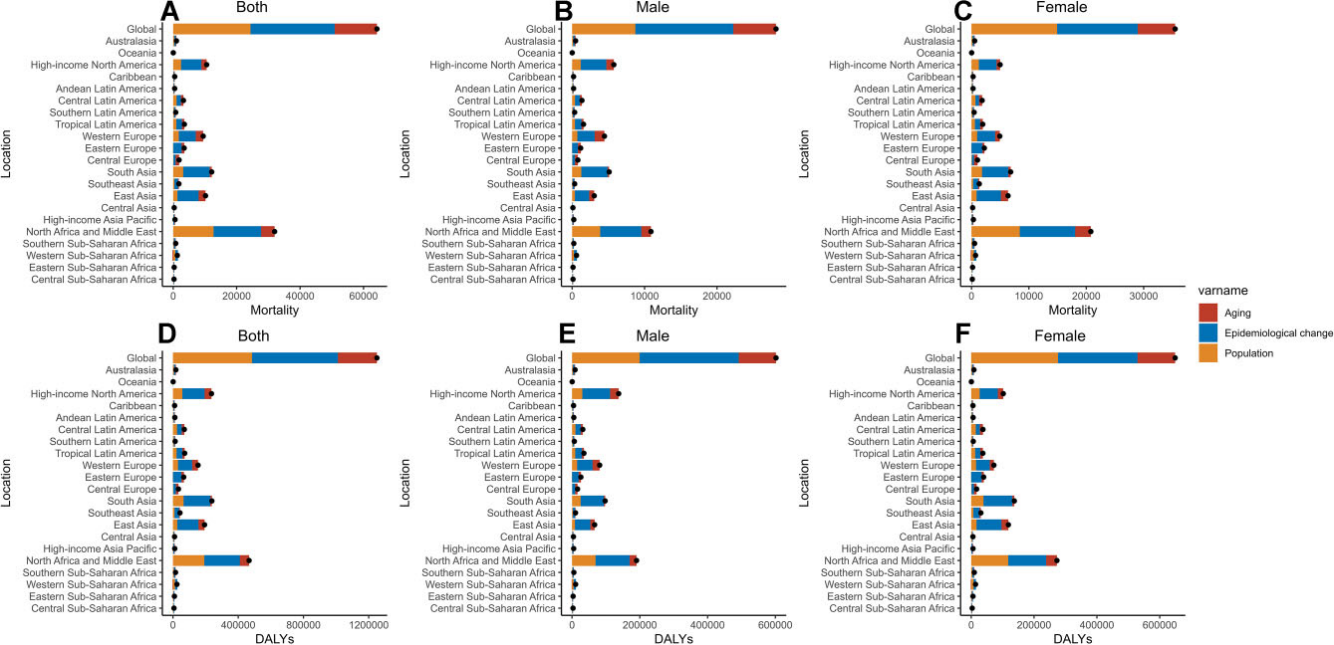
Epidemiological drivers: the contribution of aging, population growth and epidemiological changes to the burden of AF/AFL caused by high BMI in GBD regions. **(A)** Epidemiological drivers in deaths for both genders. **(B)** Epidemiological drivers in deaths for males. **(C)** Epidemiological drivers in deaths for females. **(D)** Epidemiological drivers in DALYs for both genders. **(E)** Epidemiological drivers in DALYs for males. **(F)** Epidemiological drivers in DALYs for females.

We assessed the impact of population growth, aging and epidemiological changes on these indicators. Epidemiological changes were the primary contributors to the increased burden globally and in most regions, except among females, where population growth had a greater impact (Figure 8; Supplementary Table S6). The impact of population growth showed a declining trend across all sex strata in Eastern Europe, suggesting a mitigating effect on the disease burden; in contrast, it was the largest contributor to disease burden in Oceania (Figure 8; Supplementary Table S6). Aging was a key factor in disease burden, showing a notable negative trend in central sub-Saharan Africa (SSA), eastern SSA and western SSA, while significantly increasing the burden in Central Europe (Figure 8; Supplementary Table S6).

### Frontier analysis of the association between deaths and DALYs from AF/AFL and the level of national development

**Figure 9.**
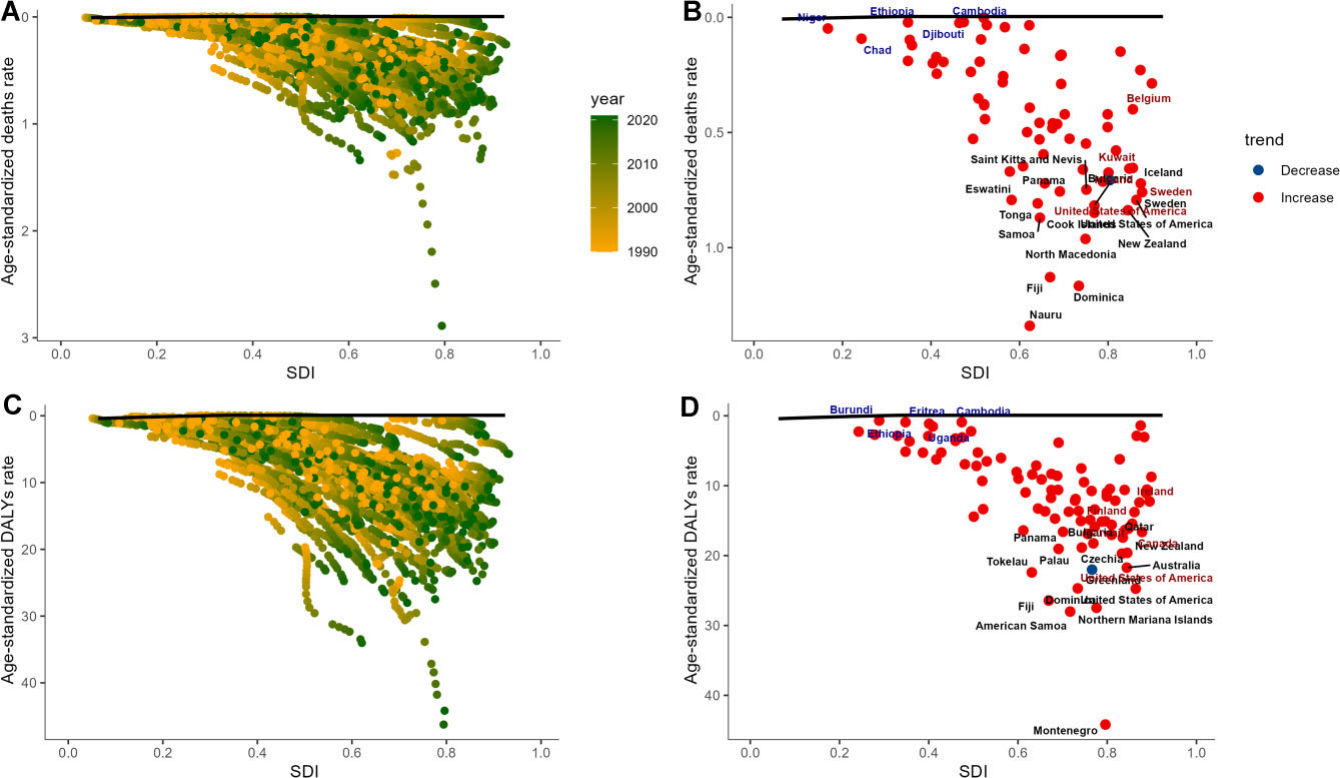
Frontier analysis of deaths and DALYs of AF/AFL attributed to high BMI based on SDI. **(A)** ASMR from 1990 to 2021, **(B)** SDI and ASMR in 2021, **(C)** ASDR from 1990 to 2021, **(D)** SDI and ASDR in 2021. Colour scale represents the years from 1990 to 2021, solid black colour delineates the frontier, dots represent countries and territories.

Results showed that the gap between actual ASR values and the theoretical optimal performance widened as SDI increased (Figure 9). In the ASMR analysis, countries with an SDI of 0.6–0.9, such as Nauru, Dominica, Fiji, Cook Islands, North Macedonia, Samoa and New Zealand, showed a widening gap from optimal performance, with this gap reaching higher levels (Figure 9B). Conversely, only a few countries, such as Bulgaria, showed a narrowing gap. In the ASDR analysis, countries such as Montenegro, American Samoa, Northern Mariana Islands, Australia, New Zealand and the USA showed a widening gap and ranked among the highest, while countries such as the Czech Republic tended to narrow the gap toward optimal performance (Figure 9D).

### Forecast of the overall burden of high BMI

**Figure 10.**
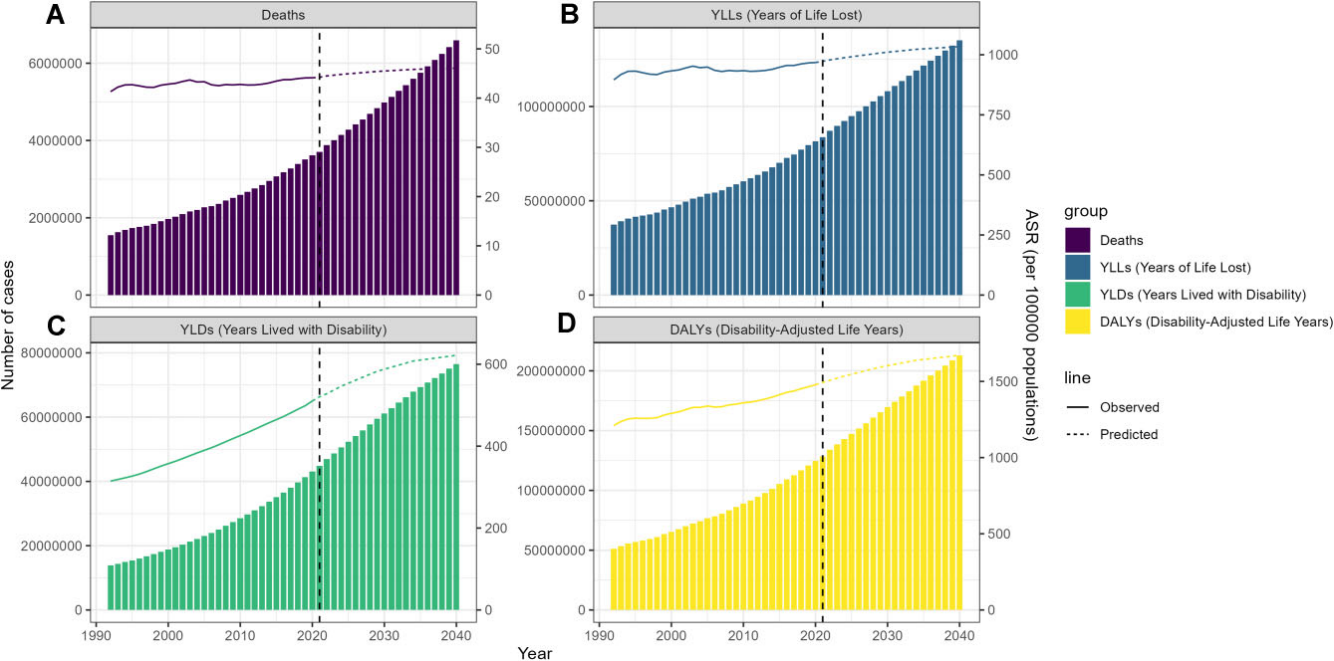
Forecast of the overall burden of high BMI. **(A)** Forecasts of age-standardized mortality rate and cases. **(B)** Forecasts of age-standardized YLL rate and cases. **(C)** Forecasts of age-standardized YLD rate and cases. **(D)** Forecasts of ASDR and cases.

According to the nordpred model, the health burden from high BMI is projected to increase significantly by 2040. Deaths are expected to reach 6 591 317, with an ASMR of 46.09 per 100 000, while DALYs are predicted to increase to 212 946 003, or 1671.12 per 100 000. Components of DALYs, including years of life lost (YLLs) and years lived with disability (YLDs), are also expected to increase continuously (Figure 10; Supplementary Table S7).

## Discussion

Over the past 30 y, the burden of both high BMI alone and high BMI–related AF/AFL has sharply increased. These findings underscore a troubling trend: despite progress in global health, high BMI continues to have a substantial impact, posing an increasing challenge in managing obesity-related CVDs.^15^ Our analysis across global and development-level regions shows that AF/AFL deaths and DALYs are primarily concentrated in economically stable areas, particularly in high and upper-middle development regions. In contrast, the high-income Asia-Pacific region has a relatively lower burden from both high BMI alone and high BMI–related AF/AFL. Less developed regions, including lower-middle development areas, have experienced a marked increase in the overall burden from high BMI and high BMI–related AF/AFL. These regional and economic disparities in disease burden likely reflect variations in lifestyle, dietary habits and healthcare systems. A longitudinal study of global BMI data from 1975 to 2016 indicated an overall increase in overweight and obesity, yet the Asia-Pacific region experienced slower growth, maintaining a lower prevalence compared with the global average. This may be attributed to traditional dietary habits, lifestyle choices and heightened health awareness in the region,^16^ explaining its superior performance in addressing the disease burden of high BMI and AF/AFL. In contrast, residents in economically developed higher SDI regions often adopt unhealthy lifestyles such as smoking, drinking and high-fat diets, increasing the risk of high BMI and, consequently, AF/AFL burden. Meanwhile, in lower-middle SDI regions like India, Southeast Asia, most African countries and Brazil, the disease burden might appear modest in static analyses, but dynamic assessments reveal a rapid escalation. This trend correlates with economic constraints, limited healthcare resources and underdeveloped public health systems, which hinder effective disease prevention and control. Therefore, enhancing public health infrastructure and medical services is imperative in these regions to mitigate the increasing disease burden.

In examining ASDR and ASMR variations based on SDI, we found that countries such as Montenegro, American Samoa and Dominic have ASRs significantly higher than expected. Conversely, high-income Asia-Pacific countries such as Japan, Singapore and Korea have lower-than-expected ASRs. These differences may be due to variations in geography, diet and public health policies. For example, Japan and Korea favour fish, shellfish and plant-based foods,^17^ while Montenegro has a diet richer in meat and alcohol. Additionally, public health initiatives in the Asia-Pacific region, such as smoking bans, obesity prevention and high BMI management,^18^ may help reduce the burden of high BMI and related diseases.

Over the past 30 y there has been explosive growth in the burden caused by high BMI alone and high BMI–related AF/AFL among younger populations. Unhealthy behaviours such as consuming high-fat, high-sugar diets, leading sedentary lifestyles and engaging in smoking and drinking are precipitating earlier onset of obesity and overweight, thereby heightening the risk of chronic diseases among young adults.^19^ The ASMR and ASDR are highest in the population ≥95 y of age and this trend is likely attributable to population aging, the accumulation of chronic diseases and the burden of comorbidities. Medical advancements and improved living conditions have extended average lifespans, thereby increasing the proportion of older populations and, consequently, the number of deaths and DALYs in those ≥95 y of age.^20^ As age increases, so does the prevalence of chronic diseases such as CVDs, diabetes and cancer, which increase the disease burden in older populations. Additionally, while the demand for health services increases, limited resources in certain regions restrict access to high-quality medical care, further exacerbating the disease burden.^21^

At the gender level, an interesting finding is that for both high BMI alone and high BMI–related AF/AFL, females have higher deaths, DALYs and age-standardized mortality rates than males. This disparity may be linked to gender-specific factors, particularly the influence of sex hormones on cardiac electrophysiology in women and the increased risk of CVDs due to declining oestrogen levels after menopause.^22^ Studies have shown higher left atrial endocardial fibrosis in female patients (β=0.99±0.56, adjusted p=0.003), highlighting the importance of female gender in atrial myopathy risk factors.^23^ Additionally, women are more susceptible to the impact of high BMI on cardiovascular diseases during specific physiological stages, such as pregnancy, postpartum and while using oral contraceptives,^24^ which may explain the relatively higher burden of high BMI and AF/AFL associated with women. However, over the past 30 y, the burdens of high BMI and AF/AFL due to high BMI have increased more in men than in women. A 14.4-y follow-up study in Copenhagen found that men had a 63% increased age-adjusted risk of AF compared with women (95% UI 55–72).^25^ This trend may be linked to significant changes in men's lifestyles and dietary habits, such as a preference for higher fat and calorie diets and less physical activity. Additionally, men may be more susceptible to CVD risk factors like smoking and drinking,^26^ which collectively contribute to the increase in AF/AFL burden associated with high BMI.

In addition to a basic analysis of high BMI and its impact on AF/AFL, we also examined which diseases are more significantly influenced by high BMI. Our findings indicate that ischaemic heart disease, type 2 diabetes and its complications and certain cancers are the primary contributors to global BMI-related deaths and DALYs. This underscores a strong link between high BMI and cardiovascular and metabolic health risks, aligning with clinical observations. High BMI increases risks of hypertension, insulin resistance, metabolic disorders and inflammation, closely associating it with these conditions. In contrast, the link between high BMI and diseases such as blood disorders remains unclear, possibly due to the complex and only partially understood mechanisms underlying these conditions.

Our decomposition analysis shows that epidemiological changes, driven by increasing high BMI populations, broader diagnostic and treatment coverage and other health risk factors, primarily increase the global AF/AFL disease burden. In women, population growth significantly contributes to the disease burden, particularly among the elderly. Gender-specific health behaviours and risk factors amplify this impact,^27^ suggesting public health policies should include gender-specific strategies for AF/AFL linked to high BMI. In Eastern Europe, population decline and healthcare improvements have resulted in a negative contribution of population growth to disease burden. Conversely, rapid population growth and healthcare resource pressures in Oceania have led to the highest contribution of population growth to disease burden. In central, eastern, and western SSA, a young population and high mortality rates result in a minimal or negative contribution of aging to the disease burden. In Central Europe, aging and chronic diseases drive increased disease burden.

These differences highlight the need for region-specific strategies to address health challenges from aging and population dynamics. Our frontier analysis reveals that with increasing SDI, the disparity between ASMR, ASDR and their ideal levels widens, likely due to enhanced longevity, lifestyle alterations and improved disease detection and reporting in more developed regions. Conversely, countries with medium SDI undergoing transitional economic and health advancements may see a narrowed gap, as these improvements might mitigate the risks linked with lifestyle changes. Variation in disease burden gaps across SDI levels underscores the need for context-specific prevention and control strategies. These findings reveal differences among countries in managing high BMI–related AF/AFL and underscore the importance of tailored public health strategies to close the gap between actual and optimal performance.

This study used GBD data to assess the overall burden of high BMI and its impact on AF/AFL trends. The nordpred model was applied to project trends through 2040, underscoring the increasing burden of high BMI on cardiovascular health, especially the need for targeted interventions. Decomposition and frontier analyses reveal epidemiologic factors and demographic influences on AF/AFL burdens, guiding targeted interventions. Nonetheless, limitations such as regional disparities, data quality inconsistencies and incomplete data for specific regions may compromise the comprehensiveness and accuracy of our analysis. Future research should focus on refining data collection and employing deeper analyses to better understand AF/AFL burden dynamics related to high BMI.

### Conclusions

In conclusion, our study reveals that from 1990 to 2021, the burden associated with high BMI steadily increased, becoming a significant contributor to the AF/AFL burden. These findings underscore the need for targeted interventions by governments and health policymakers. Tackling the interconnected challenges of AF/AFL and high BMI will aid in developing more effective, region-specific management strategies.

## Supplementary Material

ihaf005_Supplemental_Files

## Data Availability

The datasets presented in this study are available online from https://ghdx.healthdata.org/gbd-results-tool)
