# Supplementary material for: Global trends and projections of high BMI burden and its independent impact on atrial fibrillation and flutter
Source: Int Health. 2025 Feb 4;17(4):552–65. doi: 10.1093/inthealth/ihaf005 (PMC12212217; doi:10.1093/inthealth/ihaf005)
Supplement: ihaf005_Supplemental_Files [file ihaf005_supplemental_files.zip › Supplementary tables.docx]

**Supplementary table 1. Cases and Age-Standardised Rates of High BMI-Related Deaths and Their EAPCs from 1990 to 2021.**

|  | **No.1990** | **ASMR 1990** | **No.2021** | **ASMR 2021** | **EAPC (%)** |
| --- | --- | --- | --- | --- | --- |
| Global | 1459533 (723043-2287052) | 40.9 (19.87-64.16) | 3709063 (1847840-5658334) | 44.23 (22.01-67.64) | 0.17 (0.12-0.21) |
| **Sex** | | | | | |
| Female | 828147 (407103-1302480) | 41.57 (20.18-65.25) | 2013089 (979000-3076045) | 43.26 (21.08-66.05) | 0.01 (-0.04-0.07) |
| Male | 631386 (315453-988214) | 39.03 (19.35-60.8) | 1695974 (861972-2635343) | 44.9 (23.01-70.17) | 0.41 (0.37-0.44) |
| **Age** | | | | | |
| <5 years | 387 (168-663) | 0.06 (0.03-0.11) | 191 (89-313) | 0.03 (0.01-0.05) | -2.39 (-2.48--2.3) |
| 5-9 years | 132 (63-217) | 0.02 (0.01-0.04) | 92 (46-146) | 0.01 (0.01-0.02) | -1.41 (-1.53--1.29) |
| 10-14 years | 121 (59-194) | 0.02 (0.01-0.04) | 135 (68-211) | 0.02 (0.01-0.03) | -0.23 (-0.33--0.14) |
| 15-19 years | 114 (56-183) | 0.02 (0.01-0.04) | 152 (75-240) | 0.02 (0.01-0.04) | 0.16 (0.06-0.26) |
| 20-24 years | 3088 (1950-4484) | 0.63 (0.4-0.91) | 5768 (3106-8558) | 0.97 (0.52-1.43) | 1.34 (1.28-1.4) |
| 25-29 years | 6916 (3904-11683) | 1.56 (0.88-2.64) | 13852 (7317-22601) | 2.35 (1.24-3.84) | 1.34 (1.23-1.45) |
| 30-34 years | 12886 (6813-20917) | 3.34 (1.77-5.43) | 27957 (13959-43452) | 4.63 (2.31-7.19) | 1.09 (1.01-1.17) |
| 35-39 years | 22576 (11339-36724) | 6.41 (3.22-10.43) | 47373 (24506-73616) | 8.45 (4.37-13.13) | 0.82 (0.75-0.88) |
| 40-44 years | 34806 (17753-54434) | 12.15 (6.2-19) | 79318 (41323-119137) | 15.86 (8.26-23.82) | 0.63 (0.53-0.73) |
| 45-49 years | 54812 (27682-84329) | 23.61 (11.92-36.32) | 131652 (68292-197093) | 27.8 (14.42-41.62) | 0.37 (0.28-0.46) |
| 50-54 years | 94188 (46171-146917) | 44.31 (21.72-69.11) | 217629 (104549-331587) | 48.91 (23.5-74.53) | 0.25 (0.17-0.33) |
| 55-59 years | 127040 (63937-196365) | 68.6 (34.52-106.03) | 304563 (153189-456213) | 76.96 (38.71-115.28) | 0.29 (0.21-0.37) |
| 60-64 years | 174446 (87552-272305) | 108.62 (54.51-169.55) | 382571 (188158-574777) | 119.54 (58.79-179.59) | 0.13 (0.04-0.22) |
| 65-69 years | 195324 (96309-306470) | 158.02 (77.91-247.93) | 456166 (223705-693979) | 165.37 (81.1-251.59) | -0.11 (-0.21--0.01) |
| 70-74 years | 187604 (94254-293636) | 221.59 (111.33-346.84) | 476401 (230633-737099) | 231.44 (112.05-358.09) | -0.13 (-0.23--0.03) |
| 75-79 years | 207248 (103308-323917) | 336.68 (167.83-526.22) | 431193 (209866-662456) | 326.95 (159.13-502.3) | -0.04 (-0.11-0.02) |
| 80-84 years | 155406 (71768-247800) | 439.3 (202.87-700.47) | 401328 (187410-632478) | 458.23 (213.98-722.15) | 0.11 (0.05-0.17) |
| 85-89 years | 112105 (48757-180509) | 741.87 (322.65-1194.55) | 370774 (167209-588909) | 810.94 (365.71-1288.03) | 0.34 (0.28-0.4) |
| 90-94 years | 51991 (21567-85720) | 1213.27 (503.3-2000.37) | 247824 (109784-402546) | 1385.31 (613.68-2250.2) | 0.48 (0.4-0.56) |
| 95+ years | 18344 (7087-31049) | 1801.85 (696.08-3049.72) | 114124 (48987-188642) | 2093.9 (898.8-3461.12) | 0.48 (0.42-0.55) |
| SDI | | | | | |
| High-middle SDI | 430095 (194645-685431) | 48.62 (22.28-77.66) | 904396 (417515-1428690) | 46.71 (21.7-73.63) | -0.35 (-0.51--0.2) |
| High SDI | 472370 (199898-767235) | 43.07 (18.21-69.9) | 797416 (368146-1253107) | 35.13 (16.59-54.47) | -0.77 (-0.85--0.69) |
| Low-middle SDI | 177919 (100108-281095) | 31.91 (18.22-49.61) | 671617 (349426-1011204) | 49.59 (26.29-74.99) | 1.58 (1.54-1.62) |
| Low SDI | 70040 (42195-110220) | 32.89 (20.11-51.01) | 204362 (114078-318520) | 43.22 (24.93-66.4) | 0.8 (0.73-0.88) |
| Middle SDI | 306277 (185062-452734) | 33.78 (20.37-50.3) | 1126388 (581131-1700642) | 44.8 (23.6-67.57) | 0.92 (0.89-0.94) |
| Countries | | | | | |
| Afghanistan | 7660 (4192-12503) | 113.19 (63.94-182.55) | 12218 (6512-19874) | 129.38 (72.17-205.41) | 0.3 (0.22-0.37) |
| Albania | 904 (418-1494) | 50.93 (23.6-84.93) | 2049 (852-3436) | 49.26 (20.49-83.36) | 0.15 (-0.06-0.35) |
| Algeria | 6798 (4139-10419) | 72.94 (43.44-111.79) | 26555 (14666-40198) | 96 (52.85-146.29) | 1.08 (0.99-1.17) |
| American Samoa | 30 (14-45) | 135.52 (61.92-204.89) | 83 (41-122) | 181.58 (84.77-270.23) | 0.93 (0.79-1.08) |
| Andorra | 17 (8-28) | 33.5 (15.78-55.55) | 43 (20-76) | 25.13 (11.54-44.35) | -0.64 (-0.85--0.42) |
| Angola | 1681 (1029-2612) | 45.6 (29.51-68.17) | 6864 (3862-10995) | 64.54 (37.18-100.51) | 0.86 (0.74-0.98) |
| Antigua and Barbuda | 41 (23-61) | 75.35 (41.86-112.55) | 95 (59-136) | 95.82 (58.87-138.08) | 0.72 (0.55-0.9) |
| Argentina | 20293 (10045-31379) | 65.81 (32.34-102.06) | 30688 (16263-46234) | 53.52 (28.44-80.54) | -0.46 (-0.56--0.36) |
| Armenia | 1792 (789-2847) | 72.29 (31.62-114.63) | 2769 (1304-4345) | 64.23 (30.16-100.68) | -0.78 (-1.1--0.47) |
| Australia | 7788 (3048-12897) | 41.06 (15.86-68.5) | 15006 (5487-24788) | 29.79 (11.03-48.83) | -1.11 (-1.18--1.04) |
| Austria | 5394 (2310-8750) | 44.6 (19.09-72.5) | 7328 (3510-11701) | 33.13 (16.05-52.7) | -0.73 (-0.81--0.64) |
| Azerbaijan | 3610 (1711-5710) | 77.96 (37.52-125.46) | 7443 (3556-12120) | 81.94 (38.48-135.06) | 0.15 (-0.07-0.38) |
| Bahrain | 202 (96-309) | 146.3 (72.76-230.1) | 951 (486-1403) | 162.45 (80.27-247.15) | -0.06 (-0.36-0.24) |
| Bangladesh | 6069 (3592-10283) | 13.29 (7.69-22.82) | 28352 (13416-47200) | 22 (10.56-36.57) | 1.86 (1.69-2.04) |
| Barbados | 227 (110-344) | 77.92 (38.4-117.11) | 437 (213-683) | 84.45 (41.39-131.85) | 0.28 (0.16-0.4) |
| Belarus | 7233 (2581-12422) | 57.73 (20.48-99.69) | 12559 (4300-21965) | 77.39 (26.59-136.04) | 0.39 (0.01-0.77) |
| Belgium | 4938 (1753-8065) | 31.88 (11.28-51.99) | 6115 (2252-10351) | 21.72 (7.87-36.67) | -1.21 (-1.32--1.09) |
| Belize | 64 (34-96) | 69.22 (36.44-103.46) | 268 (152-378) | 93.22 (52.78-133.18) | 0.94 (0.54-1.35) |
| Benin | 736 (382-1191) | 38.03 (19.82-60.86) | 2530 (1392-4013) | 51.85 (27.46-82.05) | 0.99 (0.91-1.07) |
| Bermuda | 45 (20-71) | 75.82 (34.32-119.52) | 72 (34-114) | 49.4 (23.22-77.77) | -1.36 (-1.6--1.12) |
| Bhutan | 86 (41-153) | 35.19 (17.47-61.59) | 241 (124-406) | 40.79 (21.09-68.44) | 0.46 (0.4-0.51) |
| Bolivia | 1869 (919-3082) | 60.44 (30.26-98.79) | 6245 (3048-9949) | 73.38 (36.49-117.05) | 0.66 (0.61-0.71) |
| Bosnia and Herzegovina | 2016 (916-3261) | 53.79 (24.7-86.5) | 4043 (1759-6424) | 63.5 (27.62-100.79) | 0.43 (0.29-0.56) |
| Botswana | 336 (178-539) | 68.88 (37.44-109.23) | 1165 (608-1733) | 94.76 (50.66-141.35) | 1.4 (1.09-1.71) |
| Brazil | 47939 (24265-72746) | 56.96 (29.03-86.62) | 131576 (63859-199256) | 53.31 (25.84-80.87) | -0.2 (-0.27--0.14) |
| Brunei | 50 (25-78) | 48.9 (24.9-78.24) | 162 (80-247) | 47.46 (24.18-73.13) | 0.18 (0.06-0.3) |
| Bulgaria | 10976 (5416-17338) | 108.2 (52.81-172.21) | 18728 (11698-27024) | 132.52 (82.34-191.15) | 0.72 (0.46-0.97) |
| Burkina Faso | 940 (619-1415) | 22.72 (14.8-34.9) | 2736 (1711-4209) | 30.72 (19.14-46.24) | 1.1 (1.04-1.16) |
| Burundi | 678 (358-1086) | 30.97 (16.32-49.77) | 1382 (773-2201) | 31.45 (17.13-49.72) | -0.51 (-0.73--0.29) |
| Cambodia | 976 (570-1536) | 21.69 (13.07-33.75) | 3293 (1854-5106) | 27.24 (16.17-42.49) | 0.66 (0.52-0.8) |
| Cameroon | 2885 (1587-4479) | 71.19 (39.22-109.93) | 11601 (6102-18289) | 103.68 (53.87-167.24) | 1.12 (0.88-1.35) |
| Canada | 13143 (4934-21947) | 40.95 (15.25-68.54) | 22123 (8756-35949) | 28.24 (11.37-45.23) | -1.39 (-1.52--1.26) |
| Cape Verde | 61 (35-93) | 26.74 (15.5-40.8) | 248 (130-404) | 57.27 (29.92-93.58) | 2.18 (1.96-2.41) |
| Central African Republic | 628 (344-1077) | 56.08 (31.43-90.44) | 1902 (994-3387) | 86.75 (47.61-149.29) | 1.38 (1.33-1.43) |
| Chad | 872 (473-1432) | 32.78 (17.7-54.53) | 2518 (1381-4042) | 46.69 (25.8-76.58) | 1.04 (0.84-1.25) |
| Chile | 5005 (2381-7866) | 53.61 (25.7-84.61) | 11211 (5859-17140) | 42.85 (22.44-65.47) | -0.48 (-0.62--0.33) |
| China | 142048 (92773-216077) | 21.01 (13.05-32.02) | 575624 (291291-923748) | 30.01 (15.41-47.75) | 1.25 (1.13-1.36) |
| Colombia | 7857 (4455-11366) | 48.21 (27.99-69.6) | 21017 (9781-33536) | 37.28 (17.47-59.38) | -1.21 (-1.35--1.06) |
| Comoros | 77 (44-116) | 43.44 (24.64-64.73) | 270 (152-435) | 60.88 (33.56-98.49) | 0.88 (0.72-1.05) |
| Congo | 770 (472-1169) | 74.86 (45.74-112.72) | 2514 (1422-3880) | 100.81 (59.2-157.45) | 0.67 (0.53-0.81) |
| Cook Islands | 25 (15-34) | 204.67 (126.37-284.82) | 40 (22-56) | 157.76 (87.88-219.9) | -0.9 (-0.97--0.84) |
| Costa Rica | 657 (335-1018) | 38.84 (19.87-60.38) | 2566 (1310-3931) | 45.85 (23.47-70.24) | 0.11 (-0.08-0.31) |
| Cote d'Ivoire | 1661 (885-2721) | 45.65 (24.7-74.59) | 6899 (3761-10951) | 67.47 (37-105) | 1.23 (1.03-1.42) |
| Croatia | 4312 (2112-6873) | 79.42 (39.32-127.58) | 5703 (2630-9149) | 59.28 (27.19-95.05) | -0.88 (-0.97--0.79) |
| Cuba | 3674 (1527-5681) | 36.86 (15.3-57.14) | 8863 (4535-13791) | 43.91 (22.47-68.18) | 0.62 (0.38-0.85) |
| Cyprus | 443 (218-682) | 76.77 (37.55-116.56) | 833 (405-1357) | 48.79 (24.02-79.09) | -1.71 (-1.89--1.54) |
| Czech Republic | 12000 (4693-20020) | 88 (34.05-147.44) | 12595 (5401-20574) | 54.86 (23.76-89.2) | -1.39 (-1.52--1.26) |
| Democratic Republic of the Congo | 7266 (4056-11803) | 51.21 (29.68-81.46) | 27838 (14505-43790) | 87.35 (47.09-137.17) | 1.68 (1.65-1.72) |
| Denmark | 3057 (1153-4964) | 36.85 (14.05-59.76) | 3220 (1227-5381) | 24.34 (9.25-40.62) | -1.51 (-1.59--1.44) |
| Djibouti | 30 (19-46) | 25.47 (16.49-39.7) | 199 (110-321) | 35.67 (19.91-57.93) | 0.95 (0.9-1.01) |
| Dominica | 69 (40-98) | 118.17 (70.43-169.47) | 110 (63-159) | 137.82 (79.46-197.1) | 0.45 (0.36-0.53) |
| Dominican Republic | 1356 (735-2080) | 38.05 (20.84-58.76) | 6069 (3301-9527) | 60.7 (32.93-95.71) | 2.05 (1.88-2.22) |
| Ecuador | 2535 (1383-3897) | 50.12 (27.8-76.59) | 10200 (5590-15259) | 65.85 (36.3-99.39) | 1.21 (0.86-1.56) |
| Egypt | 35278 (20800-50048) | 164.95 (97.12-237.6) | 111096 (62741-164171) | 220.64 (126.39-323.13) | 1.37 (1.22-1.52) |
| El Salvador | 1432 (693-2220) | 48.36 (23.29-74.86) | 5184 (2599-7997) | 81.2 (41.04-124.57) | 1.68 (1.55-1.81) |
| Equatorial Guinea | 155 (91-263) | 82.81 (49.14-138.52) | 494 (251-799) | 110.71 (58.09-178.51) | 0.8 (0.45-1.14) |
| Eritrea | 299 (184-455) | 28.77 (18.18-42) | 995 (533-1657) | 40.23 (22.51-65.34) | 1.11 (1.06-1.17) |
| Estonia | 1563 (634-2578) | 79.14 (31.81-131.09) | 2484 (1471-3561) | 79.03 (49.08-110.9) | 0.16 (-0.03-0.35) |
| Ethiopia | 8273 (5101-13011) | 43.16 (26.84-65.75) | 11459 (6867-17964) | 28.16 (16.68-43.51) | -1.94 (-2.15--1.72) |
| Federated States of Micronesia | 79 (39-125) | 160.72 (81.71-254.36) | 151 (69-233) | 203.66 (91.8-315.59) | 0.76 (0.67-0.85) |
| Fiji | 725 (351-1128) | 206.63 (96.27-329.21) | 2053 (1021-3046) | 291.64 (137.36-433.79) | 0.91 (0.72-1.11) |
| Finland | 3170 (1265-5327) | 44.49 (17.54-74.84) | 4790 (2216-7713) | 31.58 (14.28-50.93) | -1.02 (-1.14--0.91) |
| France | 21792 (9733-34668) | 25.28 (11.23-40.31) | 39013 (15722-65353) | 21.57 (8.53-35.85) | -0.47 (-0.64--0.31) |
| Gabon | 523 (308-794) | 98.26 (58.47-146.77) | 1273 (721-1963) | 139.89 (79.54-214.94) | 1.01 (0.85-1.18) |
| Georgia | 4351 (1767-7085) | 72.81 (29.4-120.01) | 4513 (2526-6805) | 72.51 (40.2-109.05) | 0.35 (-0.01-0.71) |
| Germany | 74824 (33691-119286) | 57.33 (25.61-91.45) | 84764 (38990-138767) | 36.35 (17.02-59.2) | -1.35 (-1.44--1.26) |
| Ghana | 2344 (1332-3763) | 39.18 (21.94-62.97) | 11393 (5904-18625) | 76.38 (39.42-125.32) | 2.2 (2.1-2.3) |
| Greece | 5678 (2447-9129) | 39.09 (16.86-63.22) | 11748 (5432-18801) | 40.05 (18.87-64.09) | -0.07 (-0.14-0.01) |
| Greenland | 22 (9-36) | 74.27 (29.92-125.16) | 27 (11-44) | 43.43 (17.21-72.98) | -1.74 (-1.85--1.63) |
| Grenada | 57 (29-86) | 79.29 (40.45-120.1) | 113 (62-166) | 105.11 (56.96-155.05) | 1.03 (0.92-1.15) |
| Guam | 64 (36-93) | 95.09 (51.67-141) | 136 (62-213) | 64.09 (29.79-100.12) | -0.85 (-1.18--0.52) |
| Guatemala | 1534 (746-2420) | 50.32 (24.72-79.66) | 8329 (4318-12664) | 79.02 (40.75-120.69) | 1.38 (1.16-1.6) |
| Guinea | 1183 (649-1868) | 37.03 (19.99-58.44) | 2903 (1618-4538) | 54.02 (30.16-86.12) | 1.34 (1.24-1.44) |
| Guinea-Bissau | 221 (120-367) | 57.1 (31.88-93.56) | 562 (316-905) | 80.9 (45.41-127.41) | 1.15 (1.05-1.25) |
| Guyana | 437 (255-636) | 117.79 (70.11-170.39) | 837 (463-1249) | 134.96 (75.71-204.98) | 0.74 (0.61-0.86) |
| Haiti | 1577 (853-2452) | 48.71 (26.77-76.29) | 4691 (2288-7424) | 65.39 (32.33-104.25) | 1.16 (1.08-1.25) |
| Honduras | 840 (447-1281) | 43.66 (24.15-66.67) | 4581 (2406-6979) | 79.53 (41.79-120.1) | 2.1 (1.91-2.28) |
| Hungary | 13002 (6319-20473) | 92.8 (43.86-146.32) | 15125 (7892-22947) | 73.16 (38.89-111.46) | -0.66 (-0.77--0.55) |
| Iceland | 110 (40-187) | 37.26 (13.64-63.08) | 164 (57-283) | 24.85 (8.74-42.66) | -1.26 (-1.33--1.2) |
| India | 68298 (32800-115456) | 15.12 (7.8-25.44) | 343478 (160551-537363) | 30.11 (14.34-47.25) | 2.44 (2.33-2.55) |
| Indonesia | 18299 (11538-28369) | 18.44 (11.48-28.92) | 87652 (48930-132450) | 37.18 (20.42-56.27) | 2.45 (2.3-2.6) |
| Iran | 12320 (7209-17725) | 55.88 (32.38-81.67) | 49884 (26921-73223) | 71.65 (39.21-106.75) | 0.87 (0.76-0.97) |
| Iraq | 9298 (4549-14575) | 120.89 (59.29-190.04) | 27381 (12734-42989) | 136.68 (66.68-213.63) | -0.07 (-0.23-0.09) |
| Ireland | 1785 (693-3026) | 45.1 (17.35-76.68) | 2001 (701-3378) | 23.92 (8.4-40.33) | -1.98 (-2.09--1.87) |
| Israel | 2374 (997-3721) | 51.69 (21.59-81.34) | 4294 (1705-7003) | 31.28 (12.51-50.78) | -1.88 (-2.09--1.68) |
| Italy | 33725 (15760-53040) | 38.1 (17.96-59.88) | 60219 (27790-95796) | 31.64 (15.01-50.36) | -0.58 (-0.62--0.54) |
| Jamaica | 1259 (769-1810) | 69.84 (42.53-100.2) | 2796 (1479-4302) | 86.62 (45.65-132.94) | 0.74 (0.53-0.95) |
| Japan | 22428 (12154-34835) | 14.19 (7.67-22) | 46108 (22357-77180) | 9.71 (4.84-16.08) | -1.2 (-1.45--0.95) |
| Jordan | 1452 (791-2147) | 126.75 (70.3-189.92) | 6141 (3466-9276) | 103.94 (59.1-157.34) | -1.05 (-1.36--0.73) |
| Kazakhstan | 7896 (3130-13349) | 67.45 (25.9-115.4) | 10413 (4187-17247) | 68.8 (26.75-114.44) | -0.94 (-1.56--0.32) |
| Kenya | 1912 (1168-2956) | 25.29 (15.39-38.99) | 10382 (5659-16697) | 50.95 (27.18-80.63) | 2.62 (2.48-2.76) |
| Kiribati | 65 (28-107) | 163.84 (70.3-269.52) | 164 (73-260) | 223.5 (97.92-355.6) | 0.98 (0.88-1.07) |
| Kuwait | 472 (278-688) | 86.15 (51.54-129.48) | 1969 (983-2994) | 76.31 (37.06-116.65) | -0.41 (-0.76--0.06) |
| Kyrgyzstan | 1657 (625-2745) | 58.79 (21.88-97.39) | 3045 (1366-4783) | 71.01 (31.54-111.84) | 0.33 (0.01-0.65) |
| Laos | 722 (394-1172) | 35.68 (19.84-57.04) | 1950 (1035-3216) | 43.09 (23.01-71.29) | 0.58 (0.55-0.61) |
| Latvia | 2640 (916-4647) | 75.59 (26.14-133.74) | 3016 (1452-4891) | 69.54 (33.32-111.65) | -0.5 (-0.73--0.26) |
| Lebanon | 1794 (879-2844) | 91.48 (45.01-144.38) | 4509 (2205-6895) | 70.1 (34.36-106.33) | -0.81 (-1.01--0.62) |
| Lesotho | 535 (312-880) | 69.36 (39.44-114.62) | 1607 (849-2738) | 163.2 (85.18-272.08) | 3.77 (3.22-4.33) |
| Liberia | 666 (369-1058) | 59.88 (33.19-94.47) | 1816 (1014-2878) | 88.45 (50.11-139.61) | 1.24 (1.13-1.35) |
| Libya | 1107 (571-1696) | 61.9 (31.77-95.09) | 5849 (3072-9175) | 122.68 (63.8-192) | 2.74 (2.56-2.91) |
| Lithuania | 2794 (1027-4812) | 63.39 (23.27-109.02) | 3996 (1694-6618) | 63.25 (27.15-104.65) | -0.11 (-0.31-0.1) |
| Luxembourg | 225 (89-361) | 42.54 (16.78-68.66) | 316 (136-521) | 26.42 (11.52-43.63) | -1.4 (-1.49--1.31) |
| Macedonia | 1491 (729-2341) | 86.6 (42.74-135.42) | 2798 (1410-4418) | 105.41 (54.51-166.63) | 0.53 (0.16-0.89) |
| Madagascar | 1696 (1159-2533) | 36.58 (24.16-55.04) | 5588 (3104-8756) | 57.81 (33.26-90.06) | 1.37 (1.29-1.45) |
| Malawi | 1093 (678-1697) | 31.76 (19.55-49.96) | 3549 (2026-5706) | 52.19 (29.98-84.19) | 1.35 (1.14-1.55) |
| Malaysia | 3436 (1592-5420) | 36.96 (17.3-58.29) | 12532 (5630-19259) | 46.04 (20.27-71.87) | 0.5 (0.35-0.65) |
| Maldives | 33 (17-53) | 33.67 (17.63-53.97) | 80 (42-128) | 22.95 (12.16-36.84) | -1.63 (-1.84--1.41) |
| Mali | 1552 (842-2542) | 41.17 (22.49-66.61) | 3794 (1996-6086) | 45.38 (23.33-74.19) | 0.32 (0.24-0.4) |
| Malta | 173 (70-273) | 42.48 (17.4-67.18) | 340 (143-560) | 31.48 (13.31-51.72) | -0.94 (-1.08--0.79) |
| Marshall Islands | 30 (14-49) | 181.91 (84.68-294.39) | 86 (38-132) | 244.71 (109.19-376.77) | 0.93 (0.76-1.09) |
| Mauritania | 747 (405-1184) | 81.34 (44.55-130.09) | 1755 (933-2711) | 92.12 (48.97-141.63) | 0.16 (0.05-0.27) |
| Mauritius | 515 (287-753) | 72.02 (40.7-105.22) | 1936 (1053-2823) | 106.45 (57.77-155.69) | 1.87 (1.52-2.22) |
| Mexico | 30253 (15313-45454) | 76.61 (39.12-116.1) | 116838 (61628-170739) | 95.06 (49.57-140.15) | 0.72 (0.64-0.8) |
| Moldova | 2883 (1076-4789) | 75.55 (27.81-125.51) | 5151 (2529-7795) | 85.33 (42.04-129.03) | -0.07 (-0.3-0.16) |
| Monaco | 27 (10-47) | 35.52 (13.29-62.25) | 39 (15-66) | 33.46 (13.04-56.33) | -0.15 (-0.21--0.08) |
| Mongolia | 638 (265-1103) | 63.96 (26.53-110.01) | 1242 (528-2095) | 58.89 (25.55-100.06) | -0.61 (-0.86--0.36) |
| Montenegro | 357 (150-597) | 59.48 (24.83-99.98) | 810 (346-1330) | 90.63 (38.97-149.55) | 1.43 (1.28-1.58) |
| Morocco | 9792 (5548-14965) | 73.25 (41.58-110.85) | 32247 (17613-49778) | 103 (57.35-159.06) | 1.25 (1.17-1.33) |
| Mozambique | 1748 (1133-2549) | 32.01 (21.05-45.56) | 6193 (3449-10086) | 58.96 (34.09-96.68) | 2.48 (2.3-2.67) |
| Myanmar | 9669 (5277-15829) | 40.6 (22.98-65.99) | 19822 (10375-31410) | 40.81 (21.78-65.24) | -0.3 (-0.42--0.19) |
| Namibia | 423 (238-673) | 74.72 (40.86-119.38) | 1405 (716-2189) | 119.26 (59.94-187.14) | 1.31 (0.95-1.66) |
| Nauru | 12 (5-18) | 226.52 (103.17-348.29) | 18 (8-27) | 296.35 (135.41-454.58) | 0.75 (0.49-1.02) |
| Nepal | 1550 (748-2771) | 15.89 (7.91-28.41) | 6089 (3071-10364) | 26.42 (13.77-44.13) | 1.91 (1.62-2.21) |
| Netherlands | 7264 (2594-12330) | 36.04 (12.9-61.26) | 9393 (3511-15835) | 24.09 (9.09-40.44) | -1.46 (-1.58--1.35) |
| New Zealand | 1855 (762-3021) | 48.24 (19.67-78.48) | 2869 (1092-4724) | 32.25 (12.5-52.72) | -1.47 (-1.54--1.39) |
| Nicaragua | 680 (360-1044) | 46.5 (24.35-72.3) | 2991 (1696-4536) | 64.59 (36.15-98.2) | 1.27 (1.06-1.48) |
| Niger | 787 (423-1330) | 30.24 (16.55-49.9) | 2566 (1405-4064) | 33.45 (18.1-51.63) | 0.24 (0.11-0.36) |
| Nigeria | 16613 (9668-25877) | 42.38 (24.89-66.45) | 46948 (24323-72264) | 60.91 (31.14-94.76) | 0.98 (0.88-1.09) |
| Niue | 3 (1-4) | 126.64 (59.95-198.27) | 4 (2-6) | 182.98 (84.74-278.76) | 1.05 (0.95-1.15) |
| North Korea | 2495 (1346-4434) | 19.51 (10.49-34.01) | 10380 (5336-17808) | 35.73 (18.03-62.96) | 2.06 (2.01-2.11) |
| Northern Mariana Islands | 19 (8-32) | 105.26 (43.97-172.28) | 61 (28-89) | 125.4 (52.84-191.49) | 0.65 (0.57-0.74) |
| Norway | 2462 (890-4116) | 34.5 (12.68-57.56) | 2160 (795-3729) | 18.67 (6.88-31.92) | -2.17 (-2.25--2.1) |
| Oman | 516 (268-818) | 81.95 (43.56-127.51) | 1903 (1089-2717) | 119.43 (67.76-173.34) | 1.79 (1.57-2) |
| Pakistan | 15951 (8246-26425) | 29.48 (15.58-47.91) | 70519 (35388-113568) | 60.45 (31.19-96.39) | 2.33 (2.08-2.58) |
| Palau | 13 (5-21) | 137.45 (55.4-223.52) | 35 (16-56) | 170.85 (73.16-272.33) | 0.82 (0.73-0.92) |
| Palestine | 1021 (570-1592) | 134.9 (74.02-212.57) | 2681 (1432-3858) | 135.02 (72.12-196.05) | -0.03 (-0.23-0.18) |
| Panama | 621 (268-1009) | 44.29 (18.91-72.24) | 2620 (1229-4084) | 57.96 (27.27-90.09) | 0.87 (0.77-0.96) |
| Papua New Guinea | 1226 (564-2138) | 63.56 (29.94-111.04) | 3992 (1799-6536) | 72.02 (32.12-120.12) | 0.38 (0.34-0.43) |
| Paraguay | 1020 (509-1606) | 47.83 (23.63-75.44) | 4500 (2273-7100) | 80.1 (40.1-126.66) | 1.98 (1.85-2.11) |
| Peru | 4072 (2060-6834) | 34.68 (17.65-58.09) | 13225 (6648-21122) | 39.3 (19.74-62.67) | 0.13 (-0.14-0.41) |
| Philippines | 9286 (5485-13959) | 32.33 (19.52-48.5) | 44982 (24143-69453) | 55.41 (30.73-84.74) | 1.9 (1.79-2.02) |
| Poland | 31429 (13632-51352) | 75.05 (32.22-121.93) | 39608 (17992-62704) | 52.01 (23.79-81.8) | -1.38 (-1.51--1.25) |
| Portugal | 5542 (2264-9231) | 42.56 (17.55-70.67) | 9253 (3884-15182) | 31.03 (13.22-51.07) | -1.31 (-1.47--1.15) |
| Puerto Rico | 2758 (1377-4195) | 79.57 (39.63-121.78) | 5171 (2548-7773) | 66.75 (33.59-98.22) | -0.72 (-0.82--0.63) |
| Qatar | 120 (58-187) | 149.73 (73.34-238.75) | 722 (359-1104) | 124.44 (59.3-193.58) | -0.98 (-1.64--0.32) |
| Romania | 19854 (10546-30382) | 79.84 (42.35-123.06) | 28153 (15373-43268) | 71.42 (38.98-109.92) | -0.6 (-0.73--0.46) |
| Russian Federation | 108257 (38408-182550) | 65.14 (22.73-110.14) | 177027 (64241-290589) | 73.71 (26.89-120.86) | -0.13 (-0.57-0.3) |
| Rwanda | 1172 (684-1794) | 44.35 (27.15-65.86) | 2114 (1186-3465) | 38.5 (20.9-62.3) | -1.44 (-1.84--1.05) |
| Saint Kitts and Nevis | 36 (18-56) | 99.2 (50.46-153.59) | 62 (34-92) | 100.26 (56.62-148.43) | 0.47 (0.26-0.67) |
| Saint Lucia | 72 (40-106) | 89.52 (50.72-132.05) | 178 (98-272) | 75.81 (41.92-115.6) | -1.04 (-1.32--0.76) |
| Saint Vincent and the Grenadines | 54 (29-79) | 78.38 (42.84-115.81) | 125 (77-182) | 93.03 (56.46-134.51) | 0.37 (0.12-0.61) |
| Samoa | 114 (55-170) | 135.43 (65.75-204.85) | 242 (121-365) | 171.39 (82.91-261.09) | 0.75 (0.7-0.81) |
| San Marino | 11 (5-18) | 29.25 (12.9-47.82) | 17 (6-31) | 17.23 (6.64-30.78) | -0.97 (-1.27--0.67) |
| Sao Tome and Principe | 25 (14-39) | 40.93 (22.47-64.49) | 67 (36-105) | 67.68 (35.46-107.03) | 1.56 (1.47-1.65) |
| Saudi Arabia | 5827 (3075-9207) | 109.32 (58.99-171.61) | 27560 (15451-40443) | 158.89 (90.15-230.8) | 1.06 (0.91-1.21) |
| Senegal | 1456 (819-2341) | 47.54 (26.36-76.9) | 4642 (2544-7260) | 64.96 (35.59-102.13) | 0.97 (0.9-1.04) |
| Serbia | 8158 (4022-12857) | 89.28 (44.72-140.97) | 14519 (7301-22536) | 84.51 (42.53-131.25) | -0.67 (-0.88--0.45) |
| Seychelles | 38 (27-51) | 67.68 (47.82-90.5) | 91 (58-129) | 82.99 (51.75-118.27) | 0.78 (0.64-0.92) |
| Sierra Leone | 647 (358-1048) | 33.7 (18.5-54.38) | 1802 (971-2913) | 50.64 (26.97-82.84) | 1.47 (1.3-1.64) |
| Singapore | 474 (253-723) | 22.12 (12.38-34.04) | 1237 (612-1936) | 14.58 (7.16-22.8) | -1.33 (-1.5--1.17) |
| Slovakia | 5271 (2258-8575) | 90.34 (38.78-147.35) | 6240 (2786-10036) | 65.18 (29.14-104.84) | -1.01 (-1.08--0.95) |
| Slovenia | 1328 (631-2090) | 54.38 (25.63-85.79) | 2061 (1025-3216) | 39.23 (19.33-61.29) | -1.31 (-1.42--1.19) |
| Solomon Islands | 128 (56-220) | 89.56 (39.71-154.58) | 461 (203-740) | 124.6 (54.56-204.81) | 1.09 (1-1.19) |
| Somalia | 1005 (619-1659) | 41.69 (26.37-66.01) | 3155 (1773-5383) | 53.07 (29.92-87.95) | 0.79 (0.76-0.82) |
| South Africa | 14486 (7769-21859) | 72.62 (40.27-107.84) | 52667 (29889-77210) | 125.66 (71.79-182.68) | 1.88 (1.42-2.34) |
| South Korea | 6115 (3471-9860) | 25.63 (14.15-41.55) | 14469 (6561-23522) | 15.9 (7.27-26.01) | -1.89 (-2.07--1.71) |
| South Sudan | 607 (374-983) | 25.46 (15.43-40.55) | 1259 (719-2080) | 34.43 (19.5-57.87) | 0.83 (0.55-1.11) |
| Spain | 21610 (8642-35856) | 40.41 (16.23-67.14) | 36460 (14806-59641) | 29.3 (12-47.59) | -1.1 (-1.18--1.03) |
| Sri Lanka | 3049 (1764-4563) | 30.57 (17.58-46.35) | 10410 (4614-18254) | 40.16 (17.74-70.9) | 1.18 (1-1.36) |
| Sudan | 7681 (4374-11903) | 88.44 (50.78-135.6) | 19472 (10190-30822) | 108.55 (57.35-169.22) | 0.58 (0.48-0.68) |
| Suriname | 131 (73-197) | 51.66 (29.07-78.59) | 404 (202-631) | 64.06 (31.98-99.94) | 0.9 (0.7-1.1) |
| Swaziland | 302 (174-469) | 125.39 (69.78-196.83) | 1011 (518-1610) | 208.19 (104.71-325.25) | 2.09 (1.49-2.7) |
| Sweden | 5538 (1966-9387) | 34.76 (12.47-58.19) | 6409 (2810-10625) | 24.31 (10.82-40.28) | -0.99 (-1.05--0.94) |
| Switzerland | 3760 (1659-6109) | 34.22 (15.07-55.4) | 4809 (2132-7871) | 20.53 (9.11-33.7) | -1.56 (-1.6--1.51) |
| Syria | 5179 (2702-7971) | 107.54 (56.55-165.71) | 16853 (8543-27110) | 154.17 (78.12-241.68) | 0.79 (0.59-0.99) |
| Taiwan | 4932 (2512-7613) | 35.88 (19.1-57.04) | 14505 (6898-22837) | 33.15 (15.98-51.95) | -0.49 (-0.64--0.33) |
| Tajikistan | 2028 (1008-3146) | 79.03 (40.12-122.75) | 3551 (1741-5630) | 72.63 (35.45-113.84) | -0.59 (-0.94--0.23) |
| Tanzania | 4039 (2410-6143) | 41.89 (24.92-63.73) | 14367 (7773-23152) | 63.34 (34.92-101.84) | 1.27 (1.23-1.32) |
| Thailand | 6546 (3151-11018) | 18.78 (9.04-31.68) | 32153 (14997-53405) | 29.76 (13.91-49.52) | 1.01 (0.78-1.23) |
| The Bahamas | 137 (84-195) | 90.21 (55.69-129.56) | 402 (254-576) | 103.46 (65.12-150.22) | 0.47 (0.37-0.57) |
| The Gambia | 150 (81-245) | 45.13 (24.56-73.97) | 705 (389-1163) | 75.82 (41.51-124.42) | 1.53 (1.38-1.69) |
| Timor-Leste | 33 (20-51) | 12.26 (7.13-19.28) | 157 (92-248) | 18.92 (11.02-29.93) | 1.62 (1.31-1.93) |
| Togo | 448 (267-702) | 39.45 (23.41-61.84) | 2184 (1164-3441) | 65.38 (35.98-103.74) | 1.6 (1.48-1.73) |
| Tokelau | 2 (1-3) | 120.89 (58.59-196.43) | 2 (1-3) | 141.65 (64.73-221.65) | 0.45 (0.36-0.53) |
| Tonga | 61 (27-92) | 110.45 (47.7-167) | 117 (53-177) | 147.25 (65.37-224.03) | 0.95 (0.82-1.08) |
| Trinidad and Tobago | 1018 (520-1431) | 128.88 (66.77-181.27) | 2142 (1101-3262) | 111.35 (57.39-170.05) | -0.79 (-0.97--0.61) |
| Tunisia | 2278 (1387-3366) | 52.3 (31.34-78.36) | 9579 (4897-14885) | 78.91 (40.43-122.98) | 1.27 (1.22-1.32) |
| Turkey | 27730 (14499-41464) | 90.99 (48.75-136.74) | 72672 (37263-109493) | 84.43 (43.14-127.26) | -0.11 (-0.47-0.24) |
| Turkmenistan | 1351 (595-2195) | 75.63 (33.82-123.42) | 3405 (1495-5686) | 89.35 (39.66-148.9) | -0.17 (-0.49-0.15) |
| Tuvalu | 10 (5-17) | 148.71 (69.99-246.51) | 18 (8-28) | 175.66 (78.52-275.86) | 0.62 (0.59-0.65) |
| Uganda | 1613 (924-2649) | 26.52 (15.45-42.58) | 5556 (2968-8737) | 40.11 (22.68-63.8) | 0.82 (0.59-1.05) |
| Ukraine | 44354 (16355-74995) | 65.81 (23.61-112.13) | 65564 (24683-112459) | 83.71 (31.49-143.58) | 0.22 (-0.21-0.65) |
| United Arab Emirates | 384 (187-604) | 96.23 (47.41-149.9) | 2805 (1493-4163) | 127.69 (67.33-190.53) | 2.59 (2.02-3.16) |
| United Kingdom | 43231 (16204-73195) | 47.27 (17.69-79.85) | 39876 (13708-68393) | 27.78 (9.77-47.34) | -1.84 (-2--1.68) |
| United States | 169380 (70829-275487) | 52.72 (22.15-85.38) | 334863 (160446-513083) | 55.46 (26.91-83.74) | -0.06 (-0.19-0.06) |
| Uruguay | 2011 (863-3226) | 52 (22.15-83.68) | 2920 (1389-4614) | 48.02 (22.72-75.43) | -0.32 (-0.39--0.24) |
| Uzbekistan | 7219 (3015-11652) | 66.01 (27.84-106.6) | 21334 (10308-33534) | 90.9 (42.76-144.73) | 0.94 (0.62-1.26) |
| Vanuatu | 58 (26-100) | 91.1 (41.86-158.93) | 223 (101-347) | 125.47 (56.67-198.23) | 0.94 (0.88-1) |
| Venezuela | 6678 (3648-9997) | 72.76 (40.39-109.14) | 26607 (13817-41360) | 91.04 (47.28-141.99) | 0.43 (0.19-0.68) |
| Vietnam | 4277 (2650-6525) | 11.31 (6.97-17.37) | 18976 (9963-30183) | 20.44 (10.86-32.53) | 2.34 (2.12-2.56) |
| Virgin Islands, U.S. | 71 (39-108) | 96.27 (51.42-147.83) | 112 (55-170) | 65.81 (32.65-99.14) | -1.08 (-1.23--0.93) |
| Yemen | 2642 (1583-3968) | 59.8 (36.83-87.85) | 11050 (5959-17388) | 89.11 (47.68-141.28) | 1.14 (0.94-1.33) |
| Zambia | 1223 (799-1870) | 45.58 (29.18-69.03) | 4634 (2604-7220) | 73.24 (41.83-114.2) | 1.12 (0.95-1.29) |
| Zimbabwe | 1467 (856-2298) | 40.15 (23.16-63.15) | 6808 (3602-11164) | 106.99 (57.48-175.13) | 3.98 (3.34-4.61) |
| GBD Regions | | | | | |
| Advanced Health System | 793730 (334227-1283914) | 49.48 (20.6-80.18) | 1325726 (616585-2082736) | 43.15 (20.13-67.42) | -0.65 (-0.75--0.54) |
| Africa | 151517 (90193-228330) | 60.15 (35.72-89.11) | 491144 (275282-731417) | 86.48 (49.45-129.8) | 1.24 (1.16-1.31) |
| African Region | 94344 (57111-145418) | 47.03 (28.05-72.34) | 309546 (173097-462646) | 69.78 (39.98-105.27) | 1.22 (1.1-1.34) |
| America | 328029 (148669-517873) | 54.97 (24.69-87.03) | 785877 (391430-1189782) | 57.7 (28.87-87.18) | 0.03 (-0.04-0.1) |
| Andean Latin America | 8477 (4359-13446) | 42.53 (22.06-67.27) | 29671 (15386-45445) | 51.2 (26.62-78.55) | 0.6 (0.51-0.69) |
| Asia | 411063 (250995-626618) | 22.95 (13.78-34.92) | 1582563 (790020-2451590) | 32.74 (16.55-50.42) | 1.18 (1.12-1.24) |
| Australasia | 9644 (3814-15917) | 42.24 (16.49-70.14) | 17874 (6579-29574) | 30.2 (11.26-49.62) | -1.17 (-1.23--1.1) |
| Basic Health System | 456897 (276645-681229) | 35.52 (21.27-53.13) | 1588620 (831670-2431932) | 45.22 (24.04-69.32) | 0.82 (0.79-0.85) |
| Caribbean | 13542 (6637-20073) | 54.26 (26.89-80.53) | 34101 (18371-51663) | 62.7 (33.77-94.84) | 0.55 (0.45-0.65) |
| Central Africa | 13801 (7915-22435) | 53.32 (31.3-84.55) | 49589 (26511-76540) | 84.44 (46.49-128.72) | 1.39 (1.31-1.47) |
| Central Asia | 30541 (13023-49523) | 69.28 (29.46-112.3) | 57716 (27365-91196) | 78.1 (36.56-124.36) | 0.06 (-0.27-0.39) |
| Central Europe | 112904 (53681-180476) | 80.37 (38.14-128.14) | 154684 (80237-240151) | 66.48 (34.23-103.25) | -0.71 (-0.77--0.66) |
| Central Latin America | 50551 (26167-75477) | 64.8 (33.95-97.34) | 190732 (100714-282328) | 77.4 (40.57-115.19) | 0.5 (0.41-0.6) |
| Central Sub-Saharan Africa | 11023 (6409-17551) | 53.54 (32.96-83.07) | 40885 (22008-63609) | 84.78 (46.8-132.07) | 1.38 (1.34-1.43) |
| Commonwealth High Income | 68655 (26451-114863) | 45.41 (17.42-75.9) | 85674 (31590-142392) | 29.12 (10.96-48.14) | -1.56 (-1.66--1.45) |
| Commonwealth Low Income | 18294 (11390-28660) | 22.05 (13.78-34.02) | 72315 (38945-115552) | 33.52 (18.35-53.45) | 1.36 (1.25-1.47) |
| Commonwealth Middle Income | 134838 (71130-216597) | 23.36 (12.79-37.66) | 583101 (285571-893854) | 39.47 (19.68-60.82) | 1.77 (1.7-1.84) |
| East Asia | 149476 (97212-228250) | 21.26 (13.11-32.6) | 600509 (305671-959271) | 30.15 (15.52-47.93) | 1.21 (1.1-1.32) |
| East Asia & Pacific | 135351 (83686-205070) | 12.2 (7.47-18.76) | 910959 (463446-1420491) | 28.57 (14.57-44.66) | 1.36 (1.03-1.68) |
| Eastern Africa | 28958 (18093-43786) | 44.94 (28.48-66.15) | 76843 (44135-117423) | 53.35 (30.88-81.42) | 0.38 (0.32-0.44) |
| Eastern Europe | 169725 (61326-285006) | 65.37 (23.35-110.39) | 269797 (101119-441648) | 76.12 (28.67-124.57) | -0.03 (-0.43-0.38) |
| Eastern Mediterranean Region | 122010 (70640-182720) | 74.66 (43.42-111.12) | 418743 (230310-620876) | 104.48 (58.45-156.2) | 1.17 (1.13-1.2) |
| Eastern Sub-Saharan Africa | 25484 (16356-38847) | 36.58 (23.09-54.58) | 71164 (42572-111348) | 46.25 (27.66-71.23) | 0.59 (0.52-0.65) |
| Europe | 563105 (236244-916609) | 55.41 (22.97-90.13) | 838961 (381469-1347737) | 48.45 (21.92-77.79) | -0.63 (-0.77--0.5) |
| Europe & Central Asia | 340500 (136190-556757) | 32.89 (13.11-53.83) | 877572 (400685-1409499) | 49.59 (22.46-79.35) | -0.48 (-1.25-0.3) |
| European Region | 585606 (246025-953175) | 55.78 (23.15-90.69) | 884977 (404230-1421819) | 49.52 (22.42-79.23) | -0.6 (-0.74--0.45) |
| High-income Asia Pacific | 29067 (15781-45301) | 15.74 (8.69-24.7) | 61976 (30276-102313) | 10.83 (5.23-17.74) | -1.25 (-1.42--1.09) |
| High-income North America | 182550 (75592-298330) | 51.59 (21.45-83.89) | 357019 (169558-550600) | 52.47 (25.25-79.65) | -0.17 (-0.3--0.04) |
| Latin America & Caribbean | 148335 (75458-225453) | 59.02 (30.17-90.18) | 434174 (223073-657964) | 62.23 (31.83-94.43) | 0.17 (0.14-0.2) |
| Limited Health System | 179245 (99069-289914) | 24.16 (13.61-38.62) | 715483 (361494-1097899) | 38.28 (19.75-58.99) | 1.52 (1.48-1.56) |
| Middle East & North Africa | 99056 (57625-144156) | 92.26 (53.42-134.44) | 344569 (194626-505426) | 113.77 (64.49-168.9) | 0.74 (0.7-0.77) |
| Minimal Health System | 26828 (16067-43002) | 47.27 (28.59-74.65) | 74352 (41740-117669) | 64.15 (37.31-100.47) | 0.95 (0.91-0.99) |
| North Africa and Middle East | 139628 (80646-204715) | 94.42 (54.85-138.13) | 444512 (248383-651458) | 111.09 (62.99-165.05) | 0.6 (0.5-0.7) |
| North America | 182569 (75601-298358) | 51.59 (21.45-83.89) | 357059 (169582-550653) | 52.47 (25.25-79.66) | -0.17 (-0.3--0.04) |
| Northern Africa | 56000 (33592-80928) | 105.52 (63.68-154.08) | 187081 (106618-276161) | 140.23 (81.34-210.83) | 1.19 (1.1-1.28) |
| Oceania | 2844 (1359-4573) | 96.78 (46.56-156.9) | 8257 (3869-12846) | 109.44 (49.51-172.77) | 0.36 (0.3-0.42) |
| Region of the Americas | 328029 (148669-517873) | 54.97 (24.69-87.03) | 785877 (391430-1189782) | 57.7 (28.87-87.18) | 0.03 (-0.04-0.1) |
| South-East Asia Region | 116127 (61666-191416) | 16.91 (9.4-27.99) | 538813 (262583-848117) | 30.93 (15.4-48.55) | 2.09 (2.04-2.14) |
| South Asia | 102696 (52481-170962) | 17.99 (9.54-29.63) | 471385 (224979-730855) | 32.47 (15.85-50.86) | 2.05 (1.99-2.12) |
| South Asia region | 91954 (46062-155163) | 16.58 (8.74-27.79) | 448678 (214705-698248) | 31.7 (15.44-49.7) | 2.25 (2.18-2.32) |
| Southeast Asia | 56960 (34861-88608) | 22.47 (13.77-34.68) | 234361 (124222-359833) | 36.59 (19.55-56.24) | 1.6 (1.51-1.68) |
| Southern Africa | 23294 (13464-35408) | 58.19 (34.19-86.57) | 85904 (48075-129276) | 103.14 (59.7-153.49) | 1.97 (1.6-2.35) |
| Southern Latin America | 27311 (13290-42425) | 61.94 (30.01-96.55) | 44822 (23531-68037) | 49.94 (26.29-75.77) | -0.5 (-0.6--0.4) |
| Southern Sub-Saharan Africa | 17548 (9676-26624) | 68.63 (39.12-103.45) | 64664 (36007-95739) | 125.12 (71.21-183.13) | 2.14 (1.68-2.6) |
| Sub-Saharan Africa | 96233 (57630-148224) | 47.7 (28.16-73.04) | 305618 (170710-455619) | 69.25 (39.62-104.28) | 0.7 (0.06-1.33) |
| Tropical Latin America | 48960 (24788-74359) | 56.75 (28.86-86.26) | 136076 (66289-205885) | 53.9 (26.22-81.66) | -0.15 (-0.21--0.09) |
| Western Africa | 29465 (17255-45945) | 40.89 (23.59-64.36) | 91728 (49812-140265) | 59.33 (32.37-90.78) | 1.1 (1.02-1.17) |
| Western Europe | 247354 (102754-400466) | 42.09 (17.47-68.29) | 333896 (144773-546996) | 29.36 (12.96-48.02) | -1.15 (-1.2--1.11) |
| Western Pacific Region | 202640 (127350-307395) | 20.67 (12.75-31.45) | 746055 (378238-1164910) | 27.11 (13.78-42.32) | 0.94 (0.81-1.07) |
| Western Sub-Saharan Africa | 33994 (19733-52942) | 42.57 (24.58-67.37) | 107670 (58441-164491) | 62.14 (33.48-94.63) | 1.12 (1.03-1.21) |
| World Bank High Income | 587156 (251800-948711) | 45.81 (19.63-74.06) | 965411 (452992-1513821) | 37.11 (17.9-57.42) | -0.78 (-0.84--0.71) |
| World Bank Low Income | 60424 (36711-94447) | 45.06 (28.19-68.52) | 167182 (94842-256398) | 56.58 (32.74-87.03) | 0.57 (0.48-0.66) |
| World Bank Lower Middle Income | 308848 (163034-484352) | 33.06 (17.96-51.2) | 1126236 (569948-1714885) | 48.44 (24.9-73.71) | 1.24 (1.19-1.29) |
| World Bank Upper Middle Income | 500244 (266782-772103) | 38.95 (20.7-60.22) | 1445305 (719922-2226640) | 43.4 (21.86-66.71) | 0.21 (0.12-0.29) |

No.: Number, ASMR: Age-standardized Mortality Rate, EAPC: Estimated Annual Percentage Changes, SDI: Social Demographic Index.

**Supplementary table 2. Cases and Age-Standardised Rates of High BMI-Related DALYs and Their EAPCs from 1990 to 2021.**

|  | **No.1990** | **ASDR 1990** | **No.2021** | **ASDR 2021** | **EAPC (%)** |
| --- | --- | --- | --- | --- | --- |
| **Global** | 48042109 (21178404-77317173) | 1189.69 (522.74-1915.93) | 128520083 (55989249-202388462) | 1493.24 (648.2-2350.72) | 0.66 (0.62-0.69) |
| **Sex** | | | | | |
| **Female** | 26097463 (11042501-42206794) | 1219.65 (517.22-1973.78) | 67213786 (28417735-105552569) | 1483.07 (629.86-2327.33) | 0.54 (0.5-0.58) |
| **Male** | 21944646 (10106039-35110379) | 1141.04 (531.56-1819.1) | 61306297 (27566755-94931875) | 1497.29 (673.38-2322.15) | 0.82 (0.79-0.85) |
| **Age** | | | | | |
| **<5 years** | 98979 (44501-175940) | 15.97 (7.18-28.38) | 101247 (45174-184020) | 15.38 (6.86-27.96) | -0.15 (-0.28--0.01) |
| **5-9 years** | 87575 (39750-164367) | 15.01 (6.81-28.17) | 129963 (59221-252726) | 18.92 (8.62-36.78) | 0.98 (0.79-1.17) |
| **10-14 years** | 64921 (29043-121594) | 12.12 (5.42-22.7) | 113529 (49795-216442) | 17.03 (7.47-32.47) | 1.3 (1.14-1.46) |
| **15-19 years** | 41352 (19035-74746) | 7.96 (3.66-14.39) | 70677 (32192-128125) | 11.33 (5.16-20.53) | 1.15 (1.1-1.21) |
| **20-24 years** | 468440 (226088-756001) | 95.19 (45.94-153.63) | 1037161 (449025-1642431) | 173.68 (75.19-275.04) | 1.99 (1.95-2.03) |
| **25-29 years** | 864948 (399359-1470450) | 195.42 (90.23-332.22) | 2000882 (891401-3262516) | 340.09 (151.51-554.53) | 1.9 (1.82-1.97) |
| **30-34 years** | 1344722 (594619-2235107) | 348.9 (154.28-579.91) | 3381096 (1535905-5292987) | 559.34 (254.09-875.63) | 1.61 (1.55-1.67) |
| **35-39 years** | 2013626 (874723-3364243) | 571.66 (248.33-955.09) | 4938617 (2282506-7765650) | 880.53 (406.96-1384.58) | 1.37 (1.33-1.41) |
| **40-44 years** | 2634862 (1162463-4281491) | 919.73 (405.77-1494.51) | 6861164 (3132085-10529201) | 1371.54 (626.1-2104.78) | 1.16 (1.09-1.23) |
| **45-49 years** | 3454120 (1576464-5351216) | 1487.59 (678.94-2304.62) | 9636851 (4398403-14615719) | 2035.22 (928.9-3086.71) | 0.9 (0.84-0.96) |
| **50-54 years** | 4983391 (2173088-7946821) | 2344.34 (1022.29-3738.42) | 13302264 (5905706-20265912) | 2989.79 (1327.35-4554.93) | 0.74 (0.69-0.79) |
| **55-59 years** | 5763027 (2561456-9150346) | 3111.79 (1383.08-4940.79) | 15753247 (7070054-24148933) | 3980.83 (1786.59-6102.41) | 0.75 (0.69-0.81) |
| **60-64 years** | 6648148 (2994993-10612200) | 4139.33 (1864.77-6607.47) | 16500494 (7198509-25414976) | 5155.63 (2249.2-7940.99) | 0.6 (0.53-0.66) |
| **65-69 years** | 6201854 (2733811-10012558) | 5017.3 (2211.65-8100.16) | 16371028 (7213459-25586741) | 5934.93 (2615.07-9275.86) | 0.36 (0.29-0.44) |
| **70-74 years** | 4806087 (2198033-7696788) | 5676.85 (2596.27-9091.29) | 13759772 (5967150-21713194) | 6684.72 (2898.94-10548.62) | 0.3 (0.22-0.38) |
| **75-79 years** | 4141391 (1870901-6701102) | 6727.9 (3039.37-10886.27) | 9603424 (4118040-15301458) | 7281.7 (3122.46-11602.17) | 0.3 (0.24-0.36) |
| **80-84 years** | 2399638 (1028481-3913400) | 6783.25 (2907.29-11062.32) | 6684223 (2906138-10765517) | 7631.87 (3318.15-12291.78) | 0.35 (0.3-0.41) |
| **85-89 years** | 1336031 (556413-2176591) | 8841.4 (3682.15-14403.95) | 4644233 (1965708-7516299) | 10157.59 (4299.28-16439.2) | 0.49 (0.44-0.54) |
| **90-94 years** | 520635 (211351-873326) | 12149.64 (4932.13-20380.11) | 2565887 (1066575-4223937) | 14343.08 (5962.06-23611.43) | 0.58 (0.51-0.64) |
| **95+ years** | 168362 (63015-293694) | 16537.08 (6189.58-28847.68) | 1064326 (433969-1777643) | 19527.82 (7962.28-32615.45) | 0.51 (0.45-0.57) |
| **SDI** | | | | | |
| **High-middle SDI** | 13456764 (5557920-21736296) | 1361.36 (558.23-2199.25) | 28327365 (11609614-45257815) | 1482.16 (608.58-2355.15) | 0.02 (-0.1-0.15) |
| **High SDI** | 14426131 (5751872-23595458) | 1349.13 (539.85-2203.07) | 26714020 (10995809-41789440) | 1457.17 (627.77-2256.99) | 0.2 (0.13-0.27) |
| **Low-middle SDI** | 6286643 (3073215-10180681) | 938.73 (471.39-1504.69) | 24642855 (11581774-38153750) | 1598.37 (751.34-2480.38) | 1.85 (1.81-1.89) |
| **Low SDI** | 2468140 (1381991-4049774) | 969.81 (540.86-1583.95) | 7889939 (3922665-12376646) | 1346.99 (674.2-2106.27) | 0.96 (0.9-1.03) |
| **Middle SDI** | 11315469 (5727436-17561040) | 1014.16 (526.82-1571.85) | 40789161 (18222014-62362498) | 1488.92 (668.95-2281.19) | 1.22 (1.2-1.23) |
| **Countries** | | | | | |
| Afghanistan | 250030 (128134-408734) | 3422.96 (1796.24-5623.75) | 517339 (264083-831145) | 4154.03 (2152.85-6564.04) | 0.49 (0.42-0.56) |
| Albania | 29326 (11412-49132) | 1404.99 (550.33-2366.14) | 59628 (23010-98152) | 1443.61 (555.35-2379.33) | 0.24 (0.11-0.37) |
| Algeria | 227112 (120503-355995) | 1841.81 (1002.54-2845.39) | 932808 (471820-1392838) | 2598.05 (1346.53-3915.52) | 1.13 (1.05-1.21) |
| American Samoa | 1223 (573-1762) | 4477.73 (2032.4-6546.64) | 3245 (1601-4508) | 6341.73 (3062.07-8924.56) | 1.1 (0.98-1.22) |
| Andorra | 584 (236-972) | 1022.07 (423.72-1712.87) | 1446 (550-2435) | 976.56 (372.58-1633.65) | -0.02 (-0.17-0.12) |
| Angola | 61438 (37306-96985) | 1326.49 (819.8-2096.29) | 274187 (144308-435025) | 1941.81 (1042.9-3095.56) | 1.03 (0.94-1.12) |
| Antigua and Barbuda | 1182 (604-1736) | 2300 (1156.45-3369.37) | 3198 (1750-4513) | 2945.64 (1637.52-4180.14) | 0.76 (0.63-0.89) |
| Argentina | 613554 (264507-959944) | 1908.75 (819.85-2991.88) | 990283 (416992-1510016) | 1815.42 (768.78-2767.58) | -0.08 (-0.13--0.03) |
| Armenia | 56617 (23707-90114) | 2039.6 (861.4-3250.9) | 83427 (36578-130469) | 1970.86 (863.94-3075.25) | -0.42 (-0.65--0.2) |
| Australia | 249089 (90186-421121) | 1305.58 (473.1-2209.47) | 503322 (170348-824119) | 1240.57 (429.82-2022.79) | -0.22 (-0.25--0.18) |
| Austria | 138316 (53267-225339) | 1222.31 (469.89-1988.13) | 175554 (69960-279763) | 1002.24 (381.67-1596.58) | -0.57 (-0.61--0.52) |
| Azerbaijan | 117646 (52110-191629) | 2257.11 (1001.25-3692.31) | 259047 (113782-415481) | 2412.3 (1048.59-3885.09) | 0.03 (-0.17-0.23) |
| Bahrain | 7523 (3409-11525) | 3703.44 (1667.69-5691.51) | 42723 (20778-62769) | 4247.61 (1993.88-6234.86) | 0.01 (-0.18-0.2) |
| Bangladesh | 233148 (119492-413111) | 428.66 (226.43-737.84) | 1262457 (539222-2089627) | 852.77 (364.21-1402.74) | 2.66 (2.53-2.8) |
| Barbados | 6360 (2887-9580) | 2357.67 (1079.88-3514.35) | 13455 (6300-20123) | 2766.9 (1314.68-4103.44) | 0.49 (0.41-0.56) |
| Belarus | 214434 (75110-362695) | 1679.45 (587.6-2837.15) | 345000 (118948-593206) | 2223.64 (771.8-3821.47) | 0.37 (0.01-0.73) |
| Belgium | 145215 (50068-241237) | 1003.04 (350.1-1650.16) | 197598 (65508-329825) | 954.64 (330.55-1554.19) | -0.2 (-0.24--0.16) |
| Belize | 2186 (1038-3284) | 2201.58 (1031.94-3315.64) | 10229 (5339-14121) | 3101.64 (1588.12-4314.26) | 1.06 (0.75-1.36) |
| Benin | 26789 (13129-43956) | 1228.35 (607.43-2039) | 106861 (53032-167607) | 1747.3 (850.21-2785.16) | 1.07 (1.01-1.14) |
| Bermuda | 1348 (574-2106) | 2137.02 (909.51-3344.9) | 2189 (930-3351) | 1783.79 (781.94-2696.65) | -0.55 (-0.75--0.35) |
| Bhutan | 3460 (1534-6338) | 1174.81 (534.8-2090.79) | 9143 (4321-15247) | 1405.91 (668.71-2333.3) | 0.54 (0.51-0.58) |
| Bolivia | 66133 (31092-108390) | 1837.73 (863.9-2999.29) | 215812 (103013-330429) | 2251.81 (1064.95-3459.47) | 0.62 (0.57-0.68) |
| Bosnia and Herzegovina | 72337 (29830-119517) | 1697.04 (698.33-2809.26) | 122842 (50122-195424) | 2059.35 (843.92-3277.14) | 0.55 (0.45-0.64) |
| Botswana | 10572 (5436-17493) | 1800.05 (914.25-2898.27) | 39147 (18507-60521) | 2531.03 (1210.09-3847.53) | 1.37 (1.11-1.63) |
| Brazil | 1802982 (817524-2784356) | 1843.67 (843.63-2845.43) | 4753305 (2023465-7241422) | 1876.75 (797.85-2864.91) | -0.01 (-0.05-0.03) |
| Brunei | 2085 (902-3230) | 1596.78 (702.43-2470.14) | 9054 (3910-13807) | 2091.37 (913.43-3232.59) | 0.96 (0.91-1.02) |
| Bulgaria | 318680 (141019-504926) | 2746.03 (1239.04-4380.06) | 444178 (264900-656782) | 3385.67 (2016.31-5003.72) | 0.64 (0.43-0.84) |
| Burkina Faso | 34043 (21359-52104) | 699.6 (438-1069.04) | 108465 (63106-168372) | 991.78 (581.23-1551.79) | 1.2 (1.12-1.28) |
| Burundi | 22107 (11950-35753) | 859.73 (456.24-1395.06) | 51305 (26393-81748) | 894.09 (471.02-1419.37) | -0.42 (-0.63--0.2) |
| Cambodia | 35118 (19324-57401) | 679.02 (379.6-1105.81) | 126991 (65237-201463) | 921.84 (483.59-1450.48) | 0.92 (0.79-1.04) |
| Cameroon | 97473 (49224-152951) | 1994.23 (1015.02-3129.73) | 423264 (217599-661632) | 2929.8 (1490.38-4599.55) | 1.14 (0.9-1.38) |
| Canada | 391804 (140721-659638) | 1229.96 (444.66-2064.63) | 774859 (297476-1252690) | 1195.86 (470.78-1916.89) | -0.25 (-0.32--0.18) |
| Cape Verde | 2028 (1055-3191) | 924.11 (477.47-1450.97) | 8569 (4182-13374) | 1801.12 (880.93-2827.6) | 2.01 (1.9-2.12) |
| Central African Republic | 22737 (11928-39664) | 1663.11 (881.16-2863.09) | 76714 (37505-136598) | 2700.85 (1387.35-4767.74) | 1.54 (1.49-1.59) |
| Chad | 28398 (14629-47006) | 964.22 (491.95-1606.77) | 94925 (49532-152244) | 1423.77 (739.24-2265.06) | 1.16 (0.97-1.35) |
| Chile | 172239 (71730-277612) | 1664.05 (694.65-2668.9) | 414804 (174866-637214) | 1663.69 (704.17-2556.53) | 0.13 (0.06-0.2) |
| China | 5433920 (3170338-8612167) | 624.53 (366.98-975.46) | 20865172 (8495258-33636820) | 1028.85 (428.39-1635.04) | 1.71 (1.63-1.79) |
| Colombia | 291620 (140496-441872) | 1518.64 (737.72-2288.04) | 821305 (345254-1312872) | 1483.66 (626.88-2367.64) | -0.39 (-0.52--0.26) |
| Comoros | 2639 (1444-4063) | 1191.17 (672.61-1827.05) | 9391 (5043-15095) | 1772.08 (938.61-2819.65) | 1.08 (0.92-1.24) |
| Congo | 25799 (15375-40621) | 2159.39 (1307.56-3356.8) | 93637 (48412-147771) | 2921.45 (1539.99-4595.2) | 0.68 (0.52-0.83) |
| Cook Islands | 831 (496-1143) | 6124.02 (3621.03-8477.85) | 1335 (723-1841) | 5457.16 (2996.04-7503.33) | -0.39 (-0.44--0.33) |
| Costa Rica | 26532 (11806-40894) | 1395.58 (629.35-2164.16) | 101262 (45715-153474) | 1840.09 (832.4-2793.23) | 0.63 (0.51-0.75) |
| Cote d'Ivoire | 65702 (32678-107761) | 1348.49 (689.87-2216.61) | 279352 (139597-441368) | 2059.44 (1035.52-3261.37) | 1.32 (1.14-1.49) |
| Croatia | 123211 (53418-200391) | 2076.93 (903.17-3362.63) | 149982 (60604-239042) | 1803.69 (733.18-2852.09) | -0.46 (-0.52--0.41) |
| Cuba | 134231 (55625-211223) | 1302.26 (539.39-2050.37) | 302161 (138346-459235) | 1659.93 (765.52-2512.4) | 0.77 (0.63-0.92) |
| Cyprus | 11822 (5042-18706) | 1633.46 (742.94-2544.33) | 25822 (10214-41801) | 1367.9 (557.13-2200.64) | -0.78 (-0.87--0.69) |
| Czech Republic | 338385 (127846-560394) | 2508.76 (948.48-4158.01) | 359381 (144329-585388) | 1782.23 (715.79-2914.02) | -0.99 (-1.12--0.87) |
| Democratic Republic of the Congo | 241514 (128846-399969) | 1396.67 (753.12-2289.32) | 980920 (475217-1536469) | 2387.31 (1189.81-3757.8) | 1.69 (1.65-1.72) |
| Denmark | 84223 (29595-139118) | 1122.84 (397.06-1857.1) | 96030 (32366-160365) | 914.76 (315.87-1532.58) | -0.81 (-0.88--0.75) |
| Djibouti | 1153 (711-1823) | 686.34 (438.66-1061.68) | 7670 (4002-12370) | 1006.49 (546.17-1608.41) | 1.11 (1.05-1.17) |
| Dominica | 1852 (974-2644) | 3182.56 (1677.02-4529.32) | 3345 (1782-4732) | 4015.2 (2129.46-5674.4) | 0.72 (0.64-0.8) |
| Dominican Republic | 55351 (27148-83813) | 1312.55 (633.45-1988.09) | 244831 (120651-369686) | 2345.78 (1157.24-3548.2) | 2.27 (2.15-2.38) |
| Ecuador | 91552 (42806-143482) | 1562.72 (759.2-2444.84) | 359071 (176454-535255) | 2166.6 (1063.68-3235.74) | 1.21 (0.96-1.45) |
| Egypt | 1097976 (588472-1588614) | 3882.32 (2153.62-5610.15) | 3677442 (1899748-5372000) | 5575.85 (2913.12-8210.47) | 1.47 (1.36-1.58) |
| El Salvador | 54691 (24221-85889) | 1693.36 (753.63-2654.85) | 170881 (81774-257498) | 2776.25 (1340.74-4170.31) | 1.6 (1.5-1.7) |
| Equatorial Guinea | 5304 (2857-9039) | 2407.62 (1337.78-4097.99) | 18853 (9257-29758) | 3084.52 (1545.59-4872.9) | 0.64 (0.33-0.96) |
| Eritrea | 11032 (6807-16872) | 799.99 (490.92-1221.07) | 37938 (19209-63476) | 1149 (599.49-1897.16) | 1.19 (1.13-1.26) |
| Estonia | 43851 (17528-71751) | 2195.8 (880.93-3586.32) | 53612 (31859-76890) | 2070.55 (1186.39-2985.8) | -0.24 (-0.39--0.09) |
| Ethiopia | 291157 (169780-467619) | 1256.36 (749.69-1994.42) | 425788 (235773-667144) | 850.92 (472.45-1341.3) | -1.81 (-2.02--1.6) |
| Federated States of Micronesia | 2887 (1353-4585) | 5256.21 (2488.6-8326.85) | 5880 (2780-8882) | 6780.9 (3180.91-10248.73) | 0.84 (0.77-0.92) |
| Fiji | 25911 (12645-39304) | 6065.2 (2866.5-9349.69) | 69010 (34735-99971) | 8343.95 (4069.55-12130.29) | 0.9 (0.76-1.04) |
| Finland | 91955 (34355-152850) | 1342.9 (503.26-2226.83) | 129659 (49298-210994) | 1169.2 (458.55-1865.32) | -0.41 (-0.49--0.33) |
| France | 609959 (221995-1021723) | 776.15 (281.56-1305.24) | 1062403 (363890-1770740) | 846.25 (299.68-1395.32) | 0.3 (0.21-0.39) |
| Gabon | 15810 (8945-24927) | 2680.67 (1519.84-4176.84) | 42942 (22475-67116) | 3792.66 (1999.01-5893.52) | 0.99 (0.83-1.16) |
| Georgia | 132542 (52067-212345) | 2132.35 (840.1-3434.71) | 125494 (63996-192905) | 2228.94 (1111.48-3422.48) | 0.17 (-0.1-0.45) |
| Germany | 1907266 (760037-3111949) | 1566.64 (624.67-2553.49) | 2097373 (798050-3448317) | 1186.17 (456.2-1944.61) | -0.85 (-0.97--0.74) |
| Ghana | 86614 (46433-137063) | 1183.23 (639.28-1891.44) | 415234 (207042-672176) | 2196.08 (1087.67-3551.71) | 2.05 (1.94-2.15) |
| Greece | 167582 (65024-276554) | 1161.42 (452.47-1901.22) | 293840 (114323-468091) | 1394.85 (550.53-2197.33) | 0.49 (0.45-0.53) |
| Greenland | 842 (329-1399) | 2147.03 (849.42-3570.05) | 1075 (422-1739) | 1524.58 (594.37-2484.45) | -1.15 (-1.26--1.05) |
| Grenada | 1719 (839-2612) | 2597.97 (1261.75-3907.44) | 4012 (2039-5836) | 3409.13 (1708.64-4990.71) | 0.98 (0.88-1.07) |
| Guam | 2416 (1218-3543) | 2749.77 (1404.54-4076.18) | 5453 (2480-8191) | 2719.87 (1251.79-4063.39) | 0.21 (0-0.42) |
| Guatemala | 66771 (28431-109093) | 1643.5 (687.37-2689.73) | 334318 (159919-489813) | 2829.13 (1335.81-4180.95) | 1.7 (1.52-1.89) |
| Guinea | 39257 (20173-64055) | 1114.68 (567.51-1809.95) | 105473 (53726-166069) | 1649.92 (835.25-2601.2) | 1.34 (1.27-1.41) |
| Guinea-Bissau | 7896 (4140-13600) | 1728.16 (923.84-2915.94) | 22180 (11882-35353) | 2453.71 (1297.61-3917.53) | 1.15 (1.05-1.24) |
| Guyana | 15387 (8143-22706) | 3640.04 (1948.31-5358.15) | 31647 (17100-46104) | 4545.66 (2423.61-6604.19) | 0.94 (0.83-1.04) |
| Haiti | 61371 (32785-95141) | 1617.2 (867.99-2534.75) | 205706 (97247-314646) | 2343.9 (1103.1-3667.25) | 1.4 (1.32-1.47) |
| Honduras | 34596 (16629-53134) | 1505.79 (719.02-2322.13) | 173069 (80984-261175) | 2554.88 (1213.75-3881.06) | 1.81 (1.7-1.93) |
| Hungary | 381382 (165773-604035) | 2702.08 (1170.03-4276.59) | 413860 (190827-636760) | 2296.92 (1064.2-3555.5) | -0.54 (-0.62--0.46) |
| Iceland | 3413 (1189-5745) | 1231.34 (430.35-2075.1) | 5630 (1885-9385) | 1064.43 (373.08-1752.43) | -0.5 (-0.54--0.47) |
| India | 2739730 (1177445-4598928) | 506.43 (224.06-844.83) | 13146272 (5627607-20467709) | 1030.55 (442.96-1609.87) | 2.46 (2.38-2.53) |
| Indonesia | 735428 (419367-1176593) | 622.99 (362.01-987.26) | 3497935 (1675589-5429045) | 1265.88 (626.32-1960.43) | 2.4 (2.26-2.55) |
| Iran | 441125 (215530-668565) | 1570.77 (801.53-2356.32) | 1768239 (836856-2605319) | 2188.4 (1020.5-3231.03) | 1.11 (1.04-1.19) |
| Iraq | 307072 (139456-474924) | 3572.63 (1633.18-5525.57) | 1052106 (507608-1594232) | 4034.58 (1907.51-6234.12) | 0.09 (-0.01-0.18) |
| Ireland | 53514 (20108-89379) | 1370.71 (515.37-2285.19) | 72469 (24561-119518) | 995.28 (346.44-1630.76) | -1.08 (-1.15--1.01) |
| Israel | 68387 (26453-109106) | 1448.65 (556.85-2313.1) | 132750 (48726-215790) | 1116.65 (418.06-1806.67) | -1.05 (-1.17--0.93) |
| Italy | 929598 (368029-1492926) | 1086.58 (430.4-1745.02) | 1382615 (558749-2280821) | 998.72 (390.53-1638.43) | -0.33 (-0.36--0.29) |
| Jamaica | 36464 (19052-53299) | 2073.58 (1078.17-3033.39) | 86442 (44358-127965) | 2776.83 (1429.55-4106.67) | 0.91 (0.74-1.09) |
| Japan | 920724 (391777-1511391) | 558.42 (242.51-913.7) | 1641865 (627744-2810199) | 588.23 (219.54-995.7) | 0.11 (-0.01-0.22) |
| Jordan | 50270 (25081-73852) | 3458.5 (1752.45-5120.44) | 261255 (133748-382880) | 3226.95 (1660.01-4789.56) | -0.55 (-0.79--0.32) |
| Kazakhstan | 259320 (102416-432549) | 1999.82 (774.09-3345.14) | 378791 (152329-614079) | 2099.88 (829.37-3408.27) | -0.78 (-1.33--0.22) |
| Kenya | 68363 (37795-108458) | 731.53 (403.26-1156.68) | 375483 (176638-610942) | 1434.22 (683.37-2326.95) | 2.46 (2.31-2.6) |
| Kiribati | 2531 (1103-4137) | 5584.03 (2425.02-9187.29) | 6544 (2946-9981) | 7441.1 (3329.09-11466.83) | 0.9 (0.81-0.99) |
| Kuwait | 20965 (10469-30964) | 2615.24 (1341.59-3862.84) | 111497 (53966-168037) | 2904.64 (1330.6-4406.01) | 0.39 (0.15-0.63) |
| Kyrgyzstan | 55793 (20706-92572) | 1828.81 (675.48-3051.51) | 109697 (47326-173245) | 2143.85 (917.45-3362.1) | 0.13 (-0.17-0.43) |
| Laos | 24663 (13061-39952) | 1064.38 (576.82-1703.8) | 75443 (35808-121587) | 1415.22 (675.77-2344.9) | 0.93 (0.89-0.98) |
| Latvia | 74394 (25243-126638) | 2128.11 (725.15-3626.28) | 75917 (31977-121453) | 2076.41 (863.67-3291.75) | -0.44 (-0.67--0.21) |
| Lebanon | 58311 (26895-93368) | 2602.46 (1201.33-4168.67) | 147060 (65708-217284) | 2436.9 (1099.6-3584.1) | -0.11 (-0.31-0.09) |
| Lesotho | 15521 (8049-25631) | 1807.84 (973.77-2978.69) | 50038 (24926-86687) | 4380.6 (2209.21-7421.47) | 3.8 (3.29-4.32) |
| Liberia | 22816 (11546-37464) | 1826.76 (924.03-2962.51) | 72509 (37893-110886) | 2724.92 (1421.59-4185.26) | 1.27 (1.15-1.38) |
| Libya | 38376 (18529-59171) | 1853.56 (890.7-2849.78) | 220986 (114271-331076) | 3754.45 (1917.57-5638.14) | 2.65 (2.52-2.78) |
| Lithuania | 78063 (27672-133773) | 1761.94 (625.03-3019.59) | 100282 (39464-165231) | 1878.06 (752.76-3076.26) | 0.07 (-0.13-0.27) |
| Luxembourg | 6494 (2397-10703) | 1244.46 (463.13-2046.47) | 10226 (3825-16619) | 1010.38 (385.86-1642.68) | -0.68 (-0.73--0.63) |
| Macedonia | 46011 (19529-74181) | 2435.62 (1043.39-3912.38) | 84306 (38101-134034) | 2701.77 (1236.89-4275.38) | 0.23 (0.01-0.45) |
| Madagascar | 55015 (36217-82577) | 976.32 (633.07-1475.56) | 200303 (111934-314095) | 1537.36 (851.17-2404.9) | 1.37 (1.29-1.45) |
| Malawi | 38974 (23444-60998) | 874.49 (515.42-1377.51) | 127119 (66326-206155) | 1457.71 (774.21-2341.54) | 1.36 (1.16-1.57) |
| Malaysia | 133566 (55642-213898) | 1274.68 (532.55-2049.65) | 503645 (215591-793536) | 1671.06 (711.99-2634.15) | 0.73 (0.62-0.83) |
| Maldives | 1322 (620-2137) | 1163.07 (560.22-1874.64) | 4215 (1905-6571) | 969.93 (444.68-1537.42) | -0.88 (-1.08--0.67) |
| Mali | 56210 (28907-94478) | 1261.7 (645.5-2121.53) | 163445 (80976-261484) | 1562.64 (762.53-2532.66) | 0.72 (0.64-0.79) |
| Malta | 5016 (1877-8091) | 1188.26 (442.27-1917.15) | 10428 (3842-17006) | 1223.14 (472.79-1972.29) | 0.08 (0.01-0.14) |
| Marshall Islands | 1170 (530-1899) | 5886.01 (2673.99-9492.58) | 3623 (1701-5384) | 8289.49 (3804.65-12450.29) | 1.08 (0.92-1.24) |
| Mauritania | 22886 (11735-36507) | 2186.66 (1108.08-3481.93) | 54610 (26987-84842) | 2404.84 (1189.9-3724.05) | 0.09 (-0.01-0.19) |
| Mauritius | 17690 (9005-26381) | 2223.37 (1136.55-3295.19) | 62994 (32086-91205) | 3424.86 (1750.55-4969.3) | 1.91 (1.64-2.18) |
| Mexico | 1164137 (548415-1741227) | 2480.63 (1166.79-3728.22) | 4203963 (2076475-6119103) | 3197.23 (1567.84-4660.14) | 0.76 (0.69-0.83) |
| Moldova | 88969 (32467-146098) | 2050.66 (748.94-3368.36) | 148045 (68271-228624) | 2539.96 (1172.4-3922.59) | 0.34 (0.12-0.56) |
| Monaco | 696 (228-1189) | 1131.29 (369.39-1928.28) | 1016 (347-1673) | 1208.4 (444.82-1983.09) | 0.22 (0.2-0.25) |
| Mongolia | 21631 (8396-37441) | 1931.29 (758.9-3329.31) | 50465 (19969-84274) | 1904.41 (751.41-3163.07) | -0.35 (-0.56--0.13) |
| Montenegro | 11951 (4643-19934) | 1887.94 (728.3-3152.62) | 23530 (9456-37659) | 2526.19 (1010.31-4049.25) | 0.96 (0.85-1.07) |
| Morocco | 318773 (159864-497485) | 2112.11 (1092.04-3261.9) | 1144972 (586212-1748780) | 3217.98 (1672.11-4924.62) | 1.47 (1.41-1.53) |
| Mozambique | 60108 (37747-91386) | 887.81 (561.69-1335.74) | 232144 (118755-383361) | 1732.42 (914.01-2833.15) | 2.64 (2.46-2.82) |
| Myanmar | 351572 (186733-581433) | 1322.34 (706.7-2192.31) | 765995 (379196-1203765) | 1439.05 (713.55-2269.06) | -0.04 (-0.15-0.08) |
| Namibia | 13306 (6916-21855) | 1961.77 (1025.28-3222.89) | 43767 (20490-69800) | 3052.37 (1429.29-4777.68) | 1.2 (0.84-1.55) |
| Nauru | 465 (211-711) | 7668.12 (3478.8-11756.34) | 719 (334-1075) | 9922.73 (4581.83-14859.93) | 0.71 (0.45-0.98) |
| Nepal | 67091 (29291-122567) | 586.7 (263.69-1067.07) | 276214 (125923-452625) | 1071.98 (499.85-1759.22) | 2.18 (1.92-2.44) |
| Netherlands | 209268 (71816-347679) | 1086.2 (377.7-1801.53) | 287332 (95970-479123) | 912.11 (317.6-1517.09) | -0.7 (-0.78--0.61) |
| New Zealand | 62768 (24466-100619) | 1661.67 (653.68-2659.87) | 102061 (37286-166227) | 1337.11 (502.42-2151.74) | -0.77 (-0.9--0.64) |
| Nicaragua | 29245 (13618-45608) | 1655.3 (785.31-2556.33) | 125687 (63567-187081) | 2390.41 (1200.35-3567.65) | 1.29 (1.16-1.42) |
| Niger | 30920 (15301-52409) | 933.3 (449.1-1576.56) | 106525 (53686-173366) | 1105.75 (561.34-1789.94) | 0.45 (0.35-0.56) |
| Nigeria | 527440 (288646-833333) | 1140.68 (636.81-1820.14) | 1677762 (781327-2621266) | 1676.99 (773.32-2637.17) | 1.07 (0.97-1.17) |
| Niue | 86 (39-135) | 4119.26 (1866.96-6470.63) | 128 (61-186) | 6013.13 (2858.58-8800.69) | 1.06 (0.97-1.15) |
| North Korea | 86048 (45877-154181) | 535.26 (280.7-948.62) | 308837 (153264-526232) | 973.92 (476.16-1663.23) | 1.96 (1.92-1.99) |
| Northern Mariana Islands | 891 (369-1414) | 3361.15 (1370.03-5365.99) | 2325 (1046-3381) | 4057.97 (1760.38-6010.85) | 0.69 (0.64-0.73) |
| Norway | 70764 (25223-119605) | 1145.21 (415.17-1924.77) | 72196 (24265-119596) | 802.72 (281.85-1326.26) | -1.32 (-1.39--1.25) |
| Oman | 18511 (8831-29142) | 2324.67 (1145.66-3613.29) | 82127 (41095-118115) | 3323.28 (1732.01-4768.85) | 1.52 (1.39-1.65) |
| Pakistan | 568693 (278164-975167) | 926.26 (458.54-1570.55) | 2858934 (1314368-4549385) | 1990.59 (929.15-3166.87) | 2.56 (2.33-2.8) |
| Palau | 487 (203-769) | 4431.31 (1833.61-7092.92) | 1376 (631-2097) | 5693.81 (2551.19-8791.97) | 0.88 (0.8-0.96) |
| Palestine | 29071 (14602-45755) | 3298.8 (1657.94-5194.19) | 94731 (46691-135238) | 3552.55 (1761.38-5179.61) | 0.2 (0.08-0.33) |
| Panama | 20977 (8624-33999) | 1367.3 (545.52-2220.85) | 89750 (38611-136160) | 2024.91 (873.9-3071.11) | 1.26 (1.2-1.33) |
| Papua New Guinea | 49026 (22552-83703) | 2149.19 (988.15-3667.07) | 183629 (85400-293859) | 2724.21 (1236.27-4425.96) | 0.75 (0.7-0.79) |
| Paraguay | 35907 (16268-55446) | 1517.97 (696.3-2353.55) | 151495 (67556-232357) | 2503.77 (1114.96-3855.23) | 1.81 (1.68-1.93) |
| Peru | 154933 (69554-260701) | 1154.86 (501.74-1951.19) | 473344 (208804-730964) | 1369.67 (602.05-2118.83) | 0.37 (0.15-0.59) |
| Philippines | 363905 (188131-563217) | 1031.24 (556.02-1590.45) | 1639654 (800577-2580342) | 1781.95 (875.83-2818.38) | 1.87 (1.78-1.96) |
| Poland | 975734 (405103-1603196) | 2259.17 (935.05-3707.98) | 1182035 (494685-1891319) | 1749.79 (735.98-2798.6) | -0.94 (-1.06--0.81) |
| Portugal | 168486 (63497-280461) | 1277.01 (485.02-2111.17) | 267307 (98095-435946) | 1247.75 (493.9-2015.05) | -0.27 (-0.35--0.19) |
| Puerto Rico | 89367 (41750-134266) | 2503.85 (1169.37-3759.1) | 160114 (73477-239035) | 2670.19 (1298.23-3921.12) | 0.06 (-0.02-0.13) |
| Qatar | 5257 (2364-8169) | 3656.33 (1695.01-5780.45) | 49411 (23324-74875) | 3590.91 (1666.21-5417.77) | -0.34 (-0.78-0.1) |
| Romania | 583556 (270713-934147) | 2150.14 (997.94-3442.25) | 726905 (345502-1140312) | 2101.16 (964.96-3315.2) | -0.34 (-0.47--0.21) |
| Russian Federation | 3289802 (1133746-5480669) | 1856.77 (638.21-3092.95) | 5039129 (1850678-8141752) | 2178.72 (800.44-3519.73) | -0.02 (-0.42-0.39) |
| Rwanda | 41728 (24834-64648) | 1246.04 (732.68-1900.97) | 77315 (41138-126758) | 1074.1 (579.76-1753.47) | -1.48 (-1.87--1.09) |
| Saint Kitts and Nevis | 1038 (493-1601) | 2962.53 (1419.38-4511.87) | 2235 (1137-3284) | 3085.24 (1569.95-4591.16) | 0.37 (0.18-0.56) |
| Saint Lucia | 2394 (1205-3530) | 2726.85 (1381.95-4032.59) | 6487 (3260-9453) | 2714.18 (1374.22-3956.83) | -0.25 (-0.42--0.07) |
| Saint Vincent and the Grenadines | 1671 (851-2455) | 2343.01 (1195.07-3434.21) | 4313 (2344-6191) | 3059.61 (1687.13-4378.26) | 0.69 (0.52-0.85) |
| Samoa | 4153 (1954-6208) | 4395.12 (2053.97-6668.01) | 9179 (4505-13564) | 5827.78 (2845.89-8593.76) | 0.92 (0.88-0.96) |
| San Marino | 311 (115-511) | 929.19 (347.54-1526.39) | 572 (191-954) | 878.76 (303.36-1479.38) | 0.11 (-0.01-0.23) |
| Sao Tome and Principe | 816 (418-1299) | 1224.24 (624.88-1952.5) | 2577 (1295-4046) | 2045.54 (1015.76-3249.55) | 1.51 (1.39-1.63) |
| Saudi Arabia | 205187 (100061-321201) | 3000.56 (1463.62-4729.07) | 1219797 (640155-1800137) | 4588.42 (2427.19-6769.53) | 1.3 (1.17-1.44) |
| Senegal | 51608 (26205-85821) | 1444.33 (740.99-2390.05) | 170137 (86864-261669) | 1991.81 (1000.58-3098.36) | 1.04 (0.97-1.11) |
| Serbia | 242404 (106923-390460) | 2248.87 (1028.81-3612.44) | 382365 (167406-606887) | 2418.48 (1051.2-3832.91) | -0.12 (-0.28-0.05) |
| Seychelles | 1174 (774-1637) | 2074.71 (1367.19-2887.91) | 3391 (1885-4812) | 2795.5 (1575.23-3980.32) | 1.03 (0.93-1.13) |
| Sierra Leone | 21408 (11252-35017) | 984.03 (517.7-1631.67) | 69155 (34779-113586) | 1580.95 (798.7-2634.58) | 1.68 (1.55-1.82) |
| Singapore | 21670 (9844-34248) | 857.7 (394.78-1356.34) | 76881 (30346-123394) | 900.61 (356.71-1442.76) | 0.07 (-0.02-0.15) |
| Slovakia | 152169 (62610-245677) | 2574.3 (1057.4-4161.08) | 181107 (75096-292302) | 1973.99 (816.06-3188.78) | -0.86 (-0.92--0.8) |
| Slovenia | 41349 (17233-67330) | 1696.05 (708.58-2764.86) | 56558 (22857-89666) | 1361.1 (558.46-2164.88) | -0.96 (-1.04--0.87) |
| Solomon Islands | 4949 (2104-8424) | 2935.64 (1285.75-4977.46) | 18812 (8405-29167) | 4266.86 (1868.54-6698.7) | 1.23 (1.15-1.31) |
| Somalia | 39548 (23310-67691) | 1231.87 (738.73-2036.45) | 129906 (70033-223738) | 1607.39 (874.16-2755.29) | 0.82 (0.78-0.86) |
| South Africa | 509561 (237943-803015) | 2225.91 (1038.15-3422.3) | 1674546 (850082-2484720) | 3473.43 (1784.89-5131.83) | 1.55 (1.18-1.93) |
| South Korea | 248158 (118269-413968) | 775.12 (376.61-1291.22) | 731078 (268234-1222506) | 835.24 (314.79-1390.69) | 0.04 (-0.06-0.13) |
| South Sudan | 20069 (12330-32365) | 705.16 (423.11-1149.47) | 46615 (26931-76690) | 997.92 (568.24-1650.79) | 0.98 (0.71-1.26) |
| Spain | 640874 (236419-1054341) | 1227.96 (455.79-2018.83) | 1079070 (390944-1746004) | 1198.68 (460.34-1914) | -0.2 (-0.25--0.14) |
| Sri Lanka | 111671 (57715-170028) | 949.7 (492.94-1451.64) | 409100 (178665-675034) | 1508.08 (655.31-2520.98) | 1.65 (1.49-1.81) |
| Sudan | 250425 (134384-388871) | 2478.29 (1373.22-3816.79) | 715534 (361565-1097122) | 3169.25 (1612.03-4835.89) | 0.73 (0.65-0.81) |
| Suriname | 4835 (2438-7223) | 1744.07 (880.78-2612.74) | 16627 (8072-25110) | 2539.14 (1236-3849.99) | 1.32 (1.17-1.47) |
| Swaziland | 9399 (5042-15088) | 3133.66 (1675.19-4945.09) | 33100 (16826-53625) | 5505.51 (2740.44-8643.37) | 2.22 (1.6-2.85) |
| Sweden | 147969 (54237-249341) | 1071.02 (402.48-1785.82) | 174927 (66177-287294) | 905.15 (357.5-1471.79) | -0.45 (-0.53--0.37) |
| Switzerland | 104230 (38799-170412) | 1052.95 (399.84-1717.65) | 147488 (54642-242391) | 889.59 (331.37-1432.58) | -0.55 (-0.62--0.49) |
| Syria | 169671 (79873-260929) | 2931.74 (1384.17-4533.41) | 551401 (268701-859308) | 4126.38 (1994.23-6397.06) | 0.78 (0.59-0.97) |
| Taiwan | 165552 (74182-260697) | 1017.68 (457.09-1615.3) | 502478 (212167-797795) | 1252.34 (525.37-1981.58) | 0.5 (0.39-0.61) |
| Tajikistan | 61874 (29511-97363) | 2186.85 (1048.62-3450.63) | 132378 (61441-211039) | 2100.86 (995.51-3332.62) | -0.46 (-0.71--0.21) |
| Tanzania | 133201 (76308-208984) | 1131.35 (653.48-1753.64) | 493741 (251925-807221) | 1736.81 (872.52-2829.21) | 1.32 (1.27-1.38) |
| Thailand | 263053 (113058-435119) | 646.64 (279.16-1086.78) | 1228566 (521849-1985448) | 1176 (501.25-1895.21) | 1.56 (1.35-1.76) |
| The Bahamas | 4840 (2753-6931) | 2844.54 (1617.61-4088) | 14665 (8843-20534) | 3418.86 (2045.66-4786.78) | 0.63 (0.58-0.69) |
| The Gambia | 5490 (2725-9133) | 1361.81 (684.35-2253.8) | 25581 (13380-41389) | 2280.7 (1184.86-3734.76) | 1.52 (1.36-1.68) |
| Timor-Leste | 1329 (806-2098) | 364.72 (215.17-565.26) | 6338 (3409-10300) | 687.31 (374.36-1117.79) | 2.26 (1.96-2.55) |
| Togo | 16043 (8747-26159) | 1130.11 (620.34-1825.04) | 80110 (40398-125752) | 1840.88 (925.63-2897.47) | 1.55 (1.44-1.66) |
| Tokelau | 53 (25-85) | 4022.81 (1900.72-6462.46) | 73 (34-108) | 5056.21 (2373.45-7494.74) | 0.65 (0.59-0.72) |
| Tonga | 2297 (1028-3364) | 3772.41 (1661.66-5558.39) | 4203 (1991-6018) | 5044.92 (2366.21-7240.97) | 0.93 (0.83-1.02) |
| Trinidad and Tobago | 33252 (16163-47800) | 3855.71 (1866.1-5545.61) | 73549 (37182-108050) | 3848.46 (1963.72-5654.52) | -0.34 (-0.49--0.18) |
| Tunisia | 76806 (40540-117292) | 1477.27 (801.9-2242.62) | 321597 (156063-488159) | 2417.08 (1162.81-3681.22) | 1.51 (1.46-1.55) |
| Turkey | 859011 (391894-1305711) | 2394.2 (1120.67-3624.7) | 2267677 (1007432-3349038) | 2428.92 (1080.39-3601.04) | 0.02 (-0.23-0.28) |
| Turkmenistan | 44673 (18472-73077) | 2203.16 (905.17-3620.92) | 116096 (49540-190557) | 2669.94 (1136.81-4368.97) | 0.04 (-0.24-0.32) |
| Tuvalu | 366 (161-595) | 4854.83 (2153.33-7906.42) | 642 (293-983) | 5795.7 (2622.33-8865.75) | 0.64 (0.61-0.67) |
| Uganda | 57619 (32244-95826) | 777.29 (424.49-1309.49) | 217031 (110079-346776) | 1209.55 (603.57-1917.58) | 0.92 (0.69-1.14) |
| Ukraine | 1273672 (432992-2152998) | 1827.08 (621.52-3096.07) | 1756399 (660248-2922712) | 2368.71 (895.85-3951.36) | 0.38 (0.02-0.73) |
| United Arab Emirates | 18010 (8179-28708) | 2758.16 (1278.77-4368.37) | 188914 (90912-280851) | 3406.96 (1721.48-5017.12) | 1.63 (1.27-1.99) |
| United Kingdom | 1247018 (464972-2079174) | 1513.13 (569.94-2515.3) | 1434450 (527711-2330464) | 1295.59 (499.42-2077.78) | -0.6 (-0.73--0.47) |
| United States | 5376154 (2154171-8778068) | 1787.33 (727.69-2908.24) | 11642980 (5084552-17521737) | 2231.89 (1014.46-3323.49) | 0.66 (0.6-0.72) |
| Uruguay | 58449 (22747-95217) | 1553.83 (601.74-2538.07) | 84746 (34724-134788) | 1660.93 (686.76-2632.34) | 0.18 (0.11-0.25) |
| Uzbekistan | 234845 (93841-379725) | 1975.84 (790.18-3206.47) | 767281 (347840-1196294) | 2727.9 (1214.87-4298.59) | 0.92 (0.65-1.19) |
| Vanuatu | 2233 (959-3818) | 2916.48 (1269.18-5020.18) | 9075 (4163-13687) | 4293.7 (1972.73-6546.73) | 1.14 (1.08-1.2) |
| Venezuela | 237248 (116601-361197) | 2224.91 (1106.18-3368.71) | 869465 (430670-1314444) | 2855.82 (1412.2-4328.02) | 0.55 (0.36-0.75) |
| Vietnam | 134106 (79321-215825) | 324.11 (191.41-520.11) | 677733 (321526-1085593) | 648.66 (308.21-1045.01) | 2.71 (2.45-2.96) |
| Virgin Islands, U.S. | 2581 (1324-3789) | 2884.99 (1466.28-4301.82) | 4227 (1991-6154) | 2731.18 (1335.16-3932.61) | -0.06 (-0.14-0.03) |
| Yemen | 90655 (49197-139812) | 1659.38 (926.71-2504.08) | 392532 (184440-616039) | 2477.97 (1233.98-3855.24) | 1.16 (1-1.33) |
| Zambia | 42846 (26665-67665) | 1287.84 (794.59-2014.25) | 175955 (95075-273692) | 2112.84 (1157.16-3276.1) | 1.16 (0.98-1.34) |
| Zimbabwe | 48578 (25478-78524) | 1120.4 (604.85-1812.7) | 233037 (115836-381217) | 3029.86 (1523.15-4939.64) | 3.95 (3.33-4.57) |
| GBD Regions | | | | | |
| Advanced Health System | 23921276 (9498216-39134415) | 1502.5 (597.02-2455.08) | 41206177 (16736117-64083506) | 1608.85 (678.39-2493.82) | 0.05 (-0.03-0.12) |
| Africa | 5037264 (2618826-7851120) | 1639.26 (874.05-2528.1) | 17235050 (8674459-26019866) | 2415.7 (1222.07-3635.08) | 1.28 (1.22-1.35) |
| African Region | 3214206 (1751603-5125743) | 1319.78 (722.97-2090.26) | 11016944 (5441797-17063112) | 1957.22 (965.59-3007.78) | 1.22 (1.12-1.32) |
| America | 11053657 (4734175-17540207) | 1798.72 (767.84-2849.48) | 27773302 (12254318-41663834) | 2143.86 (956.79-3203.34) | 0.49 (0.45-0.54) |
| Andean Latin America | 312618 (142533-508119) | 1367.93 (612.38-2231.67) | 1048226 (480394-1590143) | 1723.61 (789.03-2619.71) | 0.72 (0.65-0.79) |
| Asia | 15473491 (7837999-24785786) | 711.16 (374.53-1134.51) | 59710204 (25620572-95011467) | 1159.86 (499.59-1848.53) | 1.62 (1.59-1.65) |
| Australasia | 311857 (114551-522541) | 1364.35 (501.64-2285.51) | 605384 (207661-990316) | 1256.62 (441.9-2038.09) | -0.32 (-0.37--0.27) |
| Basic Health System | 16491912 (8294146-25648178) | 1050.92 (545.96-1636.62) | 56943171 (25399952-87195862) | 1510.53 (678.09-2314.66) | 1.17 (1.15-1.19) |
| Caribbean | 473460 (216070-710948) | 1759.22 (802.7-2648.2) | 1231093 (600115-1826664) | 2305.98 (1124.92-3419.62) | 0.92 (0.85-0.99) |
| Central Africa | 459957 (246828-762627) | 1479.87 (813.2-2449.11) | 1785138 (885590-2845306) | 2359.1 (1173.55-3735.15) | 1.41 (1.33-1.49) |
| Central Asia | 984940 (408112-1609285) | 2043.14 (842.16-3334.96) | 2022675 (895461-3182400) | 2365.62 (1041.99-3748.97) | 0.09 (-0.2-0.39) |
| Central Europe | 3370393 (1453599-5502509) | 2284.76 (985.53-3732.47) | 4248517 (1933705-6653519) | 2036.74 (931.87-3193.64) | -0.48 (-0.52--0.43) |
| Central Latin America | 1925817 (899449-2924999) | 2097.86 (981.5-3175.77) | 6889701 (3349891-10070474) | 2674.72 (1295.95-3920.76) | 0.68 (0.59-0.76) |
| Central Sub-Saharan Africa | 372601 (208313-606473) | 1479.39 (837.48-2389.36) | 1487254 (743299-2319090) | 2369.32 (1218.46-3671.81) | 1.42 (1.38-1.47) |
| Commonwealth High Income | 2039116 (766187-3376739) | 1428.5 (539.53-2361.61) | 3047373 (1143273-4933349) | 1280.94 (498.58-2044.24) | -0.45 (-0.54--0.35) |
| Commonwealth Low Income | 654548 (377679-1058991) | 658.8 (376.35-1058.86) | 2854445 (1358403-4601684) | 1102.8 (528.03-1777.27) | 1.74 (1.7-1.78) |
| Commonwealth Middle Income | 5039006 (2278850-8235261) | 718.31 (338.05-1180.48) | 21966262 (9791597-34101904) | 1299.09 (584.49-2020.59) | 1.99 (1.93-2.06) |
| East Asia | 5685520 (3298178-9013076) | 630.1 (368.15-987.5) | 21676486 (8864964-34999023) | 1031.68 (430.7-1641.21) | 1.68 (1.6-1.76) |
| East Asia & Pacific | 9355385 (5071902-14890995) | 661.44 (369.42-1046.85) | 33637230 (14155754-53496203) | 1042.89 (445.03-1658.68) | 1.51 (1.46-1.57) |
| Eastern Africa | 990813 (575501-1570219) | 1260.81 (736.62-1962.03) | 2803100 (1425847-4432325) | 1526.26 (776.04-2396.46) | 0.43 (0.36-0.5) |
| Eastern Europe | 5063184 (1754941-8435605) | 1849.96 (642.12-3084.37) | 7518384 (2799823-12148327) | 2222.64 (828.99-3581.77) | 0.09 (-0.27-0.46) |
| Eastern Mediterranean Region | 4063409 (2105438-6297177) | 2076.15 (1104.35-3172.02) | 15556172 (7748820-23217001) | 3098.09 (1545.39-4645.54) | 1.35 (1.32-1.39) |
| Eastern Sub-Saharan Africa | 886192 (531823-1401682) | 1036.12 (621.67-1623.93) | 2609975 (1350808-4226127) | 1327.51 (703.21-2136.32) | 0.61 (0.54-0.68) |
| Europe | 16290909 (6402478-26641699) | 1614.25 (633.98-2637.26) | 23467231 (9240490-37688539) | 1592.49 (642.4-2552.99) | -0.27 (-0.39--0.16) |
| Europe & Central Asia | 16896387 (6653957-27618056) | 1625.93 (639.84-2655.38) | 24876740 (9875642-39901450) | 1627.6 (660.6-2602.64) | -0.24 (-0.36--0.12) |
| European Region | 17014959 (6704078-27811363) | 1626.29 (640.29-2655.98) | 25103150 (9965405-40264964) | 1625.36 (659.58-2599.19) | -0.24 (-0.37--0.12) |
| High-income Asia Pacific | 1192636 (521139-1936384) | 595.05 (263.75-965.14) | 2458879 (934790-4168522) | 662.64 (253.69-1110.48) | 0.26 (0.15-0.37) |
| High-income North America | 5768932 (2297070-9443800) | 1732.43 (699.84-2825.85) | 12419109 (5390741-18773941) | 2121.2 (959.21-3167.3) | 0.59 (0.53-0.65) |
| Latin America & Caribbean | 5377646 (2432184-8343024) | 1879.95 (848.01-2911.05) | 15519803 (7105891-23201348) | 2175.46 (995.96-3252.7) | 0.42 (0.37-0.46) |
| Limited Health System | 6627893 (3156402-10880838) | 741.32 (364.23-1221.21) | 27364084 (12257586-42536925) | 1263.9 (575.12-1960.27) | 1.76 (1.74-1.79) |
| Middle East & North Africa | 3255227 (1656333-4898324) | 2470.53 (1295.61-3683.81) | 12410447 (6200342-18321348) | 3342.05 (1673.69-4971.85) | 1.02 (0.99-1.04) |
| Minimal Health System | 912066 (520844-1492802) | 1370.19 (787.85-2230.35) | 2849909 (1476947-4507179) | 1927.38 (988.12-3050.33) | 1.05 (1-1.09) |
| North Africa and Middle East | 4542622 (2313706-6876469) | 2526.69 (1321.48-3786.86) | 15774861 (7841442-23600021) | 3247.44 (1609.13-4855.73) | 0.84 (0.77-0.91) |
| North America | 5769306 (2297308-9444275) | 1732.45 (699.87-2825.85) | 12420029 (5391130-18775327) | 2121.21 (959.22-3167.3) | 0.59 (0.53-0.65) |
| Northern Africa | 1781928 (929079-2642321) | 2722.68 (1474.96-4019.06) | 6352416 (3268941-9418879) | 3916.41 (2043.36-5804.41) | 1.35 (1.29-1.42) |
| Oceania | 108923 (50157-172855) | 3047.56 (1386.13-4872.33) | 340541 (157517-523089) | 3712.96 (1666.5-5765.84) | 0.61 (0.54-0.67) |
| Region of the Americas | 11053657 (4734175-17540207) | 1798.72 (767.84-2849.48) | 27773302 (12254318-41663834) | 2143.86 (956.79-3203.34) | 0.49 (0.45-0.54) |
| South-East Asia Region | 4593851 (2116288-7604202) | 560.37 (268.19-916.8) | 20915073 (9006550-32688420) | 1076.44 (467.7-1684.15) | 2.21 (2.17-2.25) |
| South Asia | 3975146 (1804108-6651644) | 583.81 (274.62-975.65) | 18483674 (8019245-28837091) | 1127.77 (490.98-1762.72) | 2.27 (2.22-2.33) |
| South Asia region | 3612123 (1610431-6096514) | 543.83 (252.26-913.62) | 17553020 (7566331-27474915) | 1098.12 (475.32-1719.6) | 2.43 (2.37-2.49) |
| Southeast Asia | 2177745 (1159586-3455238) | 740.84 (398.94-1180.56) | 9014576 (4148063-13947219) | 1269.32 (583.94-1976.77) | 1.74 (1.66-1.81) |
| Southern Africa | 810302 (419910-1275550) | 1674.38 (874.62-2603.35) | 2883040 (1451912-4429258) | 2822.07 (1450.59-4282.15) | 1.8 (1.48-2.13) |
| Southern Latin America | 844282 (359347-1334478) | 1825.09 (774.16-2888.13) | 1489915 (626386-2282434) | 1759.71 (746.03-2689.9) | -0.04 (-0.08-0.01) |
| Southern Sub-Saharan Africa | 606937 (289409-953982) | 2040.75 (971.71-3159.15) | 2073635 (1045991-3137680) | 3430.11 (1755.89-5152.18) | 1.88 (1.48-2.27) |
| Sub-Saharan Africa | 3277068 (1774620-5235071) | 1342.2 (729.73-2127.32) | 10929575 (5382963-16933215) | 1955.99 (965.85-3021.25) | 1.16 (1.06-1.26) |
| Tropical Latin America | 1838889 (833463-2838294) | 1836.08 (840.81-2832.39) | 4904801 (2091691-7456828) | 1890.68 (805.82-2880.38) | 0.03 (-0.01-0.07) |
| Western Africa | 994264 (526918-1585780) | 1157.32 (631.12-1845.95) | 3411357 (1655801-5327963) | 1718.63 (826.41-2715.57) | 1.19 (1.12-1.26) |
| Western Europe | 6818563 (2621180-11138897) | 1240.95 (479.52-2025.9) | 9166272 (3338023-15009908) | 1091.32 (418.75-1768.67) | -0.45 (-0.5--0.4) |
| Western Pacific Region | 7748792 (4221328-12290772) | 647.05 (355.57-1024.67) | 27317521 (11162601-43558710) | 1002.14 (415.71-1601.08) | 1.48 (1.4-1.57) |
| Western Sub-Saharan Africa | 1143874 (600722-1833836) | 1205.08 (649.75-1922.75) | 3986780 (1950944-6222845) | 1795.38 (867.73-2815.83) | 1.2 (1.12-1.28) |
| World Bank High Income | 17715930 (7125544-28934966) | 1424.89 (574.46-2324.62) | 31012784 (12733882-48362088) | 1498.81 (645.92-2317.86) | 0.11 (0.05-0.17) |
| World Bank Low Income | 2053763 (1165397-3301177) | 1261.05 (719.42-2012.28) | 6113062 (3074235-9564399) | 1659.68 (840.85-2565.13) | 0.72 (0.63-0.81) |
| World Bank Lower Middle Income | 10826160 (4994941-17512474) | 961.59 (451.39-1549.51) | 41488202 (19083293-64320110) | 1537.93 (702.6-2375.35) | 1.53 (1.49-1.57) |
| World Bank Upper Middle Income | 17356323 (7982434-27599292) | 1124.68 (524.91-1790.28) | 49747756 (21247660-77785645) | 1440.86 (614.84-2242.13) | 0.64 (0.56-0.71) |

No.: Number, ASDR: Age-standardized DALYs Rate, EAPC: Estimated Annual Percentage Changes, SDI: Social Demographic Index.

**Supplementary table 3. Percentage composition of the impact of high BMI on disease.**

|  | | Deaths | | DALYs | |
| --- | --- | --- | --- | --- | --- |
| **year** | **location** | **cause** | **percent** | **cause** | **percent** |
| 1990 | Global | Atrial fibrillation and flutter | 0.003287404 | Atrial fibrillation and flutter | 0.003463102 |
| 1990 | Global | Gallbladder and biliary tract cancer | 0.005561468 | Gallbladder and biliary tract cancer | 0.004503378 |
| 1990 | Global | Acute lymphoid leukemia | 0.001227839 | Acute lymphoid leukemia | 0.001706495 |
| 1990 | Global | Ovarian cancer | 0.003935617 | Ovarian cancer | 0.003736983 |
| 1990 | Global | Chronic lymphoid leukemia | 0.001431298 | Chronic lymphoid leukemia | 0.001170066 |
| 1990 | Global | Kidney cancer | 0.006958541 | Kidney cancer | 0.006293189 |
| 1990 | Global | Liver cancer due to alcohol use | 0.001428811 | Liver cancer due to alcohol use | 0.001305675 |
| 1990 | Global | Aortic aneurysm | 0.003693541 | Aortic aneurysm | 0.002715404 |
| 1990 | Global | Liver cancer due to hepatitis B | 0.001958309 | Liver cancer due to hepatitis B | 0.002284414 |
| 1990 | Global | Thyroid cancer | 0.001262965 | Thyroid cancer | 0.001223038 |
| 1990 | Global | Acute myeloid leukemia | 0.002543031 | Acute myeloid leukemia | 0.002733854 |
| 1990 | Global | Gallbladder and biliary diseases | 0.012124277 | Gallbladder and biliary diseases | 0.026201683 |
| 1990 | Global | Chronic kidney disease due to hypertension | 0.020957038 | Chronic kidney disease due to hypertension | 0.019951226 |
| 1990 | Global | Diabetes mellitus type 2 | 0.13681802 | Diabetes mellitus type 2 | 0.206460236 |
| 1990 | Global | Total Cancers excluding Non-melanoma skin cancer | 0.078914562 | Total Cancers excluding Non-melanoma skin cancer | 0.070219986 |
| 1990 | Global | Liver cancer due to hepatitis C | 0.002203266 | Liver cancer due to hepatitis C | 0.0018698 |
| 1990 | Global | Other non-Hodgkin lymphoma | 0.002894265 | Other non-Hodgkin lymphoma | 0.002840649 |
| 1990 | Global | Burkitt lymphoma | 0.0000264 | Burkitt lymphoma | 0.000033 |
| 1990 | Global | Chronic myeloid leukemia | 0.001179495 | Chronic myeloid leukemia | 0.001223289 |
| 1990 | Global | Chronic kidney disease due to other and unspecified causes | 0.005667238 | Chronic kidney disease due to other and unspecified causes | 0.006109553 |
| 1990 | Global | Asthma | 0.021855741 | Asthma | 0.044595449 |
| 1990 | Global | Total burden related to hepatitis C | 0.002203266 | Total burden related to hepatitis C | 0.0018698 |
| 1990 | Global | Total burden related to hepatitis B | 0.001958309 | Total burden related to hepatitis B | 0.002284414 |
| 1990 | Global | Ischemic stroke | 0.050602421 | Ischemic stroke | 0.04206182 |
| 1990 | Global | Lower extremity peripheral arterial disease | 0.003258592 | Lower extremity peripheral arterial disease | 0.002518825 |
| 1990 | Global | Uterine cancer | 0.007982235 | Uterine cancer | 0.00737292 |
| 1990 | Global | Colon and rectum cancer | 0.023863884 | Colon and rectum cancer | 0.020083195 |
| 1990 | Global | Chronic kidney disease due to diabetes mellitus type 2 | 0.02325645 | Chronic kidney disease due to diabetes mellitus type 2 | 0.023702575 |
| 1990 | Global | Alzheimer's disease and other dementias | 0.018142439 | Alzheimer's disease and other dementias | 0.012756757 |
| 1990 | Global | Liver cancer due to other causes | 0.000317083 | Liver cancer due to other causes | 0.000331277 |
| 1990 | Global | Multidrug-resistant tuberculosis without extensive drug resistance | 0.000267735 | Multidrug-resistant tuberculosis without extensive drug resistance | 0.000287521 |
| 1990 | Global | Drug-susceptible tuberculosis | 0.021721127 | Drug-susceptible tuberculosis | 0.025356808 |
| 1990 | Global | Chronic kidney disease due to glomerulonephritis | 0.002968876 | Chronic kidney disease due to glomerulonephritis | 0.003065815 |
| 1990 | Global | Hypertensive heart disease | 0.137944326 | Hypertensive heart disease | 0.112105416 |
| 1990 | Global | Multiple myeloma | 0.001737109 | Multiple myeloma | 0.001424758 |
| 1990 | Global | Total cancers | 0.078914562 | Total cancers | 0.070219986 |
| 1990 | Global | Ischemic heart disease | 0.280949536 | Ischemic heart disease | 0.235751237 |
| 1990 | Global | Other leukemia | 0.001084615 | Other leukemia | 0.001160387 |
| 1990 | Global | Subarachnoid hemorrhage | 0.002555649 | Subarachnoid hemorrhage | 0.003504866 |
| 1990 | Global | Intracerebral hemorrhage | 0.01357281 | Intracerebral hemorrhage | 0.015011875 |
| 1990 | Global | Breast cancer | 0.010769863 | Breast cancer | 0.008489319 |
| 2021 | Global | Ischemic heart disease | 0.221775508 | Ischemic heart disease | 0.179394339 |
| 2021 | Global | Ischemic heart disease | 0.221581149 | Chronic kidney disease due to hypertension | 0.020719647 |
| 2021 | Global | Diabetes mellitus type 2 | 0.162794709 | Diabetes mellitus type 2 | 0.294874041 |
| 2021 | Global | Diabetes mellitus type 2 | 0.160059684 | Total burden related to hepatitis B | 0.002099575 |
| 2021 | Global | Hypertensive heart disease | 0.136401162 | Uterine cancer | 0.007308636 |
| 2021 | Global | Hypertensive heart disease | 0.13381771 | Hypertensive heart disease | 0.094155682 |
| 2021 | Global | Total Cancers excluding Non-melanoma skin cancer | 0.080245393 | Total Cancers excluding Non-melanoma skin cancer | 0.066721365 |
| 2021 | Global | Total cancers | 0.080245393 | Total cancers | 0.066721365 |
| 2021 | Global | Total Cancers excluding Non-melanoma skin cancer | 0.079027918 | Chronic kidney disease due to hypertension | 0.03193373 |
| 2021 | Global | Total cancers | 0.079027918 | Chronic kidney disease due to diabetes mellitus type 2 | 0.032429118 |
| 2021 | Global | Chronic kidney disease due to hypertension | 0.041373543 | Ischemic stroke | 0.033300101 |
| 2021 | Global | Chronic kidney disease due to hypertension | 0.040441914 | Alzheimer's disease and other dementias | 0.019996823 |
| 2021 | Global | Chronic kidney disease due to diabetes mellitus type 2 | 0.039257947 | Colon and rectum cancer | 0.017738286 |
| 2021 | Global | Chronic kidney disease due to diabetes mellitus type 2 | 0.038974125 | Intracerebral hemorrhage | 0.020393382 |
| 2021 | Global | Ischemic stroke | 0.03893123 | Asthma | 0.024629305 |
| 2021 | Global | Ischemic stroke | 0.038777877 | Drug-susceptible tuberculosis | 0.013942476 |
| 2021 | Global | Alzheimer's disease and other dementias | 0.033878023 | Gallbladder and biliary diseases | 0.021021863 |
| 2021 | Global | Alzheimer's disease and other dementias | 0.031365722 | Breast cancer | 0.007811272 |
| 2021 | Global | Colon and rectum cancer | 0.022329529 | Chronic kidney disease due to other and unspecified causes | 0.008934889 |
| 2021 | Global | Colon and rectum cancer | 0.022192989 | Uterine cancer | 0.006602332 |
| 2021 | Global | Intracerebral hemorrhage | 0.019147252 | Kidney cancer | 0.005863296 |
| 2021 | Global | Intracerebral hemorrhage | 0.018491488 | Atrial fibrillation and flutter | 0.005435315 |
| 2021 | Global | Asthma | 0.014028095 | Chronic kidney disease due to glomerulonephritis | 0.00488604 |
| 2021 | Global | Asthma | 0.013948251 | Gallbladder and biliary tract cancer | 0.0033868 |
| 2021 | Global | Drug-susceptible tuberculosis | 0.011580054 | Chronic lymphoid leukemia | 0.001208372 |
| 2021 | Global | Drug-susceptible tuberculosis | 0.011326581 | Multidrug-resistant tuberculosis without extensive drug resistance | 0.000270218 |
| 2021 | Global | Gallbladder and biliary diseases | 0.010891508 | Total cancers | 0.069642819 |
| 2021 | Global | Gallbladder and biliary diseases | 0.010508347 | Subarachnoid hemorrhage | 0.003188817 |
| 2021 | Global | Breast cancer | 0.01005645 | Intracerebral hemorrhage | 0.014132161 |
| 2021 | Global | Chronic kidney disease due to other and unspecified causes | 0.009876823 | Chronic kidney disease due to other and unspecified causes | 0.00674174 |
| 2021 | Global | Breast cancer | 0.009786035 | Chronic kidney disease due to diabetes mellitus type 2 | 0.024525978 |
| 2021 | Global | Chronic kidney disease due to other and unspecified causes | 0.009554708 | Alzheimer's disease and other dementias | 0.017018996 |
| 2021 | Global | Uterine cancer | 0.007453333 | Ischemic heart disease | 0.239325654 |
| 2021 | Global | Uterine cancer | 0.007293344 | Other leukemia | 0.00106952 |
| 2021 | Global | Kidney cancer | 0.007279787 | Liver cancer due to alcohol use | 0.001270251 |
| 2021 | Global | Kidney cancer | 0.007188366 | Aortic aneurysm | 0.002828406 |
| 2021 | Global | Atrial fibrillation and flutter | 0.006600375 | Ischemic stroke | 0.044003021 |
| 2021 | Global | Atrial fibrillation and flutter | 0.006126695 | Atrial fibrillation and flutter | 0.00563385 |
| 2021 | Global | Chronic kidney disease due to glomerulonephritis | 0.005161471 | Total Cancers excluding Non-melanoma skin cancer | 0.069642819 |
| 2021 | Global | Chronic kidney disease due to glomerulonephritis | 0.005145307 | Other non-Hodgkin lymphoma | 0.002715038 |
| 2021 | Global | Gallbladder and biliary tract cancer | 0.004521769 | Total burden related to hepatitis C | 0.001866559 |
| 1990 | Global | Ischemic heart disease | 0.284785767 | Atrial fibrillation and flutter | 0.004133011 |
| 2021 | Global | Gallbladder and biliary tract cancer | 0.004483397 | Gallbladder and biliary tract cancer | 0.004565637 |
| 2021 | Global | Ovarian cancer | 0.003901498 | Acute lymphoid leukemia | 0.001424769 |
| 1990 | Global | Hypertensive heart disease | 0.141094941 | Ovarian cancer | 0.003639119 |
| 2021 | Global | Liver cancer due to hepatitis C | 0.003844226 | Kidney cancer | 0.006203012 |
| 1990 | Global | Diabetes mellitus type 2 | 0.130740665 | Liver cancer due to hepatitis B | 0.002099575 |
| 2021 | Global | Total burden related to hepatitis C | 0.003844226 | Thyroid cancer | 0.001188161 |
| 2021 | Global | Ovarian cancer | 0.003794881 | Acute myeloid leukemia | 0.002497826 |
| 1990 | Global | Total Cancers excluding Non-melanoma skin cancer | 0.075659943 | Gallbladder and biliary diseases | 0.025675858 |
| 1990 | Global | Total cancers | 0.075659943 | Diabetes mellitus type 2 | 0.200135943 |
| 1990 | Global | Ischemic stroke | 0.053143541 | Liver cancer due to hepatitis C | 0.001866559 |
| 2021 | Global | Liver cancer due to hepatitis C | 0.003783725 | Burkitt lymphoma | 0.0000293 |
| 2021 | Global | Total burden related to hepatitis C | 0.003783725 | Chronic myeloid leukemia | 0.001139325 |
| 2021 | Global | Liver cancer due to hepatitis B | 0.003531119 | Asthma | 0.040206947 |
| 2021 | Global | Total burden related to hepatitis B | 0.003531119 | Ovarian cancer | 0.00358003 |
| 1990 | Global | Alzheimer's disease and other dementias | 0.025192454 | Lower extremity peripheral arterial disease | 0.002940914 |
| 1990 | Global | Chronic kidney disease due to diabetes mellitus type 2 | 0.023925769 | Liver cancer due to hepatitis C | 0.002920018 |
| 1990 | Global | Colon and rectum cancer | 0.023539181 | Colon and rectum cancer | 0.020318823 |
| 1990 | Global | Chronic kidney disease due to hypertension | 0.022431157 | Liver cancer due to other causes | 0.000311391 |
| 2021 | Global | Liver cancer due to hepatitis B | 0.003407866 | Drug-susceptible tuberculosis | 0.023528272 |
| 1990 | Global | Asthma | 0.020726408 | Chronic kidney disease due to glomerulonephritis | 0.003017551 |
| 1990 | Global | Drug-susceptible tuberculosis | 0.019135167 | Hypertensive heart disease | 0.115153346 |
| 2021 | Global | Total burden related to hepatitis B | 0.003407866 | Multiple myeloma | 0.001441914 |
| 2021 | Global | Lower extremity peripheral arterial disease | 0.00312891 | Acute lymphoid leukemia | 0.001149614 |
| 1990 | Global | Gallbladder and biliary diseases | 0.013006791 | Breast cancer | 0.008904539 |
| 1990 | Global | Intracerebral hemorrhage | 0.011904334 | Total burden related to hepatitis C | 0.002920018 |
| 2021 | Global | Subarachnoid hemorrhage | 0.003112922 | Liver cancer due to alcohol use | 0.002027584 |
| 2021 | Global | Subarachnoid hemorrhage | 0.003027201 | Gallbladder and biliary diseases | 0.021266157 |
| 1990 | Global | Breast cancer | 0.010651839 | Asthma | 0.025481868 |
| 2021 | Global | Lower extremity peripheral arterial disease | 0.002989286 | Aortic aneurysm | 0.001860137 |
| 2021 | Global | Other non-Hodgkin lymphoma | 0.002878341 | Liver cancer due to hepatitis B | 0.003710575 |
| 1990 | Global | Uterine cancer | 0.007574999 | Liver cancer due to hepatitis B | 0.003749809 |
| 2021 | Global | Other non-Hodgkin lymphoma | 0.002856645 | Total burden related to hepatitis B | 0.003749809 |
| 2021 | Global | Acute myeloid leukemia | 0.002687465 | Lower extremity peripheral arterial disease | 0.002310304 |
| 2021 | Global | Acute myeloid leukemia | 0.002665564 | Chronic myeloid leukemia | 0.000418509 |
| 1990 | Global | Kidney cancer | 0.006540756 | Uterine cancer | 0.006507087 |
| 1990 | Global | Chronic kidney disease due to other and unspecified causes | 0.006296378 | Chronic kidney disease due to other and unspecified causes | 0.009180004 |
| 1990 | Global | Gallbladder and biliary tract cancer | 0.005433618 | Alzheimer's disease and other dementias | 0.021245087 |
| 2021 | Global | Aortic aneurysm | 0.002605027 | Subarachnoid hemorrhage | 0.003973432 |
| 2021 | Global | Aortic aneurysm | 0.002595826 | Liver cancer due to other causes | 0.000533688 |
| 1990 | Global | Atrial fibrillation and flutter | 0.004358104 | Chronic kidney disease due to glomerulonephritis | 0.004934716 |
| 1990 | Global | Lower extremity peripheral arterial disease | 0.003919022 | Lower extremity peripheral arterial disease | 0.002257273 |
| 2021 | Global | Liver cancer due to alcohol use | 0.002448727 | Other non-Hodgkin lymphoma | 0.002533571 |
| 2021 | Global | Liver cancer due to alcohol use | 0.00236124 | Hypertensive heart disease | 0.095251533 |
| 2021 | Global | Multiple myeloma | 0.0020617 | Intracerebral hemorrhage | 0.020163018 |
| 1990 | Global | Aortic aneurysm | 0.00375752 | Acute myeloid leukemia | 0.002406251 |
| 1990 | Global | Ovarian cancer | 0.003634184 | Diabetes mellitus type 2 | 0.292569933 |
| 2021 | Global | Multiple myeloma | 0.002040002 | Other leukemia | 0.000854654 |
| 2021 | Global | Multidrug-resistant tuberculosis without extensive drug resistance | 0.001225334 | Subarachnoid hemorrhage | 0.003969897 |
| 2021 | Global | Multidrug-resistant tuberculosis without extensive drug resistance | 0.00119643 | Aortic aneurysm | 0.001855556 |
| 2021 | Global | Thyroid cancer | 0.001182054 | Gallbladder and biliary tract cancer | 0.003363955 |
| 1990 | Global | Chronic kidney disease due to glomerulonephritis | 0.002956419 | Ovarian cancer | 0.003531581 |
| 2021 | Global | Thyroid cancer | 0.001171062 | Chronic lymphoid leukemia | 0.000785173 |
| 1990 | Global | Other non-Hodgkin lymphoma | 0.002702076 | Kidney cancer | 0.005813621 |
| 2021 | Global | Chronic lymphoid leukemia | 0.001083725 | Liver cancer due to alcohol use | 0.002072047 |
| 2021 | Global | Chronic lymphoid leukemia | 0.001063156 | Multiple myeloma | 0.001557545 |
| 2021 | Global | Other leukemia | 0.000943176 | Thyroid cancer | 0.001086392 |
| 1990 | Global | Acute myeloid leukemia | 0.002278009 | Acute myeloid leukemia | 0.002428235 |
| 1990 | Global | Subarachnoid hemorrhage | 0.002154799 | Chronic kidney disease due to hypertension | 0.032393347 |
| 1990 | Global | Liver cancer due to hepatitis C | 0.002091363 | Total Cancers excluding Non-melanoma skin cancer | 0.066051671 |
| 1990 | Global | Total burden related to hepatitis C | 0.002091363 | Liver cancer due to hepatitis C | 0.002883805 |
| 2021 | Global | Other leukemia | 0.000939019 | Other non-Hodgkin lymphoma | 0.00253943 |
| 1990 | Global | Multiple myeloma | 0.001681925 | Burkitt lymphoma | 0.0000444 |
| 1990 | Global | Liver cancer due to hepatitis B | 0.001671077 | Multidrug-resistant tuberculosis without extensive drug resistance | 0.001428285 |
| 1990 | Global | Total burden related to hepatitis B | 0.001671077 | Total burden related to hepatitis C | 0.002883805 |
| 1990 | Global | Chronic lymphoid leukemia | 0.001471894 | Total burden related to hepatitis B | 0.003710575 |
| 1990 | Global | Liver cancer due to alcohol use | 0.001293474 | Ischemic stroke | 0.033304652 |
| 1990 | Global | Thyroid cancer | 0.001197997 | Colon and rectum cancer | 0.017668 |
| 2021 | Global | Acute lymphoid leukemia | 0.000925926 | Chronic kidney disease due to diabetes mellitus type 2 | 0.032413804 |
| 2021 | Global | Acute lymphoid leukemia | 0.000918587 | Multidrug-resistant tuberculosis without extensive drug resistance | 0.00143165 |
| 1990 | Global | Chronic myeloid leukemia | 0.0010946 | Drug-susceptible tuberculosis | 0.013981437 |
| 2021 | Global | Liver cancer due to other causes | 0.000569115 | Thyroid cancer | 0.001087364 |
| 1990 | Global | Acute lymphoid leukemia | 0.000998529 | Multiple myeloma | 0.00154596 |
| 1990 | Global | Other leukemia | 0.000988021 | Total cancers | 0.066051671 |
| 2021 | Global | Liver cancer due to other causes | 0.00055229 | Ischemic heart disease | 0.179502161 |
| 2021 | Global | Chronic myeloid leukemia | 0.000459076 | Breast cancer | 0.007516423 |
| 2021 | Global | Chronic myeloid leukemia | 0.000456754 | Chronic lymphoid leukemia | 0.000775238 |
| 2021 | Global | Burkitt lymphoma | 0.0000383 | Other leukemia | 0.000846927 |
| 1990 | Global | Liver cancer due to other causes | 0.000282331 | Acute lymphoid leukemia | 0.001119972 |
| 1990 | Global | Multidrug-resistant tuberculosis without extensive drug resistance | 0.000239725 | Liver cancer due to other causes | 0.000539702 |
| 2021 | Global | Burkitt lymphoma | 0.000038 | Chronic myeloid leukemia | 0.00041158 |
| 1990 | Global | Burkitt lymphoma | 0.0000228 | Burkitt lymphoma | 0.0000438 |
| 1990 | High SDI | Atrial fibrillation and flutter | 0.00548978 | Atrial fibrillation and flutter | 0.006080455 |
| 1990 | High SDI | Gallbladder and biliary tract cancer | 0.007069787 | Gallbladder and biliary tract cancer | 0.005800634 |
| 1990 | High SDI | Acute lymphoid leukemia | 0.000809654 | Acute lymphoid leukemia | 0.001088074 |
| 1990 | High SDI | Ovarian cancer | 0.006245378 | Ovarian cancer | 0.00614116 |
| 1990 | High SDI | Chronic lymphoid leukemia | 0.00226378 | Chronic lymphoid leukemia | 0.001839001 |
| 1990 | High SDI | Kidney cancer | 0.011116377 | Kidney cancer | 0.01068005 |
| 1990 | High SDI | Liver cancer due to alcohol use | 0.001752007 | Liver cancer due to alcohol use | 0.00169757 |
| 1990 | High SDI | Aortic aneurysm | 0.007107175 | Aortic aneurysm | 0.00540453 |
| 1990 | High SDI | Liver cancer due to hepatitis B | 0.001208401 | Liver cancer due to hepatitis B | 0.001452601 |
| 1990 | High SDI | Thyroid cancer | 0.001331553 | Thyroid cancer | 0.001304055 |
| 1990 | High SDI | Acute myeloid leukemia | 0.003567098 | Acute myeloid leukemia | 0.003683155 |
| 1990 | High SDI | Gallbladder and biliary diseases | 0.011689373 | Gallbladder and biliary diseases | 0.027329403 |
| 1990 | High SDI | Chronic kidney disease due to hypertension | 0.016211874 | Chronic kidney disease due to hypertension | 0.015088799 |
| 1990 | High SDI | Diabetes mellitus type 2 | 0.106603636 | Diabetes mellitus type 2 | 0.164440551 |
| 1990 | High SDI | Total Cancers excluding Non-melanoma skin cancer | 0.110943692 | Total Cancers excluding Non-melanoma skin cancer | 0.100690257 |
| 1990 | High SDI | Liver cancer due to hepatitis C | 0.002320531 | Liver cancer due to hepatitis C | 0.002094024 |
| 1990 | High SDI | Other non-Hodgkin lymphoma | 0.004497963 | Other non-Hodgkin lymphoma | 0.004472613 |
| 1990 | High SDI | Burkitt lymphoma | 0.0000334 | Burkitt lymphoma | 0.0000417 |
| 1990 | High SDI | Chronic myeloid leukemia | 0.001687375 | Chronic myeloid leukemia | 0.001741483 |
| 1990 | High SDI | Chronic kidney disease due to other and unspecified causes | 0.006307587 | Chronic kidney disease due to other and unspecified causes | 0.006790419 |
| 1990 | High SDI | Asthma | 0.012684072 | Asthma | 0.048058998 |
| 1990 | High SDI | Total burden related to hepatitis C | 0.002320531 | Total burden related to hepatitis C | 0.002094024 |
| 1990 | High SDI | Total burden related to hepatitis B | 0.001208401 | Total burden related to hepatitis B | 0.001452601 |
| 1990 | High SDI | Ischemic stroke | 0.040493709 | Ischemic stroke | 0.034190629 |
| 1990 | High SDI | Lower extremity peripheral arterial disease | 0.004964659 | Lower extremity peripheral arterial disease | 0.004158949 |
| 1990 | High SDI | Uterine cancer | 0.009037547 | Uterine cancer | 0.008333379 |
| 1990 | High SDI | Colon and rectum cancer | 0.035898459 | Colon and rectum cancer | 0.031116749 |
| 1990 | High SDI | Chronic kidney disease due to diabetes mellitus type 2 | 0.018716845 | Chronic kidney disease due to diabetes mellitus type 2 | 0.020355249 |
| 1990 | High SDI | Alzheimer's disease and other dementias | 0.03032262 | Alzheimer's disease and other dementias | 0.022270264 |
| 1990 | High SDI | Liver cancer due to other causes | 0.000334776 | Liver cancer due to other causes | 0.000351016 |
| 1990 | High SDI | Multidrug-resistant tuberculosis without extensive drug resistance | 0.0000952 | Multidrug-resistant tuberculosis without extensive drug resistance | 9.84E-05 |
| 1990 | High SDI | Drug-susceptible tuberculosis | 0.003823042 | Drug-susceptible tuberculosis | 0.004016368 |
| 1990 | High SDI | Chronic kidney disease due to glomerulonephritis | 0.002566131 | Chronic kidney disease due to glomerulonephritis | 0.002485129 |
| 1990 | High SDI | Hypertensive heart disease | 0.069553673 | Hypertensive heart disease | 0.054492839 |
| 1990 | High SDI | Multiple myeloma | 0.003250021 | Multiple myeloma | 0.002817156 |
| 1990 | High SDI | Total cancers | 0.110943692 | Total cancers | 0.100690257 |
| 1990 | High SDI | Ischemic heart disease | 0.316285453 | Ischemic heart disease | 0.265364952 |
| 1990 | High SDI | Other leukemia | 0.000771587 | Other leukemia | 0.000686784 |
| 1990 | High SDI | Subarachnoid hemorrhage | 0.003098132 | Subarachnoid hemorrhage | 0.00464775 |
| 1990 | High SDI | Intracerebral hemorrhage | 0.009047213 | Intracerebral hemorrhage | 0.010446466 |
| 1990 | High SDI | Breast cancer | 0.016327827 | Breast cancer | 0.014011571 |
| 2021 | High SDI | Ischemic heart disease | 0.186666943 | Liver cancer due to alcohol use | 0.001691593 |
| 2021 | High SDI | Ischemic heart disease | 0.181209543 | Drug-susceptible tuberculosis | 0.004080904 |
| 2021 | High SDI | Total Cancers excluding Non-melanoma skin cancer | 0.127462127 | Intracerebral hemorrhage | 0.010777179 |
| 2021 | High SDI | Total cancers | 0.127462127 | Total Cancers excluding Non-melanoma skin cancer | 0.0997815 |
| 2021 | High SDI | Total Cancers excluding Non-melanoma skin cancer | 0.122832129 | Chronic myeloid leukemia | 0.001784512 |
| 2021 | High SDI | Total cancers | 0.122832129 | Total burden related to hepatitis B | 0.001524591 |
| 2021 | High SDI | Diabetes mellitus type 2 | 0.101919044 | Total cancers | 0.0997815 |
| 2021 | High SDI | Diabetes mellitus type 2 | 0.09899455 | Ischemic heart disease | 0.14011292 |
| 2021 | High SDI | Hypertensive heart disease | 0.095727213 | Total Cancers excluding Non-melanoma skin cancer | 0.099958025 |
| 2021 | High SDI | Hypertensive heart disease | 0.092427673 | Total cancers | 0.099958025 |
| 2021 | High SDI | Alzheimer's disease and other dementias | 0.057935215 | Diabetes mellitus type 2 | 0.257206977 |
| 2021 | High SDI | Chronic kidney disease due to hypertension | 0.049875505 | Hypertensive heart disease | 0.066302442 |
| 2021 | High SDI | Alzheimer's disease and other dementias | 0.048167101 | Alzheimer's disease and other dementias | 0.037223942 |
| 2021 | High SDI | Chronic kidney disease due to diabetes mellitus type 2 | 0.047075181 | Chronic kidney disease due to hypertension | 0.035195883 |
| 2021 | High SDI | Chronic kidney disease due to hypertension | 0.046140335 | Chronic kidney disease due to diabetes mellitus type 2 | 0.037257336 |
| 2021 | High SDI | Chronic kidney disease due to diabetes mellitus type 2 | 0.04481246 | Gallbladder and biliary tract cancer | 0.005667452 |
| 2021 | High SDI | Colon and rectum cancer | 0.035358436 | Colon and rectum cancer | 0.027241099 |
| 2021 | High SDI | Colon and rectum cancer | 0.034379437 | Ischemic stroke | 0.023225103 |
| 2021 | High SDI | Ischemic stroke | 0.025032902 | Alzheimer's disease and other dementias | 0.021953835 |
| 2021 | High SDI | Ischemic stroke | 0.023573673 | Breast cancer | 0.011956429 |
| 2021 | High SDI | Breast cancer | 0.01467657 | Gallbladder and biliary diseases | 0.024818677 |
| 2021 | High SDI | Breast cancer | 0.014557793 | Kidney cancer | 0.010973879 |
| 2021 | High SDI | Kidney cancer | 0.014164807 | Atrial fibrillation and flutter | 0.011383451 |
| 2021 | High SDI | Gallbladder and biliary diseases | 0.013731548 | Chronic kidney disease due to other and unspecified causes | 0.009896301 |
| 2021 | High SDI | Kidney cancer | 0.013595089 | Uterine cancer | 0.009977604 |
| 2021 | High SDI | Atrial fibrillation and flutter | 0.012268574 | Intracerebral hemorrhage | 0.00983953 |
| 2021 | High SDI | Gallbladder and biliary diseases | 0.012227729 | Lower extremity peripheral arterial disease | 0.004871975 |
| 2021 | High SDI | Chronic kidney disease due to other and unspecified causes | 0.012174537 | Gallbladder and biliary tract cancer | 0.004135952 |
| 2021 | High SDI | Uterine cancer | 0.011618775 | Chronic lymphoid leukemia | 0.001779604 |
| 2021 | High SDI | Uterine cancer | 0.011141157 | Kidney cancer | 0.010682967 |
| 2021 | High SDI | Intracerebral hemorrhage | 0.010796388 | Thyroid cancer | 0.001303407 |
| 2021 | High SDI | Chronic kidney disease due to other and unspecified causes | 0.010792422 | Burkitt lymphoma | 0.0000433 |
| 2021 | High SDI | Atrial fibrillation and flutter | 0.010404712 | Ischemic heart disease | 0.264686707 |
| 2021 | High SDI | Intracerebral hemorrhage | 0.008914203 | Total burden related to hepatitis C | 0.002082498 |
| 2021 | High SDI | Lower extremity peripheral arterial disease | 0.006569094 | Chronic kidney disease due to glomerulonephritis | 0.002468468 |
| 2021 | High SDI | Ovarian cancer | 0.00636201 | Ovarian cancer | 0.005072078 |
| 2021 | High SDI | Gallbladder and biliary tract cancer | 0.005933857 | Liver cancer due to hepatitis C | 0.00436304 |
| 2021 | High SDI | Lower extremity peripheral arterial disease | 0.0059255 | Total burden related to hepatitis C | 0.00436304 |
| 2021 | High SDI | Liver cancer due to hepatitis C | 0.005916508 | Chronic kidney disease due to glomerulonephritis | 0.004064299 |
| 2021 | High SDI | Total burden related to hepatitis C | 0.005916508 | Acute myeloid leukemia | 0.003973527 |
| 2021 | High SDI | Gallbladder and biliary tract cancer | 0.005874601 | Atrial fibrillation and flutter | 0.005900898 |
| 1990 | High SDI | Ischemic heart disease | 0.317944156 | Acute lymphoid leukemia | 0.001165554 |
| 2021 | High SDI | Ovarian cancer | 0.005822593 | Ovarian cancer | 0.006188628 |
| 2021 | High SDI | Liver cancer due to hepatitis C | 0.005749295 | Aortic aneurysm | 0.005199217 |
| 2021 | High SDI | Total burden related to hepatitis C | 0.005749295 | Liver cancer due to hepatitis B | 0.001524591 |
| 1990 | High SDI | Total Cancers excluding Non-melanoma skin cancer | 0.109872211 | Acute lymphoid leukemia | 0.00091687 |
| 1990 | High SDI | Total cancers | 0.109872211 | Acute myeloid leukemia | 0.00375708 |
| 1990 | High SDI | Diabetes mellitus type 2 | 0.105710939 | Gallbladder and biliary diseases | 0.027682132 |
| 2021 | High SDI | Acute myeloid leukemia | 0.005293081 | Chronic kidney disease due to hypertension | 0.015057831 |
| 2021 | High SDI | Other non-Hodgkin lymphoma | 0.00504502 | Diabetes mellitus type 2 | 0.164897356 |
| 1990 | High SDI | Hypertensive heart disease | 0.070167614 | Liver cancer due to hepatitis C | 0.002082498 |
| 2021 | High SDI | Chronic kidney disease due to glomerulonephritis | 0.00496869 | Other non-Hodgkin lymphoma | 0.0045057 |
| 2021 | High SDI | Acute myeloid leukemia | 0.004944166 | Aortic aneurysm | 0.003016553 |
| 2021 | High SDI | Asthma | 0.004899832 | Chronic kidney disease due to other and unspecified causes | 0.00671131 |
| 1990 | High SDI | Ischemic stroke | 0.039988984 | Asthma | 0.052343875 |
| 1990 | High SDI | Colon and rectum cancer | 0.035463147 | Ischemic stroke | 0.033312014 |
| 2021 | High SDI | Chronic kidney disease due to glomerulonephritis | 0.004884303 | Lower extremity peripheral arterial disease | 0.00401097 |
| 1990 | High SDI | Alzheimer's disease and other dementias | 0.032000153 | Uterine cancer | 0.008155846 |
| 2021 | High SDI | Other non-Hodgkin lymphoma | 0.00488291 | Colon and rectum cancer | 0.030686739 |
| 1990 | High SDI | Chronic kidney disease due to diabetes mellitus type 2 | 0.018572637 | Chronic kidney disease due to diabetes mellitus type 2 | 0.019929603 |
| 1990 | High SDI | Chronic kidney disease due to hypertension | 0.016492863 | Other non-Hodgkin lymphoma | 0.003973982 |
| 1990 | High SDI | Breast cancer | 0.016132382 | Liver cancer due to other causes | 0.000359201 |
| 2021 | High SDI | Liver cancer due to alcohol use | 0.004718462 | Multidrug-resistant tuberculosis without extensive drug resistance | 0.000100442 |
| 2021 | High SDI | Aortic aneurysm | 0.004627578 | Hypertensive heart disease | 0.054299083 |
| 1990 | High SDI | Asthma | 0.012717605 | Multiple myeloma | 0.002760824 |
| 2021 | High SDI | Aortic aneurysm | 0.004607894 | Other leukemia | 0.00068361 |
| 1990 | High SDI | Gallbladder and biliary diseases | 0.011676819 | Subarachnoid hemorrhage | 0.004962829 |
| 2021 | High SDI | Multiple myeloma | 0.004353745 | Breast cancer | 0.01363215 |
| 1990 | High SDI | Kidney cancer | 0.011053508 | Aortic aneurysm | 0.003174154 |
| 2021 | High SDI | Asthma | 0.004349547 | Acute myeloid leukemia | 0.004104618 |
| 2021 | High SDI | Liver cancer due to alcohol use | 0.004309577 | Asthma | 0.027219048 |
| 2021 | High SDI | Multiple myeloma | 0.004302733 | Liver cancer due to alcohol use | 0.00375402 |
| 1990 | High SDI | Intracerebral hemorrhage | 0.009251096 | Multiple myeloma | 0.003201389 |
| 1990 | High SDI | Uterine cancer | 0.008864822 | Total burden related to hepatitis B | 0.002743326 |
| 1990 | High SDI | Gallbladder and biliary tract cancer | 0.00691258 | Ischemic stroke | 0.022364331 |
| 1990 | High SDI | Aortic aneurysm | 0.006892381 | Alzheimer's disease and other dementias | 0.028290645 |
| 2021 | High SDI | Subarachnoid hemorrhage | 0.003625295 | Subarachnoid hemorrhage | 0.003776182 |
| 1990 | High SDI | Chronic kidney disease due to other and unspecified causes | 0.006291887 | Liver cancer due to hepatitis B | 0.00245591 |
| 1990 | High SDI | Ovarian cancer | 0.006251868 | Total burden related to hepatitis B | 0.00245591 |
| 2021 | High SDI | Liver cancer due to hepatitis B | 0.002894098 | Chronic lymphoid leukemia | 0.00131012 |
| 2021 | High SDI | Total burden related to hepatitis B | 0.002894098 | Ovarian cancer | 0.005166049 |
| 2021 | High SDI | Subarachnoid hemorrhage | 0.002765495 | Liver cancer due to alcohol use | 0.003698784 |
| 2021 | High SDI | Liver cancer due to hepatitis B | 0.00239924 | Chronic kidney disease due to diabetes mellitus type 2 | 0.033968216 |
| 1990 | High SDI | Atrial fibrillation and flutter | 0.005686389 | Drug-susceptible tuberculosis | 0.001317869 |
| 2021 | High SDI | Total burden related to hepatitis B | 0.00239924 | Gallbladder and biliary diseases | 0.026116213 |
| 2021 | High SDI | Chronic lymphoid leukemia | 0.001942346 | Thyroid cancer | 0.001143368 |
| 1990 | High SDI | Lower extremity peripheral arterial disease | 0.005026923 | Lower extremity peripheral arterial disease | 0.004089634 |
| 2021 | High SDI | Chronic lymphoid leukemia | 0.001798741 | Other leukemia | 0.000796845 |
| 2021 | High SDI | Drug-susceptible tuberculosis | 0.001502334 | Atrial fibrillation and flutter | 0.009402699 |
| 2021 | High SDI | Drug-susceptible tuberculosis | 0.001354351 | Gallbladder and biliary tract cancer | 0.003810502 |
| 2021 | High SDI | Thyroid cancer | 0.001323545 | Chronic lymphoid leukemia | 0.001128663 |
| 1990 | High SDI | Other non-Hodgkin lymphoma | 0.004486784 | Kidney cancer | 0.010684717 |
| 2021 | High SDI | Thyroid cancer | 0.001287401 | Liver cancer due to other causes | 0.000768786 |
| 1990 | High SDI | Drug-susceptible tuberculosis | 0.003848789 | Liver cancer due to hepatitis B | 0.002743326 |
| 2021 | High SDI | Other leukemia | 0.001094934 | Thyroid cancer | 0.001157144 |
| 1990 | High SDI | Acute myeloid leukemia | 0.003580272 | Chronic kidney disease due to hypertension | 0.032358368 |
| 1990 | High SDI | Subarachnoid hemorrhage | 0.003311133 | Diabetes mellitus type 2 | 0.270516657 |
| 1990 | High SDI | Multiple myeloma | 0.003183391 | Total Cancers excluding Non-melanoma skin cancer | 0.096759271 |
| 2021 | High SDI | Other leukemia | 0.001085729 | Liver cancer due to hepatitis C | 0.004112701 |
| 2021 | High SDI | Liver cancer due to other causes | 0.000944464 | Other non-Hodgkin lymphoma | 0.0039326 |
| 1990 | High SDI | Chronic kidney disease due to glomerulonephritis | 0.002548961 | Burkitt lymphoma | 0.0000837 |
| 1990 | High SDI | Liver cancer due to hepatitis C | 0.002305055 | Chronic myeloid leukemia | 0.000485679 |
| 1990 | High SDI | Total burden related to hepatitis C | 0.002305055 | Chronic kidney disease due to other and unspecified causes | 0.008803857 |
| 1990 | High SDI | Chronic lymphoid leukemia | 0.002224724 | Asthma | 0.041094434 |
| 2021 | High SDI | Liver cancer due to other causes | 0.000826842 | Total burden related to hepatitis C | 0.004112701 |
| 1990 | High SDI | Liver cancer due to alcohol use | 0.001735764 | Uterine cancer | 0.009635193 |
| 1990 | High SDI | Chronic myeloid leukemia | 0.001705063 | Colon and rectum cancer | 0.026468405 |
| 2021 | High SDI | Acute lymphoid leukemia | 0.000806434 | Liver cancer due to other causes | 0.000818235 |
| 2021 | High SDI | Chronic myeloid leukemia | 0.000622542 | Multidrug-resistant tuberculosis without extensive drug resistance | 0.0000717 |
| 1990 | High SDI | Thyroid cancer | 0.001316859 | Drug-susceptible tuberculosis | 0.001528531 |
| 1990 | High SDI | Liver cancer due to hepatitis B | 0.001260977 | Chronic kidney disease due to glomerulonephritis | 0.004166778 |
| 1990 | High SDI | Total burden related to hepatitis B | 0.001260977 | Hypertensive heart disease | 0.064357842 |
| 2021 | High SDI | Acute lymphoid leukemia | 0.000619558 | Multiple myeloma | 0.002971556 |
| 2021 | High SDI | Chronic myeloid leukemia | 0.000595227 | Total cancers | 0.096759271 |
| 1990 | High SDI | Acute lymphoid leukemia | 0.000849123 | Ischemic heart disease | 0.140195351 |
| 2021 | High SDI | Burkitt lymphoma | 0.0000769 | Other leukemia | 0.000774891 |
| 1990 | High SDI | Other leukemia | 0.000763982 | Subarachnoid hemorrhage | 0.004683024 |
| 2021 | High SDI | Multidrug-resistant tuberculosis without extensive drug resistance | 0.0000666 | Intracerebral hemorrhage | 0.01141956 |
| 1990 | High SDI | Liver cancer due to other causes | 0.000339355 | Breast cancer | 0.010487381 |
| 1990 | High SDI | Multidrug-resistant tuberculosis without extensive drug resistance | 0.0000963 | Acute lymphoid leukemia | 0.00068895 |
| 2021 | High SDI | Burkitt lymphoma | 0.0000644 | Chronic myeloid leukemia | 0.000461025 |
| 2021 | High SDI | Multidrug-resistant tuberculosis without extensive drug resistance | 0.0000553 | Burkitt lymphoma | 0.0000725 |
| 1990 | High SDI | Burkitt lymphoma | 0.0000342 | Multidrug-resistant tuberculosis without extensive drug resistance | 0.0000585 |
| 1990 | High-middle SDI | Atrial fibrillation and flutter | 0.003277225 | Atrial fibrillation and flutter | 0.003720789 |
| 1990 | High-middle SDI | Gallbladder and biliary tract cancer | 0.005844856 | Gallbladder and biliary tract cancer | 0.005026883 |
| 1990 | High-middle SDI | Acute lymphoid leukemia | 0.001343881 | Acute lymphoid leukemia | 0.001906589 |
| 1990 | High-middle SDI | Ovarian cancer | 0.004315603 | Ovarian cancer | 0.004527131 |
| 1990 | High-middle SDI | Chronic lymphoid leukemia | 0.001390778 | Chronic lymphoid leukemia | 0.001268446 |
| 1990 | High-middle SDI | Kidney cancer | 0.007637752 | Kidney cancer | 0.007517681 |
| 1990 | High-middle SDI | Liver cancer due to alcohol use | 0.001609773 | Liver cancer due to alcohol use | 0.001531493 |
| 1990 | High-middle SDI | Aortic aneurysm | 0.002794134 | Aortic aneurysm | 0.00241277 |
| 1990 | High-middle SDI | Liver cancer due to hepatitis B | 0.002089414 | Liver cancer due to hepatitis B | 0.0025257 |
| 1990 | High-middle SDI | Thyroid cancer | 0.001281372 | Thyroid cancer | 0.001278531 |
| 1990 | High-middle SDI | Acute myeloid leukemia | 0.002175047 | Acute myeloid leukemia | 0.002582575 |
| 1990 | High-middle SDI | Gallbladder and biliary diseases | 0.012859927 | Gallbladder and biliary diseases | 0.028395278 |
| 1990 | High-middle SDI | Chronic kidney disease due to hypertension | 0.014106451 | Chronic kidney disease due to hypertension | 0.013861706 |
| 1990 | High-middle SDI | Diabetes mellitus type 2 | 0.093814231 | Diabetes mellitus type 2 | 0.157559954 |
| 1990 | High-middle SDI | Total Cancers excluding Non-melanoma skin cancer | 0.084702127 | Total Cancers excluding Non-melanoma skin cancer | 0.081324904 |
| 1990 | High-middle SDI | Liver cancer due to hepatitis C | 0.00204713 | Liver cancer due to hepatitis C | 0.001743802 |
| 1990 | High-middle SDI | Other non-Hodgkin lymphoma | 0.002187048 | Other non-Hodgkin lymphoma | 0.002336943 |
| 1990 | High-middle SDI | Burkitt lymphoma | 0.0000209 | Burkitt lymphoma | 0.0000274 |
| 1990 | High-middle SDI | Chronic myeloid leukemia | 0.001023318 | Chronic myeloid leukemia | 0.001104585 |
| 1990 | High-middle SDI | Chronic kidney disease due to other and unspecified causes | 0.006383777 | Chronic kidney disease due to other and unspecified causes | 0.006749819 |
| 1990 | High-middle SDI | Asthma | 0.01325162 | Asthma | 0.03098987 |
| 1990 | High-middle SDI | Total burden related to hepatitis C | 0.00204713 | Total burden related to hepatitis C | 0.001743802 |
| 1990 | High-middle SDI | Total burden related to hepatitis B | 0.002089414 | Total burden related to hepatitis B | 0.0025257 |
| 1990 | High-middle SDI | Ischemic stroke | 0.082542247 | Ischemic stroke | 0.068678417 |
| 1990 | High-middle SDI | Lower extremity peripheral arterial disease | 0.004419273 | Lower extremity peripheral arterial disease | 0.003410175 |
| 1990 | High-middle SDI | Uterine cancer | 0.010440557 | Uterine cancer | 0.010455163 |
| 1990 | High-middle SDI | Colon and rectum cancer | 0.026311783 | Colon and rectum cancer | 0.023970905 |
| 1990 | High-middle SDI | Chronic kidney disease due to diabetes mellitus type 2 | 0.016926156 | Chronic kidney disease due to diabetes mellitus type 2 | 0.018734492 |
| 1990 | High-middle SDI | Alzheimer's disease and other dementias | 0.017855325 | Alzheimer's disease and other dementias | 0.013802481 |
| 1990 | High-middle SDI | Liver cancer due to other causes | 0.000250171 | Liver cancer due to other causes | 0.000271914 |
| 1990 | High-middle SDI | Multidrug-resistant tuberculosis without extensive drug resistance | 0.000253392 | Multidrug-resistant tuberculosis without extensive drug resistance | 0.000284028 |
| 1990 | High-middle SDI | Drug-susceptible tuberculosis | 0.008511913 | Drug-susceptible tuberculosis | 0.010287665 |
| 1990 | High-middle SDI | Chronic kidney disease due to glomerulonephritis | 0.003052499 | Chronic kidney disease due to glomerulonephritis | 0.003184634 |
| 1990 | High-middle SDI | Hypertensive heart disease | 0.107549011 | Hypertensive heart disease | 0.08334746 |
| 1990 | High-middle SDI | Multiple myeloma | 0.001359923 | Multiple myeloma | 0.001242269 |
| 1990 | High-middle SDI | Total cancers | 0.084702127 | Total cancers | 0.081324904 |
| 1990 | High-middle SDI | Ischemic heart disease | 0.33116115 | Ischemic heart disease | 0.280303295 |
| 1990 | High-middle SDI | Other leukemia | 0.001305904 | Other leukemia | 0.001446763 |
| 1990 | High-middle SDI | Subarachnoid hemorrhage | 0.003181428 | Subarachnoid hemorrhage | 0.004301232 |
| 1990 | High-middle SDI | Intracerebral hemorrhage | 0.020596181 | Intracerebral hemorrhage | 0.022400382 |
| 1990 | High-middle SDI | Breast cancer | 0.01128804 | Breast cancer | 0.009891501 |
| 2021 | High-middle SDI | Ischemic heart disease | 0.274405601 | Acute lymphoid leukemia | 0.001721679 |
| 2021 | High-middle SDI | Ischemic heart disease | 0.273977472 | Ovarian cancer | 0.004309319 |
| 2021 | High-middle SDI | Hypertensive heart disease | 0.121571895 | Colon and rectum cancer | 0.023469185 |
| 2021 | High-middle SDI | Hypertensive heart disease | 0.118541249 | Multidrug-resistant tuberculosis without extensive drug resistance | 0.000270632 |
| 2021 | High-middle SDI | Diabetes mellitus type 2 | 0.106672227 | Chronic kidney disease due to glomerulonephritis | 0.003176395 |
| 2021 | High-middle SDI | Diabetes mellitus type 2 | 0.105134283 | Ischemic heart disease | 0.215842108 |
| 2021 | High-middle SDI | Total Cancers excluding Non-melanoma skin cancer | 0.093661222 | Hypertensive heart disease | 0.075843096 |
| 2021 | High-middle SDI | Total cancers | 0.093661222 | Diabetes mellitus type 2 | 0.232696977 |
| 2021 | High-middle SDI | Total Cancers excluding Non-melanoma skin cancer | 0.091988678 | Total Cancers excluding Non-melanoma skin cancer | 0.085697297 |
| 2021 | High-middle SDI | Total cancers | 0.091988678 | Total cancers | 0.085697297 |
| 2021 | High-middle SDI | Ischemic stroke | 0.058697551 | Ischemic stroke | 0.050742427 |
| 2021 | High-middle SDI | Ischemic stroke | 0.058192389 | Chronic kidney disease due to hypertension | 0.014707546 |
| 2021 | High-middle SDI | Alzheimer's disease and other dementias | 0.037482447 | Other leukemia | 0.00136765 |
| 2021 | High-middle SDI | Alzheimer's disease and other dementias | 0.035749864 | Alzheimer's disease and other dementias | 0.025478768 |
| 2021 | High-middle SDI | Colon and rectum cancer | 0.029454583 | Colon and rectum cancer | 0.02540824 |
| 2021 | High-middle SDI | Colon and rectum cancer | 0.029022534 | Chronic kidney disease due to diabetes mellitus type 2 | 0.025065625 |
| 2021 | High-middle SDI | Chronic kidney disease due to diabetes mellitus type 2 | 0.027148411 | Chronic kidney disease due to hypertension | 0.020378076 |
| 2021 | High-middle SDI | Chronic kidney disease due to diabetes mellitus type 2 | 0.02713711 | Intracerebral hemorrhage | 0.022713198 |
| 2021 | High-middle SDI | Chronic kidney disease due to hypertension | 0.025977233 | Breast cancer | 0.010274067 |
| 2021 | High-middle SDI | Chronic kidney disease due to hypertension | 0.025369036 | Gallbladder and biliary diseases | 0.02453761 |
| 2021 | High-middle SDI | Intracerebral hemorrhage | 0.020432286 | Chronic kidney disease due to other and unspecified causes | 0.008323342 |
| 2021 | High-middle SDI | Intracerebral hemorrhage | 0.020426999 | Kidney cancer | 0.008370753 |
| 2021 | High-middle SDI | Breast cancer | 0.01158118 | Uterine cancer | 0.008917197 |
| 2021 | High-middle SDI | Breast cancer | 0.011049279 | Atrial fibrillation and flutter | 0.006016691 |
| 2021 | High-middle SDI | Gallbladder and biliary diseases | 0.010298389 | Gallbladder and biliary tract cancer | 0.004098386 |
| 2021 | High-middle SDI | Gallbladder and biliary diseases | 0.010061907 | Asthma | 0.0129474 |
| 2021 | High-middle SDI | Chronic kidney disease due to other and unspecified causes | 0.009920274 | Ovarian cancer | 0.004562058 |
| 2021 | High-middle SDI | Chronic kidney disease due to other and unspecified causes | 0.0095845 | Burkitt lymphoma | 0.0000253 |
| 2021 | High-middle SDI | Kidney cancer | 0.009169617 | Asthma | 0.029781138 |
| 2021 | High-middle SDI | Uterine cancer | 0.009126414 | Ischemic stroke | 0.070419999 |
| 2021 | High-middle SDI | Kidney cancer | 0.008975902 | Chronic myeloid leukemia | 0.001053026 |
| 2021 | High-middle SDI | Uterine cancer | 0.008875767 | Total burden related to hepatitis B | 0.002383741 |
| 2021 | High-middle SDI | Atrial fibrillation and flutter | 0.006740454 | Multiple myeloma | 0.001204537 |
| 2021 | High-middle SDI | Atrial fibrillation and flutter | 0.006440851 | Total Cancers excluding Non-melanoma skin cancer | 0.084104256 |
| 2021 | High-middle SDI | Asthma | 0.00500437 | Acute myeloid leukemia | 0.002401906 |
| 2021 | High-middle SDI | Gallbladder and biliary tract cancer | 0.004993242 | Gallbladder and biliary diseases | 0.028112145 |
| 2021 | High-middle SDI | Asthma | 0.00490777 | Other non-Hodgkin lymphoma | 0.002223288 |
| 2021 | High-middle SDI | Gallbladder and biliary tract cancer | 0.004889759 | Chronic kidney disease due to other and unspecified causes | 0.007543111 |
| 2021 | High-middle SDI | Ovarian cancer | 0.004552192 | Total cancers | 0.078315932 |
| 2021 | High-middle SDI | Ovarian cancer | 0.004459292 | Liver cancer due to hepatitis C | 0.002554873 |
| 2021 | High-middle SDI | Liver cancer due to hepatitis B | 0.004274407 | Liver cancer due to hepatitis B | 0.004959982 |
| 1990 | High-middle SDI | Ischemic heart disease | 0.336950931 | Total burden related to hepatitis B | 0.004959982 |
| 2021 | High-middle SDI | Total burden related to hepatitis B | 0.004274407 | Lower extremity peripheral arterial disease | 0.003138689 |
| 2021 | High-middle SDI | Liver cancer due to hepatitis B | 0.004221148 | Atrial fibrillation and flutter | 0.004256414 |
| 1990 | High-middle SDI | Hypertensive heart disease | 0.114459433 | Gallbladder and biliary tract cancer | 0.004955 |
| 2021 | High-middle SDI | Total burden related to hepatitis B | 0.004221148 | Chronic lymphoid leukemia | 0.001248044 |
| 2021 | High-middle SDI | Lower extremity peripheral arterial disease | 0.003876272 | Kidney cancer | 0.007213282 |
| 2021 | High-middle SDI | Lower extremity peripheral arterial disease | 0.003822218 | Liver cancer due to alcohol use | 0.001458784 |
| 1990 | High-middle SDI | Diabetes mellitus type 2 | 0.09039828 | Aortic aneurysm | 0.002375618 |
| 1990 | High-middle SDI | Ischemic stroke | 0.085280465 | Liver cancer due to hepatitis B | 0.002383741 |
| 1990 | High-middle SDI | Total Cancers excluding Non-melanoma skin cancer | 0.07805494 | Thyroid cancer | 0.001241094 |
| 1990 | High-middle SDI | Total cancers | 0.07805494 | Diabetes mellitus type 2 | 0.153304424 |
| 2021 | High-middle SDI | Chronic kidney disease due to glomerulonephritis | 0.003641691 | Total Cancers excluding Non-melanoma skin cancer | 0.078315932 |
| 2021 | High-middle SDI | Chronic kidney disease due to glomerulonephritis | 0.00361004 | Liver cancer due to hepatitis C | 0.001711156 |
| 2021 | High-middle SDI | Liver cancer due to hepatitis C | 0.003305898 | Total burden related to hepatitis C | 0.001711156 |
| 2021 | High-middle SDI | Total burden related to hepatitis C | 0.003305898 | Chronic kidney disease due to diabetes mellitus type 2 | 0.024424983 |
| 2021 | High-middle SDI | Liver cancer due to hepatitis C | 0.003230349 | Lower extremity peripheral arterial disease | 0.003788949 |
| 1990 | High-middle SDI | Colon and rectum cancer | 0.02482329 | Uterine cancer | 0.010036253 |
| 1990 | High-middle SDI | Alzheimer's disease and other dementias | 0.024659203 | Chronic kidney disease due to diabetes mellitus type 2 | 0.019251858 |
| 2021 | High-middle SDI | Total burden related to hepatitis C | 0.003230349 | Alzheimer's disease and other dementias | 0.017694503 |
| 1990 | High-middle SDI | Intracerebral hemorrhage | 0.018225215 | Liver cancer due to other causes | 0.000258336 |
| 1990 | High-middle SDI | Chronic kidney disease due to diabetes mellitus type 2 | 0.017645321 | Drug-susceptible tuberculosis | 0.00980151 |
| 1990 | High-middle SDI | Chronic kidney disease due to hypertension | 0.01564045 | Hypertensive heart disease | 0.087428002 |
| 1990 | High-middle SDI | Gallbladder and biliary diseases | 0.013586739 | Alzheimer's disease and other dementias | 0.025480343 |
| 1990 | High-middle SDI | Asthma | 0.012852234 | Ischemic heart disease | 0.284337435 |
| 2021 | High-middle SDI | Subarachnoid hemorrhage | 0.003124921 | Chronic kidney disease due to glomerulonephritis | 0.003193257 |
| 1990 | High-middle SDI | Breast cancer | 0.010377298 | Subarachnoid hemorrhage | 0.004069428 |
| 2021 | High-middle SDI | Aortic aneurysm | 0.003058225 | Intracerebral hemorrhage | 0.021273129 |
| 2021 | High-middle SDI | Subarachnoid hemorrhage | 0.003030764 | Breast cancer | 0.0093994 |
| 1990 | High-middle SDI | Uterine cancer | 0.009479175 | Liver cancer due to hepatitis C | 0.002653418 |
| 2021 | High-middle SDI | Aortic aneurysm | 0.003017671 | Total burden related to hepatitis C | 0.002653418 |
| 2021 | High-middle SDI | Drug-susceptible tuberculosis | 0.002742428 | Acute lymphoid leukemia | 0.001430534 |
| 1990 | High-middle SDI | Drug-susceptible tuberculosis | 0.007656302 | Chronic lymphoid leukemia | 0.001063231 |
| 1990 | High-middle SDI | Chronic kidney disease due to other and unspecified causes | 0.007310163 | Kidney cancer | 0.008188072 |
| 1990 | High-middle SDI | Kidney cancer | 0.006924465 | Aortic aneurysm | 0.002558535 |
| 2021 | High-middle SDI | Drug-susceptible tuberculosis | 0.002657662 | Subarachnoid hemorrhage | 0.004053926 |
| 1990 | High-middle SDI | Gallbladder and biliary tract cancer | 0.005544051 | Atrial fibrillation and flutter | 0.00593062 |
| 1990 | High-middle SDI | Lower extremity peripheral arterial disease | 0.005098413 | Drug-susceptible tuberculosis | 0.003840348 |
| 2021 | High-middle SDI | Other non-Hodgkin lymphoma | 0.002583928 | Drug-susceptible tuberculosis | 0.003602801 |
| 2021 | High-middle SDI | Other non-Hodgkin lymphoma | 0.002578282 | Liver cancer due to alcohol use | 0.002012675 |
| 2021 | High-middle SDI | Acute myeloid leukemia | 0.002503464 | Liver cancer due to hepatitis B | 0.005145318 |
| 1990 | High-middle SDI | Atrial fibrillation and flutter | 0.004302455 | Acute myeloid leukemia | 0.002616452 |
| 2021 | High-middle SDI | Acute myeloid leukemia | 0.002445933 | Other non-Hodgkin lymphoma | 0.002535865 |
| 2021 | High-middle SDI | Liver cancer due to alcohol use | 0.002249424 | Chronic kidney disease due to hypertension | 0.020506041 |
| 2021 | High-middle SDI | Liver cancer due to alcohol use | 0.002169865 | Acute myeloid leukemia | 0.002441239 |
| 1990 | High-middle SDI | Ovarian cancer | 0.003827305 | Liver cancer due to alcohol use | 0.002088777 |
| 2021 | High-middle SDI | Multiple myeloma | 0.00198775 | Uterine cancer | 0.008636368 |
| 2021 | High-middle SDI | Multiple myeloma | 0.001939914 | Chronic myeloid leukemia | 0.00037958 |
| 2021 | High-middle SDI | Chronic lymphoid leukemia | 0.001263913 | Multiple myeloma | 0.001713569 |
| 2021 | High-middle SDI | Chronic lymphoid leukemia | 0.001262881 | Total cancers | 0.084104256 |
| 1990 | High-middle SDI | Chronic kidney disease due to glomerulonephritis | 0.003034932 | Other leukemia | 0.001084635 |
| 2021 | High-middle SDI | Acute lymphoid leukemia | 0.001081391 | Chronic lymphoid leukemia | 0.001064844 |
| 1990 | High-middle SDI | Subarachnoid hemorrhage | 0.002787418 | Gallbladder and biliary tract cancer | 0.003974035 |
| 2021 | High-middle SDI | Other leukemia | 0.001068094 | Ovarian cancer | 0.004498859 |
| 1990 | High-middle SDI | Aortic aneurysm | 0.00266345 | Aortic aneurysm | 0.002521377 |
| 2021 | High-middle SDI | Thyroid cancer | 0.001066958 | Thyroid cancer | 0.001035159 |
| 2021 | High-middle SDI | Thyroid cancer | 0.001058263 | Thyroid cancer | 0.001032595 |
| 2021 | High-middle SDI | Other leukemia | 0.001044794 | Gallbladder and biliary diseases | 0.025463406 |
| 1990 | High-middle SDI | Other non-Hodgkin lymphoma | 0.001986923 | Diabetes mellitus type 2 | 0.235570669 |
| 2021 | High-middle SDI | Acute lymphoid leukemia | 0.000996207 | Other non-Hodgkin lymphoma | 0.002597736 |
| 1990 | High-middle SDI | Liver cancer due to hepatitis C | 0.001926884 | Burkitt lymphoma | 0.0000418 |
| 1990 | High-middle SDI | Total burden related to hepatitis C | 0.001926884 | Chronic kidney disease due to other and unspecified causes | 0.008448897 |
| 1990 | High-middle SDI | Acute myeloid leukemia | 0.001918918 | Asthma | 0.016145796 |
| 1990 | High-middle SDI | Liver cancer due to hepatitis B | 0.001816252 | Total burden related to hepatitis C | 0.002554873 |
| 1990 | High-middle SDI | Total burden related to hepatitis B | 0.001816252 | Total burden related to hepatitis B | 0.005145318 |
| 1990 | High-middle SDI | Liver cancer due to alcohol use | 0.00142759 | Ischemic stroke | 0.049652103 |
| 1990 | High-middle SDI | Chronic lymphoid leukemia | 0.001340493 | Lower extremity peripheral arterial disease | 0.003042606 |
| 2021 | High-middle SDI | Multidrug-resistant tuberculosis without extensive drug resistance | 0.000480738 | Other leukemia | 0.001019925 |
| 1990 | High-middle SDI | Multiple myeloma | 0.001253119 | Colon and rectum cancer | 0.024936938 |
| 1990 | High-middle SDI | Thyroid cancer | 0.001199084 | Liver cancer due to other causes | 0.000488868 |
| 1990 | High-middle SDI | Other leukemia | 0.001190934 | Multidrug-resistant tuberculosis without extensive drug resistance | 0.000658043 |
| 1990 | High-middle SDI | Acute lymphoid leukemia | 0.001146443 | Chronic kidney disease due to glomerulonephritis | 0.003221619 |
| 2021 | High-middle SDI | Multidrug-resistant tuberculosis without extensive drug resistance | 0.000464032 | Hypertensive heart disease | 0.075613491 |
| 2021 | High-middle SDI | Liver cancer due to other causes | 0.00046047 | Multiple myeloma | 0.001661795 |
| 2021 | High-middle SDI | Liver cancer due to other causes | 0.000458623 | Ischemic heart disease | 0.214302073 |
| 1990 | High-middle SDI | Chronic myeloid leukemia | 0.000945054 | Acute lymphoid leukemia | 0.001222321 |
| 2021 | High-middle SDI | Chronic myeloid leukemia | 0.000405495 | Subarachnoid hemorrhage | 0.004324548 |
| 2021 | High-middle SDI | Chronic myeloid leukemia | 0.000396729 | Intracerebral hemorrhage | 0.023197538 |
| 2021 | High-middle SDI | Burkitt lymphoma | 0.0000325 | Breast cancer | 0.00939982 |
| 1990 | High-middle SDI | Multidrug-resistant tuberculosis without extensive drug resistance | 0.000227338 | Multidrug-resistant tuberculosis without extensive drug resistance | 0.000616734 |
| 1990 | High-middle SDI | Liver cancer due to other causes | 0.000222594 | Liver cancer due to other causes | 0.000482072 |
| 2021 | High-middle SDI | Burkitt lymphoma | 0.0000307 | Chronic myeloid leukemia | 0.000359894 |
| 1990 | High-middle SDI | Burkitt lymphoma | 0.0000184 | Burkitt lymphoma | 0.0000375 |
| 1990 | Middle SDI | Atrial fibrillation and flutter | 0.00127554 | Atrial fibrillation and flutter | 0.001468338 |
| 1990 | Middle SDI | Gallbladder and biliary tract cancer | 0.004739906 | Gallbladder and biliary tract cancer | 0.003807542 |
| 1990 | Middle SDI | Acute lymphoid leukemia | 0.002014172 | Acute lymphoid leukemia | 0.002628561 |
| 1990 | Middle SDI | Ovarian cancer | 0.001585837 | Ovarian cancer | 0.001562868 |
| 1990 | Middle SDI | Chronic lymphoid leukemia | 0.000813983 | Chronic lymphoid leukemia | 0.000766713 |
| 1990 | Middle SDI | Kidney cancer | 0.002876264 | Kidney cancer | 0.002578631 |
| 1990 | Middle SDI | Liver cancer due to alcohol use | 0.000989014 | Liver cancer due to alcohol use | 0.000870716 |
| 1990 | Middle SDI | Aortic aneurysm | 0.001240995 | Aortic aneurysm | 0.00099174 |
| 1990 | Middle SDI | Liver cancer due to hepatitis B | 0.00307372 | Liver cancer due to hepatitis B | 0.003284432 |
| 1990 | Middle SDI | Thyroid cancer | 0.00126954 | Thyroid cancer | 0.001156663 |
| 1990 | Middle SDI | Acute myeloid leukemia | 0.002184202 | Acute myeloid leukemia | 0.002498324 |
| 1990 | Middle SDI | Gallbladder and biliary diseases | 0.013902603 | Gallbladder and biliary diseases | 0.029237824 |
| 1990 | Middle SDI | Chronic kidney disease due to hypertension | 0.030765862 | Chronic kidney disease due to hypertension | 0.027219488 |
| 1990 | Middle SDI | Diabetes mellitus type 2 | 0.198587199 | Diabetes mellitus type 2 | 0.280544049 |
| 1990 | Middle SDI | Total Cancers excluding Non-melanoma skin cancer | 0.049686318 | Total Cancers excluding Non-melanoma skin cancer | 0.045642865 |
| 1990 | Middle SDI | Liver cancer due to hepatitis C | 0.001588124 | Liver cancer due to hepatitis C | 0.001230968 |
| 1990 | Middle SDI | Other non-Hodgkin lymphoma | 0.002093831 | Other non-Hodgkin lymphoma | 0.002122667 |
| 1990 | Middle SDI | Burkitt lymphoma | 2.00E-05 | Burkitt lymphoma | 0.0000237 |
| 1990 | Middle SDI | Chronic myeloid leukemia | 0.000909966 | Chronic myeloid leukemia | 0.001013307 |
| 1990 | Middle SDI | Chronic kidney disease due to other and unspecified causes | 0.003966394 | Chronic kidney disease due to other and unspecified causes | 0.004787557 |
| 1990 | Middle SDI | Asthma | 0.027725613 | Asthma | 0.040564339 |
| 1990 | Middle SDI | Total burden related to hepatitis C | 0.001588124 | Total burden related to hepatitis C | 0.001230968 |
| 1990 | Middle SDI | Total burden related to hepatitis B | 0.00307372 | Total burden related to hepatitis B | 0.003284432 |
| 1990 | Middle SDI | Ischemic stroke | 0.035198572 | Ischemic stroke | 0.030699878 |
| 1990 | Middle SDI | Lower extremity peripheral arterial disease | 0.000722283 | Lower extremity peripheral arterial disease | 0.000759016 |
| 1990 | Middle SDI | Uterine cancer | 0.005640951 | Uterine cancer | 0.00515542 |
| 1990 | Middle SDI | Colon and rectum cancer | 0.012385751 | Colon and rectum cancer | 0.010930944 |
| 1990 | Middle SDI | Chronic kidney disease due to diabetes mellitus type 2 | 0.035979475 | Chronic kidney disease due to diabetes mellitus type 2 | 0.031971779 |
| 1990 | Middle SDI | Alzheimer's disease and other dementias | 0.007194999 | Alzheimer's disease and other dementias | 0.005275047 |
| 1990 | Middle SDI | Liver cancer due to other causes | 0.000338652 | Liver cancer due to other causes | 0.000341732 |
| 1990 | Middle SDI | Multidrug-resistant tuberculosis without extensive drug resistance | 0.000620082 | Multidrug-resistant tuberculosis without extensive drug resistance | 0.000586955 |
| 1990 | Middle SDI | Drug-susceptible tuberculosis | 0.032721174 | Drug-susceptible tuberculosis | 0.034523611 |
| 1990 | Middle SDI | Chronic kidney disease due to glomerulonephritis | 0.003221224 | Chronic kidney disease due to glomerulonephritis | 0.003457509 |
| 1990 | Middle SDI | Hypertensive heart disease | 0.234376041 | Hypertensive heart disease | 0.17209803 |
| 1990 | Middle SDI | Multiple myeloma | 0.000658127 | Multiple myeloma | 0.000563848 |
| 1990 | Middle SDI | Total cancers | 0.049686318 | Total cancers | 0.045642865 |
| 1990 | Middle SDI | Ischemic heart disease | 0.205119319 | Ischemic heart disease | 0.178620943 |
| 1990 | Middle SDI | Other leukemia | 0.001472347 | Other leukemia | 0.001636068 |
| 1990 | Middle SDI | Subarachnoid hemorrhage | 0.00159027 | Subarachnoid hemorrhage | 0.002321227 |
| 1990 | Middle SDI | Intracerebral hemorrhage | 0.011354651 | Intracerebral hemorrhage | 0.012826291 |
| 1990 | Middle SDI | Breast cancer | 0.005748871 | Breast cancer | 0.004072099 |
| 2021 | Middle SDI | Ischemic heart disease | 0.214171665 | Aortic aneurysm | 0.001021298 |
| 2021 | Middle SDI | Ischemic heart disease | 0.214002139 | Diabetes mellitus type 2 | 0.271142604 |
| 2021 | Middle SDI | Diabetes mellitus type 2 | 0.206700491 | Burkitt lymphoma | 0.000019 |
| 2021 | Middle SDI | Diabetes mellitus type 2 | 0.200109433 | Uterine cancer | 0.004983819 |
| 2021 | Middle SDI | Hypertensive heart disease | 0.162397703 | Multidrug-resistant tuberculosis without extensive drug resistance | 0.000553187 |
| 2021 | Middle SDI | Hypertensive heart disease | 0.151869007 | Breast cancer | 0.004628169 |
| 2021 | Middle SDI | Total Cancers excluding Non-melanoma skin cancer | 0.062163625 | Ischemic heart disease | 0.173886859 |
| 2021 | Middle SDI | Total cancers | 0.062163625 | Diabetes mellitus type 2 | 0.330593383 |
| 2021 | Middle SDI | Total Cancers excluding Non-melanoma skin cancer | 0.058602795 | Hypertensive heart disease | 0.101911978 |
| 2021 | Middle SDI | Total cancers | 0.058602795 | Total Cancers excluding Non-melanoma skin cancer | 0.054373379 |
| 2021 | Middle SDI | Chronic kidney disease due to diabetes mellitus type 2 | 0.048216265 | Total cancers | 0.054373379 |
| 2021 | Middle SDI | Chronic kidney disease due to diabetes mellitus type 2 | 0.046852162 | Chronic kidney disease due to diabetes mellitus type 2 | 0.037794458 |
| 2021 | Middle SDI | Chronic kidney disease due to hypertension | 0.046561683 | Chronic kidney disease due to hypertension | 0.035553317 |
| 2021 | Middle SDI | Chronic kidney disease due to hypertension | 0.044457615 | Ischemic stroke | 0.032218981 |
| 2021 | Middle SDI | Ischemic stroke | 0.038318325 | Intracerebral hemorrhage | 0.024400569 |
| 2021 | Middle SDI | Ischemic stroke | 0.037713998 | Alzheimer's disease and other dementias | 0.01431701 |
| 2021 | Middle SDI | Alzheimer's disease and other dementias | 0.025999261 | Colon and rectum cancer | 0.013996584 |
| 2021 | Middle SDI | Intracerebral hemorrhage | 0.024368788 | Asthma | 0.019898173 |
| 2021 | Middle SDI | Intracerebral hemorrhage | 0.022095864 | Drug-susceptible tuberculosis | 0.012330026 |
| 2021 | Middle SDI | Alzheimer's disease and other dementias | 0.021075245 | Gallbladder and biliary diseases | 0.022855604 |
| 2021 | Middle SDI | Colon and rectum cancer | 0.016709654 | Breast cancer | 0.00643591 |
| 2021 | Middle SDI | Colon and rectum cancer | 0.016114095 | Chronic kidney disease due to other and unspecified causes | 0.007890136 |
| 2021 | Middle SDI | Asthma | 0.014708642 | Chronic kidney disease due to glomerulonephritis | 0.005600093 |
| 2021 | Middle SDI | Asthma | 0.014532037 | Kidney cancer | 0.002492358 |
| 2021 | Middle SDI | Gallbladder and biliary diseases | 0.011264009 | Uterine cancer | 0.004841013 |
| 2021 | Middle SDI | Drug-susceptible tuberculosis | 0.010726817 | Liver cancer due to hepatitis C | 0.001290565 |
| 2021 | Middle SDI | Gallbladder and biliary diseases | 0.01052197 | Liver cancer due to hepatitis B | 0.004802263 |
| 2021 | Middle SDI | Drug-susceptible tuberculosis | 0.009996593 | Total burden related to hepatitis B | 0.004802263 |
| 2021 | Middle SDI | Breast cancer | 0.00806148 | Asthma | 0.03571678 |
| 2021 | Middle SDI | Breast cancer | 0.007399414 | Kidney cancer | 0.003706792 |
| 2021 | Middle SDI | Chronic kidney disease due to other and unspecified causes | 0.007225685 | Chronic kidney disease due to glomerulonephritis | 0.003225067 |
| 2021 | Middle SDI | Chronic kidney disease due to other and unspecified causes | 0.007164232 | Drug-susceptible tuberculosis | 0.03133922 |
| 2021 | Middle SDI | Chronic kidney disease due to glomerulonephritis | 0.005579755 | Lower extremity peripheral arterial disease | 0.000963331 |
| 2021 | Middle SDI | Chronic kidney disease due to glomerulonephritis | 0.005382588 | Hypertensive heart disease | 0.190900164 |
| 2021 | Middle SDI | Uterine cancer | 0.005323449 | Gallbladder and biliary tract cancer | 0.00321806 |
| 2021 | Middle SDI | Uterine cancer | 0.004958369 | Ischemic heart disease | 0.177692769 |
| 2021 | Middle SDI | Liver cancer due to hepatitis B | 0.004722663 | Liver cancer due to alcohol use | 0.000842279 |
| 2021 | Middle SDI | Total burden related to hepatitis B | 0.004722663 | Gallbladder and biliary diseases | 0.027918755 |
| 2021 | Middle SDI | Atrial fibrillation and flutter | 0.004716276 | Intracerebral hemorrhage | 0.011170389 |
| 2021 | Middle SDI | Kidney cancer | 0.004284397 | Atrial fibrillation and flutter | 0.003614858 |
| 2021 | Middle SDI | Liver cancer due to hepatitis B | 0.004254487 | Subarachnoid hemorrhage | 0.004479394 |
| 1990 | Middle SDI | Hypertensive heart disease | 0.25787952 | Other non-Hodgkin lymphoma | 0.001876467 |
| 2021 | Middle SDI | Total burden related to hepatitis B | 0.004254487 | Ovarian cancer | 0.002900758 |
| 1990 | Middle SDI | Ischemic heart disease | 0.201600278 | Chronic myeloid leukemia | 0.000844559 |
| 2021 | Middle SDI | Gallbladder and biliary tract cancer | 0.004164057 | Liver cancer due to hepatitis C | 0.002021087 |
| 1990 | Middle SDI | Diabetes mellitus type 2 | 0.18761653 | Total burden related to hepatitis C | 0.002021087 |
| 2021 | Middle SDI | Kidney cancer | 0.004052972 | Chronic kidney disease due to other and unspecified causes | 0.005231139 |
| 2021 | Middle SDI | Gallbladder and biliary tract cancer | 0.004021573 | Total burden related to hepatitis C | 0.001290565 |
| 2021 | Middle SDI | Atrial fibrillation and flutter | 0.003786821 | Alzheimer's disease and other dementias | 0.008117171 |
| 2021 | Middle SDI | Subarachnoid hemorrhage | 0.003724867 | Other non-Hodgkin lymphoma | 0.002113839 |
| 2021 | Middle SDI | Subarachnoid hemorrhage | 0.003354609 | Acute myeloid leukemia | 0.001969046 |
| 1990 | Middle SDI | Total Cancers excluding Non-melanoma skin cancer | 0.044375263 | Atrial fibrillation and flutter | 0.001949478 |
| 1990 | Middle SDI | Total cancers | 0.044375263 | Gallbladder and biliary tract cancer | 0.003905032 |
| 1990 | Middle SDI | Chronic kidney disease due to diabetes mellitus type 2 | 0.038926745 | Acute lymphoid leukemia | 0.001949425 |
| 2021 | Middle SDI | Ovarian cancer | 0.002957501 | Ovarian cancer | 0.001444116 |
| 1990 | Middle SDI | Ischemic stroke | 0.036688054 | Chronic lymphoid leukemia | 0.000714288 |
| 1990 | Middle SDI | Chronic kidney disease due to hypertension | 0.034633595 | Liver cancer due to alcohol use | 0.001600984 |
| 1990 | Middle SDI | Asthma | 0.028280497 | Liver cancer due to hepatitis B | 0.002868317 |
| 1990 | Middle SDI | Drug-susceptible tuberculosis | 0.027941422 | Thyroid cancer | 0.001116134 |
| 2021 | Middle SDI | Liver cancer due to hepatitis C | 0.002737445 | Acute myeloid leukemia | 0.002028986 |
| 2021 | Middle SDI | Total burden related to hepatitis C | 0.002737445 | Chronic kidney disease due to hypertension | 0.029276611 |
| 2021 | Middle SDI | Ovarian cancer | 0.002681466 | Total Cancers excluding Non-melanoma skin cancer | 0.043315755 |
| 1990 | Middle SDI | Gallbladder and biliary diseases | 0.015164162 | Total burden related to hepatitis B | 0.002868317 |
| 2021 | Middle SDI | Liver cancer due to hepatitis C | 0.002674491 | Ischemic stroke | 0.032312663 |
| 1990 | Middle SDI | Alzheimer's disease and other dementias | 0.011804284 | Colon and rectum cancer | 0.010683294 |
| 1990 | Middle SDI | Colon and rectum cancer | 0.011507498 | Chronic kidney disease due to diabetes mellitus type 2 | 0.034892782 |
| 2021 | Middle SDI | Total burden related to hepatitis C | 0.002674491 | Liver cancer due to other causes | 0.000306366 |
| 2021 | Middle SDI | Other non-Hodgkin lymphoma | 0.002232564 | Aortic aneurysm | 0.001200889 |
| 1990 | Middle SDI | Intracerebral hemorrhage | 0.008453151 | Multiple myeloma | 0.000558646 |
| 2021 | Middle SDI | Other non-Hodgkin lymphoma | 0.002105431 | Total cancers | 0.043315755 |
| 2021 | Middle SDI | Acute myeloid leukemia | 0.001935754 | Thyroid cancer | 0.001136188 |
| 1990 | Middle SDI | Breast cancer | 0.005669671 | Other leukemia | 0.001362046 |
| 2021 | Middle SDI | Liver cancer due to alcohol use | 0.001876268 | Subarachnoid hemorrhage | 0.001872288 |
| 1990 | Middle SDI | Uterine cancer | 0.005004367 | Multiple myeloma | 0.001041776 |
| 2021 | Middle SDI | Acute myeloid leukemia | 0.001829789 | Acute lymphoid leukemia | 0.001453163 |
| 2021 | Middle SDI | Liver cancer due to alcohol use | 0.001720862 | Multidrug-resistant tuberculosis without extensive drug resistance | 0.001205683 |
| 1990 | Middle SDI | Gallbladder and biliary tract cancer | 0.004545074 | Lower extremity peripheral arterial disease | 0.001075828 |
| 1990 | Middle SDI | Chronic kidney disease due to other and unspecified causes | 0.004396474 | Atrial fibrillation and flutter | 0.004059897 |
| 2021 | Middle SDI | Aortic aneurysm | 0.001583995 | Other leukemia | 0.000882908 |
| 2021 | Middle SDI | Aortic aneurysm | 0.001575131 | Chronic lymphoid leukemia | 0.000558314 |
| 2021 | Middle SDI | Thyroid cancer | 0.001272866 | Ovarian cancer | 0.002769235 |
| 2021 | Middle SDI | Multiple myeloma | 0.001244325 | Liver cancer due to other causes | 0.000480791 |
| 2021 | Middle SDI | Thyroid cancer | 0.001232107 | Acute lymphoid leukemia | 0.001471436 |
| 1990 | Middle SDI | Chronic kidney disease due to glomerulonephritis | 0.003065388 | Gallbladder and biliary tract cancer | 0.003177219 |
| 2021 | Middle SDI | Acute lymphoid leukemia | 0.001229463 | Alzheimer's disease and other dementias | 0.017036758 |
| 2021 | Middle SDI | Multiple myeloma | 0.00116785 | Chronic myeloid leukemia | 0.000416108 |
| 2021 | Middle SDI | Acute lymphoid leukemia | 0.00115555 | Liver cancer due to other causes | 0.000463322 |
| 1990 | Middle SDI | Kidney cancer | 0.002596254 | Diabetes mellitus type 2 | 0.323913821 |
| 1990 | Middle SDI | Liver cancer due to hepatitis B | 0.002389756 | Acute myeloid leukemia | 0.001966033 |
| 1990 | Middle SDI | Total burden related to hepatitis B | 0.002389756 | Liver cancer due to alcohol use | 0.001537367 |
| 2021 | Middle SDI | Lower extremity peripheral arterial disease | 0.00113306 | Chronic lymphoid leukemia | 0.000562517 |
| 1990 | Middle SDI | Atrial fibrillation and flutter | 0.002066018 | Kidney cancer | 0.003615447 |
| 2021 | Middle SDI | Multidrug-resistant tuberculosis without extensive drug resistance | 0.001092206 | Burkitt lymphoma | 0.0000271 |
| 1990 | Middle SDI | Other non-Hodgkin lymphoma | 0.001754936 | Aortic aneurysm | 0.001207851 |
| 2021 | Middle SDI | Multidrug-resistant tuberculosis without extensive drug resistance | 0.001014794 | Liver cancer due to hepatitis B | 0.004585661 |
| 1990 | Middle SDI | Acute myeloid leukemia | 0.00169882 | Thyroid cancer | 0.001123169 |
| 2021 | Middle SDI | Lower extremity peripheral arterial disease | 0.001012748 | Multiple myeloma | 0.001013531 |
| 1990 | Middle SDI | Liver cancer due to hepatitis C | 0.001555588 | Gallbladder and biliary diseases | 0.023053586 |
| 1990 | Middle SDI | Total burden related to hepatitis C | 0.001555588 | Chronic kidney disease due to hypertension | 0.036644635 |
| 1990 | Middle SDI | Acute lymphoid leukemia | 0.001407547 | Total burden related to hepatitis C | 0.002009068 |
| 1990 | Middle SDI | Ovarian cancer | 0.001325409 | Total Cancers excluding Non-melanoma skin cancer | 0.052890069 |
| 1990 | Middle SDI | Aortic aneurysm | 0.001245569 | Liver cancer due to hepatitis C | 0.002009068 |
| 2021 | Middle SDI | Other leukemia | 0.000905548 | Other non-Hodgkin lymphoma | 0.002081351 |
| 1990 | Middle SDI | Other leukemia | 0.001181403 | Burkitt lymphoma | 0.0000271 |
| 1990 | Middle SDI | Thyroid cancer | 0.001179684 | Chronic myeloid leukemia | 0.000419182 |
| 2021 | Middle SDI | Other leukemia | 0.000863398 | Chronic kidney disease due to other and unspecified causes | 0.008149729 |
| 2021 | Middle SDI | Chronic lymphoid leukemia | 0.000656634 | Asthma | 0.021056758 |
| 2021 | Middle SDI | Chronic lymphoid leukemia | 0.000651301 | Total burden related to hepatitis B | 0.004585661 |
| 2021 | Middle SDI | Liver cancer due to other causes | 0.000511565 | Ischemic stroke | 0.032674164 |
| 1990 | Middle SDI | Subarachnoid hemorrhage | 0.000987662 | Lower extremity peripheral arterial disease | 0.001152724 |
| 1990 | Middle SDI | Lower extremity peripheral arterial disease | 0.000954468 | Uterine cancer | 0.00467137 |
| 2021 | Middle SDI | Liver cancer due to other causes | 0.000470255 | Colon and rectum cancer | 0.013801976 |
| 1990 | Middle SDI | Liver cancer due to alcohol use | 0.000855373 | Chronic kidney disease due to diabetes mellitus type 2 | 0.03848893 |
| 1990 | Middle SDI | Chronic lymphoid leukemia | 0.000750977 | Multidrug-resistant tuberculosis without extensive drug resistance | 0.001179295 |
| 1990 | Middle SDI | Chronic myeloid leukemia | 0.000743515 | Drug-susceptible tuberculosis | 0.012075037 |
| 2021 | Middle SDI | Chronic myeloid leukemia | 0.000428021 | Chronic kidney disease due to glomerulonephritis | 0.005561121 |
| 1990 | Middle SDI | Multiple myeloma | 0.0005962 | Hypertensive heart disease | 0.107289817 |
| 1990 | Middle SDI | Multidrug-resistant tuberculosis without extensive drug resistance | 0.000545948 | Total cancers | 0.052890069 |
| 2021 | Middle SDI | Chronic myeloid leukemia | 0.000416798 | Ischemic heart disease | 0.174283916 |
| 2021 | Middle SDI | Burkitt lymphoma | 0.0000246 | Other leukemia | 0.000881242 |
| 1990 | Middle SDI | Liver cancer due to other causes | 0.000277094 | Subarachnoid hemorrhage | 0.004310651 |
| 2021 | Middle SDI | Burkitt lymphoma | 0.0000232 | Intracerebral hemorrhage | 0.023333166 |
| 1990 | Middle SDI | Burkitt lymphoma | 1.52E-05 | Breast cancer | 0.00597708 |
| 1990 | Low-middle SDI | Atrial fibrillation and flutter | 0.001027454 | Atrial fibrillation and flutter | 0.001118746 |
| 1990 | Low-middle SDI | Gallbladder and biliary tract cancer | 0.00306491 | Gallbladder and biliary tract cancer | 0.002590254 |
| 1990 | Low-middle SDI | Acute lymphoid leukemia | 0.000965103 | Acute lymphoid leukemia | 0.001307732 |
| 1990 | Low-middle SDI | Ovarian cancer | 0.000975416 | Ovarian cancer | 0.001002012 |
| 1990 | Low-middle SDI | Chronic lymphoid leukemia | 0.00031471 | Chronic lymphoid leukemia | 0.000260434 |
| 1990 | Low-middle SDI | Kidney cancer | 0.001470107 | Kidney cancer | 0.001348906 |
| 1990 | Low-middle SDI | Liver cancer due to alcohol use | 0.000903321 | Liver cancer due to alcohol use | 0.000795739 |
| 1990 | Low-middle SDI | Aortic aneurysm | 0.00078027 | Aortic aneurysm | 0.000622999 |
| 1990 | Low-middle SDI | Liver cancer due to hepatitis B | 0.00174601 | Liver cancer due to hepatitis B | 0.001826187 |
| 1990 | Low-middle SDI | Thyroid cancer | 0.001028846 | Thyroid cancer | 0.001022264 |
| 1990 | Low-middle SDI | Acute myeloid leukemia | 0.001547504 | Acute myeloid leukemia | 0.001790156 |
| 1990 | Low-middle SDI | Gallbladder and biliary diseases | 0.00960387 | Gallbladder and biliary diseases | 0.018238354 |
| 1990 | Low-middle SDI | Chronic kidney disease due to hypertension | 0.031923191 | Chronic kidney disease due to hypertension | 0.028748677 |
| 1990 | Low-middle SDI | Diabetes mellitus type 2 | 0.196516927 | Diabetes mellitus type 2 | 0.256069863 |
| 1990 | Low-middle SDI | Total Cancers excluding Non-melanoma skin cancer | 0.033273885 | Total Cancers excluding Non-melanoma skin cancer | 0.03053211 |
| 1990 | Low-middle SDI | Liver cancer due to hepatitis C | 0.003542383 | Liver cancer due to hepatitis C | 0.002937469 |
| 1990 | Low-middle SDI | Other non-Hodgkin lymphoma | 0.0015848 | Other non-Hodgkin lymphoma | 0.001628062 |
| 1990 | Low-middle SDI | Burkitt lymphoma | 0.0000177 | Burkitt lymphoma | 0.0000228 |
| 1990 | Low-middle SDI | Chronic myeloid leukemia | 0.000754668 | Chronic myeloid leukemia | 0.000859129 |
| 1990 | Low-middle SDI | Chronic kidney disease due to other and unspecified causes | 0.005126584 | Chronic kidney disease due to other and unspecified causes | 0.005666169 |
| 1990 | Low-middle SDI | Asthma | 0.051352942 | Asthma | 0.063332695 |
| 1990 | Low-middle SDI | Total burden related to hepatitis C | 0.003542383 | Total burden related to hepatitis C | 0.002937469 |
| 1990 | Low-middle SDI | Total burden related to hepatitis B | 0.00174601 | Total burden related to hepatitis B | 0.001826187 |
| 1990 | Low-middle SDI | Ischemic stroke | 0.035124629 | Ischemic stroke | 0.030304764 |
| 1990 | Low-middle SDI | Lower extremity peripheral arterial disease | 0.000333098 | Lower extremity peripheral arterial disease | 0.00041841 |
| 1990 | Low-middle SDI | Uterine cancer | 0.003859363 | Uterine cancer | 0.003542674 |
| 1990 | Low-middle SDI | Colon and rectum cancer | 0.006490412 | Colon and rectum cancer | 0.005993235 |
| 1990 | Low-middle SDI | Chronic kidney disease due to diabetes mellitus type 2 | 0.029767183 | Chronic kidney disease due to diabetes mellitus type 2 | 0.027222793 |
| 1990 | Low-middle SDI | Alzheimer's disease and other dementias | 0.006000063 | Alzheimer's disease and other dementias | 0.004452274 |
| 1990 | Low-middle SDI | Liver cancer due to other causes | 0.000390849 | Liver cancer due to other causes | 0.000390697 |
| 1990 | Low-middle SDI | Multidrug-resistant tuberculosis without extensive drug resistance | 0.000164845 | Multidrug-resistant tuberculosis without extensive drug resistance | 0.000166183 |
| 1990 | Low-middle SDI | Drug-susceptible tuberculosis | 0.067472357 | Drug-susceptible tuberculosis | 0.069598348 |
| 1990 | Low-middle SDI | Chronic kidney disease due to glomerulonephritis | 0.002683594 | Chronic kidney disease due to glomerulonephritis | 0.002753119 |
| 1990 | Low-middle SDI | Hypertensive heart disease | 0.215948375 | Hypertensive heart disease | 0.169779853 |
| 1990 | Low-middle SDI | Multiple myeloma | 0.000438974 | Multiple myeloma | 0.00038069 |
| 1990 | Low-middle SDI | Total cancers | 0.033273885 | Total cancers | 0.03053211 |
| 1990 | Low-middle SDI | Ischemic heart disease | 0.225448268 | Ischemic heart disease | 0.208164139 |
| 1990 | Low-middle SDI | Other leukemia | 0.000926217 | Other leukemia | 0.000980343 |
| 1990 | Low-middle SDI | Subarachnoid hemorrhage | 0.001625944 | Subarachnoid hemorrhage | 0.00207077 |
| 1990 | Low-middle SDI | Intracerebral hemorrhage | 0.013748206 | Intracerebral hemorrhage | 0.014717883 |
| 1990 | Low-middle SDI | Breast cancer | 0.003494715 | Breast cancer | 0.002047331 |
| 2021 | Low-middle SDI | Atrial fibrillation and flutter | 0.00240661 | Gallbladder and biliary diseases | 0.017276719 |
| 2021 | Low-middle SDI | Gallbladder and biliary tract cancer | 0.003235883 | Total burden related to hepatitis B | 0.001649703 |
| 2021 | Low-middle SDI | Acute lymphoid leukemia | 0.00076918 | Alzheimer's disease and other dementias | 0.006845529 |
| 2021 | Low-middle SDI | Ovarian cancer | 0.002392156 | Colon and rectum cancer | 0.005872027 |
| 2021 | Low-middle SDI | Chronic lymphoid leukemia | 0.000348536 | Ischemic heart disease | 0.203494731 |
| 2021 | Low-middle SDI | Kidney cancer | 0.002257423 | Breast cancer | 0.002782573 |
| 2021 | Low-middle SDI | Liver cancer due to alcohol use | 0.001440008 | Thyroid cancer | 0.000955612 |
| 2021 | Low-middle SDI | Aortic aneurysm | 0.001183261 | Acute myeloid leukemia | 0.001482398 |
| 2021 | Low-middle SDI | Liver cancer due to hepatitis B | 0.002175929 | Other non-Hodgkin lymphoma | 0.001466521 |
| 2021 | Low-middle SDI | Thyroid cancer | 0.001055577 | Asthma | 0.056240094 |
| 2021 | Low-middle SDI | Acute myeloid leukemia | 0.001613574 | Multidrug-resistant tuberculosis without extensive drug resistance | 0.000154899 |
| 2021 | Low-middle SDI | Gallbladder and biliary diseases | 0.007006462 | Drug-susceptible tuberculosis | 0.065052696 |
| 2021 | Low-middle SDI | Chronic kidney disease due to hypertension | 0.042685953 | Hypertensive heart disease | 0.185560184 |
| 2021 | Low-middle SDI | Diabetes mellitus type 2 | 0.235353364 | Atrial fibrillation and flutter | 0.002290583 |
| 2021 | Low-middle SDI | Total Cancers excluding Non-melanoma skin cancer | 0.043607857 | Gallbladder and biliary tract cancer | 0.002574395 |
| 2021 | Low-middle SDI | Liver cancer due to hepatitis C | 0.004369179 | Acute lymphoid leukemia | 0.000956996 |
| 2021 | Low-middle SDI | Other non-Hodgkin lymphoma | 0.001800644 | Ovarian cancer | 0.002339557 |
| 2021 | Low-middle SDI | Burkitt lymphoma | 0.0000257 | Chronic lymphoid leukemia | 0.000272379 |
| 2021 | Low-middle SDI | Chronic myeloid leukemia | 0.000434562 | Kidney cancer | 0.001947798 |
| 2021 | Low-middle SDI | Chronic kidney disease due to other and unspecified causes | 0.010705253 | Liver cancer due to alcohol use | 0.001229381 |
| 2021 | Low-middle SDI | Asthma | 0.033166505 | Aortic aneurysm | 0.000894631 |
| 2021 | Low-middle SDI | Total burden related to hepatitis C | 0.004369179 | Liver cancer due to hepatitis B | 0.002195896 |
| 2021 | Low-middle SDI | Total burden related to hepatitis B | 0.002175929 | Thyroid cancer | 0.00099463 |
| 2021 | Low-middle SDI | Ischemic stroke | 0.034269496 | Acute myeloid leukemia | 0.001693575 |
| 2021 | Low-middle SDI | Lower extremity peripheral arterial disease | 0.000688814 | Gallbladder and biliary diseases | 0.012215269 |
| 2021 | Low-middle SDI | Uterine cancer | 0.004286956 | Chronic kidney disease due to hypertension | 0.035611095 |
| 2021 | Low-middle SDI | Colon and rectum cancer | 0.008631552 | Diabetes mellitus type 2 | 0.338268722 |
| 2021 | Low-middle SDI | Chronic kidney disease due to diabetes mellitus type 2 | 0.034303887 | Total Cancers excluding Non-melanoma skin cancer | 0.03749044 |
| 2021 | Low-middle SDI | Alzheimer's disease and other dementias | 0.012047661 | Liver cancer due to hepatitis C | 0.003494883 |
| 2021 | Low-middle SDI | Liver cancer due to other causes | 0.00050264 | Other non-Hodgkin lymphoma | 0.001719076 |
| 2021 | Low-middle SDI | Multidrug-resistant tuberculosis without extensive drug resistance | 0.003307758 | Burkitt lymphoma | 0.0000305 |
| 2021 | Low-middle SDI | Drug-susceptible tuberculosis | 0.028329457 | Chronic myeloid leukemia | 0.000427303 |
| 2021 | Low-middle SDI | Chronic kidney disease due to glomerulonephritis | 0.00599213 | Chronic kidney disease due to other and unspecified causes | 0.010759699 |
| 2021 | Low-middle SDI | Hypertensive heart disease | 0.153614828 | Asthma | 0.035340415 |
| 2021 | Low-middle SDI | Multiple myeloma | 0.000825698 | Total burden related to hepatitis C | 0.003494883 |
| 2021 | Low-middle SDI | Total cancers | 0.043607857 | Total burden related to hepatitis B | 0.002195896 |
| 2021 | Low-middle SDI | Ischemic heart disease | 0.234214259 | Ischemic stroke | 0.028872283 |
| 2021 | Low-middle SDI | Other leukemia | 0.000743735 | Lower extremity peripheral arterial disease | 0.000669504 |
| 2021 | Low-middle SDI | Subarachnoid hemorrhage | 0.002985145 | Uterine cancer | 0.003768899 |
| 2021 | Low-middle SDI | Intracerebral hemorrhage | 0.020977192 | Colon and rectum cancer | 0.007452193 |
| 2021 | Low-middle SDI | Breast cancer | 0.00609219 | Chronic kidney disease due to diabetes mellitus type 2 | 0.029195513 |
| 1990 | Low-middle SDI | Hypertensive heart disease | 0.237221505 | Alzheimer's disease and other dementias | 0.007969091 |
| 2021 | Low-middle SDI | Diabetes mellitus type 2 | 0.23382517 | Liver cancer due to other causes | 0.000480991 |
| 2021 | Low-middle SDI | Ischemic heart disease | 0.226222717 | Multidrug-resistant tuberculosis without extensive drug resistance | 0.003413973 |
| 1990 | Low-middle SDI | Ischemic heart disease | 0.216560316 | Drug-susceptible tuberculosis | 0.030304836 |
| 1990 | Low-middle SDI | Diabetes mellitus type 2 | 0.18903684 | Chronic kidney disease due to glomerulonephritis | 0.005780852 |
| 2021 | Low-middle SDI | Hypertensive heart disease | 0.164717573 | Hypertensive heart disease | 0.112116493 |
| 1990 | Low-middle SDI | Drug-susceptible tuberculosis | 0.060119086 | Multiple myeloma | 0.000683225 |
| 1990 | Low-middle SDI | Asthma | 0.050526403 | Total cancers | 0.03749044 |
| 2021 | Low-middle SDI | Chronic kidney disease due to hypertension | 0.045315142 | Ischemic heart disease | 0.203474729 |
| 2021 | Low-middle SDI | Total Cancers excluding Non-melanoma skin cancer | 0.04130281 | Other leukemia | 0.000706314 |
| 2021 | Low-middle SDI | Total cancers | 0.04130281 | Subarachnoid hemorrhage | 0.003705471 |
| 1990 | Low-middle SDI | Ischemic stroke | 0.037607466 | Intracerebral hemorrhage | 0.021468672 |
| 2021 | Low-middle SDI | Chronic kidney disease due to diabetes mellitus type 2 | 0.035487875 | Breast cancer | 0.004008476 |
| 2021 | Low-middle SDI | Ischemic stroke | 0.035412277 | Liver cancer due to other causes | 0.000360972 |
| 1990 | Low-middle SDI | Chronic kidney disease due to hypertension | 0.035035307 | Intracerebral hemorrhage | 0.01331456 |
| 2021 | Low-middle SDI | Asthma | 0.033649524 | Kidney cancer | 0.001323662 |
| 1990 | Low-middle SDI | Chronic kidney disease due to diabetes mellitus type 2 | 0.03216611 | Liver cancer due to hepatitis B | 0.001649703 |
| 1990 | Low-middle SDI | Total Cancers excluding Non-melanoma skin cancer | 0.030855411 | Ischemic stroke | 0.032529433 |
| 1990 | Low-middle SDI | Total cancers | 0.030855411 | Subarachnoid hemorrhage | 0.001775369 |
| 2021 | Low-middle SDI | Drug-susceptible tuberculosis | 0.025542044 | Atrial fibrillation and flutter | 0.001509325 |
| 2021 | Low-middle SDI | Intracerebral hemorrhage | 0.018246838 | Gallbladder and biliary tract cancer | 0.002665832 |
| 2021 | Low-middle SDI | Alzheimer's disease and other dementias | 0.017020175 | Acute lymphoid leukemia | 0.000968157 |
| 1990 | Low-middle SDI | Intracerebral hemorrhage | 0.011243817 | Ovarian cancer | 0.000926569 |
| 2021 | Low-middle SDI | Chronic kidney disease due to other and unspecified causes | 0.011020123 | Chronic lymphoid leukemia | 0.000273719 |
| 1990 | Low-middle SDI | Gallbladder and biliary diseases | 0.010324914 | Liver cancer due to alcohol use | 0.000794441 |
| 1990 | Low-middle SDI | Alzheimer's disease and other dementias | 0.009760317 | Aortic aneurysm | 0.00066039 |
| 2021 | Low-middle SDI | Colon and rectum cancer | 0.008280927 | Chronic kidney disease due to hypertension | 0.030703599 |
| 2021 | Low-middle SDI | Gallbladder and biliary diseases | 0.007522702 | Diabetes mellitus type 2 | 0.250792442 |
| 2021 | Low-middle SDI | Breast cancer | 0.006164137 | Total Cancers excluding Non-melanoma skin cancer | 0.029877553 |
| 1990 | Low-middle SDI | Colon and rectum cancer | 0.006105501 | Liver cancer due to hepatitis C | 0.003050601 |
| 2021 | Low-middle SDI | Chronic kidney disease due to glomerulonephritis | 0.005811782 | Burkitt lymphoma | 0.0000178 |
| 1990 | Low-middle SDI | Chronic kidney disease due to other and unspecified causes | 0.005774895 | Chronic myeloid leukemia | 0.000722203 |
| 2021 | Low-middle SDI | Liver cancer due to hepatitis C | 0.004184029 | Chronic kidney disease due to other and unspecified causes | 0.006382963 |
| 2021 | Low-middle SDI | Total burden related to hepatitis C | 0.004184029 | Total burden related to hepatitis C | 0.003050601 |
| 2021 | Low-middle SDI | Uterine cancer | 0.004041023 | Lower extremity peripheral arterial disease | 0.000550144 |
| 1990 | Low-middle SDI | Breast cancer | 0.003744718 | Uterine cancer | 0.003512833 |
| 1990 | Low-middle SDI | Uterine cancer | 0.003577372 | Chronic kidney disease due to diabetes mellitus type 2 | 0.02994067 |
| 1990 | Low-middle SDI | Liver cancer due to hepatitis C | 0.003414678 | Chronic kidney disease due to glomerulonephritis | 0.00267784 |
| 1990 | Low-middle SDI | Total burden related to hepatitis C | 0.003414678 | Multiple myeloma | 0.000386205 |
| 2021 | Low-middle SDI | Atrial fibrillation and flutter | 0.003310346 | Total cancers | 0.029877553 |
| 2021 | Low-middle SDI | Gallbladder and biliary tract cancer | 0.003166207 | Other leukemia | 0.000871213 |
| 1990 | Low-middle SDI | Gallbladder and biliary tract cancer | 0.002997986 | Atrial fibrillation and flutter | 0.00283395 |
| 2021 | Low-middle SDI | Multidrug-resistant tuberculosis without extensive drug resistance | 0.002965563 | Burkitt lymphoma | 0.0000265 |
| 1990 | Low-middle SDI | Chronic kidney disease due to glomerulonephritis | 0.002679261 | Uterine cancer | 0.003732622 |
| 2021 | Low-middle SDI | Subarachnoid hemorrhage | 0.002467994 | Acute myeloid leukemia | 0.001527101 |
| 2021 | Low-middle SDI | Kidney cancer | 0.002142456 | Ovarian cancer | 0.002207027 |
| 2021 | Low-middle SDI | Ovarian cancer | 0.002124701 | Total Cancers excluding Non-melanoma skin cancer | 0.037161402 |
| 2021 | Low-middle SDI | Liver cancer due to hepatitis B | 0.00189046 | Alzheimer's disease and other dementias | 0.010824504 |
| 2021 | Low-middle SDI | Total burden related to hepatitis B | 0.00189046 | Chronic kidney disease due to other and unspecified causes | 0.011214867 |
| 2021 | Low-middle SDI | Other non-Hodgkin lymphoma | 0.00164975 | Liver cancer due to other causes | 0.000453447 |
| 1990 | Low-middle SDI | Atrial fibrillation and flutter | 0.001629429 | Asthma | 0.03443074 |
| 1990 | Low-middle SDI | Liver cancer due to hepatitis B | 0.001439967 | Total burden related to hepatitis C | 0.00354449 |
| 1990 | Low-middle SDI | Total burden related to hepatitis B | 0.001439967 | Chronic kidney disease due to diabetes mellitus type 2 | 0.030663458 |
| 2021 | Low-middle SDI | Acute myeloid leukemia | 0.001422252 | Drug-susceptible tuberculosis | 0.028136386 |
| 1990 | Low-middle SDI | Other non-Hodgkin lymphoma | 0.00138771 | Lower extremity peripheral arterial disease | 0.000790144 |
| 1990 | Low-middle SDI | Kidney cancer | 0.001363726 | Ischemic stroke | 0.029957881 |
| 2021 | Low-middle SDI | Liver cancer due to alcohol use | 0.001327205 | Chronic kidney disease due to glomerulonephritis | 0.005608033 |
| 1990 | Low-middle SDI | Acute myeloid leukemia | 0.00125199 | Total cancers | 0.037161402 |
| 1990 | Low-middle SDI | Subarachnoid hemorrhage | 0.001233504 | Hypertensive heart disease | 0.119568747 |
| 2021 | Low-middle SDI | Aortic aneurysm | 0.001204152 | Gallbladder and biliary tract cancer | 0.002616679 |
| 2021 | Low-middle SDI | Thyroid cancer | 0.000998363 | Acute lymphoid leukemia | 0.000807091 |
| 1990 | Low-middle SDI | Thyroid cancer | 0.000942802 | Chronic lymphoid leukemia | 0.000281897 |
| 2021 | Low-middle SDI | Lower extremity peripheral arterial disease | 0.000838432 | Kidney cancer | 0.001925628 |
| 1990 | Low-middle SDI | Ovarian cancer | 0.000826751 | Liver cancer due to alcohol use | 0.00121275 |
| 1990 | Low-middle SDI | Liver cancer due to alcohol use | 0.000819495 | Aortic aneurysm | 0.000923253 |
| 1990 | Low-middle SDI | Other leukemia | 0.000811282 | Liver cancer due to hepatitis B | 0.002033126 |
| 1990 | Low-middle SDI | Aortic aneurysm | 0.000807443 | Thyroid cancer | 0.000955347 |
| 2021 | Low-middle SDI | Multiple myeloma | 0.000788182 | Gallbladder and biliary diseases | 0.01188181 |
| 2021 | Low-middle SDI | Other leukemia | 0.000695909 | Chronic kidney disease due to hypertension | 0.03702455 |
| 1990 | Low-middle SDI | Acute lymphoid leukemia | 0.000693436 | Diabetes mellitus type 2 | 0.33306612 |
| 1990 | Low-middle SDI | Chronic myeloid leukemia | 0.000636947 | Liver cancer due to hepatitis C | 0.00354449 |
| 2021 | Low-middle SDI | Acute lymphoid leukemia | 0.00063011 | Other non-Hodgkin lymphoma | 0.001616928 |
| 1990 | Low-middle SDI | Lower extremity peripheral arterial disease | 0.000454713 | Chronic myeloid leukemia | 0.00039621 |
| 2021 | Low-middle SDI | Liver cancer due to other causes | 0.000447676 | Total burden related to hepatitis B | 0.002033126 |
| 1990 | Low-middle SDI | Multiple myeloma | 0.00041407 | Colon and rectum cancer | 0.007365447 |
| 2021 | Low-middle SDI | Chronic myeloid leukemia | 0.000406211 | Multidrug-resistant tuberculosis without extensive drug resistance | 0.003162244 |
| 2021 | Low-middle SDI | Chronic lymphoid leukemia | 0.0003583 | Multiple myeloma | 0.000685159 |
| 1990 | Low-middle SDI | Liver cancer due to other causes | 0.000335652 | Ischemic heart disease | 0.200121166 |
| 1990 | Low-middle SDI | Chronic lymphoid leukemia | 0.00032946 | Other leukemia | 0.000668178 |
| 1990 | Low-middle SDI | Multidrug-resistant tuberculosis without extensive drug resistance | 0.000146382 | Subarachnoid hemorrhage | 0.003286872 |
| 2021 | Low-middle SDI | Burkitt lymphoma | 0.0000216 | Intracerebral hemorrhage | 0.019953154 |
| 1990 | Low-middle SDI | Burkitt lymphoma | 0.0000133 | Breast cancer | 0.004596072 |
| 1990 | Low SDI | Atrial fibrillation and flutter | 0.000405932 | Atrial fibrillation and flutter | 0.000498789 |
| 1990 | Low SDI | Gallbladder and biliary tract cancer | 0.001440166 | Gallbladder and biliary tract cancer | 0.00125726 |
| 1990 | Low SDI | Acute lymphoid leukemia | 0.000925275 | Acute lymphoid leukemia | 0.001232218 |
| 1990 | Low SDI | Ovarian cancer | 0.000835765 | Ovarian cancer | 0.000858349 |
| 1990 | Low SDI | Chronic lymphoid leukemia | 0.000633056 | Chronic lymphoid leukemia | 0.00052564 |
| 1990 | Low SDI | Kidney cancer | 0.0011267 | Kidney cancer | 0.001011338 |
| 1990 | Low SDI | Liver cancer due to alcohol use | 0.000883053 | Liver cancer due to alcohol use | 0.000795574 |
| 1990 | Low SDI | Aortic aneurysm | 0.000867876 | Aortic aneurysm | 0.000685618 |
| 1990 | Low SDI | Liver cancer due to hepatitis B | 0.002621168 | Liver cancer due to hepatitis B | 0.002715049 |
| 1990 | Low SDI | Thyroid cancer | 0.001146728 | Thyroid cancer | 0.001200551 |
| 1990 | Low SDI | Acute myeloid leukemia | 0.000986243 | Acute myeloid leukemia | 0.001085735 |
| 1990 | Low SDI | Gallbladder and biliary diseases | 0.008934049 | Gallbladder and biliary diseases | 0.012328973 |
| 1990 | Low SDI | Chronic kidney disease due to hypertension | 0.034547926 | Chronic kidney disease due to hypertension | 0.03058324 |
| 1990 | Low SDI | Diabetes mellitus type 2 | 0.246067928 | Diabetes mellitus type 2 | 0.28910278 |
| 1990 | Low SDI | Total Cancers excluding Non-melanoma skin cancer | 0.028779287 | Total Cancers excluding Non-melanoma skin cancer | 0.026389134 |
| 1990 | Low SDI | Liver cancer due to hepatitis C | 0.001749372 | Liver cancer due to hepatitis C | 0.001468515 |
| 1990 | Low SDI | Other non-Hodgkin lymphoma | 0.001819459 | Other non-Hodgkin lymphoma | 0.00183635 |
| 1990 | Low SDI | Burkitt lymphoma | 0.0000595 | Burkitt lymphoma | 0.0000792 |
| 1990 | Low SDI | Chronic myeloid leukemia | 0.000460399 | Chronic myeloid leukemia | 0.000524452 |
| 1990 | Low SDI | Chronic kidney disease due to other and unspecified causes | 0.004526634 | Chronic kidney disease due to other and unspecified causes | 0.00521344 |
| 1990 | Low SDI | Asthma | 0.054089234 | Asthma | 0.073405979 |
| 1990 | Low SDI | Total burden related to hepatitis C | 0.001749372 | Total burden related to hepatitis C | 0.001468515 |
| 1990 | Low SDI | Total burden related to hepatitis B | 0.002621168 | Total burden related to hepatitis B | 0.002715049 |
| 1990 | Low SDI | Ischemic stroke | 0.020363708 | Ischemic stroke | 0.019124997 |
| 1990 | Low SDI | Lower extremity peripheral arterial disease | 0.000366499 | Lower extremity peripheral arterial disease | 0.000360788 |
| 1990 | Low SDI | Uterine cancer | 0.003541009 | Uterine cancer | 0.003252657 |
| 1990 | Low SDI | Colon and rectum cancer | 0.005921917 | Colon and rectum cancer | 0.005407281 |
| 1990 | Low SDI | Chronic kidney disease due to diabetes mellitus type 2 | 0.029697647 | Chronic kidney disease due to diabetes mellitus type 2 | 0.026947896 |
| 1990 | Low SDI | Alzheimer's disease and other dementias | 0.002368354 | Alzheimer's disease and other dementias | 0.001988394 |
| 1990 | Low SDI | Liver cancer due to other causes | 0.000356027 | Liver cancer due to other causes | 0.000358168 |
| 1990 | Low SDI | Multidrug-resistant tuberculosis without extensive drug resistance | 0.000433097 | Multidrug-resistant tuberculosis without extensive drug resistance | 0.000435584 |
| 1990 | Low SDI | Drug-susceptible tuberculosis | 0.092257046 | Drug-susceptible tuberculosis | 0.09487986 |
| 1990 | Low SDI | Chronic kidney disease due to glomerulonephritis | 0.005175117 | Chronic kidney disease due to glomerulonephritis | 0.004976894 |
| 1990 | Low SDI | Hypertensive heart disease | 0.265554108 | Hypertensive heart disease | 0.222108667 |
| 1990 | Low SDI | Multiple myeloma | 0.000322474 | Multiple myeloma | 0.00028228 |
| 1990 | Low SDI | Total cancers | 0.028779287 | Total cancers | 0.026389134 |
| 1990 | Low SDI | Ischemic heart disease | 0.131905911 | Ischemic heart disease | 0.12158825 |
| 1990 | Low SDI | Other leukemia | 0.000731867 | Other leukemia | 0.000759753 |
| 1990 | Low SDI | Subarachnoid hemorrhage | 0.000550965 | Subarachnoid hemorrhage | 0.000675417 |
| 1990 | Low SDI | Intracerebral hemorrhage | 0.010804016 | Intracerebral hemorrhage | 0.011437915 |
| 1990 | Low SDI | Breast cancer | 0.003594686 | Breast cancer | 0.002044336 |
| 2021 | Low SDI | Diabetes mellitus type 2 | 0.252847506 | Atrial fibrillation and flutter | 0.000633576 |
| 2021 | Low SDI | Diabetes mellitus type 2 | 0.247531552 | Gallbladder and biliary tract cancer | 0.001285907 |
| 2021 | Low SDI | Hypertensive heart disease | 0.24183972 | Chronic myeloid leukemia | 0.000440035 |
| 2021 | Low SDI | Hypertensive heart disease | 0.221321407 | Asthma | 0.060381204 |
| 2021 | Low SDI | Ischemic heart disease | 0.15564432 | Colon and rectum cancer | 0.005400433 |
| 2021 | Low SDI | Ischemic heart disease | 0.147354756 | Kidney cancer | 0.001016362 |
| 2021 | Low SDI | Drug-susceptible tuberculosis | 0.054873712 | Liver cancer due to alcohol use | 0.000791304 |
| 2021 | Low SDI | Drug-susceptible tuberculosis | 0.047448704 | Uterine cancer | 0.003247496 |
| 2021 | Low SDI | Chronic kidney disease due to hypertension | 0.04509405 | Alzheimer's disease and other dementias | 0.002816384 |
| 2021 | Low SDI | Chronic kidney disease due to hypertension | 0.040650706 | Total cancers | 0.026133038 |
| 2021 | Low SDI | Asthma | 0.039706751 | Ischemic heart disease | 0.120830819 |
| 2021 | Low SDI | Asthma | 0.038719733 | Other leukemia | 0.000688491 |
| 2021 | Low SDI | Total Cancers excluding Non-melanoma skin cancer | 0.036291399 | Subarachnoid hemorrhage | 0.000614278 |
| 2021 | Low SDI | Total cancers | 0.036291399 | Diabetes mellitus type 2 | 0.344233933 |
| 2021 | Low SDI | Total Cancers excluding Non-melanoma skin cancer | 0.034024029 | Hypertensive heart disease | 0.167416058 |
| 2021 | Low SDI | Total cancers | 0.034024029 | Ischemic heart disease | 0.134932393 |
| 2021 | Low SDI | Chronic kidney disease due to diabetes mellitus type 2 | 0.032087459 | Drug-susceptible tuberculosis | 0.056631852 |
| 2021 | Low SDI | Chronic kidney disease due to diabetes mellitus type 2 | 0.02967772 | Chronic kidney disease due to hypertension | 0.033958308 |
| 2021 | Low SDI | Ischemic stroke | 0.027230583 | Asthma | 0.051649931 |
| 2021 | Low SDI | Ischemic stroke | 0.025277915 | Total Cancers excluding Non-melanoma skin cancer | 0.030809022 |
| 2021 | Low SDI | Intracerebral hemorrhage | 0.025128636 | Total cancers | 0.030809022 |
| 2021 | Low SDI | Intracerebral hemorrhage | 0.020298727 | Chronic kidney disease due to diabetes mellitus type 2 | 0.025141699 |
| 2021 | Low SDI | Alzheimer's disease and other dementias | 0.010887925 | Ischemic stroke | 0.022324852 |
| 2021 | Low SDI | Gallbladder and biliary diseases | 0.009988533 | Intracerebral hemorrhage | 0.025038713 |
| 2021 | Low SDI | Gallbladder and biliary diseases | 0.008993034 | Gallbladder and biliary diseases | 0.011752573 |
| 2021 | Low SDI | Chronic kidney disease due to glomerulonephritis | 0.008383443 | Chronic kidney disease due to glomerulonephritis | 0.007769967 |
| 2021 | Low SDI | Chronic kidney disease due to glomerulonephritis | 0.008025709 | Gallbladder and biliary diseases | 0.012288654 |
| 2021 | Low SDI | Chronic kidney disease due to other and unspecified causes | 0.007370785 | Other non-Hodgkin lymphoma | 0.001683906 |
| 2021 | Low SDI | Colon and rectum cancer | 0.00707391 | Colon and rectum cancer | 0.005974943 |
| 2021 | Low SDI | Chronic kidney disease due to other and unspecified causes | 0.00694704 | Chronic kidney disease due to other and unspecified causes | 0.007599159 |
| 2021 | Low SDI | Colon and rectum cancer | 0.006779973 | Lower extremity peripheral arterial disease | 0.000474857 |
| 2021 | Low SDI | Alzheimer's disease and other dementias | 0.006683588 | Alzheimer's disease and other dementias | 0.004584677 |
| 2021 | Low SDI | Breast cancer | 0.005947336 | Multiple myeloma | 0.000288361 |
| 2021 | Low SDI | Breast cancer | 0.005076913 | Breast cancer | 0.002571939 |
| 2021 | Low SDI | Multidrug-resistant tuberculosis without extensive drug resistance | 0.004527724 | Multidrug-resistant tuberculosis without extensive drug resistance | 0.004466331 |
| 2021 | Low SDI | Uterine cancer | 0.004273979 | Uterine cancer | 0.003683897 |
| 2021 | Low SDI | Uterine cancer | 0.003940951 | Liver cancer due to hepatitis B | 0.003121941 |
| 2021 | Low SDI | Multidrug-resistant tuberculosis without extensive drug resistance | 0.003939732 | Total burden related to hepatitis B | 0.003121941 |
| 2021 | Low SDI | Liver cancer due to hepatitis B | 0.003084687 | Liver cancer due to hepatitis C | 0.001539256 |
| 2021 | Low SDI | Total burden related to hepatitis B | 0.003084687 | Chronic kidney disease due to other and unspecified causes | 0.005701307 |
| 2021 | Low SDI | Liver cancer due to hepatitis B | 0.0024554 | Liver cancer due to hepatitis C | 0.001711931 |
| 1990 | Low SDI | Hypertensive heart disease | 0.287548925 | Total burden related to hepatitis C | 0.001711931 |
| 2021 | Low SDI | Total burden related to hepatitis B | 0.0024554 | Other non-Hodgkin lymphoma | 0.00210764 |
| 2021 | Low SDI | Liver cancer due to hepatitis C | 0.002191361 | Subarachnoid hemorrhage | 0.002536667 |
| 1990 | Low SDI | Diabetes mellitus type 2 | 0.236082591 | Ovarian cancer | 0.001897718 |
| 2021 | Low SDI | Total burden related to hepatitis C | 0.002191361 | Gallbladder and biliary tract cancer | 0.001398396 |
| 1990 | Low SDI | Ischemic heart disease | 0.12619688 | Kidney cancer | 0.001458199 |
| 1990 | Low SDI | Drug-susceptible tuberculosis | 0.084192461 | Acute lymphoid leukemia | 0.000907499 |
| 1990 | Low SDI | Asthma | 0.052419214 | Ovarian cancer | 0.000798327 |
| 2021 | Low SDI | Other non-Hodgkin lymphoma | 0.002173002 | Chronic lymphoid leukemia | 0.000561622 |
| 2021 | Low SDI | Liver cancer due to hepatitis C | 0.002144662 | Aortic aneurysm | 0.000749605 |
| 1990 | Low SDI | Chronic kidney disease due to hypertension | 0.039121849 | Liver cancer due to hepatitis B | 0.002481398 |
| 2021 | Low SDI | Total burden related to hepatitis C | 0.002144662 | Thyroid cancer | 0.001081367 |
| 2021 | Low SDI | Subarachnoid hemorrhage | 0.002048918 | Acute myeloid leukemia | 0.000925361 |
| 2021 | Low SDI | Ovarian cancer | 0.001952093 | Chronic kidney disease due to hypertension | 0.033163896 |
| 2021 | Low SDI | Other non-Hodgkin lymphoma | 0.001875638 | Diabetes mellitus type 2 | 0.284602052 |
| 1990 | Low SDI | Chronic kidney disease due to diabetes mellitus type 2 | 0.031903265 | Total Cancers excluding Non-melanoma skin cancer | 0.026133038 |
| 2021 | Low SDI | Atrial fibrillation and flutter | 0.001798006 | Burkitt lymphoma | 0.0000595 |
| 1990 | Low SDI | Total Cancers excluding Non-melanoma skin cancer | 0.026867261 | Total burden related to hepatitis C | 0.001539256 |
| 1990 | Low SDI | Total cancers | 0.026867261 | Total burden related to hepatitis B | 0.002481398 |
| 1990 | Low SDI | Ischemic stroke | 0.022052536 | Ischemic stroke | 0.020544262 |
| 2021 | Low SDI | Gallbladder and biliary tract cancer | 0.001774487 | Chronic kidney disease due to diabetes mellitus type 2 | 0.02964111 |
| 2021 | Low SDI | Gallbladder and biliary tract cancer | 0.001733431 | Liver cancer due to other causes | 0.000331946 |
| 1990 | Low SDI | Gallbladder and biliary diseases | 0.010183603 | Multidrug-resistant tuberculosis without extensive drug resistance | 0.000412475 |
| 2021 | Low SDI | Kidney cancer | 0.001719337 | Drug-susceptible tuberculosis | 0.090060642 |
| 1990 | Low SDI | Intracerebral hemorrhage | 0.008533877 | Chronic kidney disease due to glomerulonephritis | 0.004872425 |
| 2021 | Low SDI | Ovarian cancer | 0.001612674 | Hypertensive heart disease | 0.238906765 |
| 2021 | Low SDI | Kidney cancer | 0.001602455 | Liver cancer due to alcohol use | 0.001074452 |
| 2021 | Low SDI | Subarachnoid hemorrhage | 0.00147662 | Intracerebral hemorrhage | 0.01055846 |
| 2021 | Low SDI | Liver cancer due to alcohol use | 0.001256486 | Breast cancer | 0.00293191 |
| 1990 | Low SDI | Colon and rectum cancer | 0.005618049 | Atrial fibrillation and flutter | 0.001218621 |
| 1990 | Low SDI | Chronic kidney disease due to glomerulonephritis | 0.005159415 | Gallbladder and biliary tract cancer | 0.001473402 |
| 1990 | Low SDI | Chronic kidney disease due to other and unspecified causes | 0.005049478 | Thyroid cancer | 0.001131727 |
| 1990 | Low SDI | Breast cancer | 0.004024713 | Aortic aneurysm | 0.000868575 |
| 2021 | Low SDI | Atrial fibrillation and flutter | 0.001194312 | Acute myeloid leukemia | 0.00116321 |
| 2021 | Low SDI | Aortic aneurysm | 0.001180557 | Ovarian cancer | 0.001746806 |
| 1990 | Low SDI | Alzheimer's disease and other dementias | 0.003796263 | Thyroid cancer | 0.00101968 |
| 1990 | Low SDI | Uterine cancer | 0.003280522 | Acute lymphoid leukemia | 0.001041127 |
| 2021 | Low SDI | Thyroid cancer | 0.001155653 | Lower extremity peripheral arterial disease | 0.000636008 |
| 2021 | Low SDI | Aortic aneurysm | 0.001145675 | Other non-Hodgkin lymphoma | 0.00187462 |
| 1990 | Low SDI | Liver cancer due to hepatitis B | 0.002196354 | Other leukemia | 0.000617222 |
| 1990 | Low SDI | Total burden related to hepatitis B | 0.002196354 | Chronic kidney disease due to glomerulonephritis | 0.007411207 |
| 2021 | Low SDI | Liver cancer due to alcohol use | 0.001123431 | Multidrug-resistant tuberculosis without extensive drug resistance | 0.004051953 |
| 2021 | Low SDI | Acute myeloid leukemia | 0.001076691 | Kidney cancer | 0.001468898 |
| 2021 | Low SDI | Thyroid cancer | 0.001025033 | Liver cancer due to alcohol use | 0.001071202 |
| 2021 | Low SDI | Lower extremity peripheral arterial disease | 0.001008862 | Chronic kidney disease due to diabetes mellitus type 2 | 0.028058454 |
| 2021 | Low SDI | Acute myeloid leukemia | 0.000846247 | Chronic lymphoid leukemia | 0.000455049 |
| 1990 | Low SDI | Liver cancer due to hepatitis C | 0.001711924 | Multiple myeloma | 0.000492236 |
| 1990 | Low SDI | Total burden related to hepatitis C | 0.001711924 | Total cancers | 0.031181878 |
| 1990 | Low SDI | Other non-Hodgkin lymphoma | 0.001625866 | Atrial fibrillation and flutter | 0.001634549 |
| 2021 | Low SDI | Acute lymphoid leukemia | 0.00079952 | Acute lymphoid leukemia | 0.00071217 |
| 2021 | Low SDI | Lower extremity peripheral arterial disease | 0.000788092 | Other leukemia | 0.000550881 |
| 2021 | Low SDI | Other leukemia | 0.00063624 | Chronic lymphoid leukemia | 0.000497015 |
| 1990 | Low SDI | Gallbladder and biliary tract cancer | 0.001390064 | Aortic aneurysm | 0.000929523 |
| 2021 | Low SDI | Chronic lymphoid leukemia | 0.000635232 | Liver cancer due to hepatitis B | 0.00274125 |
| 2021 | Low SDI | Chronic lymphoid leukemia | 0.000598599 | Ischemic heart disease | 0.133304995 |
| 1990 | Low SDI | Kidney cancer | 0.001062103 | Acute myeloid leukemia | 0.000936618 |
| 2021 | Low SDI | Multiple myeloma | 0.000597381 | Gallbladder and biliary diseases | 0.011464292 |
| 1990 | Low SDI | Thyroid cancer | 0.00101339 | Chronic kidney disease due to hypertension | 0.036308099 |
| 2021 | Low SDI | Multiple myeloma | 0.000561871 | Diabetes mellitus type 2 | 0.335436274 |
| 1990 | Low SDI | Aortic aneurysm | 0.000938563 | Total Cancers excluding Non-melanoma skin cancer | 0.031181878 |
| 2021 | Low SDI | Other leukemia | 0.000556366 | Liver cancer due to hepatitis C | 0.001826429 |
| 1990 | Low SDI | Acute myeloid leukemia | 0.000823954 | Burkitt lymphoma | 0.000068 |
| 1990 | Low SDI | Liver cancer due to alcohol use | 0.000800547 | Chronic myeloid leukemia | 0.000291995 |
| 1990 | Low SDI | Ovarian cancer | 0.000697584 | Chronic kidney disease due to other and unspecified causes | 0.00790199 |
| 1990 | Low SDI | Acute lymphoid leukemia | 0.000662858 | Asthma | 0.043019937 |
| 1990 | Low SDI | Chronic lymphoid leukemia | 0.000657196 | Total burden related to hepatitis C | 0.001826429 |
| 1990 | Low SDI | Other leukemia | 0.000647929 | Total burden related to hepatitis B | 0.00274125 |
| 2021 | Low SDI | Acute lymphoid leukemia | 0.000526722 | Ischemic stroke | 0.024114338 |
| 1990 | Low SDI | Atrial fibrillation and flutter | 0.000617808 | Lower extremity peripheral arterial disease | 0.000831626 |
| 2021 | Low SDI | Liver cancer due to other causes | 0.000448683 | Uterine cancer | 0.003728326 |
| 2021 | Low SDI | Liver cancer due to other causes | 0.00037265 | Colon and rectum cancer | 0.006030983 |
| 2021 | Low SDI | Chronic myeloid leukemia | 0.000336851 | Alzheimer's disease and other dementias | 0.007169479 |
| 1990 | Low SDI | Lower extremity peripheral arterial disease | 0.000495787 | Liver cancer due to other causes | 0.000392492 |
| 1990 | Low SDI | Subarachnoid hemorrhage | 0.000413667 | Intracerebral hemorrhage | 0.02267359 |
| 1990 | Low SDI | Multidrug-resistant tuberculosis without extensive drug resistance | 0.000394152 | Drug-susceptible tuberculosis | 0.051235357 |
| 1990 | Low SDI | Chronic myeloid leukemia | 0.000388854 | Hypertensive heart disease | 0.18427742 |
| 2021 | Low SDI | Chronic myeloid leukemia | 0.000279462 | Multiple myeloma | 0.000505573 |
| 1990 | Low SDI | Multiple myeloma | 0.00030683 | Liver cancer due to other causes | 0.000435559 |
| 1990 | Low SDI | Liver cancer due to other causes | 0.000305795 | Subarachnoid hemorrhage | 0.002067096 |
| 2021 | Low SDI | Burkitt lymphoma | 0.0000748 | Chronic myeloid leukemia | 0.000356531 |
| 2021 | Low SDI | Burkitt lymphoma | 0.0000503 | Breast cancer | 0.004242058 |
| 1990 | Low SDI | Burkitt lymphoma | 0.0000423 | Burkitt lymphoma | 0.0000941 |
|  | | ASMR | | ASDR | |
| year | location | cause | percent | cause | percent |
| 1990 | Global | Ischemic heart disease | 0.284785767 | Atrial fibrillation and flutter | 0.004133011 |
| 1990 | Global | Ischemic heart disease | 0.221581149 | Gallbladder and biliary tract cancer | 0.004565637 |
| 1990 | Global | Diabetes mellitus type 2 | 0.160059684 | Acute lymphoid leukemia | 0.001424769 |
| 1990 | Global | Hypertensive heart disease | 0.141094941 | Ovarian cancer | 0.003639119 |
| 1990 | Global | Hypertensive heart disease | 0.136401162 | Chronic lymphoid leukemia | 0.001208372 |
| 1990 | Global | Diabetes mellitus type 2 | 0.130740665 | Kidney cancer | 0.006203012 |
| 1990 | Global | Total Cancers excluding Non-melanoma skin cancer | 0.079027918 | Liver cancer due to alcohol use | 0.001270251 |
| 1990 | Global | Total cancers | 0.079027918 | Aortic aneurysm | 0.002828406 |
| 1990 | Global | Total Cancers excluding Non-melanoma skin cancer | 0.075659943 | Liver cancer due to hepatitis B | 0.002099575 |
| 1990 | Global | Total cancers | 0.075659943 | Thyroid cancer | 0.001188161 |
| 1990 | Global | Ischemic stroke | 0.053143541 | Acute myeloid leukemia | 0.002497826 |
| 1990 | Global | Chronic kidney disease due to hypertension | 0.041373543 | Gallbladder and biliary diseases | 0.025675858 |
| 1990 | Global | Chronic kidney disease due to diabetes mellitus type 2 | 0.039257947 | Chronic kidney disease due to hypertension | 0.020719647 |
| 1990 | Global | Ischemic stroke | 0.03893123 | Diabetes mellitus type 2 | 0.200135943 |
| 1990 | Global | Alzheimer's disease and other dementias | 0.033878023 | Total Cancers excluding Non-melanoma skin cancer | 0.069642819 |
| 1990 | Global | Alzheimer's disease and other dementias | 0.025192454 | Liver cancer due to hepatitis C | 0.001866559 |
| 1990 | Global | Chronic kidney disease due to diabetes mellitus type 2 | 0.023925769 | Other non-Hodgkin lymphoma | 0.002715038 |
| 1990 | Global | Colon and rectum cancer | 0.023539181 | Burkitt lymphoma | 0.00 |
| 1990 | Global | Chronic kidney disease due to hypertension | 0.022431157 | Chronic myeloid leukemia | 0.001139325 |
| 1990 | Global | Colon and rectum cancer | 0.022192989 | Chronic kidney disease due to other and unspecified causes | 0.00674174 |
| 1990 | Global | Asthma | 0.020726408 | Asthma | 0.040206947 |
| 1990 | Global | Drug-susceptible tuberculosis | 0.019135167 | Total burden related to hepatitis C | 0.001866559 |
| 1990 | Global | Intracerebral hemorrhage | 0.018491488 | Total burden related to hepatitis B | 0.002099575 |
| 1990 | Global | Asthma | 0.013948251 | Ischemic stroke | 0.044003021 |
| 1990 | Global | Gallbladder and biliary diseases | 0.013006791 | Lower extremity peripheral arterial disease | 0.002940914 |
| 1990 | Global | Intracerebral hemorrhage | 0.011904334 | Uterine cancer | 0.007308636 |
| 1990 | Global | Drug-susceptible tuberculosis | 0.011326581 | Colon and rectum cancer | 0.020318823 |
| 1990 | Global | Gallbladder and biliary diseases | 0.010891508 | Chronic kidney disease due to diabetes mellitus type 2 | 0.024525978 |
| 1990 | Global | Breast cancer | 0.010651839 | Alzheimer's disease and other dementias | 0.017018996 |
| 1990 | Global | Chronic kidney disease due to other and unspecified causes | 0.009876823 | Liver cancer due to other causes | 0.000311391 |
| 1990 | Global | Breast cancer | 0.009786035 | Multidrug-resistant tuberculosis without extensive drug resistance | 0.000270218 |
| 1990 | Global | Uterine cancer | 0.007574999 | Drug-susceptible tuberculosis | 0.023528272 |
| 1990 | Global | Uterine cancer | 0.007293344 | Chronic kidney disease due to glomerulonephritis | 0.003017551 |
| 1990 | Global | Kidney cancer | 0.007188366 | Hypertensive heart disease | 0.115153346 |
| 1990 | Global | Atrial fibrillation and flutter | 0.006600375 | Multiple myeloma | 0.001441914 |
| 1990 | Global | Kidney cancer | 0.006540756 | Total cancers | 0.069642819 |
| 1990 | Global | Chronic kidney disease due to other and unspecified causes | 0.006296378 | Ischemic heart disease | 0.239325654 |
| 1990 | Global | Gallbladder and biliary tract cancer | 0.005433618 | Other leukemia | 0.00106952 |
| 1990 | Global | Chronic kidney disease due to glomerulonephritis | 0.005161471 | Subarachnoid hemorrhage | 0.003188817 |
| 1990 | Global | Gallbladder and biliary tract cancer | 0.004483397 | Intracerebral hemorrhage | 0.014132161 |
| 1990 | Global | Atrial fibrillation and flutter | 0.004358104 | Breast cancer | 0.008904539 |
| 2021 | Global | Lower extremity peripheral arterial disease | 0.003919022 | Atrial fibrillation and flutter | 0.00563385 |
| 2021 | Global | Ovarian cancer | 0.003794881 | Gallbladder and biliary tract cancer | 0.003363955 |
| 2021 | Global | Liver cancer due to hepatitis C | 0.003783725 | Acute lymphoid leukemia | 0.001149614 |
| 2021 | Global | Total burden related to hepatitis C | 0.003783725 | Ovarian cancer | 0.003531581 |
| 2021 | Global | Aortic aneurysm | 0.00375752 | Chronic lymphoid leukemia | 0.000785173 |
| 2021 | Global | Ovarian cancer | 0.003634184 | Kidney cancer | 0.005813621 |
| 2021 | Global | Liver cancer due to hepatitis B | 0.003407866 | Liver cancer due to alcohol use | 0.002027584 |
| 2021 | Global | Total burden related to hepatitis B | 0.003407866 | Aortic aneurysm | 0.001860137 |
| 2021 | Global | Lower extremity peripheral arterial disease | 0.00312891 | Liver cancer due to hepatitis B | 0.003710575 |
| 2021 | Global | Subarachnoid hemorrhage | 0.003027201 | Thyroid cancer | 0.001086392 |
| 2021 | Global | Chronic kidney disease due to glomerulonephritis | 0.002956419 | Acute myeloid leukemia | 0.002428235 |
| 2021 | Global | Other non-Hodgkin lymphoma | 0.002856645 | Gallbladder and biliary diseases | 0.021266157 |
| 2021 | Global | Other non-Hodgkin lymphoma | 0.002702076 | Chronic kidney disease due to hypertension | 0.032393347 |
| 2021 | Global | Acute myeloid leukemia | 0.002665564 | Diabetes mellitus type 2 | 0.292569933 |
| 2021 | Global | Aortic aneurysm | 0.002605027 | Total Cancers excluding Non-melanoma skin cancer | 0.066051671 |
| 2021 | Global | Liver cancer due to alcohol use | 0.00236124 | Liver cancer due to hepatitis C | 0.002883805 |
| 2021 | Global | Acute myeloid leukemia | 0.002278009 | Other non-Hodgkin lymphoma | 0.00253943 |
| 2021 | Global | Subarachnoid hemorrhage | 0.002154799 | Burkitt lymphoma | 0.00 |
| 2021 | Global | Liver cancer due to hepatitis C | 0.002091363 | Chronic myeloid leukemia | 0.000418509 |
| 2021 | Global | Total burden related to hepatitis C | 0.002091363 | Chronic kidney disease due to other and unspecified causes | 0.009180004 |
| 2021 | Global | Multiple myeloma | 0.002040002 | Asthma | 0.025481868 |
| 2021 | Global | Multiple myeloma | 0.001681925 | Total burden related to hepatitis C | 0.002883805 |
| 2021 | Global | Liver cancer due to hepatitis B | 0.001671077 | Total burden related to hepatitis B | 0.003710575 |
| 2021 | Global | Total burden related to hepatitis B | 0.001671077 | Ischemic stroke | 0.033304652 |
| 2021 | Global | Chronic lymphoid leukemia | 0.001471894 | Lower extremity peripheral arterial disease | 0.002310304 |
| 2021 | Global | Liver cancer due to alcohol use | 0.001293474 | Uterine cancer | 0.006507087 |
| 2021 | Global | Thyroid cancer | 0.001197997 | Colon and rectum cancer | 0.017668 |
| 2021 | Global | Multidrug-resistant tuberculosis without extensive drug resistance | 0.00119643 | Chronic kidney disease due to diabetes mellitus type 2 | 0.032413804 |
| 2021 | Global | Thyroid cancer | 0.001171062 | Alzheimer's disease and other dementias | 0.021245087 |
| 2021 | Global | Chronic myeloid leukemia | 0.0010946 | Liver cancer due to other causes | 0.000533688 |
| 2021 | Global | Chronic lymphoid leukemia | 0.001083725 | Multidrug-resistant tuberculosis without extensive drug resistance | 0.00143165 |
| 2021 | Global | Acute lymphoid leukemia | 0.000998529 | Drug-susceptible tuberculosis | 0.013981437 |
| 2021 | Global | Other leukemia | 0.000988021 | Chronic kidney disease due to glomerulonephritis | 0.004934716 |
| 2021 | Global | Other leukemia | 0.000939019 | Hypertensive heart disease | 0.095251533 |
| 2021 | Global | Acute lymphoid leukemia | 0.000918587 | Multiple myeloma | 0.00154596 |
| 2021 | Global | Liver cancer due to other causes | 0.00055229 | Total cancers | 0.066051671 |
| 2021 | Global | Chronic myeloid leukemia | 0.000459076 | Ischemic heart disease | 0.179502161 |
| 2021 | Global | Liver cancer due to other causes | 0.000282331 | Other leukemia | 0.000854654 |
| 2021 | Global | Multidrug-resistant tuberculosis without extensive drug resistance | 0.000239725 | Subarachnoid hemorrhage | 0.003969897 |
| 2021 | Global | Burkitt lymphoma | 0.000038 | Intracerebral hemorrhage | 0.020163018 |
| 2021 | Global | Burkitt lymphoma | 0.0000228 | Breast cancer | 0.007516423 |
| 1990 | High SDI | Ischemic heart disease | 0.317944156 | Atrial fibrillation and flutter | 0.005900898 |
| 1990 | High SDI | Ischemic heart disease | 0.186666943 | Gallbladder and biliary tract cancer | 0.005667452 |
| 1990 | High SDI | Total Cancers excluding Non-melanoma skin cancer | 0.127462127 | Acute lymphoid leukemia | 0.001165554 |
| 1990 | High SDI | Total cancers | 0.127462127 | Ovarian cancer | 0.006188628 |
| 1990 | High SDI | Total Cancers excluding Non-melanoma skin cancer | 0.109872211 | Chronic lymphoid leukemia | 0.001779604 |
| 1990 | High SDI | Total cancers | 0.109872211 | Kidney cancer | 0.010682967 |
| 1990 | High SDI | Diabetes mellitus type 2 | 0.105710939 | Liver cancer due to alcohol use | 0.001691593 |
| 1990 | High SDI | Diabetes mellitus type 2 | 0.101919044 | Aortic aneurysm | 0.005199217 |
| 1990 | High SDI | Hypertensive heart disease | 0.092427673 | Liver cancer due to hepatitis B | 0.001524591 |
| 1990 | High SDI | Hypertensive heart disease | 0.070167614 | Thyroid cancer | 0.001303407 |
| 1990 | High SDI | Alzheimer's disease and other dementias | 0.048167101 | Acute myeloid leukemia | 0.00375708 |
| 1990 | High SDI | Chronic kidney disease due to hypertension | 0.046140335 | Gallbladder and biliary diseases | 0.027682132 |
| 1990 | High SDI | Chronic kidney disease due to diabetes mellitus type 2 | 0.04481246 | Chronic kidney disease due to hypertension | 0.015057831 |
| 1990 | High SDI | Ischemic stroke | 0.039988984 | Diabetes mellitus type 2 | 0.164897356 |
| 1990 | High SDI | Colon and rectum cancer | 0.035463147 | Total Cancers excluding Non-melanoma skin cancer | 0.0997815 |
| 1990 | High SDI | Colon and rectum cancer | 0.035358436 | Liver cancer due to hepatitis C | 0.002082498 |
| 1990 | High SDI | Alzheimer's disease and other dementias | 0.032000153 | Other non-Hodgkin lymphoma | 0.0045057 |
| 1990 | High SDI | Ischemic stroke | 0.023573673 | Burkitt lymphoma | 0.00 |
| 1990 | High SDI | Chronic kidney disease due to diabetes mellitus type 2 | 0.018572637 | Chronic myeloid leukemia | 0.001784512 |
| 1990 | High SDI | Chronic kidney disease due to hypertension | 0.016492863 | Chronic kidney disease due to other and unspecified causes | 0.00671131 |
| 1990 | High SDI | Breast cancer | 0.016132382 | Asthma | 0.052343875 |
| 1990 | High SDI | Breast cancer | 0.014557793 | Total burden related to hepatitis C | 0.002082498 |
| 1990 | High SDI | Kidney cancer | 0.014164807 | Total burden related to hepatitis B | 0.001524591 |
| 1990 | High SDI | Asthma | 0.012717605 | Ischemic stroke | 0.033312014 |
| 1990 | High SDI | Gallbladder and biliary diseases | 0.012227729 | Lower extremity peripheral arterial disease | 0.00401097 |
| 1990 | High SDI | Gallbladder and biliary diseases | 0.011676819 | Uterine cancer | 0.008155846 |
| 1990 | High SDI | Uterine cancer | 0.011618775 | Colon and rectum cancer | 0.030686739 |
| 1990 | High SDI | Kidney cancer | 0.011053508 | Chronic kidney disease due to diabetes mellitus type 2 | 0.019929603 |
| 1990 | High SDI | Intracerebral hemorrhage | 0.010796388 | Alzheimer's disease and other dementias | 0.021953835 |
| 1990 | High SDI | Chronic kidney disease due to other and unspecified causes | 0.010792422 | Liver cancer due to other causes | 0.000359201 |
| 1990 | High SDI | Atrial fibrillation and flutter | 0.010404712 | Multidrug-resistant tuberculosis without extensive drug resistance | 0.000100442 |
| 1990 | High SDI | Intracerebral hemorrhage | 0.009251096 | Drug-susceptible tuberculosis | 0.004080904 |
| 1990 | High SDI | Uterine cancer | 0.008864822 | Chronic kidney disease due to glomerulonephritis | 0.002468468 |
| 1990 | High SDI | Gallbladder and biliary tract cancer | 0.00691258 | Hypertensive heart disease | 0.054299083 |
| 1990 | High SDI | Aortic aneurysm | 0.006892381 | Multiple myeloma | 0.002760824 |
| 1990 | High SDI | Ovarian cancer | 0.00636201 | Total cancers | 0.0997815 |
| 1990 | High SDI | Chronic kidney disease due to other and unspecified causes | 0.006291887 | Ischemic heart disease | 0.264686707 |
| 1990 | High SDI | Ovarian cancer | 0.006251868 | Other leukemia | 0.00068361 |
| 1990 | High SDI | Lower extremity peripheral arterial disease | 0.0059255 | Subarachnoid hemorrhage | 0.004962829 |
| 1990 | High SDI | Liver cancer due to hepatitis C | 0.005916508 | Intracerebral hemorrhage | 0.010777179 |
| 1990 | High SDI | Total burden related to hepatitis C | 0.005916508 | Breast cancer | 0.01363215 |
| 2021 | High SDI | Gallbladder and biliary tract cancer | 0.005874601 | Atrial fibrillation and flutter | 0.009402699 |
| 2021 | High SDI | Atrial fibrillation and flutter | 0.005686389 | Gallbladder and biliary tract cancer | 0.003810502 |
| 2021 | High SDI | Acute myeloid leukemia | 0.005293081 | Acute lymphoid leukemia | 0.00091687 |
| 2021 | High SDI | Other non-Hodgkin lymphoma | 0.00504502 | Ovarian cancer | 0.005166049 |
| 2021 | High SDI | Lower extremity peripheral arterial disease | 0.005026923 | Chronic lymphoid leukemia | 0.001128663 |
| 2021 | High SDI | Asthma | 0.004899832 | Kidney cancer | 0.010684717 |
| 2021 | High SDI | Chronic kidney disease due to glomerulonephritis | 0.004884303 | Liver cancer due to alcohol use | 0.003698784 |
| 2021 | High SDI | Liver cancer due to alcohol use | 0.004718462 | Aortic aneurysm | 0.003016553 |
| 2021 | High SDI | Aortic aneurysm | 0.004607894 | Liver cancer due to hepatitis B | 0.002743326 |
| 2021 | High SDI | Other non-Hodgkin lymphoma | 0.004486784 | Thyroid cancer | 0.001157144 |
| 2021 | High SDI | Multiple myeloma | 0.004353745 | Acute myeloid leukemia | 0.004104618 |
| 2021 | High SDI | Drug-susceptible tuberculosis | 0.003848789 | Gallbladder and biliary diseases | 0.026116213 |
| 2021 | High SDI | Subarachnoid hemorrhage | 0.003625295 | Chronic kidney disease due to hypertension | 0.032358368 |
| 2021 | High SDI | Acute myeloid leukemia | 0.003580272 | Diabetes mellitus type 2 | 0.270516657 |
| 2021 | High SDI | Subarachnoid hemorrhage | 0.003311133 | Total Cancers excluding Non-melanoma skin cancer | 0.096759271 |
| 2021 | High SDI | Multiple myeloma | 0.003183391 | Liver cancer due to hepatitis C | 0.004112701 |
| 2021 | High SDI | Liver cancer due to hepatitis B | 0.002894098 | Other non-Hodgkin lymphoma | 0.0039326 |
| 2021 | High SDI | Total burden related to hepatitis B | 0.002894098 | Burkitt lymphoma | 0.00 |
| 2021 | High SDI | Chronic kidney disease due to glomerulonephritis | 0.002548961 | Chronic myeloid leukemia | 0.000485679 |
| 2021 | High SDI | Liver cancer due to hepatitis C | 0.002305055 | Chronic kidney disease due to other and unspecified causes | 0.008803857 |
| 2021 | High SDI | Total burden related to hepatitis C | 0.002305055 | Asthma | 0.041094434 |
| 2021 | High SDI | Chronic lymphoid leukemia | 0.002224724 | Total burden related to hepatitis C | 0.004112701 |
| 2021 | High SDI | Chronic lymphoid leukemia | 0.001798741 | Total burden related to hepatitis B | 0.002743326 |
| 2021 | High SDI | Liver cancer due to alcohol use | 0.001735764 | Ischemic stroke | 0.022364331 |
| 2021 | High SDI | Chronic myeloid leukemia | 0.001705063 | Lower extremity peripheral arterial disease | 0.004089634 |
| 2021 | High SDI | Drug-susceptible tuberculosis | 0.001502334 | Uterine cancer | 0.009635193 |
| 2021 | High SDI | Thyroid cancer | 0.001323545 | Colon and rectum cancer | 0.026468405 |
| 2021 | High SDI | Thyroid cancer | 0.001316859 | Chronic kidney disease due to diabetes mellitus type 2 | 0.033968216 |
| 2021 | High SDI | Liver cancer due to hepatitis B | 0.001260977 | Alzheimer's disease and other dementias | 0.028290645 |
| 2021 | High SDI | Total burden related to hepatitis B | 0.001260977 | Liver cancer due to other causes | 0.000818235 |
| 2021 | High SDI | Other leukemia | 0.001094934 | Multidrug-resistant tuberculosis without extensive drug resistance | 0.00 |
| 2021 | High SDI | Liver cancer due to other causes | 0.000944464 | Drug-susceptible tuberculosis | 0.001528531 |
| 2021 | High SDI | Acute lymphoid leukemia | 0.000849123 | Chronic kidney disease due to glomerulonephritis | 0.004166778 |
| 2021 | High SDI | Acute lymphoid leukemia | 0.000806434 | Hypertensive heart disease | 0.064357842 |
| 2021 | High SDI | Other leukemia | 0.000763982 | Multiple myeloma | 0.002971556 |
| 2021 | High SDI | Chronic myeloid leukemia | 0.000622542 | Total cancers | 0.096759271 |
| 2021 | High SDI | Liver cancer due to other causes | 0.000339355 | Ischemic heart disease | 0.140195351 |
| 2021 | High SDI | Multidrug-resistant tuberculosis without extensive drug resistance | 0.0000963 | Other leukemia | 0.000774891 |
| 2021 | High SDI | Burkitt lymphoma | 0.0000769 | Subarachnoid hemorrhage | 0.004683024 |
| 2021 | High SDI | Multidrug-resistant tuberculosis without extensive drug resistance | 0.0000666 | Intracerebral hemorrhage | 0.01141956 |
| 2021 | High SDI | Burkitt lymphoma | 0.0000342 | Breast cancer | 0.010487381 |
| 1990 | High-middle SDI | Ischemic heart disease | 0.336950931 | Atrial fibrillation and flutter | 0.004256414 |
| 1990 | High-middle SDI | Ischemic heart disease | 0.274405601 | Gallbladder and biliary tract cancer | 0.004955 |
| 1990 | High-middle SDI | Hypertensive heart disease | 0.121571895 | Acute lymphoid leukemia | 0.001721679 |
| 1990 | High-middle SDI | Hypertensive heart disease | 0.114459433 | Ovarian cancer | 0.004309319 |
| 1990 | High-middle SDI | Diabetes mellitus type 2 | 0.105134283 | Chronic lymphoid leukemia | 0.001248044 |
| 1990 | High-middle SDI | Total Cancers excluding Non-melanoma skin cancer | 0.091988678 | Kidney cancer | 0.007213282 |
| 1990 | High-middle SDI | Total cancers | 0.091988678 | Liver cancer due to alcohol use | 0.001458784 |
| 1990 | High-middle SDI | Diabetes mellitus type 2 | 0.09039828 | Aortic aneurysm | 0.002375618 |
| 1990 | High-middle SDI | Ischemic stroke | 0.085280465 | Liver cancer due to hepatitis B | 0.002383741 |
| 1990 | High-middle SDI | Total Cancers excluding Non-melanoma skin cancer | 0.07805494 | Thyroid cancer | 0.001241094 |
| 1990 | High-middle SDI | Total cancers | 0.07805494 | Acute myeloid leukemia | 0.002401906 |
| 1990 | High-middle SDI | Ischemic stroke | 0.058192389 | Gallbladder and biliary diseases | 0.028112145 |
| 1990 | High-middle SDI | Alzheimer's disease and other dementias | 0.037482447 | Chronic kidney disease due to hypertension | 0.014707546 |
| 1990 | High-middle SDI | Colon and rectum cancer | 0.029022534 | Diabetes mellitus type 2 | 0.153304424 |
| 1990 | High-middle SDI | Chronic kidney disease due to diabetes mellitus type 2 | 0.02713711 | Total Cancers excluding Non-melanoma skin cancer | 0.078315932 |
| 1990 | High-middle SDI | Chronic kidney disease due to hypertension | 0.025977233 | Liver cancer due to hepatitis C | 0.001711156 |
| 1990 | High-middle SDI | Colon and rectum cancer | 0.02482329 | Other non-Hodgkin lymphoma | 0.002223288 |
| 1990 | High-middle SDI | Alzheimer's disease and other dementias | 0.024659203 | Burkitt lymphoma | 0.00 |
| 1990 | High-middle SDI | Intracerebral hemorrhage | 0.020426999 | Chronic myeloid leukemia | 0.001053026 |
| 1990 | High-middle SDI | Intracerebral hemorrhage | 0.018225215 | Chronic kidney disease due to other and unspecified causes | 0.007543111 |
| 1990 | High-middle SDI | Chronic kidney disease due to diabetes mellitus type 2 | 0.017645321 | Asthma | 0.029781138 |
| 1990 | High-middle SDI | Chronic kidney disease due to hypertension | 0.01564045 | Total burden related to hepatitis C | 0.001711156 |
| 1990 | High-middle SDI | Gallbladder and biliary diseases | 0.013586739 | Total burden related to hepatitis B | 0.002383741 |
| 1990 | High-middle SDI | Asthma | 0.012852234 | Ischemic stroke | 0.070419999 |
| 1990 | High-middle SDI | Breast cancer | 0.011049279 | Lower extremity peripheral arterial disease | 0.003788949 |
| 1990 | High-middle SDI | Breast cancer | 0.010377298 | Uterine cancer | 0.010036253 |
| 1990 | High-middle SDI | Gallbladder and biliary diseases | 0.010298389 | Colon and rectum cancer | 0.023469185 |
| 1990 | High-middle SDI | Chronic kidney disease due to other and unspecified causes | 0.009920274 | Chronic kidney disease due to diabetes mellitus type 2 | 0.019251858 |
| 1990 | High-middle SDI | Uterine cancer | 0.009479175 | Alzheimer's disease and other dementias | 0.017694503 |
| 1990 | High-middle SDI | Kidney cancer | 0.008975902 | Liver cancer due to other causes | 0.000258336 |
| 1990 | High-middle SDI | Uterine cancer | 0.008875767 | Multidrug-resistant tuberculosis without extensive drug resistance | 0.000270632 |
| 1990 | High-middle SDI | Drug-susceptible tuberculosis | 0.007656302 | Drug-susceptible tuberculosis | 0.00980151 |
| 1990 | High-middle SDI | Chronic kidney disease due to other and unspecified causes | 0.007310163 | Chronic kidney disease due to glomerulonephritis | 0.003176395 |
| 1990 | High-middle SDI | Kidney cancer | 0.006924465 | Hypertensive heart disease | 0.087428002 |
| 1990 | High-middle SDI | Atrial fibrillation and flutter | 0.006740454 | Multiple myeloma | 0.001204537 |
| 1990 | High-middle SDI | Gallbladder and biliary tract cancer | 0.005544051 | Total cancers | 0.078315932 |
| 1990 | High-middle SDI | Lower extremity peripheral arterial disease | 0.005098413 | Ischemic heart disease | 0.284337435 |
| 1990 | High-middle SDI | Asthma | 0.00500437 | Other leukemia | 0.00136765 |
| 1990 | High-middle SDI | Gallbladder and biliary tract cancer | 0.004889759 | Subarachnoid hemorrhage | 0.004069428 |
| 1990 | High-middle SDI | Ovarian cancer | 0.004459292 | Intracerebral hemorrhage | 0.021273129 |
| 1990 | High-middle SDI | Atrial fibrillation and flutter | 0.004302455 | Breast cancer | 0.0093994 |
| 2021 | High-middle SDI | Liver cancer due to hepatitis B | 0.004274407 | Atrial fibrillation and flutter | 0.00593062 |
| 2021 | High-middle SDI | Total burden related to hepatitis B | 0.004274407 | Gallbladder and biliary tract cancer | 0.003974035 |
| 2021 | High-middle SDI | Lower extremity peripheral arterial disease | 0.003876272 | Acute lymphoid leukemia | 0.001430534 |
| 2021 | High-middle SDI | Ovarian cancer | 0.003827305 | Ovarian cancer | 0.004498859 |
| 2021 | High-middle SDI | Chronic kidney disease due to glomerulonephritis | 0.003641691 | Chronic lymphoid leukemia | 0.001063231 |
| 2021 | High-middle SDI | Liver cancer due to hepatitis C | 0.003230349 | Kidney cancer | 0.008188072 |
| 2021 | High-middle SDI | Total burden related to hepatitis C | 0.003230349 | Liver cancer due to alcohol use | 0.002012675 |
| 2021 | High-middle SDI | Subarachnoid hemorrhage | 0.003124921 | Aortic aneurysm | 0.002521377 |
| 2021 | High-middle SDI | Chronic kidney disease due to glomerulonephritis | 0.003034932 | Liver cancer due to hepatitis B | 0.005145318 |
| 2021 | High-middle SDI | Aortic aneurysm | 0.003017671 | Thyroid cancer | 0.001035159 |
| 2021 | High-middle SDI | Subarachnoid hemorrhage | 0.002787418 | Acute myeloid leukemia | 0.002616452 |
| 2021 | High-middle SDI | Drug-susceptible tuberculosis | 0.002742428 | Gallbladder and biliary diseases | 0.025463406 |
| 2021 | High-middle SDI | Aortic aneurysm | 0.00266345 | Chronic kidney disease due to hypertension | 0.020506041 |
| 2021 | High-middle SDI | Other non-Hodgkin lymphoma | 0.002583928 | Diabetes mellitus type 2 | 0.235570669 |
| 2021 | High-middle SDI | Acute myeloid leukemia | 0.002503464 | Total Cancers excluding Non-melanoma skin cancer | 0.084104256 |
| 2021 | High-middle SDI | Liver cancer due to alcohol use | 0.002169865 | Liver cancer due to hepatitis C | 0.002554873 |
| 2021 | High-middle SDI | Other non-Hodgkin lymphoma | 0.001986923 | Other non-Hodgkin lymphoma | 0.002597736 |
| 2021 | High-middle SDI | Multiple myeloma | 0.001939914 | Burkitt lymphoma | 0.00 |
| 2021 | High-middle SDI | Liver cancer due to hepatitis C | 0.001926884 | Chronic myeloid leukemia | 0.00037958 |
| 2021 | High-middle SDI | Total burden related to hepatitis C | 0.001926884 | Chronic kidney disease due to other and unspecified causes | 0.008448897 |
| 2021 | High-middle SDI | Acute myeloid leukemia | 0.001918918 | Asthma | 0.016145796 |
| 2021 | High-middle SDI | Liver cancer due to hepatitis B | 0.001816252 | Total burden related to hepatitis C | 0.002554873 |
| 2021 | High-middle SDI | Total burden related to hepatitis B | 0.001816252 | Total burden related to hepatitis B | 0.005145318 |
| 2021 | High-middle SDI | Liver cancer due to alcohol use | 0.00142759 | Ischemic stroke | 0.049652103 |
| 2021 | High-middle SDI | Chronic lymphoid leukemia | 0.001340493 | Lower extremity peripheral arterial disease | 0.003042606 |
| 2021 | High-middle SDI | Chronic lymphoid leukemia | 0.001263913 | Uterine cancer | 0.008636368 |
| 2021 | High-middle SDI | Multiple myeloma | 0.001253119 | Colon and rectum cancer | 0.024936938 |
| 2021 | High-middle SDI | Thyroid cancer | 0.001199084 | Chronic kidney disease due to diabetes mellitus type 2 | 0.024424983 |
| 2021 | High-middle SDI | Other leukemia | 0.001190934 | Alzheimer's disease and other dementias | 0.025480343 |
| 2021 | High-middle SDI | Acute lymphoid leukemia | 0.001146443 | Liver cancer due to other causes | 0.000488868 |
| 2021 | High-middle SDI | Acute lymphoid leukemia | 0.001081391 | Multidrug-resistant tuberculosis without extensive drug resistance | 0.000658043 |
| 2021 | High-middle SDI | Other leukemia | 0.001068094 | Drug-susceptible tuberculosis | 0.003840348 |
| 2021 | High-middle SDI | Thyroid cancer | 0.001058263 | Chronic kidney disease due to glomerulonephritis | 0.003221619 |
| 2021 | High-middle SDI | Chronic myeloid leukemia | 0.000945054 | Hypertensive heart disease | 0.075613491 |
| 2021 | High-middle SDI | Multidrug-resistant tuberculosis without extensive drug resistance | 0.000480738 | Multiple myeloma | 0.001661795 |
| 2021 | High-middle SDI | Liver cancer due to other causes | 0.000458623 | Total cancers | 0.084104256 |
| 2021 | High-middle SDI | Chronic myeloid leukemia | 0.000405495 | Ischemic heart disease | 0.214302073 |
| 2021 | High-middle SDI | Multidrug-resistant tuberculosis without extensive drug resistance | 0.000227338 | Other leukemia | 0.001084635 |
| 2021 | High-middle SDI | Liver cancer due to other causes | 0.000222594 | Subarachnoid hemorrhage | 0.004324548 |
| 2021 | High-middle SDI | Burkitt lymphoma | 0.0000325 | Intracerebral hemorrhage | 0.023197538 |
| 2021 | High-middle SDI | Burkitt lymphoma | 0.0000184 | Breast cancer | 0.00939982 |
| 1990 | Middle SDI | Hypertensive heart disease | 0.25787952 | Atrial fibrillation and flutter | 0.001949478 |
| 1990 | Middle SDI | Ischemic heart disease | 0.214002139 | Gallbladder and biliary tract cancer | 0.003905032 |
| 1990 | Middle SDI | Ischemic heart disease | 0.201600278 | Acute lymphoid leukemia | 0.001949425 |
| 1990 | Middle SDI | Diabetes mellitus type 2 | 0.200109433 | Ovarian cancer | 0.001444116 |
| 1990 | Middle SDI | Diabetes mellitus type 2 | 0.18761653 | Chronic lymphoid leukemia | 0.000714288 |
| 1990 | Middle SDI | Hypertensive heart disease | 0.162397703 | Kidney cancer | 0.002492358 |
| 1990 | Middle SDI | Total Cancers excluding Non-melanoma skin cancer | 0.058602795 | Liver cancer due to alcohol use | 0.000842279 |
| 1990 | Middle SDI | Total cancers | 0.058602795 | Aortic aneurysm | 0.001021298 |
| 1990 | Middle SDI | Chronic kidney disease due to diabetes mellitus type 2 | 0.048216265 | Liver cancer due to hepatitis B | 0.002868317 |
| 1990 | Middle SDI | Chronic kidney disease due to hypertension | 0.046561683 | Thyroid cancer | 0.001116134 |
| 1990 | Middle SDI | Total Cancers excluding Non-melanoma skin cancer | 0.044375263 | Acute myeloid leukemia | 0.002028986 |
| 1990 | Middle SDI | Total cancers | 0.044375263 | Gallbladder and biliary diseases | 0.027918755 |
| 1990 | Middle SDI | Chronic kidney disease due to diabetes mellitus type 2 | 0.038926745 | Chronic kidney disease due to hypertension | 0.029276611 |
| 1990 | Middle SDI | Ischemic stroke | 0.038318325 | Diabetes mellitus type 2 | 0.271142604 |
| 1990 | Middle SDI | Ischemic stroke | 0.036688054 | Total Cancers excluding Non-melanoma skin cancer | 0.043315755 |
| 1990 | Middle SDI | Chronic kidney disease due to hypertension | 0.034633595 | Liver cancer due to hepatitis C | 0.001290565 |
| 1990 | Middle SDI | Asthma | 0.028280497 | Other non-Hodgkin lymphoma | 0.001876467 |
| 1990 | Middle SDI | Drug-susceptible tuberculosis | 0.027941422 | Burkitt lymphoma | 0.00 |
| 1990 | Middle SDI | Alzheimer's disease and other dementias | 0.025999261 | Chronic myeloid leukemia | 0.000844559 |
| 1990 | Middle SDI | Intracerebral hemorrhage | 0.022095864 | Chronic kidney disease due to other and unspecified causes | 0.005231139 |
| 1990 | Middle SDI | Colon and rectum cancer | 0.016114095 | Asthma | 0.03571678 |
| 1990 | Middle SDI | Gallbladder and biliary diseases | 0.015164162 | Total burden related to hepatitis C | 0.001290565 |
| 1990 | Middle SDI | Asthma | 0.014708642 | Total burden related to hepatitis B | 0.002868317 |
| 1990 | Middle SDI | Alzheimer's disease and other dementias | 0.011804284 | Ischemic stroke | 0.032312663 |
| 1990 | Middle SDI | Colon and rectum cancer | 0.011507498 | Lower extremity peripheral arterial disease | 0.000963331 |
| 1990 | Middle SDI | Gallbladder and biliary diseases | 0.011264009 | Uterine cancer | 0.004983819 |
| 1990 | Middle SDI | Drug-susceptible tuberculosis | 0.009996593 | Colon and rectum cancer | 0.010683294 |
| 1990 | Middle SDI | Intracerebral hemorrhage | 0.008453151 | Chronic kidney disease due to diabetes mellitus type 2 | 0.034892782 |
| 1990 | Middle SDI | Breast cancer | 0.007399414 | Alzheimer's disease and other dementias | 0.008117171 |
| 1990 | Middle SDI | Chronic kidney disease due to other and unspecified causes | 0.007225685 | Liver cancer due to other causes | 0.000306366 |
| 1990 | Middle SDI | Breast cancer | 0.005669671 | Multidrug-resistant tuberculosis without extensive drug resistance | 0.000553187 |
| 1990 | Middle SDI | Chronic kidney disease due to glomerulonephritis | 0.005382588 | Drug-susceptible tuberculosis | 0.03133922 |
| 1990 | Middle SDI | Uterine cancer | 0.005004367 | Chronic kidney disease due to glomerulonephritis | 0.003225067 |
| 1990 | Middle SDI | Uterine cancer | 0.004958369 | Hypertensive heart disease | 0.190900164 |
| 1990 | Middle SDI | Atrial fibrillation and flutter | 0.004716276 | Multiple myeloma | 0.000558646 |
| 1990 | Middle SDI | Gallbladder and biliary tract cancer | 0.004545074 | Total cancers | 0.043315755 |
| 1990 | Middle SDI | Chronic kidney disease due to other and unspecified causes | 0.004396474 | Ischemic heart disease | 0.177692769 |
| 1990 | Middle SDI | Liver cancer due to hepatitis B | 0.004254487 | Other leukemia | 0.001362046 |
| 1990 | Middle SDI | Total burden related to hepatitis B | 0.004254487 | Subarachnoid hemorrhage | 0.001872288 |
| 1990 | Middle SDI | Kidney cancer | 0.004052972 | Intracerebral hemorrhage | 0.011170389 |
| 1990 | Middle SDI | Gallbladder and biliary tract cancer | 0.004021573 | Breast cancer | 0.004628169 |
| 2021 | Middle SDI | Subarachnoid hemorrhage | 0.003354609 | Atrial fibrillation and flutter | 0.004059897 |
| 2021 | Middle SDI | Chronic kidney disease due to glomerulonephritis | 0.003065388 | Gallbladder and biliary tract cancer | 0.003177219 |
| 2021 | Middle SDI | Ovarian cancer | 0.002681466 | Acute lymphoid leukemia | 0.001471436 |
| 2021 | Middle SDI | Liver cancer due to hepatitis C | 0.002674491 | Ovarian cancer | 0.002769235 |
| 2021 | Middle SDI | Total burden related to hepatitis C | 0.002674491 | Chronic lymphoid leukemia | 0.000562517 |
| 2021 | Middle SDI | Kidney cancer | 0.002596254 | Kidney cancer | 0.003615447 |
| 2021 | Middle SDI | Liver cancer due to hepatitis B | 0.002389756 | Liver cancer due to alcohol use | 0.001537367 |
| 2021 | Middle SDI | Total burden related to hepatitis B | 0.002389756 | Aortic aneurysm | 0.001207851 |
| 2021 | Middle SDI | Other non-Hodgkin lymphoma | 0.002105431 | Liver cancer due to hepatitis B | 0.004585661 |
| 2021 | Middle SDI | Atrial fibrillation and flutter | 0.002066018 | Thyroid cancer | 0.001123169 |
| 2021 | Middle SDI | Acute myeloid leukemia | 0.001829789 | Acute myeloid leukemia | 0.001966033 |
| 2021 | Middle SDI | Other non-Hodgkin lymphoma | 0.001754936 | Gallbladder and biliary diseases | 0.023053586 |
| 2021 | Middle SDI | Liver cancer due to alcohol use | 0.001720862 | Chronic kidney disease due to hypertension | 0.036644635 |
| 2021 | Middle SDI | Acute myeloid leukemia | 0.00169882 | Diabetes mellitus type 2 | 0.323913821 |
| 2021 | Middle SDI | Aortic aneurysm | 0.001575131 | Total Cancers excluding Non-melanoma skin cancer | 0.052890069 |
| 2021 | Middle SDI | Liver cancer due to hepatitis C | 0.001555588 | Liver cancer due to hepatitis C | 0.002009068 |
| 2021 | Middle SDI | Total burden related to hepatitis C | 0.001555588 | Other non-Hodgkin lymphoma | 0.002081351 |
| 2021 | Middle SDI | Acute lymphoid leukemia | 0.001407547 | Burkitt lymphoma | 0.00 |
| 2021 | Middle SDI | Ovarian cancer | 0.001325409 | Chronic myeloid leukemia | 0.000419182 |
| 2021 | Middle SDI | Aortic aneurysm | 0.001245569 | Chronic kidney disease due to other and unspecified causes | 0.008149729 |
| 2021 | Middle SDI | Thyroid cancer | 0.001232107 | Asthma | 0.021056758 |
| 2021 | Middle SDI | Other leukemia | 0.001181403 | Total burden related to hepatitis C | 0.002009068 |
| 2021 | Middle SDI | Thyroid cancer | 0.001179684 | Total burden related to hepatitis B | 0.004585661 |
| 2021 | Middle SDI | Multiple myeloma | 0.00116785 | Ischemic stroke | 0.032674164 |
| 2021 | Middle SDI | Acute lymphoid leukemia | 0.00115555 | Lower extremity peripheral arterial disease | 0.001152724 |
| 2021 | Middle SDI | Lower extremity peripheral arterial disease | 0.00113306 | Uterine cancer | 0.00467137 |
| 2021 | Middle SDI | Multidrug-resistant tuberculosis without extensive drug resistance | 0.001014794 | Colon and rectum cancer | 0.013801976 |
| 2021 | Middle SDI | Subarachnoid hemorrhage | 0.000987662 | Chronic kidney disease due to diabetes mellitus type 2 | 0.03848893 |
| 2021 | Middle SDI | Lower extremity peripheral arterial disease | 0.000954468 | Alzheimer's disease and other dementias | 0.017036758 |
| 2021 | Middle SDI | Other leukemia | 0.000863398 | Liver cancer due to other causes | 0.000463322 |
| 2021 | Middle SDI | Liver cancer due to alcohol use | 0.000855373 | Multidrug-resistant tuberculosis without extensive drug resistance | 0.001179295 |
| 2021 | Middle SDI | Chronic lymphoid leukemia | 0.000750977 | Drug-susceptible tuberculosis | 0.012075037 |
| 2021 | Middle SDI | Chronic myeloid leukemia | 0.000743515 | Chronic kidney disease due to glomerulonephritis | 0.005561121 |
| 2021 | Middle SDI | Chronic lymphoid leukemia | 0.000651301 | Hypertensive heart disease | 0.107289817 |
| 2021 | Middle SDI | Multiple myeloma | 0.0005962 | Multiple myeloma | 0.001013531 |
| 2021 | Middle SDI | Multidrug-resistant tuberculosis without extensive drug resistance | 0.000545948 | Total cancers | 0.052890069 |
| 2021 | Middle SDI | Liver cancer due to other causes | 0.000470255 | Ischemic heart disease | 0.174283916 |
| 2021 | Middle SDI | Chronic myeloid leukemia | 0.000416798 | Other leukemia | 0.000881242 |
| 2021 | Middle SDI | Liver cancer due to other causes | 0.000277094 | Subarachnoid hemorrhage | 0.004310651 |
| 2021 | Middle SDI | Burkitt lymphoma | 0.0000232 | Intracerebral hemorrhage | 0.023333166 |
| 2021 | Middle SDI | Burkitt lymphoma | 0.0000152 | Breast cancer | 0.00597708 |
| 1990 | Low-middle SDI | Hypertensive heart disease | 0.237221505 | Atrial fibrillation and flutter | 0.001509325 |
| 1990 | Low-middle SDI | Diabetes mellitus type 2 | 0.23382517 | Gallbladder and biliary tract cancer | 0.002665832 |
| 1990 | Low-middle SDI | Ischemic heart disease | 0.226222717 | Acute lymphoid leukemia | 0.000968157 |
| 1990 | Low-middle SDI | Ischemic heart disease | 0.216560316 | Ovarian cancer | 0.000926569 |
| 1990 | Low-middle SDI | Diabetes mellitus type 2 | 0.18903684 | Chronic lymphoid leukemia | 0.000273719 |
| 1990 | Low-middle SDI | Hypertensive heart disease | 0.164717573 | Kidney cancer | 0.001323662 |
| 1990 | Low-middle SDI | Drug-susceptible tuberculosis | 0.060119086 | Liver cancer due to alcohol use | 0.000794441 |
| 1990 | Low-middle SDI | Asthma | 0.050526403 | Aortic aneurysm | 0.00066039 |
| 1990 | Low-middle SDI | Chronic kidney disease due to hypertension | 0.045315142 | Liver cancer due to hepatitis B | 0.001649703 |
| 1990 | Low-middle SDI | Total Cancers excluding Non-melanoma skin cancer | 0.04130281 | Thyroid cancer | 0.000955612 |
| 1990 | Low-middle SDI | Total cancers | 0.04130281 | Acute myeloid leukemia | 0.001482398 |
| 1990 | Low-middle SDI | Ischemic stroke | 0.037607466 | Gallbladder and biliary diseases | 0.017276719 |
| 1990 | Low-middle SDI | Chronic kidney disease due to diabetes mellitus type 2 | 0.035487875 | Chronic kidney disease due to hypertension | 0.030703599 |
| 1990 | Low-middle SDI | Ischemic stroke | 0.035412277 | Diabetes mellitus type 2 | 0.250792442 |
| 1990 | Low-middle SDI | Chronic kidney disease due to hypertension | 0.035035307 | Total Cancers excluding Non-melanoma skin cancer | 0.029877553 |
| 1990 | Low-middle SDI | Asthma | 0.033649524 | Liver cancer due to hepatitis C | 0.003050601 |
| 1990 | Low-middle SDI | Chronic kidney disease due to diabetes mellitus type 2 | 0.03216611 | Other non-Hodgkin lymphoma | 0.001466521 |
| 1990 | Low-middle SDI | Total Cancers excluding Non-melanoma skin cancer | 0.030855411 | Burkitt lymphoma | 0.00 |
| 1990 | Low-middle SDI | Total cancers | 0.030855411 | Chronic myeloid leukemia | 0.000722203 |
| 1990 | Low-middle SDI | Drug-susceptible tuberculosis | 0.025542044 | Chronic kidney disease due to other and unspecified causes | 0.006382963 |
| 1990 | Low-middle SDI | Intracerebral hemorrhage | 0.018246838 | Asthma | 0.056240094 |
| 1990 | Low-middle SDI | Alzheimer's disease and other dementias | 0.017020175 | Total burden related to hepatitis C | 0.003050601 |
| 1990 | Low-middle SDI | Intracerebral hemorrhage | 0.011243817 | Total burden related to hepatitis B | 0.001649703 |
| 1990 | Low-middle SDI | Chronic kidney disease due to other and unspecified causes | 0.011020123 | Ischemic stroke | 0.032529433 |
| 1990 | Low-middle SDI | Gallbladder and biliary diseases | 0.010324914 | Lower extremity peripheral arterial disease | 0.000550144 |
| 1990 | Low-middle SDI | Alzheimer's disease and other dementias | 0.009760317 | Uterine cancer | 0.003512833 |
| 1990 | Low-middle SDI | Colon and rectum cancer | 0.008280927 | Colon and rectum cancer | 0.005872027 |
| 1990 | Low-middle SDI | Gallbladder and biliary diseases | 0.007522702 | Chronic kidney disease due to diabetes mellitus type 2 | 0.02994067 |
| 1990 | Low-middle SDI | Breast cancer | 0.006164137 | Alzheimer's disease and other dementias | 0.006845529 |
| 1990 | Low-middle SDI | Colon and rectum cancer | 0.006105501 | Liver cancer due to other causes | 0.000360972 |
| 1990 | Low-middle SDI | Chronic kidney disease due to glomerulonephritis | 0.005811782 | Multidrug-resistant tuberculosis without extensive drug resistance | 0.000154899 |
| 1990 | Low-middle SDI | Chronic kidney disease due to other and unspecified causes | 0.005774895 | Drug-susceptible tuberculosis | 0.065052696 |
| 1990 | Low-middle SDI | Liver cancer due to hepatitis C | 0.004184029 | Chronic kidney disease due to glomerulonephritis | 0.00267784 |
| 1990 | Low-middle SDI | Total burden related to hepatitis C | 0.004184029 | Hypertensive heart disease | 0.185560184 |
| 1990 | Low-middle SDI | Uterine cancer | 0.004041023 | Multiple myeloma | 0.000386205 |
| 1990 | Low-middle SDI | Breast cancer | 0.003744718 | Total cancers | 0.029877553 |
| 1990 | Low-middle SDI | Uterine cancer | 0.003577372 | Ischemic heart disease | 0.203494731 |
| 1990 | Low-middle SDI | Liver cancer due to hepatitis C | 0.003414678 | Other leukemia | 0.000871213 |
| 1990 | Low-middle SDI | Total burden related to hepatitis C | 0.003414678 | Subarachnoid hemorrhage | 0.001775369 |
| 1990 | Low-middle SDI | Atrial fibrillation and flutter | 0.003310346 | Intracerebral hemorrhage | 0.01331456 |
| 1990 | Low-middle SDI | Gallbladder and biliary tract cancer | 0.003166207 | Breast cancer | 0.002782573 |
| 2021 | Low-middle SDI | Gallbladder and biliary tract cancer | 0.002997986 | Atrial fibrillation and flutter | 0.00283395 |
| 2021 | Low-middle SDI | Multidrug-resistant tuberculosis without extensive drug resistance | 0.002965563 | Gallbladder and biliary tract cancer | 0.002616679 |
| 2021 | Low-middle SDI | Chronic kidney disease due to glomerulonephritis | 0.002679261 | Acute lymphoid leukemia | 0.000807091 |
| 2021 | Low-middle SDI | Subarachnoid hemorrhage | 0.002467994 | Ovarian cancer | 0.002207027 |
| 2021 | Low-middle SDI | Kidney cancer | 0.002142456 | Chronic lymphoid leukemia | 0.000281897 |
| 2021 | Low-middle SDI | Ovarian cancer | 0.002124701 | Kidney cancer | 0.001925628 |
| 2021 | Low-middle SDI | Liver cancer due to hepatitis B | 0.00189046 | Liver cancer due to alcohol use | 0.00121275 |
| 2021 | Low-middle SDI | Total burden related to hepatitis B | 0.00189046 | Aortic aneurysm | 0.000923253 |
| 2021 | Low-middle SDI | Other non-Hodgkin lymphoma | 0.00164975 | Liver cancer due to hepatitis B | 0.002033126 |
| 2021 | Low-middle SDI | Atrial fibrillation and flutter | 0.001629429 | Thyroid cancer | 0.000955347 |
| 2021 | Low-middle SDI | Liver cancer due to hepatitis B | 0.001439967 | Acute myeloid leukemia | 0.001527101 |
| 2021 | Low-middle SDI | Total burden related to hepatitis B | 0.001439967 | Gallbladder and biliary diseases | 0.01188181 |
| 2021 | Low-middle SDI | Acute myeloid leukemia | 0.001422252 | Chronic kidney disease due to hypertension | 0.03702455 |
| 2021 | Low-middle SDI | Other non-Hodgkin lymphoma | 0.00138771 | Diabetes mellitus type 2 | 0.33306612 |
| 2021 | Low-middle SDI | Kidney cancer | 0.001363726 | Total Cancers excluding Non-melanoma skin cancer | 0.037161402 |
| 2021 | Low-middle SDI | Liver cancer due to alcohol use | 0.001327205 | Liver cancer due to hepatitis C | 0.00354449 |
| 2021 | Low-middle SDI | Acute myeloid leukemia | 0.00125199 | Other non-Hodgkin lymphoma | 0.001616928 |
| 2021 | Low-middle SDI | Subarachnoid hemorrhage | 0.001233504 | Burkitt lymphoma | 0.00 |
| 2021 | Low-middle SDI | Aortic aneurysm | 0.001204152 | Chronic myeloid leukemia | 0.00039621 |
| 2021 | Low-middle SDI | Thyroid cancer | 0.000998363 | Chronic kidney disease due to other and unspecified causes | 0.011214867 |
| 2021 | Low-middle SDI | Thyroid cancer | 0.000942802 | Asthma | 0.03443074 |
| 2021 | Low-middle SDI | Lower extremity peripheral arterial disease | 0.000838432 | Total burden related to hepatitis C | 0.00354449 |
| 2021 | Low-middle SDI | Ovarian cancer | 0.000826751 | Total burden related to hepatitis B | 0.002033126 |
| 2021 | Low-middle SDI | Liver cancer due to alcohol use | 0.000819495 | Ischemic stroke | 0.029957881 |
| 2021 | Low-middle SDI | Other leukemia | 0.000811282 | Lower extremity peripheral arterial disease | 0.000790144 |
| 2021 | Low-middle SDI | Aortic aneurysm | 0.000807443 | Uterine cancer | 0.003732622 |
| 2021 | Low-middle SDI | Multiple myeloma | 0.000788182 | Colon and rectum cancer | 0.007365447 |
| 2021 | Low-middle SDI | Other leukemia | 0.000695909 | Chronic kidney disease due to diabetes mellitus type 2 | 0.030663458 |
| 2021 | Low-middle SDI | Acute lymphoid leukemia | 0.000693436 | Alzheimer's disease and other dementias | 0.010824504 |
| 2021 | Low-middle SDI | Chronic myeloid leukemia | 0.000636947 | Liver cancer due to other causes | 0.000453447 |
| 2021 | Low-middle SDI | Acute lymphoid leukemia | 0.00063011 | Multidrug-resistant tuberculosis without extensive drug resistance | 0.003162244 |
| 2021 | Low-middle SDI | Lower extremity peripheral arterial disease | 0.000454713 | Drug-susceptible tuberculosis | 0.028136386 |
| 2021 | Low-middle SDI | Liver cancer due to other causes | 0.000447676 | Chronic kidney disease due to glomerulonephritis | 0.005608033 |
| 2021 | Low-middle SDI | Multiple myeloma | 0.00041407 | Hypertensive heart disease | 0.119568747 |
| 2021 | Low-middle SDI | Chronic myeloid leukemia | 0.000406211 | Multiple myeloma | 0.000685159 |
| 2021 | Low-middle SDI | Chronic lymphoid leukemia | 0.0003583 | Total cancers | 0.037161402 |
| 2021 | Low-middle SDI | Liver cancer due to other causes | 0.000335652 | Ischemic heart disease | 0.200121166 |
| 2021 | Low-middle SDI | Chronic lymphoid leukemia | 0.00032946 | Other leukemia | 0.000668178 |
| 2021 | Low-middle SDI | Multidrug-resistant tuberculosis without extensive drug resistance | 0.000146382 | Subarachnoid hemorrhage | 0.003286872 |
| 2021 | Low-middle SDI | Burkitt lymphoma | 0.0000216 | Intracerebral hemorrhage | 0.019953154 |
| 2021 | Low-middle SDI | Burkitt lymphoma | 0.0000133 | Breast cancer | 0.004596072 |
| 1990 | Low SDI | Hypertensive heart disease | 0.287548925 | Atrial fibrillation and flutter | 0.000633576 |
| 1990 | Low SDI | Diabetes mellitus type 2 | 0.247531552 | Gallbladder and biliary tract cancer | 0.001285907 |
| 1990 | Low SDI | Hypertensive heart disease | 0.24183972 | Acute lymphoid leukemia | 0.000907499 |
| 1990 | Low SDI | Diabetes mellitus type 2 | 0.236082591 | Ovarian cancer | 0.000798327 |
| 1990 | Low SDI | Ischemic heart disease | 0.147354756 | Chronic lymphoid leukemia | 0.000561622 |
| 1990 | Low SDI | Ischemic heart disease | 0.12619688 | Kidney cancer | 0.001016362 |
| 1990 | Low SDI | Drug-susceptible tuberculosis | 0.084192461 | Liver cancer due to alcohol use | 0.000791304 |
| 1990 | Low SDI | Asthma | 0.052419214 | Aortic aneurysm | 0.000749605 |
| 1990 | Low SDI | Drug-susceptible tuberculosis | 0.047448704 | Liver cancer due to hepatitis B | 0.002481398 |
| 1990 | Low SDI | Chronic kidney disease due to hypertension | 0.04509405 | Thyroid cancer | 0.001081367 |
| 1990 | Low SDI | Chronic kidney disease due to hypertension | 0.039121849 | Acute myeloid leukemia | 0.000925361 |
| 1990 | Low SDI | Asthma | 0.038719733 | Gallbladder and biliary diseases | 0.012288654 |
| 1990 | Low SDI | Total Cancers excluding Non-melanoma skin cancer | 0.034024029 | Chronic kidney disease due to hypertension | 0.033163896 |
| 1990 | Low SDI | Total cancers | 0.034024029 | Diabetes mellitus type 2 | 0.284602052 |
| 1990 | Low SDI | Chronic kidney disease due to diabetes mellitus type 2 | 0.032087459 | Total Cancers excluding Non-melanoma skin cancer | 0.026133038 |
| 1990 | Low SDI | Chronic kidney disease due to diabetes mellitus type 2 | 0.031903265 | Liver cancer due to hepatitis C | 0.001539256 |
| 1990 | Low SDI | Ischemic stroke | 0.027230583 | Other non-Hodgkin lymphoma | 0.001683906 |
| 1990 | Low SDI | Total Cancers excluding Non-melanoma skin cancer | 0.026867261 | Burkitt lymphoma | 0.00 |
| 1990 | Low SDI | Total cancers | 0.026867261 | Chronic myeloid leukemia | 0.000440035 |
| 1990 | Low SDI | Ischemic stroke | 0.022052536 | Chronic kidney disease due to other and unspecified causes | 0.005701307 |
| 1990 | Low SDI | Intracerebral hemorrhage | 0.020298727 | Asthma | 0.060381204 |
| 1990 | Low SDI | Alzheimer's disease and other dementias | 0.010887925 | Total burden related to hepatitis C | 0.001539256 |
| 1990 | Low SDI | Gallbladder and biliary diseases | 0.010183603 | Total burden related to hepatitis B | 0.002481398 |
| 1990 | Low SDI | Gallbladder and biliary diseases | 0.009988533 | Ischemic stroke | 0.020544262 |
| 1990 | Low SDI | Intracerebral hemorrhage | 0.008533877 | Lower extremity peripheral arterial disease | 0.000474857 |
| 1990 | Low SDI | Chronic kidney disease due to glomerulonephritis | 0.008025709 | Uterine cancer | 0.003247496 |
| 1990 | Low SDI | Chronic kidney disease due to other and unspecified causes | 0.007370785 | Colon and rectum cancer | 0.005400433 |
| 1990 | Low SDI | Colon and rectum cancer | 0.006779973 | Chronic kidney disease due to diabetes mellitus type 2 | 0.02964111 |
| 1990 | Low SDI | Breast cancer | 0.005947336 | Alzheimer's disease and other dementias | 0.002816384 |
| 1990 | Low SDI | Colon and rectum cancer | 0.005618049 | Liver cancer due to other causes | 0.000331946 |
| 1990 | Low SDI | Chronic kidney disease due to glomerulonephritis | 0.005159415 | Multidrug-resistant tuberculosis without extensive drug resistance | 0.000412475 |
| 1990 | Low SDI | Chronic kidney disease due to other and unspecified causes | 0.005049478 | Drug-susceptible tuberculosis | 0.090060642 |
| 1990 | Low SDI | Breast cancer | 0.004024713 | Chronic kidney disease due to glomerulonephritis | 0.004872425 |
| 1990 | Low SDI | Uterine cancer | 0.003940951 | Hypertensive heart disease | 0.238906765 |
| 1990 | Low SDI | Multidrug-resistant tuberculosis without extensive drug resistance | 0.003939732 | Multiple myeloma | 0.000288361 |
| 1990 | Low SDI | Alzheimer's disease and other dementias | 0.003796263 | Total cancers | 0.026133038 |
| 1990 | Low SDI | Uterine cancer | 0.003280522 | Ischemic heart disease | 0.120830819 |
| 1990 | Low SDI | Liver cancer due to hepatitis B | 0.0024554 | Other leukemia | 0.000688491 |
| 1990 | Low SDI | Total burden related to hepatitis B | 0.0024554 | Subarachnoid hemorrhage | 0.000614278 |
| 1990 | Low SDI | Liver cancer due to hepatitis B | 0.002196354 | Intracerebral hemorrhage | 0.01055846 |
| 1990 | Low SDI | Total burden related to hepatitis B | 0.002196354 | Breast cancer | 0.00293191 |
| 2021 | Low SDI | Liver cancer due to hepatitis C | 0.002144662 | Atrial fibrillation and flutter | 0.001634549 |
| 2021 | Low SDI | Total burden related to hepatitis C | 0.002144662 | Gallbladder and biliary tract cancer | 0.001473402 |
| 2021 | Low SDI | Other non-Hodgkin lymphoma | 0.001875638 | Acute lymphoid leukemia | 0.00071217 |
| 2021 | Low SDI | Atrial fibrillation and flutter | 0.001798006 | Ovarian cancer | 0.001746806 |
| 2021 | Low SDI | Gallbladder and biliary tract cancer | 0.001733431 | Chronic lymphoid leukemia | 0.000497015 |
| 2021 | Low SDI | Liver cancer due to hepatitis C | 0.001711924 | Kidney cancer | 0.001468898 |
| 2021 | Low SDI | Total burden related to hepatitis C | 0.001711924 | Liver cancer due to alcohol use | 0.001071202 |
| 2021 | Low SDI | Other non-Hodgkin lymphoma | 0.001625866 | Aortic aneurysm | 0.000929523 |
| 2021 | Low SDI | Ovarian cancer | 0.001612674 | Liver cancer due to hepatitis B | 0.00274125 |
| 2021 | Low SDI | Kidney cancer | 0.001602455 | Thyroid cancer | 0.00101968 |
| 2021 | Low SDI | Subarachnoid hemorrhage | 0.00147662 | Acute myeloid leukemia | 0.000936618 |
| 2021 | Low SDI | Gallbladder and biliary tract cancer | 0.001390064 | Gallbladder and biliary diseases | 0.011464292 |
| 2021 | Low SDI | Aortic aneurysm | 0.001180557 | Chronic kidney disease due to hypertension | 0.036308099 |
| 2021 | Low SDI | Liver cancer due to alcohol use | 0.001123431 | Diabetes mellitus type 2 | 0.335436274 |
| 2021 | Low SDI | Kidney cancer | 0.001062103 | Total Cancers excluding Non-melanoma skin cancer | 0.031181878 |
| 2021 | Low SDI | Thyroid cancer | 0.001025033 | Liver cancer due to hepatitis C | 0.001826429 |
| 2021 | Low SDI | Thyroid cancer | 0.00101339 | Other non-Hodgkin lymphoma | 0.00187462 |
| 2021 | Low SDI | Lower extremity peripheral arterial disease | 0.001008862 | Burkitt lymphoma | 0.00 |
| 2021 | Low SDI | Aortic aneurysm | 0.000938563 | Chronic myeloid leukemia | 0.000291995 |
| 2021 | Low SDI | Acute myeloid leukemia | 0.000846247 | Chronic kidney disease due to other and unspecified causes | 0.00790199 |
| 2021 | Low SDI | Acute myeloid leukemia | 0.000823954 | Asthma | 0.043019937 |
| 2021 | Low SDI | Liver cancer due to alcohol use | 0.000800547 | Total burden related to hepatitis C | 0.001826429 |
| 2021 | Low SDI | Ovarian cancer | 0.000697584 | Total burden related to hepatitis B | 0.00274125 |
| 2021 | Low SDI | Acute lymphoid leukemia | 0.000662858 | Ischemic stroke | 0.024114338 |
| 2021 | Low SDI | Chronic lymphoid leukemia | 0.000657196 | Lower extremity peripheral arterial disease | 0.000831626 |
| 2021 | Low SDI | Other leukemia | 0.000647929 | Uterine cancer | 0.003728326 |
| 2021 | Low SDI | Chronic lymphoid leukemia | 0.000635232 | Colon and rectum cancer | 0.006030983 |
| 2021 | Low SDI | Atrial fibrillation and flutter | 0.000617808 | Chronic kidney disease due to diabetes mellitus type 2 | 0.028058454 |
| 2021 | Low SDI | Multiple myeloma | 0.000561871 | Alzheimer's disease and other dementias | 0.007169479 |
| 2021 | Low SDI | Other leukemia | 0.000556366 | Liver cancer due to other causes | 0.000392492 |
| 2021 | Low SDI | Acute lymphoid leukemia | 0.000526722 | Multidrug-resistant tuberculosis without extensive drug resistance | 0.004051953 |
| 2021 | Low SDI | Lower extremity peripheral arterial disease | 0.000495787 | Drug-susceptible tuberculosis | 0.051235357 |
| 2021 | Low SDI | Subarachnoid hemorrhage | 0.000413667 | Chronic kidney disease due to glomerulonephritis | 0.007411207 |
| 2021 | Low SDI | Multidrug-resistant tuberculosis without extensive drug resistance | 0.000394152 | Hypertensive heart disease | 0.18427742 |
| 2021 | Low SDI | Chronic myeloid leukemia | 0.000388854 | Multiple myeloma | 0.000505573 |
| 2021 | Low SDI | Liver cancer due to other causes | 0.00037265 | Total cancers | 0.031181878 |
| 2021 | Low SDI | Multiple myeloma | 0.00030683 | Ischemic heart disease | 0.133304995 |
| 2021 | Low SDI | Liver cancer due to other causes | 0.000305795 | Other leukemia | 0.000550881 |
| 2021 | Low SDI | Chronic myeloid leukemia | 0.000279462 | Subarachnoid hemorrhage | 0.002067096 |
| 2021 | Low SDI | Burkitt lymphoma | 0.0000503 | Intracerebral hemorrhage | 0.02267359 |
| 2021 | Low SDI | Burkitt lymphoma | 0.0000423 | Breast cancer | 0.004242058 |

DALYs: disability-adjusted life years; ASMR: Age-standardized Mortality Rate; ASDR: Age-standardized DALYs Rate; SDI: Social Demographic Index.

**Supplementary table 4. Cases and age-standardised rate of AF/AFL deaths and their EAPCs from 1990 to 2021**

|  | No. 1990 | ASMR 1990 | No. 2021 | ASMR 2021 | EAPC (%) |
| --- | --- | --- | --- | --- | --- |
| Global | 5722 (2352-9912) | 0.21 (0.09-0.36) | 27237 (11747-46605) | 0.35 (0.15-0.6) | 1.65 (1.6-1.71) |
| Countries and territories |  |  |  |  |  |
| Afghanistan | 4 (1-9) | 0.07 (0.02-0.15) | 12 (4-23) | 0.19 (0.06-0.37) | 3.48 (3.3-3.67) |
| Albania | 5 (2-9) | 0.33 (0.13-0.63) | 22 (8-42) | 0.56 (0.22-1.1) | 2.05 (1.84-2.26) |
| Algeria | 12 (4-25) | 0.19 (0.06-0.44) | 144 (56-264) | 0.72 (0.29-1.3) | 5.17 (4.84-5.51) |
| American Samoa | 0 (0-0) | 0.54 (0.18-1.11) | 0 (0-1) | 1.03 (0.42-1.83) | 2.28 (2.21-2.35) |
| Andorra | 0 (0-0) | 0.25 (0.09-0.5) | 1 (0-1) | 0.29 (0.11-0.59) | 0.77 (0.6-0.94) |
| Angola | 0 (0-1) | 0.01 (0-0.03) | 8 (3-17) | 0.11 (0.03-0.24) | 7.33 (7.2-7.46) |
| Antigua and Barbuda | 0 (0-0) | 0.22 (0.08-0.45) | 0 (0-1) | 0.58 (0.23-1.1) | 3.21 (2.96-3.46) |
| Argentina | 68 (28-130) | 0.26 (0.1-0.5) | 254 (103-475) | 0.43 (0.17-0.8) | 2.4 (2-2.8) |
| Armenia | 3 (1-5) | 0.14 (0.06-0.25) | 12 (5-22) | 0.29 (0.12-0.51) | 2.68 (2.33-3.04) |
| Australia | 76 (30-150) | 0.44 (0.17-0.87) | 483 (197-897) | 0.87 (0.35-1.61) | 2.27 (1.96-2.58) |
| Austria | 44 (18-82) | 0.37 (0.15-0.7) | 150 (64-291) | 0.62 (0.26-1.19) | 1.78 (1.43-2.13) |
| Azerbaijan | 4 (2-8) | 0.11 (0.05-0.21) | 15 (6-26) | 0.21 (0.08-0.37) | 2.26 (2.04-2.47) |
| Bahamas | 0 (0-1) | 0.29 (0.11-0.55) | 2 (1-4) | 0.67 (0.26-1.29) | 2.64 (2.34-2.94) |
| Bahrain | 0 (0-1) | 0.44 (0.13-1) | 3 (1-5) | 0.96 (0.3-1.94) | 2.37 (1.92-2.82) |
| Bangladesh | 0 (-1-2) | 0 (0-0) | 36 (8-92) | 0.03 (0.01-0.09) | 15.37 (14.14-16.61) |
| Barbados | 1 (0-1) | 0.26 (0.09-0.5) | 3 (1-6) | 0.64 (0.27-1.22) | 2.94 (2.64-3.24) |
| Belarus | 33 (14-62) | 0.29 (0.12-0.53) | 91 (40-165) | 0.55 (0.24-0.99) | 1.83 (1.68-1.98) |
| Belgium | 36 (15-65) | 0.23 (0.1-0.43) | 132 (52-234) | 0.4 (0.16-0.71) | 2.1 (1.75-2.45) |
| Belize | 0 (0-1) | 0.27 (0.1-0.6) | 2 (1-3) | 0.65 (0.27-1.14) | 2.58 (1.94-3.22) |
| Benin | 1 (0-1) | 0.04 (0.01-0.1) | 4 (1-8) | 0.12 (0.04-0.25) | 3.59 (3.41-3.77) |
| Bermuda | 0 (0-0) | 0.4 (0.15-0.82) | 1 (0-2) | 0.58 (0.23-1.09) | 0.97 (0.88-1.07) |
| Bhutan | 0 (0-0) | 0.02 (0-0.05) | 0 (0-1) | 0.09 (0.03-0.19) | 5.42 (5.28-5.55) |
| Bolivia (Plurinational State of) | 3 (1-5) | 0.1 (0.03-0.2) | 26 (10-48) | 0.38 (0.15-0.69) | 4.65 (4.49-4.81) |
| Bosnia and Herzegovina | 6 (3-11) | 0.2 (0.08-0.37) | 31 (13-53) | 0.48 (0.21-0.83) | 2.77 (2.6-2.94) |
| Botswana | 0 (0-1) | 0.1 (0.03-0.21) | 3 (1-6) | 0.41 (0.16-0.74) | 5.55 (5.09-6.01) |
| Brazil | 112 (42-201) | 0.19 (0.07-0.35) | 1238 (497-2151) | 0.53 (0.21-0.92) | 3.43 (3.17-3.69) |
| Brunei Darussalam | 0 (0-0) | 0.03 (0.01-0.06) | 1 (0-1) | 0.15 (0.06-0.26) | 5.72 (5.41-6.02) |
| Bulgaria | 43 (17-73) | 0.57 (0.22-0.98) | 119 (51-211) | 0.82 (0.35-1.44) | 1.22 (1.06-1.38) |
| Burkina Faso | 0 (0-0) | 0 (0-0.01) | 1 (0-4) | 0.02 (0-0.06) | 7.76 (7.65-7.87) |
| Burundi | 0 (0-0) | 0 (0-0.02) | 1 (0-2) | 0.02 (0-0.06) | 4.7 (4.38-5.02) |
| Cabo Verde | 0 (0-0) | 0.05 (0.01-0.12) | 1 (0-3) | 0.33 (0.12-0.63) | 6.24 (5.94-6.55) |
| Cambodia | 0 (0-0) | 0 (0-0.01) | 2 (0-5) | 0.02 (0-0.06) | 7.66 (7.57-7.74) |
| Cameroon | 5 (2-9) | 0.19 (0.06-0.38) | 34 (14-60) | 0.53 (0.2-0.95) | 3.18 (3.13-3.24) |
| Canada | 121 (54-227) | 0.39 (0.17-0.74) | 453 (182-822) | 0.52 (0.21-0.94) | 0.57 (0.37-0.77) |
| Central African Republic | 0 (0-0) | 0.02 (0-0.05) | 1 (0-3) | 0.09 (0.03-0.21) | 5.49 (5.41-5.57) |
| Chad | 1 (0-2) | 0.04 (0.01-0.09) | 3 (1-6) | 0.09 (0.03-0.21) | 2.69 (2.64-2.74) |
| Chile | 20 (8-38) | 0.25 (0.1-0.47) | 133 (53-238) | 0.5 (0.2-0.89) | 3.45 (2.86-4.04) |
| China | 51 (10-116) | 0.01 (0-0.03) | 2378 (942-4212) | 0.15 (0.06-0.27) | 8.34 (8.12-8.56) |
| Colombia | 24 (9-43) | 0.19 (0.07-0.34) | 250 (109-459) | 0.43 (0.19-0.79) | 2.59 (2.46-2.72) |
| Comoros | 0 (0-0) | 0.03 (0.01-0.08) | 0 (0-1) | 0.12 (0.04-0.25) | 4.82 (4.64-5) |
| Congo | 0 (0-1) | 0.04 (0.01-0.08) | 5 (2-10) | 0.3 (0.1-0.6) | 6.62 (6.43-6.8) |
| Cook Islands | 0 (0-0) | 0.58 (0.24-1.16) | 0 (0-0) | 0.85 (0.34-1.52) | 1.26 (1.21-1.3) |
| Costa Rica | 3 (1-7) | 0.21 (0.08-0.42) | 27 (11-49) | 0.46 (0.19-0.84) | 2.29 (2.04-2.54) |
| Croatia | 11 (5-21) | 0.24 (0.1-0.44) | 49 (21-84) | 0.48 (0.2-0.82) | 2.47 (2.11-2.84) |
| Cuba | 13 (5-23) | 0.14 (0.05-0.26) | 90 (38-156) | 0.41 (0.17-0.7) | 3.6 (3.42-3.78) |
| Cyprus | 2 (1-4) | 0.46 (0.15-0.94) | 11 (4-19) | 0.72 (0.29-1.33) | 1.38 (1.16-1.6) |
| Czechia | 54 (22-103) | 0.43 (0.18-0.83) | 151 (61-271) | 0.63 (0.26-1.13) | 1.57 (1.46-1.68) |
| Côte d'Ivoire | 2 (1-3) | 0.1 (0.03-0.22) | 13 (5-24) | 0.24 (0.09-0.45) | 2.89 (2.77-3.01) |
| Democratic People's Republic of Korea | 1 (0-4) | 0.01 (0-0.05) | 57 (16-135) | 0.26 (0.06-0.62) | 10.21 (10.01-10.41) |
| Democratic Republic of the Congo | 3 (1-7) | 0.03 (0.01-0.08) | 47 (15-99) | 0.22 (0.07-0.47) | 6.43 (6.21-6.65) |
| Denmark | 21 (9-36) | 0.24 (0.1-0.4) | 68 (26-125) | 0.48 (0.18-0.87) | 2.31 (1.82-2.81) |
| Djibouti | 0 (0-0) | 0 (0-0.01) | 0 (0-0) | 0.02 (0-0.07) | 7.81 (7.5-8.12) |
| Dominica | 0 (0-1) | 0.67 (0.26-1.23) | 1 (0-1) | 1.17 (0.52-2.05) | 1.71 (1.58-1.85) |
| Dominican Republic | 2 (1-3) | 0.05 (0.02-0.11) | 22 (8-43) | 0.23 (0.08-0.45) | 5.08 (4.82-5.34) |
| Ecuador | 6 (2-12) | 0.14 (0.05-0.27) | 65 (25-123) | 0.46 (0.18-0.88) | 4.1 (3.87-4.33) |
| Egypt | 50 (20-90) | 0.42 (0.16-0.77) | 209 (93-351) | 0.73 (0.32-1.24) | 1.91 (1.78-2.05) |
| El Salvador | 8 (3-15) | 0.29 (0.11-0.54) | 50 (21-93) | 0.67 (0.28-1.26) | 2.61 (2.49-2.74) |
| Equatorial Guinea | 0 (0-0) | 0.06 (0.02-0.15) | 1 (1-3) | 0.47 (0.18-0.87) | 6.93 (6.82-7.03) |
| Eritrea | 0 (0-0) | 0.01 (0-0.03) | 1 (0-1) | 0.04 (0.01-0.09) | 5.02 (4.91-5.12) |
| Estonia | 7 (3-12) | 0.4 (0.17-0.69) | 25 (10-45) | 0.7 (0.29-1.26) | 1.75 (1.65-1.85) |
| Eswatini | 1 (0-1) | 0.44 (0.18-0.78) | 2 (1-4) | 0.79 (0.35-1.31) | 2.58 (2.18-2.97) |
| Ethiopia | 1 (0-3) | 0.01 (0-0.02) | 7 (3-15) | 0.02 (0.01-0.05) | 4.18 (3.99-4.38) |
| Fiji | 1 (0-2) | 0.43 (0.16-0.92) | 5 (2-8) | 1.13 (0.48-1.96) | 2.84 (2.5-3.18) |
| Finland | 38 (15-67) | 0.53 (0.21-0.96) | 83 (34-149) | 0.49 (0.21-0.88) | -0.57 (-0.95--0.18) |
| France | 223 (87-392) | 0.25 (0.1-0.44) | 907 (371-1584) | 0.43 (0.18-0.75) | 1.82 (1.64-2.01) |
| Gabon | 1 (0-2) | 0.2 (0.07-0.39) | 4 (2-8) | 0.64 (0.26-1.23) | 3.69 (3.55-3.83) |
| Gambia | 0 (0-0) | 0.06 (0.02-0.14) | 1 (0-2) | 0.2 (0.07-0.39) | 3.63 (3.53-3.72) |
| Georgia | 14 (6-28) | 0.28 (0.11-0.56) | 34 (15-59) | 0.51 (0.22-0.89) | 1.9 (1.04-2.76) |
| Germany | 691 (271-1271) | 0.53 (0.21-0.99) | 2071 (864-3980) | 0.8 (0.33-1.53) | 1.79 (1.59-1.99) |
| Ghana | 1 (0-3) | 0.04 (0.01-0.1) | 23 (9-43) | 0.28 (0.1-0.55) | 6.28 (6.02-6.55) |
| Greece | 31 (12-56) | 0.23 (0.09-0.42) | 147 (57-287) | 0.42 (0.16-0.82) | 1.85 (1.76-1.94) |
| Greenland | 0 (0-0) | 0.87 (0.36-1.59) | 0 (0-1) | 0.83 (0.33-1.47) | 0.12 (-0.01-0.26) |
| Grenada | 0 (0-0) | 0.12 (0.04-0.24) | 0 (0-1) | 0.48 (0.18-0.91) | 4.79 (4.49-5.08) |
| Guam | 0 (0-0) | 0.47 (0.17-1.01) | 1 (0-1) | 0.25 (0.1-0.46) | -1.16 (-1.63--0.69) |
| Guatemala | 5 (2-9) | 0.26 (0.1-0.56) | 35 (15-65) | 0.42 (0.18-0.77) | 1.63 (1.46-1.81) |
| Guinea | 1 (0-2) | 0.04 (0.01-0.11) | 5 (1-10) | 0.12 (0.04-0.26) | 3.5 (3.39-3.61) |
| Guinea-Bissau | 0 (0-0) | 0.04 (0.01-0.11) | 1 (0-1) | 0.15 (0.05-0.3) | 3.97 (3.89-4.05) |
| Guyana | 0 (0-1) | 0.13 (0.04-0.26) | 2 (1-3) | 0.39 (0.16-0.71) | 3.76 (3.45-4.07) |
| Haiti | 0 (0-1) | 0.02 (0-0.04) | 5 (2-10) | 0.09 (0.03-0.21) | 6.31 (6.15-6.46) |
| Honduras | 2 (1-4) | 0.16 (0.06-0.31) | 27 (11-52) | 0.64 (0.25-1.21) | 4.59 (4.21-4.98) |
| Hungary | 61 (25-104) | 0.49 (0.2-0.85) | 117 (52-202) | 0.53 (0.24-0.91) | 0.47 (0.33-0.61) |
| Iceland | 1 (1-2) | 0.41 (0.17-0.78) | 5 (2-10) | 0.72 (0.28-1.35) | 2.19 (1.99-2.4) |
| India | 26 (7-55) | 0.01 (0-0.02) | 675 (284-1274) | 0.08 (0.03-0.16) | 7.58 (7.38-7.78) |
| Indonesia | 2 (-1-7) | 0 (0-0.01) | 106 (33-203) | 0.07 (0.02-0.15) | 12.26 (11.52-13.01) |
| Iran (Islamic Republic of) | 20 (8-35) | 0.14 (0.05-0.27) | 274 (116-485) | 0.46 (0.19-0.81) | 3.73 (3.63-3.84) |
| Iraq | 26 (9-55) | 0.37 (0.13-0.79) | 99 (41-183) | 0.73 (0.3-1.37) | 1.65 (1.44-1.86) |
| Ireland | 13 (5-23) | 0.34 (0.13-0.61) | 47 (19-83) | 0.54 (0.22-0.95) | 1.65 (1.31-1.98) |
| Israel | 19 (8-33) | 0.46 (0.19-0.81) | 74 (32-134) | 0.5 (0.22-0.91) | 0.43 (0.25-0.61) |
| Italy | 167 (69-296) | 0.2 (0.08-0.36) | 888 (360-1681) | 0.41 (0.17-0.77) | 2.7 (2.55-2.86) |
| Jamaica | 3 (1-7) | 0.19 (0.07-0.37) | 20 (9-36) | 0.55 (0.25-0.99) | 3.59 (3.41-3.77) |
| Japan | 30 (9-67) | 0.02 (0.01-0.04) | 246 (84-508) | 0.04 (0.02-0.09) | 1.89 (1.53-2.25) |
| Jordan | 3 (1-5) | 0.34 (0.14-0.61) | 22 (10-39) | 0.56 (0.24-1.01) | 1.47 (1.18-1.77) |
| Kazakhstan | 23 (10-41) | 0.24 (0.1-0.44) | 51 (23-86) | 0.43 (0.19-0.74) | 1.32 (1.05-1.6) |
| Kenya | 1 (0-2) | 0.01 (0-0.03) | 14 (6-26) | 0.1 (0.04-0.18) | 6.72 (6.61-6.83) |
| Kiribati | 0 (0-0) | 0.12 (0.04-0.27) | 0 (0-0) | 0.37 (0.15-0.73) | 3.61 (3.53-3.68) |
| Kuwait | 1 (0-2) | 0.21 (0.08-0.44) | 13 (6-23) | 0.65 (0.29-1.14) | 4.1 (3.51-4.69) |
| Kyrgyzstan | 4 (1-7) | 0.16 (0.06-0.3) | 9 (4-15) | 0.26 (0.1-0.43) | 1.47 (1.29-1.66) |
| Lao People's Democratic Republic | 0 (0-0) | 0.01 (0-0.04) | 2 (1-5) | 0.07 (0.02-0.15) | 6.86 (6.67-7.06) |
| Latvia | 13 (5-24) | 0.38 (0.15-0.72) | 31 (13-55) | 0.62 (0.26-1.11) | 1.8 (1.62-1.98) |
| Lebanon | 6 (2-12) | 0.39 (0.13-0.82) | 37 (15-68) | 0.53 (0.22-0.97) | 0.73 (0.56-0.9) |
| Lesotho | 1 (0-2) | 0.15 (0.05-0.35) | 3 (1-5) | 0.43 (0.17-0.77) | 4.52 (3.98-5.07) |
| Liberia | 1 (0-1) | 0.08 (0.02-0.16) | 3 (1-6) | 0.26 (0.09-0.51) | 4.02 (3.87-4.17) |
| Libya | 2 (1-5) | 0.15 (0.05-0.3) | 21 (8-40) | 0.57 (0.21-1.09) | 4.89 (4.63-5.16) |
| Lithuania | 14 (6-25) | 0.34 (0.14-0.6) | 47 (20-85) | 0.66 (0.28-1.16) | 2.22 (2.08-2.37) |
| Luxembourg | 2 (1-4) | 0.49 (0.2-0.88) | 10 (4-17) | 0.73 (0.28-1.26) | 1.71 (1.44-1.98) |
| Madagascar | 1 (0-3) | 0.03 (0.01-0.08) | 8 (3-17) | 0.14 (0.04-0.31) | 4.47 (4.27-4.68) |
| Malawi | 0 (0-1) | 0.01 (0-0.05) | 4 (1-8) | 0.09 (0.03-0.2) | 6.08 (5.93-6.23) |
| Malaysia | 3 (1-7) | 0.04 (0.01-0.09) | 56 (22-96) | 0.28 (0.11-0.49) | 6.66 (6.28-7.04) |
| Maldives | 0 (0-0) | 0.01 (0-0.02) | 0 (0-0) | 0.04 (0.01-0.1) | 6.68 (6.54-6.81) |
| Mali | 1 (0-2) | 0.03 (0.01-0.08) | 3 (1-5) | 0.05 (0.01-0.11) | 1.52 (1.22-1.83) |
| Malta | 1 (0-1) | 0.22 (0.07-0.4) | 5 (2-10) | 0.45 (0.19-0.83) | 2.6 (2.32-2.88) |
| Marshall Islands | 0 (0-0) | 0.47 (0.17-1) | 0 (0-0) | 0.94 (0.38-1.78) | 2.09 (1.99-2.18) |
| Mauritania | 2 (1-4) | 0.31 (0.11-0.63) | 10 (4-17) | 0.71 (0.27-1.26) | 2.42 (2.31-2.52) |
| Mauritius | 1 (0-1) | 0.11 (0.04-0.22) | 5 (2-8) | 0.3 (0.12-0.53) | 2.95 (2.73-3.16) |
| Mexico | 83 (32-159) | 0.27 (0.1-0.52) | 642 (279-1139) | 0.59 (0.26-1.05) | 2.52 (2.38-2.67) |
| Micronesia (Federated States of) | 0 (0-0) | 0.4 (0.15-0.84) | 0 (0-1) | 0.96 (0.38-1.79) | 2.83 (2.81-2.85) |
| Monaco | 0 (0-1) | 0.36 (0.14-0.69) | 1 (0-1) | 0.49 (0.2-0.9) | 1.13 (0.89-1.36) |
| Mongolia | 1 (0-1) | 0.1 (0.04-0.18) | 2 (1-4) | 0.14 (0.06-0.25) | 0.57 (0.33-0.82) |
| Montenegro | 7 (3-12) | 1.26 (0.52-2.26) | 21 (9-38) | 2.72 (1.14-4.89) | 2.8 (2.38-3.22) |
| Morocco | 11 (4-23) | 0.1 (0.03-0.22) | 89 (35-168) | 0.36 (0.14-0.69) | 4.5 (4.34-4.67) |
| Mozambique | 0 (0-1) | 0.01 (0-0.03) | 6 (2-12) | 0.07 (0.02-0.16) | 7.71 (7.46-7.97) |
| Myanmar | 2 (0-4) | 0.01 (0-0.02) | 14 (4-32) | 0.03 (0.01-0.09) | 5.7 (5.46-5.94) |
| Namibia | 0 (0-1) | 0.13 (0.04-0.26) | 4 (2-7) | 0.5 (0.21-0.9) | 4.37 (4.06-4.68) |
| Nauru | 0 (0-0) | 0.55 (0.22-1.06) | 0 (0-0) | 1.34 (0.5-2.85) | 2.93 (2.81-3.05) |
| Nepal | 0 (0-1) | 0 (0-0) | 4 (1-9) | 0.02 (0-0.05) | 11.23 (11.05-11.4) |
| Netherlands | 76 (29-135) | 0.38 (0.14-0.68) | 203 (78-374) | 0.49 (0.19-0.9) | 0.54 (0.41-0.67) |
| New Zealand | 21 (9-36) | 0.59 (0.24-1.02) | 80 (33-147) | 0.84 (0.35-1.53) | 1.46 (1.29-1.63) |
| Nicaragua | 3 (1-5) | 0.21 (0.08-0.41) | 17 (7-31) | 0.44 (0.17-0.83) | 2.7 (2.42-2.97) |
| Niger | 0 (0-1) | 0.03 (0.01-0.09) | 2 (1-5) | 0.05 (0.01-0.12) | 0.97 (0.67-1.27) |
| Nigeria | 33 (12-62) | 0.13 (0.04-0.26) | 183 (76-319) | 0.38 (0.16-0.65) | 2.92 (2.71-3.13) |
| Niue | 0 (0-0) | 0.38 (0.14-0.77) | 0 (0-0) | 0.93 (0.37-1.69) | 2.83 (2.7-2.97) |
| North Macedonia | 5 (2-11) | 0.36 (0.15-0.72) | 20 (8-38) | 0.96 (0.42-1.79) | 2.99 (2.39-3.6) |
| Northern Mariana Islands | 0 (0-0) | 0.49 (0.19-0.99) | 0 (0-1) | 1.12 (0.48-1.96) | 2.63 (2.34-2.92) |
| Norway | 25 (10-42) | 0.32 (0.13-0.54) | 52 (22-89) | 0.4 (0.17-0.68) | 0.56 (0.31-0.81) |
| Oman | 1 (0-1) | 0.18 (0.07-0.33) | 7 (3-13) | 0.71 (0.3-1.29) | 5.22 (4.8-5.63) |
| Pakistan | 4 (0-10) | 0.01 (0-0.02) | 107 (42-205) | 0.13 (0.05-0.27) | 10.22 (9.52-10.92) |
| Palau | 0 (0-0) | 0.38 (0.14-0.78) | 0 (0-0) | 0.66 (0.27-1.23) | 1.88 (1.8-1.96) |
| Palestine | 3 (1-5) | 0.46 (0.18-0.94) | 12 (5-21) | 0.94 (0.39-1.6) | 2.18 (1.98-2.38) |
| Panama | 5 (2-10) | 0.42 (0.17-0.79) | 36 (15-63) | 0.76 (0.32-1.31) | 2.02 (1.89-2.15) |
| Papua New Guinea | 1 (0-1) | 0.02 (0.01-0.06) | 3 (1-7) | 0.06 (0.02-0.12) | 2.56 (2.49-2.63) |
| Paraguay | 3 (1-7) | 0.18 (0.07-0.38) | 27 (10-56) | 0.53 (0.2-1.09) | 4.01 (3.84-4.18) |
| Peru | 12 (4-24) | 0.11 (0.04-0.23) | 91 (36-167) | 0.27 (0.11-0.5) | 2.62 (2.36-2.88) |
| Philippines | 6 (2-12) | 0.03 (0.01-0.07) | 80 (32-151) | 0.14 (0.05-0.28) | 5 (4.66-5.35) |
| Poland | 234 (101-410) | 0.63 (0.27-1.1) | 500 (211-899) | 0.62 (0.26-1.11) | 0.28 (-0.27-0.83) |
| Portugal | 23 (8-44) | 0.2 (0.07-0.4) | 113 (47-207) | 0.33 (0.14-0.62) | 1.36 (1.08-1.63) |
| Puerto Rico | 13 (5-27) | 0.45 (0.16-0.93) | 51 (22-94) | 0.52 (0.22-0.94) | 0.11 (-0.08-0.29) |
| Qatar | 0 (0-1) | 0.71 (0.19-1.58) | 2 (1-4) | 0.88 (0.22-1.81) | -0.1 (-0.8-0.6) |
| Republic of Korea | 2 (0-4) | 0.01 (0-0.02) | 91 (29-198) | 0.1 (0.03-0.22) | 9.27 (8.88-9.66) |
| Republic of Moldova | 11 (5-20) | 0.39 (0.17-0.71) | 34 (15-59) | 0.56 (0.24-0.95) | 0.85 (0.7-1) |
| Romania | 60 (24-102) | 0.3 (0.12-0.52) | 187 (79-319) | 0.44 (0.19-0.75) | 0.77 (0.58-0.96) |
| Russian Federation | 473 (196-812) | 0.35 (0.15-0.61) | 1586 (672-2740) | 0.65 (0.28-1.12) | 1.99 (1.79-2.19) |
| Rwanda | 0 (0-1) | 0.01 (0-0.02) | 1 (0-3) | 0.03 (0-0.08) | 4.03 (3.55-4.51) |
| Saint Kitts and Nevis | 0 (0-0) | 0.29 (0.1-0.58) | 0 (0-1) | 0.75 (0.3-1.35) | 3.3 (3.01-3.6) |
| Saint Lucia | 0 (0-0) | 0.16 (0.05-0.36) | 1 (0-2) | 0.44 (0.16-0.83) | 2.43 (2.16-2.71) |
| Saint Vincent and the Grenadines | 0 (0-0) | 0.09 (0.03-0.24) | 0 (0-1) | 0.35 (0.13-0.65) | 4.61 (4.38-4.84) |
| Samoa | 0 (0-1) | 0.43 (0.15-0.87) | 1 (0-2) | 0.87 (0.33-1.7) | 2.27 (2.2-2.34) |
| San Marino | 0 (0-0) | 0.43 (0.17-0.81) | 0 (0-1) | 0.33 (0.12-0.64) | -0.06 (-0.44-0.33) |
| Sao Tome and Principe | 0 (0-0) | 0.07 (0.02-0.18) | 0 (0-0) | 0.35 (0.13-0.67) | 5.17 (4.91-5.43) |
| Saudi Arabia | 9 (4-16) | 0.24 (0.1-0.44) | 55 (24-95) | 0.7 (0.3-1.22) | 3.4 (3.27-3.53) |
| Senegal | 1 (0-3) | 0.07 (0.02-0.14) | 9 (4-17) | 0.19 (0.07-0.35) | 3.26 (3.14-3.38) |
| Serbia | 27 (11-52) | 0.37 (0.14-0.72) | 110 (46-196) | 0.62 (0.26-1.12) | 1.01 (0.65-1.37) |
| Seychelles | 0 (0-0) | 0.09 (0.03-0.19) | 0 (0-1) | 0.46 (0.17-0.84) | 5.75 (5.46-6.04) |
| Sierra Leone | 0 (0-1) | 0.03 (0.01-0.09) | 2 (1-5) | 0.1 (0.03-0.2) | 3.72 (3.58-3.85) |
| Singapore | 0 (0-0) | 0.01 (0-0.01) | 4 (1-8) | 0.05 (0.02-0.09) | 6.47 (5.93-7.01) |
| Slovakia | 38 (16-66) | 0.7 (0.3-1.24) | 84 (38-145) | 0.89 (0.4-1.54) | 0.98 (0.87-1.08) |
| Slovenia | 9 (4-17) | 0.4 (0.16-0.7) | 31 (13-53) | 0.53 (0.23-0.91) | 1.26 (0.84-1.68) |
| Solomon Islands | 0 (0-0) | 0.06 (0.02-0.14) | 1 (0-1) | 0.17 (0.05-0.38) | 3.74 (3.63-3.86) |
| Somalia | 0 (0-0) | 0.01 (0-0.02) | 1 (0-3) | 0.02 (0-0.06) | 4.16 (3.99-4.33) |
| South Africa | 33 (14-58) | 0.21 (0.09-0.38) | 184 (84-308) | 0.6 (0.27-1) | 3.27 (2.84-3.71) |
| South Sudan | 0 (0-0) | 0 (0-0.01) | 0 (0-1) | 0.01 (0-0.03) | 9 (8.64-9.35) |
| Spain | 205 (83-380) | 0.4 (0.16-0.75) | 830 (328-1526) | 0.56 (0.23-1.04) | 1.02 (0.88-1.15) |
| Sri Lanka | 2 (1-5) | 0.03 (0.01-0.07) | 33 (11-71) | 0.16 (0.05-0.36) | 5.98 (5.71-6.26) |
| Sudan | 9 (3-18) | 0.15 (0.05-0.31) | 49 (18-86) | 0.38 (0.14-0.68) | 2.99 (2.83-3.15) |
| Suriname | 0 (0-0) | 0.07 (0.02-0.16) | 1 (0-3) | 0.24 (0.08-0.48) | 4.25 (4.08-4.41) |
| Sweden | 46 (19-86) | 0.27 (0.11-0.5) | 227 (90-423) | 0.76 (0.31-1.41) | 3.7 (3.38-4.02) |
| Switzerland | 19 (8-35) | 0.17 (0.07-0.3) | 62 (25-113) | 0.24 (0.1-0.43) | 1.53 (1.39-1.67) |
| Syrian Arab Republic | 13 (5-24) | 0.34 (0.13-0.65) | 65 (26-116) | 0.87 (0.35-1.6) | 2.65 (2.42-2.89) |
| Taiwan (Province of China) | 6 (2-14) | 0.06 (0.02-0.17) | 109 (43-198) | 0.23 (0.09-0.42) | 3.74 (3.17-4.31) |
| Tajikistan | 2 (1-4) | 0.09 (0.04-0.18) | 4 (2-8) | 0.12 (0.05-0.21) | 0.41 (-0.02-0.83) |
| Thailand | 6 (1-16) | 0.02 (0-0.07) | 184 (55-387) | 0.17 (0.05-0.35) | 6.49 (6.18-6.8) |
| Timor-Leste | 0 (0-0) | 0 (-0.01-0) | 0 (0-0) | 0 (0-0.01) | 84.6 (41.8-140.31) |
| Togo | 0 (0-1) | 0.06 (0.02-0.13) | 4 (2-8) | 0.23 (0.09-0.47) | 4.28 (4.22-4.35) |
| Tokelau | 0 (0-0) | 0.36 (0.13-0.75) | 0 (0-0) | 0.86 (0.35-1.64) | 2.82 (2.67-2.96) |
| Tonga | 0 (0-0) | 0.33 (0.13-0.67) | 1 (0-1) | 0.81 (0.33-1.45) | 3.01 (2.81-3.2) |
| Trinidad and Tobago | 2 (1-3) | 0.36 (0.15-0.67) | 10 (4-17) | 0.55 (0.24-0.99) | 1.34 (1.24-1.44) |
| Tunisia | 3 (1-7) | 0.1 (0.03-0.22) | 56 (21-105) | 0.54 (0.2-1.06) | 5.65 (5.39-5.92) |
| Turkmenistan | 2 (1-3) | 0.14 (0.05-0.25) | 8 (3-15) | 0.26 (0.1-0.48) | 1.53 (1.29-1.78) |
| Tuvalu | 0 (0-0) | 0.28 (0.11-0.56) | 0 (0-0) | 0.74 (0.3-1.47) | 3.27 (3.23-3.3) |
| Türkiye | 73 (29-131) | 0.3 (0.12-0.56) | 459 (191-800) | 0.6 (0.25-1.05) | 2.66 (2.06-3.27) |
| Uganda | 0 (0-1) | 0 (0-0.02) | 4 (1-9) | 0.04 (0.01-0.09) | 6.63 (6.43-6.84) |
| Ukraine | 233 (101-387) | 0.4 (0.18-0.69) | 469 (194-813) | 0.59 (0.25-1.03) | 0.82 (0.54-1.1) |
| United Arab Emirates | 1 (0-1) | 0.23 (0.09-0.44) | 7 (3-12) | 0.77 (0.31-1.39) | 6.69 (5.6-7.79) |
| United Kingdom | 366 (145-688) | 0.39 (0.16-0.73) | 1053 (418-1955) | 0.65 (0.26-1.2) | 1.76 (1.66-1.86) |
| United Republic of Tanzania | 3 (1-7) | 0.05 (0.01-0.11) | 33 (11-64) | 0.19 (0.07-0.38) | 4.63 (4.48-4.78) |
| United States of America | 1119 (449-2165) | 0.33 (0.13-0.65) | 5278 (2228-9082) | 0.79 (0.34-1.36) | 2.7 (2.55-2.85) |
| United States Virgin Islands | 0 (0-1) | 0.8 (0.3-1.53) | 1 (0-2) | 0.71 (0.29-1.3) | -0.21 (-0.41-0) |
| Uruguay | 8 (4-15) | 0.23 (0.1-0.41) | 30 (13-56) | 0.42 (0.17-0.77) | 2.18 (2-2.35) |
| Uzbekistan | 7 (2-16) | 0.07 (0.02-0.17) | 27 (11-50) | 0.15 (0.06-0.28) | 2.56 (2.37-2.76) |
| Vanuatu | 0 (0-0) | 0.08 (0.03-0.2) | 0 (0-1) | 0.24 (0.09-0.49) | 3.4 (3.35-3.45) |
| Venezuela (Bolivarian Republic of) | 23 (10-45) | 0.31 (0.13-0.63) | 159 (66-304) | 0.6 (0.25-1.15) | 2 (1.87-2.13) |
| Viet Nam | 0 (-1-1) | 0 (0-0) | 25 (4-65) | 0.03 (0.01-0.1) | 20.81 (17.71-23.99) |
| Yemen | 2 (0-4) | 0.06 (0.01-0.13) | 24 (9-45) | 0.29 (0.1-0.57) | 5.25 (4.92-5.59) |
| Zambia | 0 (0-1) | 0.02 (0-0.05) | 9 (3-21) | 0.19 (0.06-0.43) | 7.77 (7.53-8) |
| Zimbabwe | 1 (0-2) | 0.05 (0.01-0.1) | 10 (4-19) | 0.24 (0.09-0.44) | 5.81 (5.31-6.32) |
| SDI | | | | | |
|  | No. 1990 | ASMR 1990 | No. 2021 | ASMR 2021 | EAPC (%) |
| High SDI | 3342 (1379-5954) | 0.31 (0.13-0.56) | 13036 (5608-22825) | 0.49 (0.21-0.86) | 1.5 (1.41-1.6) |
| High-middle SDI | 1704 (712-2821) | 0.25 (0.1-0.41) | 7209 (3108-12447) | 0.39 (0.17-0.67) | 1.47 (1.41-1.53) |
| Middle SDI | 436 (173-749) | 0.08 (0.03-0.13) | 4907 (2166-8209) | 0.24 (0.11-0.41) | 3.73 (3.6-3.85) |
| Low-middle SDI | 197 (77-350) | 0.06 (0.02-0.1) | 1782 (757-2955) | 0.18 (0.08-0.3) | 4.03 (3.93-4.14) |
| Low SDI | 30 (9-63) | 0.02 (0.01-0.04) | 265 (105-485) | 0.08 (0.03-0.15) | 4.49 (4.23-4.75) |
| Gender and Age | | | | | |
|  | No. 1990 | ASMR 1990 | No. 2021 | ASMR 2021 | EAPC (%) |
| Sex |  |  |  |  |  |
| Female | 4194 (1752-7181) | 0.25 (0.1-0.42) | 18059 (7717-30760) | 0.38 (0.16-0.65) | 1.37 (1.31-1.43) |
| Male | 1528 (617-2659) | 0.15 (0.06-0.25) | 9178 (3976-16050) | 0.3 (0.13-0.53) | 2.53 (2.46-2.61) |
| Age |  |  |  |  |  |
| 30-34 years | 2 (1-3) | 0 (0-0) | 8 (4-13) | 0 (0-0) | 3.23 (3.05-3.4) |
| 35-39 years | 4 (2-6) | 0 (0-0) | 15 (7-24) | 0 (0-0) | 3.02 (2.94-3.1) |
| 40-44 years | 11 (5-19) | 0 (0-0.01) | 44 (20-71) | 0.01 (0-0.01) | 2.57 (2.45-2.69) |
| 45-49 years | 26 (11-46) | 0.01 (0-0.02) | 108 (49-175) | 0.02 (0.01-0.04) | 2.17 (2.03-2.32) |
| 50-54 years | 62 (26-110) | 0.03 (0.01-0.05) | 242 (109-396) | 0.05 (0.02-0.09) | 2.02 (1.87-2.18) |
| 55-59 years | 123 (51-212) | 0.07 (0.03-0.11) | 484 (220-787) | 0.12 (0.06-0.2) | 2.08 (1.96-2.19) |
| 60-64 years | 235 (100-410) | 0.15 (0.06-0.26) | 814 (365-1322) | 0.25 (0.11-0.41) | 1.84 (1.79-1.9) |
| 65-69 years | 351 (149-634) | 0.28 (0.12-0.51) | 1275 (576-2083) | 0.46 (0.21-0.76) | 1.57 (1.5-1.64) |
| 70-74 years | 518 (214-912) | 0.61 (0.25-1.08) | 2219 (983-3693) | 1.08 (0.48-1.79) | 1.6 (1.47-1.73) |
| 75-79 years | 917 (382-1622) | 1.49 (0.62-2.63) | 2990 (1324-4937) | 2.27 (1-3.74) | 1.55 (1.43-1.67) |
| 80-84 years | 1182 (488-1987) | 3.34 (1.38-5.62) | 4712 (2016-8091) | 5.38 (2.3-9.24) | 1.54 (1.42-1.66) |
| 85-89 years | 1166 (469-1970) | 7.72 (3.1-13.04) | 5817 (2455-10148) | 12.72 (5.37-22.19) | 1.75 (1.65-1.85) |
| 90-94 years | 820 (332-1396) | 19.15 (7.74-32.58) | 5773 (2424-10127) | 32.27 (13.55-56.61) | 1.71 (1.62-1.8) |
| 95+ years | 304 (124-531) | 29.88 (12.2-52.13) | 2736 (1120-4774) | 50.2 (20.55-87.59) | 1.52 (1.39-1.65) |

AF/AFL: Atrial Fibrillation and Flutter, ASMR: Age-standardized Mortality Rate, EAPC: Estimated Annual Percentage Changes. SDI: Social Demographic Index.

**Supplementary table 5. Cases and age-standardised of AF/AFL DALYs and their EAPCs from 1990 to 2021**

|  | No. 1990 | ASDR 1990 | No. 2021 | ASDR 2021 | EAPC (%) |
| --- | --- | --- | --- | --- | --- |
| Global | 175032 (67999-298165) | 5.19 (2.02-8.75) | 724574 (303525-1246374) | 8.71 (3.65-15.06) | 1.64 (1.6-1.69) |
| Countries and territories |  |  |  |  |  |
| Afghanistan | 151 (52-299) | 2.19 (0.75-4.33) | 413 (161-739) | 5.14 (1.98-9.27) | 2.79 (2.67-2.92) |
| Albania | 141 (52-265) | 8.08 (2.97-15.28) | 525 (203-1043) | 12.2 (4.7-24.34) | 1.44 (1.33-1.56) |
| Algeria | 368 (137-709) | 3.91 (1.33-7.79) | 3222 (1326-5946) | 12.19 (5.16-22.43) | 4.12 (3.96-4.28) |
| American Samoa | 4 (2-6) | 16.94 (7.04-29.44) | 13 (6-21) | 28.01 (12.58-47.89) | 1.7 (1.65-1.75) |
| Andorra | 4 (1-7) | 7.2 (2.58-13.63) | 14 (5-28) | 8.74 (3.21-17.02) | 0.73 (0.64-0.82) |
| Angola | 19 (5-41) | 0.45 (0.08-1.1) | 326 (123-610) | 3.09 (1.13-5.88) | 6.17 (6.08-6.26) |
| Antigua and Barbuda | 3 (1-6) | 6.06 (2.33-10.86) | 13 (5-23) | 13.34 (5.18-24.36) | 2.58 (2.45-2.72) |
| Argentina | 2005 (782-3631) | 6.64 (2.56-12.02) | 5542 (2208-10136) | 9.6 (3.83-17.48) | 1.67 (1.42-1.91) |
| Armenia | 150 (58-273) | 6.07 (2.36-11.04) | 424 (176-759) | 9.66 (3.97-17.33) | 1.65 (1.49-1.81) |
| Australia | 2125 (828-4052) | 11.22 (4.43-21.52) | 10664 (4621-18755) | 21.73 (9.39-38.12) | 2.23 (2.03-2.42) |
| Austria | 997 (394-1806) | 8.15 (3.19-14.8) | 3404 (1392-6439) | 16.34 (6.57-30.83) | 2.46 (2.23-2.68) |
| Azerbaijan | 233 (92-428) | 5.18 (2.05-9.43) | 788 (312-1454) | 8.6 (3.37-15.5) | 1.73 (1.62-1.84) |
| Bahamas | 12 (5-22) | 8.55 (3.35-15.37) | 64 (26-116) | 17.04 (6.75-31.29) | 2.22 (2.07-2.37) |
| Bahrain | 9 (3-19) | 8.17 (2.89-16.99) | 89 (37-162) | 16.9 (6.33-32.41) | 2.14 (1.86-2.41) |
| Bangladesh | 19 (-21-72) | 0.04 (-0.06-0.16) | 1386 (462-2903) | 1.06 (0.33-2.31) | 11.93 (11.43-12.42) |
| Barbados | 21 (8-38) | 7.22 (2.7-12.98) | 83 (34-151) | 15.98 (6.45-28.97) | 2.54 (2.36-2.73) |
| Belarus | 991 (402-1814) | 7.86 (3.2-14.26) | 2409 (1008-4180) | 14.62 (6.11-25.45) | 1.86 (1.73-2) |
| Belgium | 955 (390-1682) | 6.05 (2.49-10.64) | 2684 (1080-4624) | 10.03 (4.05-17.44) | 1.72 (1.5-1.94) |
| Belize | 8 (3-16) | 9.03 (3.3-17.4) | 47 (21-81) | 17.36 (7.57-29.9) | 2.01 (1.66-2.38) |
| Benin | 31 (13-56) | 1.64 (0.65-2.9) | 167 (65-295) | 3.66 (1.42-6.5) | 2.6 (2.48-2.72) |
| Bermuda | 6 (2-11) | 9.87 (3.68-19.32) | 24 (10-43) | 16.02 (6.63-29.42) | 1.45 (1.4-1.5) |
| Bhutan | 3 (1-5) | 1.02 (0.35-2.04) | 19 (7-35) | 3.25 (1.17-5.82) | 4.01 (3.94-4.08) |
| Bolivia (Plurinational State of) | 106 (37-207) | 3.39 (1.18-6.56) | 860 (346-1512) | 10.25 (4.13-18.21) | 3.64 (3.5-3.77) |
| Bosnia and Herzegovina | 243 (93-441) | 6.64 (2.52-11.93) | 788 (333-1358) | 12.25 (5.16-21.05) | 1.9 (1.77-2.03) |
| Botswana | 15 (6-30) | 3.14 (1.2-5.98) | 111 (45-189) | 9.87 (3.93-16.74) | 4.09 (3.84-4.33) |
| Brazil | 5024 (1930-9138) | 6.21 (2.36-11.29) | 35706 (14339-63076) | 14.6 (5.86-25.86) | 2.8 (2.64-2.95) |
| Brunei Darussalam | 2 (1-4) | 1.5 (0.51-2.93) | 25 (10-43) | 6.22 (2.48-10.46) | 4.75 (4.44-5.05) |
| Bulgaria | 1396 (562-2392) | 13.03 (5.09-22.35) | 2729 (1142-4882) | 18.25 (7.66-32.61) | 1.07 (0.99-1.16) |
| Burkina Faso | 6 (0-17) | 0.12 (-0.03-0.4) | 50 (11-119) | 0.57 (0.1-1.44) | 5.27 (5.12-5.42) |
| Burundi | 4 (0-13) | 0.19 (-0.01-0.55) | 30 (8-64) | 0.69 (0.18-1.52) | 3.74 (3.51-3.96) |
| Cabo Verde | 3 (1-7) | 1.53 (0.52-2.89) | 27 (11-48) | 6.52 (2.63-11.29) | 4.67 (4.5-4.85) |
| Cambodia | 10 (1-25) | 0.19 (-0.01-0.5) | 115 (35-215) | 0.91 (0.25-1.86) | 5.24 (5.17-5.3) |
| Cameroon | 150 (56-269) | 4.2 (1.56-7.57) | 973 (413-1779) | 10.62 (4.45-18.59) | 2.96 (2.9-3.01) |
| Canada | 4292 (1710-8134) | 13.17 (5.25-24.95) | 12932 (5263-23457) | 16.63 (6.86-30.37) | 0.65 (0.54-0.77) |
| Central African Republic | 7 (2-17) | 0.61 (0.13-1.58) | 55 (20-112) | 2.71 (0.97-5.76) | 5 (4.93-5.07) |
| Chad | 28 (9-55) | 1.12 (0.35-2.17) | 104 (38-195) | 2.26 (0.75-4.45) | 2.23 (2.18-2.28) |
| Chile | 664 (256-1235) | 7.16 (2.74-13.16) | 3255 (1342-5785) | 12.42 (5.12-22.08) | 2.32 (1.97-2.67) |
| China | 2095 (478-4445) | 0.3 (0.06-0.68) | 75252 (29280-129907) | 3.84 (1.51-6.57) | 8.58 (8.42-8.75) |
| Colombia | 795 (314-1387) | 5.14 (2.03-9.07) | 6576 (2605-11806) | 11.76 (4.67-21.09) | 2.61 (2.53-2.69) |
| Comoros | 1 (0-3) | 0.76 (0.2-1.71) | 16 (6-29) | 3.59 (1.36-6.63) | 5.06 (5-5.12) |
| Congo | 17 (6-34) | 1.44 (0.49-2.81) | 169 (70-298) | 7.33 (2.81-13.57) | 4.98 (4.77-5.18) |
| Cook Islands | 2 (1-4) | 17.19 (6.97-31.25) | 6 (3-11) | 23.84 (10.06-41.88) | 1.12 (1.08-1.16) |
| Costa Rica | 114 (45-219) | 6.87 (2.71-13.3) | 738 (292-1308) | 13.23 (5.25-23.5) | 1.96 (1.84-2.08) |
| Croatia | 351 (139-634) | 6.27 (2.44-11.17) | 1068 (454-1867) | 11 (4.62-19.29) | 2.21 (1.97-2.45) |
| Cuba | 447 (175-750) | 4.49 (1.77-7.58) | 2249 (902-3779) | 10.94 (4.43-18.41) | 2.98 (2.89-3.06) |
| Cyprus | 51 (21-92) | 7.97 (2.92-15.17) | 210 (84-384) | 11.66 (4.78-21.2) | 1.21 (1.08-1.34) |
| Czechia | 1728 (695-3109) | 12.57 (5.04-22.58) | 4585 (1865-8303) | 19.72 (8.02-35.59) | 1.72 (1.51-1.94) |
| Côte d'Ivoire | 62 (24-110) | 2.17 (0.83-4.04) | 432 (174-742) | 5.2 (2.19-9.01) | 2.87 (2.78-2.95) |
| Democratic People's Republic of Korea | 21 (-2-71) | 0.23 (-0.01-0.81) | 1090 (350-2340) | 4.2 (1.22-9.43) | 10 (9.83-10.17) |
| Democratic Republic of the Congo | 124 (36-255) | 0.91 (0.25-2.03) | 1510 (530-2879) | 5.27 (1.87-10.04) | 5.81 (5.65-5.96) |
| Denmark | 589 (239-984) | 6.94 (2.78-11.63) | 1564 (594-2783) | 12.25 (4.7-21.71) | 1.7 (1.38-2.02) |
| Djibouti | 0 (0-1) | 0.11 (-0.04-0.41) | 5 (1-10) | 0.78 (0.17-1.72) | 6.17 (6.04-6.3) |
| Dominica | 8 (3-15) | 14.62 (5.95-26.15) | 19 (8-34) | 24.69 (10.88-42.85) | 1.65 (1.57-1.73) |
| Dominican Republic | 84 (29-159) | 2.29 (0.79-4.41) | 795 (301-1425) | 8.04 (3.04-14.45) | 4.15 (3.96-4.34) |
| Ecuador | 245 (88-471) | 4.85 (1.72-9.2) | 2078 (847-3642) | 13.27 (5.36-23.26) | 3.4 (3.28-3.53) |
| Egypt | 1438 (589-2551) | 8.08 (3.15-14.29) | 6842 (3074-11425) | 16.07 (7.06-26.75) | 2.25 (2.18-2.32) |
| El Salvador | 237 (88-435) | 8.3 (3.08-15.23) | 1096 (473-1978) | 16.58 (7.2-29.86) | 2.17 (2.1-2.25) |
| Equatorial Guinea | 4 (1-8) | 2.02 (0.69-4.06) | 42 (16-73) | 10.6 (3.89-18.41) | 5.51 (5.44-5.58) |
| Eritrea | 3 (1-7) | 0.3 (0.07-0.65) | 28 (10-53) | 1.13 (0.38-2.27) | 4.32 (4.2-4.45) |
| Estonia | 208 (85-357) | 10.44 (4.26-17.74) | 495 (204-899) | 16.33 (6.87-29.65) | 1.46 (1.4-1.52) |
| Eswatini | 19 (8-32) | 9.47 (3.85-16.14) | 75 (33-124) | 18.71 (8.09-30.59) | 2.54 (2.24-2.84) |
| Ethiopia | 62 (16-133) | 0.28 (0.06-0.61) | 388 (143-746) | 0.92 (0.33-1.76) | 3.82 (3.68-3.96) |
| Fiji | 39 (16-72) | 12.22 (4.69-23.64) | 165 (76-273) | 26.44 (11.98-44.22) | 2.4 (2.22-2.58) |
| Finland | 992 (394-1749) | 13.66 (5.42-24.16) | 1967 (782-3511) | 13.77 (5.52-25.1) | -0.23 (-0.44--0.02) |
| France | 5517 (2209-9521) | 6.31 (2.57-10.81) | 17684 (6785-30575) | 10.61 (4.08-18.58) | 1.68 (1.54-1.82) |
| Gabon | 26 (11-46) | 4.94 (1.89-8.68) | 117 (48-211) | 13.68 (5.7-24.17) | 3.16 (3.03-3.29) |
| Gambia | 6 (2-11) | 1.95 (0.67-3.58) | 38 (15-65) | 4.62 (1.76-8.15) | 2.72 (2.64-2.79) |
| Georgia | 485 (189-851) | 8.18 (3.16-14.63) | 839 (356-1428) | 13.32 (5.63-22.84) | 1.52 (1.12-1.93) |
| Germany | 17335 (6612-30680) | 13.2 (5.01-23.39) | 41697 (16784-76351) | 19.02 (7.63-34.65) | 1.38 (1.21-1.55) |
| Ghana | 54 (17-110) | 1 (0.27-2.19) | 745 (288-1268) | 6.04 (2.22-10.96) | 5.62 (5.44-5.8) |
| Greece | 944 (374-1661) | 6.31 (2.5-11.24) | 3204 (1267-6114) | 11.56 (4.54-21.8) | 1.74 (1.53-1.96) |
| Greenland | 6 (2-11) | 22.19 (8.87-39.6) | 13 (6-24) | 22.01 (9.17-39.28) | 0.16 (0.06-0.27) |
| Grenada | 3 (1-5) | 4.28 (1.62-7.5) | 12 (5-21) | 11.74 (4.73-20.96) | 3.36 (3.22-3.49) |
| Guam | 7 (3-14) | 11.17 (4.04-21.81) | 26 (11-47) | 12.15 (5.06-22.07) | 0.62 (0.41-0.82) |
| Guatemala | 190 (74-334) | 6.62 (2.64-12.18) | 1160 (487-2095) | 11.46 (4.81-20.61) | 1.85 (1.73-1.96) |
| Guinea | 37 (12-72) | 1.2 (0.39-2.49) | 138 (52-254) | 2.87 (1.04-5.38) | 2.81 (2.71-2.91) |
| Guinea-Bissau | 4 (2-9) | 1.24 (0.38-2.53) | 19 (8-33) | 3.48 (1.41-6.17) | 3.35 (3.3-3.4) |
| Guyana | 16 (6-29) | 4.54 (1.73-8.07) | 62 (25-111) | 10.6 (4.28-18.83) | 2.88 (2.76-3) |
| Haiti | 21 (4-44) | 0.62 (0.08-1.4) | 193 (79-360) | 2.9 (1.12-5.39) | 5.49 (5.36-5.62) |
| Honduras | 97 (36-175) | 5.24 (1.95-9.56) | 784 (306-1433) | 14.43 (5.8-26.84) | 3.37 (3.2-3.55) |
| Hungary | 1964 (779-3402) | 13.69 (5.38-23.83) | 3150 (1329-5492) | 15.09 (6.45-26.11) | 0.4 (0.34-0.47) |
| Iceland | 31 (12-59) | 10.34 (4.11-19.58) | 104 (41-192) | 16.19 (6.43-30.32) | 1.56 (1.44-1.68) |
| India | 1352 (429-2669) | 0.33 (0.1-0.66) | 24727 (9521-43934) | 2.32 (0.88-4.19) | 6.75 (6.59-6.91) |
| Indonesia | 213 (11-522) | 0.17 (-0.02-0.48) | 5364 (1974-9863) | 2.32 (0.77-4.42) | 8.93 (8.46-9.4) |
| Iran (Islamic Republic of) | 707 (277-1173) | 3.5 (1.35-5.97) | 7061 (2942-12081) | 10.54 (4.38-18.06) | 3.49 (3.42-3.57) |
| Iraq | 684 (252-1356) | 9.25 (3.35-18.46) | 2602 (1105-4655) | 14.47 (6.11-26.28) | 1.14 (1.03-1.25) |
| Ireland | 343 (137-589) | 8.52 (3.36-14.72) | 1032 (409-1857) | 12.38 (4.91-22.3) | 1.06 (0.85-1.27) |
| Israel | 529 (220-926) | 11.19 (4.68-19.63) | 2133 (907-3791) | 16.18 (6.91-28.94) | 1.46 (1.25-1.68) |
| Italy | 5842 (2242-10398) | 6.48 (2.48-11.51) | 19002 (7515-35242) | 10.76 (4.26-19.93) | 1.57 (1.54-1.61) |
| Jamaica | 105 (41-189) | 5.84 (2.31-10.53) | 479 (209-820) | 14.66 (6.38-25.04) | 3.08 (2.98-3.18) |
| Japan | 1155 (325-2492) | 0.69 (0.2-1.5) | 5415 (1909-10251) | 1.39 (0.51-2.62) | 1.94 (1.64-2.24) |
| Jordan | 86 (35-151) | 8.19 (3.31-14.4) | 766 (345-1307) | 13.62 (6.1-23.16) | 1.48 (1.28-1.68) |
| Kazakhstan | 853 (355-1493) | 7.69 (3.18-13.48) | 1852 (775-3282) | 12.14 (5.13-21.28) | 1.19 (1.06-1.33) |
| Kenya | 54 (18-103) | 0.66 (0.21-1.29) | 697 (289-1203) | 3.28 (1.3-5.66) | 5.39 (5.35-5.43) |
| Kiribati | 2 (1-5) | 6.12 (2.36-12.02) | 9 (4-16) | 13.2 (5.43-23.16) | 2.49 (2.43-2.56) |
| Kuwait | 30 (12-56) | 5.97 (2.32-11.35) | 368 (172-625) | 15.45 (7-25.78) | 3.32 (2.99-3.65) |
| Kyrgyzstan | 161 (66-281) | 5.84 (2.39-10.1) | 385 (153-671) | 9 (3.6-15.5) | 1.31 (1.21-1.4) |
| Lao People's Democratic Republic | 7 (1-17) | 0.36 (0.04-0.98) | 95 (34-183) | 2.22 (0.74-4.24) | 6.38 (6.1-6.66) |
| Latvia | 347 (138-627) | 9.84 (3.94-17.85) | 683 (282-1196) | 15.62 (6.49-27.61) | 1.74 (1.65-1.82) |
| Lebanon | 149 (56-290) | 8.06 (2.98-15.6) | 743 (301-1357) | 11.43 (4.62-20.81) | 0.93 (0.77-1.09) |
| Lesotho | 33 (13-61) | 4.46 (1.73-8.45) | 91 (40-161) | 10.49 (4.67-18.52) | 3.35 (3.05-3.66) |
| Liberia | 27 (11-46) | 2.52 (0.97-4.29) | 115 (47-204) | 6.57 (2.55-11.76) | 3.08 (2.99-3.17) |
| Libya | 74 (27-133) | 4.33 (1.59-7.83) | 600 (235-1088) | 13.72 (5.32-25.06) | 4.02 (3.88-4.16) |
| Lithuania | 393 (158-707) | 8.84 (3.55-15.81) | 1034 (434-1846) | 16.13 (6.69-28.91) | 1.99 (1.87-2.11) |
| Luxembourg | 59 (23-102) | 10.91 (4.34-18.87) | 182 (72-317) | 15.51 (6.19-26.92) | 1.35 (1.2-1.5) |
| Madagascar | 36 (10-76) | 0.82 (0.22-1.76) | 284 (106-540) | 3.27 (1.22-6.11) | 4.41 (4.26-4.56) |
| Malawi | 9 (2-21) | 0.33 (0.05-0.93) | 140 (52-260) | 2.38 (0.88-4.52) | 6.41 (6.29-6.52) |
| Malaysia | 192 (73-344) | 2.07 (0.77-3.77) | 1916 (805-3134) | 7.53 (3.09-12.44) | 4.3 (4.08-4.52) |
| Maldives | 1 (0-2) | 0.65 (0.18-1.39) | 10 (4-19) | 2.79 (1.04-5.03) | 4.85 (4.77-4.93) |
| Mali | 31 (10-62) | 0.92 (0.25-1.86) | 118 (42-214) | 1.55 (0.53-2.74) | 1.64 (1.41-1.87) |
| Malta | 23 (9-40) | 5.49 (2.12-9.95) | 114 (47-205) | 10.47 (4.34-18.92) | 2.31 (2.15-2.47) |
| Marshall Islands | 2 (1-4) | 13.43 (5.25-25.24) | 8 (3-14) | 23.95 (10.22-43.05) | 1.77 (1.69-1.85) |
| Mauritania | 48 (19-85) | 6.13 (2.36-11.15) | 205 (81-351) | 12.45 (4.77-21.59) | 2.1 (2.04-2.17) |
| Mauritius | 23 (9-39) | 3.41 (1.35-6.04) | 138 (56-237) | 7.84 (3.17-13.41) | 2.49 (2.3-2.67) |
| Mexico | 3125 (1209-5904) | 8.15 (3.18-15.35) | 19766 (8322-35974) | 16.54 (6.95-29.97) | 2.28 (2.22-2.35) |
| Micronesia (Federated States of) | 7 (3-12) | 13.66 (5.29-25.02) | 18 (7-30) | 25.2 (10.19-43.2) | 1.98 (1.96-2) |
| Monaco | 8 (3-15) | 10.35 (3.91-19.33) | 15 (6-27) | 13.05 (5.06-24.52) | 0.73 (0.63-0.84) |
| Mongolia | 40 (16-70) | 4.08 (1.62-7.3) | 109 (44-192) | 5.53 (2.23-9.56) | 0.73 (0.61-0.86) |
| Montenegro | 145 (59-253) | 24.61 (10.06-43.14) | 395 (172-695) | 44.18 (18.82-77.58) | 2.06 (1.88-2.25) |
| Morocco | 323 (124-610) | 2.55 (0.97-4.74) | 2372 (966-4355) | 7.94 (3.19-14.64) | 3.98 (3.89-4.07) |
| Mozambique | 26 (5-62) | 0.4 (0.07-0.99) | 273 (91-514) | 2.45 (0.8-4.73) | 6.26 (6.08-6.44) |
| Myanmar | 111 (19-235) | 0.41 (0.04-0.9) | 751 (251-1430) | 1.5 (0.48-2.97) | 4.06 (3.92-4.2) |
| Namibia | 17 (6-31) | 3.4 (1.27-6.48) | 114 (46-195) | 10.97 (4.46-19.03) | 3.84 (3.62-4.07) |
| Nauru | 1 (0-2) | 19.19 (8.07-33.53) | 2 (1-3) | 34.5 (14.4-61.45) | 1.88 (1.77-2) |
| Nepal | 14 (2-41) | 0.12 (0-0.39) | 219 (72-429) | 0.94 (0.29-1.78) | 7.26 (7.11-7.4) |
| Netherlands | 1695 (666-2957) | 8.26 (3.25-14.44) | 4268 (1614-7622) | 11.1 (4.22-20.13) | 0.65 (0.54-0.76) |
| New Zealand | 502 (205-876) | 13.13 (5.35-22.75) | 1750 (719-3115) | 19.61 (8.15-35.06) | 1.58 (1.43-1.73) |
| Nicaragua | 104 (41-184) | 7.43 (2.84-13.23) | 598 (244-1067) | 13.36 (5.41-23.98) | 2.02 (1.9-2.14) |
| Niger | 22 (7-43) | 0.98 (0.29-1.95) | 106 (38-209) | 1.49 (0.49-3.05) | 1.29 (1.14-1.45) |
| Nigeria | 781 (303-1419) | 2.38 (0.88-4.4) | 4651 (1867-7982) | 7.25 (2.93-12.45) | 3.3 (3.16-3.44) |
| Niue | 0 (0-1) | 12.05 (4.68-23.03) | 0 (0-1) | 23.93 (10.1-42.38) | 2.12 (2.03-2.2) |
| North Macedonia | 168 (66-296) | 9.92 (3.91-17.77) | 509 (205-939) | 18.4 (7.59-33.88) | 1.97 (1.67-2.26) |
| Northern Mariana Islands | 3 (1-5) | 14.84 (6.08-26.69) | 13 (6-21) | 27.47 (12.16-46.85) | 2 (1.88-2.12) |
| Norway | 577 (226-961) | 7.87 (3.06-13.14) | 1062 (420-1807) | 9.51 (3.73-16.16) | 0.39 (0.26-0.53) |
| Oman | 24 (10-41) | 4.11 (1.65-7.17) | 231 (96-399) | 15.13 (6.39-26.18) | 4.7 (4.5-4.9) |
| Pakistan | 190 (38-446) | 0.34 (0.06-0.82) | 4137 (1556-6994) | 3.9 (1.48-6.89) | 8.93 (8.35-9.51) |
| Palau | 1 (0-2) | 12.11 (4.89-22.01) | 4 (2-7) | 18.86 (8.09-33.37) | 1.44 (1.39-1.5) |
| Palestine | 60 (22-114) | 8.49 (3.16-16.49) | 289 (121-496) | 16.35 (6.77-27.68) | 2.03 (1.89-2.17) |
| Panama | 147 (60-272) | 10.86 (4.4-20.16) | 864 (363-1510) | 19.04 (7.99-33.31) | 1.86 (1.8-1.92) |
| Papua New Guinea | 29 (9-61) | 1.29 (0.39-2.72) | 187 (70-370) | 2.9 (1.07-5.64) | 2.55 (2.46-2.65) |
| Paraguay | 133 (47-251) | 6.29 (2.22-11.89) | 788 (313-1476) | 14.27 (5.63-26.86) | 2.82 (2.72-2.92) |
| Peru | 463 (169-883) | 4.01 (1.45-7.58) | 3035 (1251-5432) | 9.1 (3.74-16.24) | 2.59 (2.49-2.69) |
| Philippines | 333 (111-621) | 1.19 (0.36-2.26) | 3167 (1199-5548) | 4.28 (1.59-7.66) | 4.17 (3.92-4.41) |
| Poland | 5525 (2235-9740) | 13.34 (5.4-23.45) | 12557 (5127-22383) | 16.28 (6.64-29.05) | 0.54 (0.15-0.94) |
| Portugal | 739 (261-1427) | 5.55 (1.99-10.54) | 2734 (1035-5042) | 9.49 (3.65-17.65) | 1.62 (1.48-1.77) |
| Puerto Rico | 398 (154-752) | 11.65 (4.52-22.25) | 1314 (561-2319) | 16.57 (7.1-29.04) | 0.95 (0.83-1.06) |
| Qatar | 7 (3-14) | 11.94 (3.84-25.07) | 99 (44-171) | 17.43 (6.71-32.18) | 0.83 (0.38-1.29) |
| Republic of Korea | 89 (16-210) | 0.31 (0.05-0.75) | 2834 (1071-5417) | 3.04 (1.16-5.84) | 8.16 (7.84-8.47) |
| Republic of Moldova | 385 (157-667) | 10.03 (4.07-17.76) | 1008 (432-1726) | 16.6 (7.12-28.5) | 1.49 (1.39-1.59) |
| Romania | 2222 (852-3909) | 8.68 (3.26-15.31) | 4313 (1762-7403) | 10.75 (4.39-18.43) | 0.35 (0.21-0.48) |
| Russian Federation | 15295 (6341-25881) | 9.3 (3.82-15.52) | 41695 (17494-72255) | 17.03 (7.16-29.53) | 2.01 (1.85-2.18) |
| Rwanda | 7 (1-22) | 0.25 (0.01-0.83) | 66 (20-133) | 1.12 (0.32-2.39) | 4.23 (3.93-4.53) |
| Saint Kitts and Nevis | 3 (1-5) | 7.34 (2.73-13.58) | 9 (4-17) | 16.23 (6.45-29.25) | 2.71 (2.56-2.85) |
| Saint Lucia | 4 (1-7) | 4.63 (1.65-8.58) | 25 (10-45) | 10.62 (4.24-18.87) | 2.37 (2.27-2.48) |
| Saint Vincent and the Grenadines | 2 (1-4) | 2.74 (0.95-5.42) | 11 (4-20) | 8.4 (3.38-14.9) | 3.75 (3.65-3.85) |
| Samoa | 13 (5-23) | 14.84 (5.85-26.81) | 34 (15-58) | 24.7 (10.4-42.89) | 1.62 (1.58-1.66) |
| San Marino | 4 (2-7) | 10.31 (4.06-18.46) | 9 (3-17) | 10.56 (3.97-19.18) | 0.38 (0.22-0.55) |
| Sao Tome and Principe | 1 (0-2) | 2.17 (0.77-4.07) | 6 (3-11) | 7.17 (2.92-13) | 3.88 (3.74-4.03) |
| Saudi Arabia | 278 (111-484) | 5.82 (2.25-10.06) | 2120 (971-3574) | 15.59 (6.84-26.63) | 3.19 (3.08-3.3) |
| Senegal | 51 (18-92) | 1.82 (0.64-3.33) | 277 (108-490) | 4.27 (1.71-7.54) | 2.71 (2.63-2.8) |
| Serbia | 801 (316-1507) | 8.5 (3.38-16.27) | 2339 (974-4143) | 13.4 (5.62-23.79) | 1.1 (0.9-1.29) |
| Seychelles | 2 (1-4) | 4.09 (1.69-7.13) | 13 (5-22) | 11.9 (4.78-20.42) | 3.62 (3.46-3.78) |
| Sierra Leone | 19 (6-39) | 1.02 (0.32-2.14) | 88 (34-158) | 2.71 (1.05-4.98) | 3.13 (3-3.25) |
| Singapore | 10 (3-22) | 0.4 (0.1-0.91) | 250 (98-446) | 2.87 (1.12-5.08) | 6 (5.51-6.5) |
| Slovakia | 1106 (459-1942) | 18.94 (7.87-33.09) | 2330 (1022-4081) | 23.84 (10.51-41.55) | 0.78 (0.68-0.87) |
| Slovenia | 279 (111-494) | 11.4 (4.52-20.23) | 701 (290-1218) | 14.12 (5.83-24.61) | 0.82 (0.61-1.04) |
| Solomon Islands | 4 (1-9) | 2.72 (0.9-5.97) | 25 (10-49) | 6.73 (2.42-13.48) | 2.95 (2.9-3) |
| Somalia | 12 (3-24) | 0.38 (0.08-0.87) | 74 (25-145) | 1.13 (0.35-2.33) | 3.53 (3.41-3.66) |
| South Africa | 1300 (538-2185) | 6.94 (2.83-11.68) | 5810 (2482-9939) | 14.72 (6.33-25.11) | 2.42 (2.19-2.64) |
| South Sudan | 2 (-1-7) | 0.07 (-0.04-0.3) | 15 (2-37) | 0.37 (0.04-0.91) | 5.65 (5.37-5.94) |
| Spain | 5869 (2359-10688) | 10.75 (4.29-19.49) | 18188 (7321-32506) | 15.88 (6.49-28.41) | 1.16 (0.96-1.36) |
| Sri Lanka | 119 (40-234) | 1.2 (0.38-2.42) | 1130 (426-2121) | 4.53 (1.67-8.83) | 4.53 (4.38-4.68) |
| Sudan | 293 (112-524) | 3.71 (1.43-6.8) | 1499 (594-2601) | 9.32 (3.59-15.9) | 3.01 (2.91-3.11) |
| Suriname | 6 (2-12) | 2.49 (0.83-4.75) | 44 (16-82) | 7.12 (2.48-13.26) | 3.48 (3.37-3.6) |
| Sweden | 1352 (514-2416) | 8.31 (3.16-14.82) | 4882 (1906-9042) | 19.49 (7.62-36.4) | 2.78 (2.62-2.94) |
| Switzerland | 491 (199-838) | 4.4 (1.82-7.55) | 1197 (471-2096) | 5.58 (2.18-9.75) | 1.2 (0.96-1.44) |
| Syrian Arab Republic | 291 (118-534) | 6.82 (2.73-12.58) | 1734 (741-3027) | 17.3 (7.27-30.38) | 2.74 (2.6-2.89) |
| Taiwan (Province of China) | 241 (80-502) | 1.76 (0.55-4) | 2617 (1021-4470) | 5.91 (2.31-10.04) | 3.62 (3.24-4) |
| Tajikistan | 113 (43-200) | 4.57 (1.74-8) | 280 (109-513) | 5.76 (2.34-10.21) | 0.6 (0.47-0.74) |
| Thailand | 273 (69-575) | 0.77 (0.16-1.85) | 5402 (2156-9724) | 4.92 (1.96-8.9) | 5.98 (5.7-6.26) |
| Timor-Leste | 0 (0-0) | -0.07 (-0.12--0.03) | 2 (0-5) | 0.25 (0-0.59) | 28.88 (21.69-36.5) |
| Togo | 16 (6-30) | 1.6 (0.59-3.14) | 134 (51-237) | 4.95 (1.88-8.93) | 3.67 (3.64-3.71) |
| Tokelau | 0 (0-0) | 11.65 (4.68-22.65) | 0 (0-1) | 22.41 (9.52-40.33) | 2.07 (1.96-2.18) |
| Tonga | 7 (3-13) | 13.21 (5.6-23.01) | 19 (8-32) | 24.35 (10.45-41.44) | 1.99 (1.9-2.09) |
| Trinidad and Tobago | 67 (30-112) | 9.04 (3.86-15.03) | 283 (118-506) | 14.89 (6.26-26.54) | 1.5 (1.43-1.57) |
| Tunisia | 117 (44-214) | 2.62 (0.98-5.06) | 1195 (479-2093) | 10.29 (4.1-18.52) | 4.52 (4.36-4.68) |
| Turkmenistan | 91 (35-164) | 5.29 (1.99-9.49) | 301 (116-561) | 8.33 (3.16-15.23) | 1.29 (1.18-1.4) |
| Tuvalu | 1 (0-1) | 10.57 (4.22-20.08) | 2 (1-4) | 21.12 (9.04-37.35) | 2.3 (2.28-2.31) |
| Türkiye | 1888 (770-3364) | 6.54 (2.59-11.46) | 9315 (4013-16305) | 11.07 (4.71-19.19) | 1.67 (1.26-2.08) |
| Uganda | 24 (5-56) | 0.34 (0.06-0.82) | 221 (81-427) | 1.53 (0.53-3.13) | 4.79 (4.72-4.86) |
| Ukraine | 6915 (2861-11422) | 10.23 (4.28-16.85) | 12019 (5073-20789) | 15.05 (6.35-26.14) | 1.1 (0.96-1.24) |
| United Arab Emirates | 21 (8-38) | 5.93 (2.2-10.82) | 437 (200-739) | 17.91 (8.18-30.72) | 5.29 (4.67-5.91) |
| United Kingdom | 8716 (3438-16302) | 9.29 (3.7-17.52) | 21278 (8803-39200) | 14.77 (6.11-27.19) | 1.5 (1.41-1.59) |
| United Republic of Tanzania | 136 (46-273) | 1.41 (0.45-2.89) | 1156 (470-2052) | 5.26 (2.14-9.39) | 4.33 (4.23-4.43) |
| United States of America | 38133 (14850-71114) | 11.68 (4.55-21.87) | 152716 (64970-258787) | 24.74 (10.59-41.79) | 2.39 (2.22-2.55) |
| United States Virgin Islands | 11 (5-21) | 16.89 (6.76-31.65) | 31 (13-55) | 17.27 (7.14-30.81) | 0.13 (0.01-0.24) |
| Uruguay | 232 (93-400) | 6 (2.43-10.38) | 618 (240-1081) | 9.97 (3.94-17.55) | 1.68 (1.57-1.79) |
| Uzbekistan | 433 (169-780) | 4.05 (1.57-7.32) | 1567 (604-2924) | 6.87 (2.63-12.59) | 1.77 (1.69-1.85) |
| Vanuatu | 2 (1-5) | 3.43 (1.16-7.8) | 15 (6-28) | 8.61 (3.35-16.03) | 2.88 (2.81-2.96) |
| Venezuela (Bolivarian Republic of) | 807 (309-1536) | 9.31 (3.6-17.86) | 4686 (2004-8452) | 16.4 (6.93-29.51) | 1.76 (1.69-1.83) |
| Viet Nam | 3 (-33-45) | 0 (-0.1-0.11) | 873 (246-1790) | 0.94 (0.25-2.06) | 18.46 (16.78-20.16) |
| Yemen | 59 (20-123) | 1.45 (0.45-3.1) | 678 (269-1172) | 6.24 (2.5-11.3) | 4.92 (4.67-5.17) |
| Zambia | 16 (4-37) | 0.59 (0.11-1.41) | 331 (125-658) | 5.26 (1.95-10.16) | 7.15 (7.03-7.26) |
| Zimbabwe | 60 (22-105) | 1.63 (0.6-2.95) | 414 (172-715) | 6.93 (2.76-12.24) | 5.14 (4.75-5.52) |
| SDI | | | | | |
|  | No. 1990 | ASDR 1990 | No. 2021 | ASDR 2021 | EAPC (%) |
| High SDI | 95785 (37100-167987) | 8.64 (3.36-15.2) | 324193 (134750-566662) | 14.21 (5.95-24.65) | 1.58 (1.48-1.68) |
| High-middle SDI | 53742 (21649-91086) | 6.2 (2.52-10.44) | 182168 (74547-317112) | 9.33 (3.81-16.2) | 1.28 (1.24-1.32) |
| Middle SDI | 16889 (6475-28280) | 2.01 (0.77-3.37) | 150960 (62576-256705) | 6.18 (2.57-10.42) | 3.67 (3.59-3.75) |
| Low-middle SDI | 7015 (2799-11742) | 1.42 (0.55-2.35) | 56694 (23028-93081) | 4.57 (1.84-7.43) | 3.97 (3.9-4.05) |
| Low SDI | 1236 (416-2329) | 0.62 (0.2-1.19) | 9595 (3655-16707) | 2.21 (0.85-3.91) | 4.22 (4.07-4.36) |
| Gender and Age | | | | | |
|  | No. 1990 | ASDR 1990 | No. 2021 | ASDR 2021 | EAPC (%) |
| Sex |  |  |  |  |  |
| Female | 111652 (44457-189380) | 5.75 (2.29-9.72) | 418636 (176495-716940) | 8.93 (3.77-15.3) | 1.32 (1.27-1.37) |
| Male | 63380 (23550-113099) | 4.23 (1.58-7.44) | 305938 (126786-525494) | 8.32 (3.44-14.4) | 2.32 (2.24-2.4) |
| Sex |  |  |  |  |  |
| 30-34 years | 192 (77-338) | 0.05 (0.02-0.09) | 728 (301-1216) | 0.12 (0.05-0.2) | 3.08 (2.95-3.21) |
| 35-39 years | 709 (270-1377) | 0.2 (0.08-0.39) | 2592 (1060-4885) | 0.46 (0.19-0.87) | 2.67 (2.56-2.79) |
| 40-44 years | 1919 (720-3759) | 0.67 (0.25-1.31) | 7089 (2893-12970) | 1.42 (0.58-2.59) | 2.36 (2.21-2.51) |
| 45-49 years | 3664 (1437-7037) | 1.58 (0.62-3.03) | 14865 (6227-27008) | 3.14 (1.32-5.7) | 2.18 (2.04-2.33) |
| 50-54 years | 7046 (2724-13435) | 3.31 (1.28-6.32) | 27716 (11425-51574) | 6.23 (2.57-11.59) | 2.11 (1.98-2.24) |
| 55-59 years | 11269 (4753-20930) | 6.08 (2.57-11.3) | 45494 (20198-82578) | 11.5 (5.1-20.87) | 2.17 (2.07-2.26) |
| 60-64 years | 17938 (7026-32399) | 11.17 (4.37-20.17) | 64435 (27736-112480) | 20.13 (8.67-35.14) | 1.99 (1.92-2.05) |
| 65-69 years | 23147 (8887-40918) | 18.73 (7.19-33.1) | 86719 (35838-152238) | 31.44 (12.99-55.19) | 1.67 (1.62-1.72) |
| 70-74 years | 24798 (9403-43119) | 29.29 (11.11-50.93) | 106639 (43202-179215) | 51.81 (20.99-87.07) | 1.6 (1.51-1.7) |
| 75-79 years | 30475 (11780-53483) | 49.51 (19.14-86.89) | 100516 (41321-171465) | 76.22 (31.33-130.01) | 1.46 (1.36-1.56) |
| 80-84 years | 24944 (10038-41591) | 70.51 (28.37-117.57) | 98237 (39936-167241) | 112.16 (45.6-190.95) | 1.4 (1.3-1.51) |
| 85-89 years | 17083 (6658-28875) | 113.05 (44.06-191.09) | 82861 (33751-141574) | 181.23 (73.82-309.64) | 1.56 (1.48-1.64) |
| 90-94 years | 8907 (3499-15164) | 207.85 (81.66-353.87) | 61111 (25181-105603) | 341.61 (140.76-590.31) | 1.59 (1.51-1.67) |
| 95+ years | 2944 (1172-5140) | 289.12 (115.12-504.87) | 25571 (10632-44228) | 469.17 (195.07-811.47) | 1.36 (1.23-1.5) |

AF/AFL: Atrial Fibrillation and Flutter, DALYs: Disability-adjusted Life Years, ASDR: Age-standardized DALYs Rate, EAPC: Estimated Annual Percentage Changes. SDI: Social Demographic Index.

**Supplementary table 6. Epidemiological drivers-the contribution of aging, population growth, and epidemiological changes to the burden of AF/AFL caused by high BMI**

| Deaths | | | | | | | | |
| --- | --- | --- | --- | --- | --- | --- | --- | --- |
|  | Location | Overll difference | Age | Population | Epidemiological change | Age (%) | Population(%) | Epidemiological change (%) |
| Both | Central Sub-Saharan Africa | 268.42 | -16.395 | 103.253 | 181.561 | -6.11 | 38.47 | 67.64 |
|  | Eastern Sub-Saharan Africa | 322.87 | -18.898 | 125.475 | 216.291 | -5.85 | 38.86 | 66.99 |
|  | Western Sub-Saharan Africa | 1303.1 | -188.267 | 673.134 | 818.233 | -14.45 | 51.66 | 62.79 |
|  | Southern Sub-Saharan Africa | 801.55 | 78.469 | 242.611 | 480.466 | 9.79 | 30.27 | 59.94 |
|  | North Africa and Middle East | 31962.85 | 4237.634 | 12733.889 | 14991.327 | 13.26 | 39.84 | 46.9 |
|  | High-income Asia Pacific | 580.51 | 191.876 | 58.641 | 329.991 | 33.05 | 10.1 | 56.85 |
|  | Central Asia | 326.62 | -0.214 | 140.524 | 186.313 | -0.07 | 43.02 | 57.04 |
|  | East Asia | 10191.72 | 2183.979 | 1345.816 | 6661.92 | 21.43 | 13.2 | 65.37 |
|  | Southeast Asia | 1801.06 | 240.472 | 374.902 | 1185.69 | 13.35 | 20.82 | 65.83 |
|  | South Asia | 12143 | 621.907 | 3141.214 | 8379.879 | 5.12 | 25.87 | 69.01 |
|  | Central Europe | 1862.88 | 1066.715 | 94.154 | 702.013 | 57.26 | 5.05 | 37.68 |
|  | Eastern Europe | 3466.81 | 854.418 | -48.756 | 2661.144 | 24.65 | -1.41 | 76.76 |
|  | Western Europe | 9470.8 | 2330.13 | 1776.635 | 5364.034 | 24.6 | 18.76 | 56.64 |
|  | Tropical Latin America | 3529.22 | 742.094 | 991.318 | 1795.812 | 21.03 | 28.09 | 50.88 |
|  | Southern Latin America | 813.46 | 120.758 | 285.307 | 407.398 | 14.84 | 35.07 | 50.08 |
|  | Central Latin America | 3243.96 | 816.277 | 1081.321 | 1346.363 | 25.16 | 33.33 | 41.5 |
|  | Andean Latin America | 475.28 | 81.972 | 151.689 | 241.619 | 17.25 | 31.92 | 50.84 |
|  | Caribbean | 490.46 | 92.294 | 135.742 | 262.426 | 18.82 | 27.68 | 53.51 |
|  | High-income North America | 10554.04 | 1641.425 | 2473.465 | 6439.147 | 15.55 | 23.44 | 61.01 |
|  | Oceania | 35.96 | 1.333 | 20.303 | 14.327 | 3.71 | 56.46 | 39.84 |
|  | Australasia | 1031.78 | 210.789 | 316.565 | 504.426 | 20.43 | 30.68 | 48.89 |
|  | Global | 64223.33 | 13306.265 | 24359.164 | 26557.905 | 20.72 | 37.93 | 41.35 |
|  | Location | Overll difference | Age | Population | Epidemiological change | Age (%) | Population(%) | Epidemiological change (%) |
| Male | Central Sub-Saharan Africa | 125.88 | -12.491 | 45.605 | 92.768 | -9.92 | 36.23 | 73.7 |
|  | Eastern Sub-Saharan Africa | 123.85 | -10.355 | 44.163 | 90.044 | -8.36 | 35.66 | 72.7 |
|  | Western Sub-Saharan Africa | 604.76 | -61.406 | 254.859 | 411.303 | -10.15 | 42.14 | 68.01 |
|  | Southern Sub-Saharan Africa | 232.28 | 14.317 | 67.835 | 150.131 | 6.16 | 29.2 | 64.63 |
|  | North Africa and Middle East | 10880.72 | 1328.056 | 3879.742 | 5672.921 | 12.21 | 35.66 | 52.14 |
|  | High-income Asia Pacific | 228.24 | 68.72 | 20.571 | 138.952 | 30.11 | 9.01 | 60.88 |
|  | Central Asia | 109.33 | 8.836 | 31.512 | 68.983 | 8.08 | 28.82 | 63.1 |
|  | East Asia | 3048.71 | 695.81 | 375.27 | 1977.633 | 22.82 | 12.31 | 64.87 |
|  | Southeast Asia | 347.96 | 39.426 | 71.54 | 236.989 | 11.33 | 20.56 | 68.11 |
|  | South Asia | 5110.16 | 229.823 | 1272.285 | 3608.052 | 4.5 | 24.9 | 70.61 |
|  | Central Europe | 795.12 | 412.373 | 30.858 | 351.884 | 51.86 | 3.88 | 44.26 |
|  | Eastern Europe | 1178.73 | 398.148 | -14.383 | 794.966 | 33.78 | -1.22 | 67.44 |
|  | Western Europe | 4459.07 | 1342.987 | 724.52 | 2391.567 | 30.12 | 16.25 | 53.63 |
|  | Tropical Latin America | 1571.44 | 273.442 | 379.429 | 918.571 | 17.4 | 24.15 | 58.45 |
|  | Southern Latin America | 348.15 | 47.791 | 102.858 | 197.504 | 13.73 | 29.54 | 56.73 |
|  | Central Latin America | 1352.1 | 284.333 | 394.51 | 673.261 | 21.03 | 29.18 | 49.79 |
|  | Andean Latin America | 199.44 | 34.324 | 66.769 | 98.349 | 17.21 | 33.48 | 49.31 |
|  | Caribbean | 198.89 | 29.631 | 47.613 | 121.643 | 14.9 | 23.94 | 61.16 |
|  | High-income North America | 5755.81 | 1033.803 | 1183.033 | 3538.97 | 17.96 | 20.55 | 61.49 |
|  | Oceania | 13.93 | 0.557 | 6.903 | 6.466 | 4 | 49.55 | 46.42 |
|  | Australasia | 475.46 | 115.224 | 127.402 | 232.834 | 24.23 | 26.8 | 48.97 |
|  | Global | 28148.57 | 5900.114 | 8726.192 | 13522.264 | 20.96 | 31 | 48.04 |
|  | Location | Overll difference | Age | Population | Epidemiological change | Age (%) | Population(%) | Epidemiological change (%) |
| Female | Central Sub-Saharan Africa | 144.25 | -4.813 | 58.297 | 90.764 | -3.34 | 40.41 | 62.92 |
|  | Eastern Sub-Saharan Africa | 193.23 | -5.93 | 78.154 | 121.011 | -3.07 | 40.45 | 62.63 |
|  | Western Sub-Saharan Africa | 706.55 | -124.27 | 413.847 | 416.971 | -17.59 | 58.57 | 59.02 |
|  | Southern Sub-Saharan Africa | 534.97 | 66.475 | 161.6 | 306.898 | 12.43 | 30.21 | 57.37 |
|  | North Africa and Middle East | 20797.52 | 2726.531 | 8407.937 | 9663.048 | 13.11 | 40.43 | 46.46 |
|  | High-income Asia Pacific | 329.78 | 106.337 | 35.079 | 188.36 | 32.24 | 10.64 | 57.12 |
|  | Central Asia | 202.21 | -17.583 | 99.29 | 120.506 | -8.7 | 49.1 | 59.59 |
|  | East Asia | 6367.25 | 1253.741 | 894.036 | 4219.473 | 19.69 | 14.04 | 66.27 |
|  | Southeast Asia | 1354.86 | 198.036 | 277.556 | 879.264 | 14.62 | 20.49 | 64.9 |
|  | South Asia | 6824.67 | 392.132 | 1833.704 | 4598.838 | 5.75 | 26.87 | 67.39 |
|  | Central Europe | 1045.91 | 614.124 | 62.754 | 369.035 | 58.72 | 6 | 35.28 |
|  | Eastern Europe | 2233.38 | 354.923 | -29.999 | 1908.454 | 15.89 | -1.34 | 85.45 |
|  | Western Europe | 4922.67 | 802.168 | 973.036 | 3147.463 | 16.3 | 19.77 | 63.94 |
|  | Tropical Latin America | 1956.94 | 460.505 | 600.629 | 895.802 | 23.53 | 30.69 | 45.78 |
|  | Southern Latin America | 457.1 | 67.969 | 175.701 | 213.428 | 14.87 | 38.44 | 46.69 |
|  | Central Latin America | 1854.41 | 535.86 | 685.351 | 633.195 | 28.9 | 36.96 | 34.15 |
|  | Andean Latin America | 271.34 | 47.399 | 83.751 | 140.191 | 17.47 | 30.87 | 51.67 |
|  | Caribbean | 284.69 | 62.75 | 85.709 | 136.229 | 22.04 | 30.11 | 47.85 |
|  | High-income North America | 4983.09 | 613.095 | 1279.298 | 3090.693 | 12.3 | 25.67 | 62.02 |
|  | Oceania | 21.61 | 0.67 | 12.869 | 8.07 | 3.1 | 59.55 | 37.34 |
|  | Australasia | 550.55 | 83.877 | 183.691 | 282.979 | 15.24 | 33.36 | 51.4 |
|  | Global | 35487.65 | 6503.931 | 14908.815 | 14074.908 | 18.33 | 42.01 | 39.66 |
| DALYs | | | | | | | | |
|  | Location | Overll difference | Age | Population | Epidemiological change | Age (%) | Population(%) | Epidemiological change (%) |
| Both | Central Sub-Saharan Africa | 6176.42 | -228.648 | 2418.141 | 3986.927 | -3.7 | 39.15 | 64.55 |
|  | Eastern Sub-Saharan Africa | 8959.88 | -272.571 | 3512.671 | 5719.782 | -3.04 | 39.2 | 63.84 |
|  | Western Sub-Saharan Africa | 24148.38 | -2516.707 | 12082.596 | 14582.495 | -10.42 | 50.03 | 60.39 |
|  | Southern Sub-Saharan Africa | 15632.87 | 1762.853 | 5329.181 | 8540.834 | 11.28 | 34.09 | 54.63 |
|  | North Africa and Middle East | 466562.16 | 56519.865 | 192127.866 | 217914.431 | 12.11 | 41.18 | 46.71 |
|  | High-income Asia Pacific | 10784.98 | 2641.116 | 1246.436 | 6897.429 | 24.49 | 11.56 | 63.95 |
|  | Central Asia | 9775.17 | 930.455 | 4266.477 | 4578.239 | 9.52 | 43.65 | 46.84 |
|  | East Asia | 193424 | 37182.107 | 26148.188 | 130093.701 | 19.22 | 13.52 | 67.26 |
|  | Southeast Asia | 42759.59 | 5398.659 | 9665.394 | 27695.541 | 12.63 | 22.6 | 64.77 |
|  | South Asia | 238315.64 | 9854.622 | 64769.6 | 163691.415 | 4.14 | 27.18 | 68.69 |
|  | Central Europe | 33573.29 | 15555.81 | 1746.175 | 16271.3 | 46.33 | 5.2 | 48.47 |
|  | Eastern Europe | 65856.87 | 15023.421 | -941.708 | 51775.159 | 22.81 | -1.43 | 78.62 |
|  | Western Europe | 153498.49 | 35824.198 | 30826.013 | 86848.281 | 23.34 | 20.08 | 56.58 |
|  | Tropical Latin America | 71801.03 | 14077.664 | 22268.224 | 35455.138 | 19.61 | 31.01 | 49.38 |
|  | Southern Latin America | 13279.64 | 1817.061 | 5248.672 | 6213.906 | 13.68 | 39.52 | 46.79 |
|  | Central Latin America | 69354.07 | 15747.653 | 24530.778 | 29075.642 | 22.71 | 35.37 | 41.92 |
|  | Andean Latin America | 11357.6 | 1716.038 | 3948.578 | 5692.981 | 15.11 | 34.77 | 50.12 |
|  | Caribbean | 10078.54 | 1824.323 | 2824.547 | 5429.675 | 18.1 | 28.03 | 53.87 |
|  | High-income North America | 235976.27 | 42235.125 | 57181.904 | 136559.239 | 17.9 | 24.23 | 57.87 |
|  | Oceania | 1047.67 | 56.08 | 581.153 | 410.439 | 5.35 | 55.47 | 39.18 |
|  | Australasia | 17674.14 | 3328.178 | 5517.223 | 8828.734 | 18.83 | 31.22 | 49.95 |
|  | Global | 1250312.9 | 239317.753 | 484336.748 | 526658.398 | 19.14 | 38.74 | 42.12 |
|  | Location | Overll difference | Age | Population | Epidemiological change | Age (%) | Population(%) | Epidemiological change (%) |
| Male | Central Sub-Saharan Africa | 2957.95 | -150.554 | 1070.719 | 2037.789 | -5.09 | 36.2 | 68.89 |
|  | Eastern Sub-Saharan Africa | 3247.35 | -153.344 | 1134.321 | 2266.372 | -4.72 | 34.93 | 69.79 |
|  | Western Sub-Saharan Africa | 10516.93 | -1022.76 | 4434.684 | 7105.002 | -9.72 | 42.17 | 67.56 |
|  | Southern Sub-Saharan Africa | 5384.12 | 410.841 | 1653.536 | 3319.742 | 7.63 | 30.71 | 61.66 |
|  | North Africa and Middle East | 190274.88 | 20514.945 | 69316.184 | 100443.754 | 10.78 | 36.43 | 52.79 |
|  | High-income Asia Pacific | 4871.34 | 952.43 | 515.246 | 3403.668 | 19.55 | 10.58 | 69.87 |
|  | Central Asia | 4128.58 | 517.5 | 1427.026 | 2184.051 | 12.53 | 34.56 | 52.9 |
|  | East Asia | 66651.28 | 12769.484 | 8525.254 | 45356.542 | 19.16 | 12.79 | 68.05 |
|  | Southeast Asia | 10291.73 | 1126.002 | 2353.114 | 6812.617 | 10.94 | 22.86 | 66.2 |
|  | South Asia | 97649.67 | 3603.725 | 25424.636 | 68621.313 | 3.69 | 26.04 | 70.27 |
|  | Central Europe | 16240.98 | 6656.125 | 664.322 | 8920.53 | 40.98 | 4.09 | 54.93 |
|  | Eastern Europe | 26106.77 | 7001.41 | -337.991 | 19443.352 | 26.82 | -1.29 | 74.48 |
|  | Western Europe | 81476.16 | 20979.855 | 14867.079 | 45629.225 | 25.75 | 18.25 | 56 |
|  | Tropical Latin America | 35015.55 | 5676.168 | 9492.456 | 19846.923 | 16.21 | 27.11 | 56.68 |
|  | Southern Latin America | 6743.86 | 792.731 | 2226.038 | 3725.094 | 11.75 | 33.01 | 55.24 |
|  | Central Latin America | 31721.85 | 5925.796 | 9904.141 | 15891.909 | 18.68 | 31.22 | 50.1 |
|  | Andean Latin America | 5222.06 | 738.226 | 1840.345 | 2643.485 | 14.14 | 35.24 | 50.62 |
|  | Caribbean | 4406.97 | 654.526 | 1099.391 | 2653.055 | 14.85 | 24.95 | 60.2 |
|  | High-income North America | 137580.79 | 25164.526 | 30503.171 | 81913.093 | 18.29 | 22.17 | 59.54 |
|  | Oceania | 445.01 | 24.177 | 222.739 | 198.09 | 5.43 | 50.05 | 44.51 |
|  | Australasia | 8938.1 | 1772.843 | 2542.183 | 4623.07 | 19.83 | 28.44 | 51.72 |
|  | Global | 601902.4 | 109930.038 | 199547.166 | 292425.191 | 18.26 | 33.15 | 48.58 |
|  | Location | Overll difference | Age | Population | Epidemiological change | Age (%) | Population(%) | Epidemiological change (%) |
| Female | Central Sub-Saharan Africa | 3239.36 | -96.294 | 1354.175 | 1981.477 | -2.97 | 41.8 | 61.17 |
|  | Eastern Sub-Saharan Africa | 5590.32 | -78.829 | 2326.677 | 3342.469 | -1.41 | 41.62 | 59.79 |
|  | Western Sub-Saharan Africa | 13756.82 | -1301.2 | 7600.958 | 7457.06 | -9.46 | 55.25 | 54.21 |
|  | Southern Sub-Saharan Africa | 9845.45 | 1388.961 | 3467.016 | 4989.476 | 14.11 | 35.21 | 50.68 |
|  | North Africa and Middle East | 272771.52 | 34122.288 | 117754.642 | 120894.592 | 12.51 | 43.17 | 44.32 |
|  | High-income Asia Pacific | 5666.73 | 1454.26 | 688.911 | 3523.554 | 25.66 | 12.16 | 62.18 |
|  | Central Asia | 5503.06 | 264.901 | 2646.429 | 2591.73 | 4.81 | 48.09 | 47.1 |
|  | East Asia | 118314.13 | 22001.763 | 16854.875 | 79457.488 | 18.6 | 14.25 | 67.16 |
|  | Southeast Asia | 30737.01 | 4166.027 | 6842.071 | 19728.911 | 13.55 | 22.26 | 64.19 |
|  | South Asia | 137124.45 | 6249.758 | 38892.739 | 91981.956 | 4.56 | 28.36 | 67.08 |
|  | Central Europe | 17305.17 | 8473.855 | 1075.111 | 7756.2 | 48.97 | 6.21 | 44.82 |
|  | **Eastern Europe** | 39592.08 | 6716.814 | -537.384 | 33412.651 | 16.97 | -1.36 | 84.39 |
|  | Western Europe | 72128.13 | 13156.235 | 15346.732 | 43625.168 | 18.24 | 21.28 | 60.48 |
|  | Tropical Latin America | 37052.76 | 8329.634 | 12676.965 | 16046.164 | 22.48 | 34.21 | 43.31 |
|  | Southern Latin America | 6589.3 | 975.636 | 2959.044 | 2654.625 | 14.81 | 44.91 | 40.29 |
|  | Central Latin America | 37321.34 | 9894.571 | 14622.07 | 12804.701 | 26.51 | 39.18 | 34.31 |
|  | Andean Latin America | 6091.21 | 978.167 | 2096.294 | 3016.746 | 16.06 | 34.42 | 49.53 |
|  | Caribbean | 5588.64 | 1172.128 | 1695.94 | 2720.573 | 20.97 | 30.35 | 48.68 |
|  | High-income North America | 102061.78 | 17339.673 | 26894.193 | 57827.916 | 16.99 | 26.35 | 56.66 |
|  | Oceania | 596.29 | 30.383 | 352.753 | 213.155 | 5.1 | 59.16 | 35.75 |
|  | Australasia | 8700.92 | 1438.643 | 2923.399 | 4338.876 | 16.53 | 33.6 | 49.87 |
|  | Global | 648922.94 | 119633.227 | 276248.768 | 253040.942 | 18.44 | 42.57 | 38.99 |

AF/AFL: Atrial Fibrillation and Flutter, DALYs: Disability-adjusted Life Years

**Supplementary table 7. Forecast of the Overall Burden of High BMI.**

|  | | **Both** | | **male** | | **femail** | |
| --- | --- | --- | --- | --- | --- | --- | --- |
| **year** | **measure** | **ASR** | **Cases** | **ASR** | **Cases** | **ASR** | **Cases** |
| 2022 | Deaths | 44.51 | 3882810 | 45.26 | 1784485 | 43.47 | 2098324 |
| 2023 | Deaths | 44.66 | 4006462 | 45.44 | 1841641 | 43.61 | 2164821 |
| 2024 | Deaths | 44.82 | 4140932 | 45.62 | 1903360 | 43.76 | 2237572 |
| 2025 | Deaths | 44.94 | 4277200 | 45.74 | 1965061 | 43.88 | 2312138 |
| 2026 | Deaths | 45.06 | 4414139 | 45.87 | 2026940 | 44.00 | 2387199 |
| 2027 | Deaths | 45.18 | 4548771 | 46.00 | 2087679 | 44.12 | 2461092 |
| 2028 | Deaths | 45.30 | 4687344 | 46.12 | 2150046 | 44.24 | 2537298 |
| 2029 | Deaths | 45.42 | 4834559 | 46.25 | 2216093 | 44.36 | 2618466 |
| 2030 | Deaths | 45.50 | 4982683 | 46.32 | 2281320 | 44.46 | 2701363 |
| 2031 | Deaths | 45.59 | 5132606 | 46.39 | 2347005 | 44.55 | 2785601 |
| 2032 | Deaths | 45.67 | 5281818 | 46.46 | 2411982 | 44.65 | 2869836 |
| 2033 | Deaths | 45.75 | 5435342 | 46.53 | 2478546 | 44.75 | 2956796 |
| 2034 | Deaths | 45.84 | 5596997 | 46.60 | 2548440 | 44.85 | 3048557 |
| 2035 | Deaths | 45.88 | 5758419 | 46.61 | 2616839 | 44.91 | 3141580 |
| 2036 | Deaths | 45.92 | 5920956 | 46.63 | 2685347 | 44.98 | 3235609 |
| 2037 | Deaths | 45.96 | 6081698 | 46.64 | 2752682 | 45.05 | 3329016 |
| 2038 | Deaths | 46.00 | 6245831 | 46.65 | 2821175 | 45.12 | 3424656 |
| 2039 | Deaths | 46.04 | 6417169 | 46.67 | 2892563 | 45.18 | 3524606 |
| 2040 | Deaths | 46.09 | 6591317 | 46.68 | 2964970 | 45.25 | 3626347 |
| 2022 | DALYs | 1507.26 | 134126820 | 1513.20 | 64060864 | 1495.36 | 70065957 |
| 2023 | DALYs | 1521.24 | 138474121 | 1527.13 | 66091513 | 1509.46 | 72382608 |
| 2024 | DALYs | 1535.22 | 142977848 | 1541.06 | 68188678 | 1523.57 | 74789169 |
| 2025 | DALYs | 1546.99 | 147379760 | 1552.36 | 70211920 | 1535.84 | 77167840 |
| 2026 | DALYs | 1558.77 | 151819615 | 1563.67 | 72249229 | 1548.11 | 79570386 |
| 2027 | DALYs | 1570.55 | 156255842 | 1574.97 | 74281675 | 1560.38 | 81974167 |
| 2028 | DALYs | 1582.34 | 160750631 | 1586.28 | 76337689 | 1572.65 | 84412942 |
| 2029 | DALYs | 1594.11 | 165357290 | 1597.58 | 78441379 | 1584.92 | 86915912 |
| 2030 | DALYs | 1603.02 | 169748815 | 1605.62 | 80415049 | 1594.66 | 89333766 |
| 2031 | DALYs | 1611.94 | 174165961 | 1613.66 | 82394901 | 1604.39 | 91771060 |
| 2032 | DALYs | 1620.86 | 178576044 | 1621.70 | 84365293 | 1614.12 | 94210752 |
| 2033 | DALYs | 1629.78 | 183033743 | 1629.73 | 86351739 | 1623.86 | 96682004 |
| 2034 | DALYs | 1638.69 | 187583628 | 1637.77 | 88375680 | 1633.59 | 99207948 |
| 2035 | DALYs | 1644.09 | 191810035 | 1641.96 | 90218494 | 1640.10 | 101591540 |
| 2036 | DALYs | 1649.49 | 196033029 | 1646.14 | 92055332 | 1646.60 | 103977697 |
| 2037 | DALYs | 1654.89 | 200215046 | 1650.33 | 93869349 | 1653.11 | 106345697 |
| 2038 | DALYs | 1660.30 | 204411324 | 1654.51 | 95685188 | 1659.61 | 108726135 |
| 2039 | DALYs | 1665.71 | 208672259 | 1658.70 | 97526502 | 1666.12 | 111145757 |
| 2040 | DALYs | 1671.12 | 212946003 | 1662.88 | 99371300 | 1672.62 | 113574703 |
| 2022 | YLLs | 978.58 | 87122968 | 1032.30 | 43475586 | 922.23 | 43647382 |
| 2023 | YLLs | 983.18 | 89639019 | 1037.20 | 44692891 | 926.57 | 44946128 |
| 2024 | YLLs | 987.77 | 92260473 | 1042.11 | 45953968 | 930.91 | 46306505 |
| 2025 | YLLs | 991.72 | 94885049 | 1046.19 | 47205013 | 934.78 | 47680036 |
| 2026 | YLLs | 995.67 | 97521251 | 1050.27 | 48459109 | 938.64 | 49062142 |
| 2027 | YLLs | 999.64 | 100136807 | 1054.36 | 49701612 | 942.50 | 50435195 |
| 2028 | YLLs | 1003.60 | 102783969 | 1058.44 | 50956071 | 946.37 | 51827897 |
| 2029 | YLLs | 1007.56 | 105505722 | 1062.52 | 52241681 | 950.23 | 53264041 |
| 2030 | YLLs | 1010.60 | 108180345 | 1065.47 | 53490254 | 953.33 | 54690091 |
| 2031 | YLLs | 1013.64 | 110865275 | 1068.43 | 54739283 | 956.43 | 56125992 |
| 2032 | YLLs | 1016.68 | 113535717 | 1071.38 | 55976894 | 959.53 | 57558823 |
| 2033 | YLLs | 1019.73 | 116236352 | 1074.33 | 57223744 | 962.62 | 59012608 |
| 2034 | YLLs | 1022.77 | 119002301 | 1077.28 | 58496546 | 965.72 | 60505756 |
| 2035 | YLLs | 1024.60 | 121678650 | 1078.81 | 59710289 | 967.79 | 61968360 |
| 2036 | YLLs | 1026.43 | 124350778 | 1080.33 | 60918359 | 969.85 | 63432418 |
| 2037 | YLLs | 1028.27 | 126989707 | 1081.86 | 62107720 | 971.92 | 64881987 |
| 2038 | YLLs | 1030.11 | 129642276 | 1083.39 | 63299360 | 973.98 | 66342917 |
| 2039 | YLLs | 1031.96 | 132346042 | 1084.91 | 64510846 | 976.05 | 67835195 |
| 2040 | YLLs | 1033.80 | 135059494 | 1086.44 | 65724516 | 978.11 | 69334979 |
| 2022 | YLDs | 527.97 | 46944515 | 480.41 | 20566292 | 572.14 | 26378224 |
| 2023 | YLDs | 537.12 | 48756456 | 489.28 | 21373663 | 581.57 | 27382794 |
| 2024 | YLDs | 546.26 | 50619827 | 498.15 | 22203977 | 591.01 | 28415850 |
| 2025 | YLDs | 553.58 | 52355470 | 505.12 | 22966973 | 598.67 | 29388498 |
| 2026 | YLDs | 560.90 | 54117161 | 512.09 | 23741253 | 606.34 | 30375908 |
| 2027 | YLDs | 568.22 | 55895583 | 519.06 | 24522547 | 614.00 | 31373036 |
| 2028 | YLDs | 575.54 | 57700879 | 526.03 | 25315809 | 621.67 | 32385071 |
| 2029 | YLDs | 582.85 | 59543479 | 533.00 | 26125978 | 629.34 | 33417501 |
| 2030 | YLDs | 587.86 | 61184852 | 537.59 | 26833099 | 634.77 | 34351753 |
| 2031 | YLDs | 592.86 | 62840843 | 542.18 | 27546261 | 640.19 | 35294582 |
| 2032 | YLDs | 597.87 | 64503418 | 546.77 | 28261651 | 645.62 | 36241767 |
| 2033 | YLDs | 602.87 | 66182538 | 551.36 | 28984123 | 651.05 | 37198416 |
| 2034 | YLDs | 607.87 | 67887758 | 555.95 | 29718471 | 656.48 | 38169287 |
| 2035 | YLDs | 610.19 | 69320949 | 557.78 | 30315945 | 659.29 | 39005004 |
| 2036 | YLDs | 612.51 | 70754042 | 559.62 | 30913468 | 662.11 | 39840574 |
| 2037 | YLDs | 614.83 | 72178485 | 561.45 | 31507180 | 664.92 | 40671305 |
| 2038 | YLDs | 617.14 | 73603018 | 563.28 | 32100985 | 667.73 | 41502033 |
| 2039 | YLDs | 619.46 | 75040216 | 565.12 | 32700870 | 670.55 | 42339346 |
| 2040 | YLDs | 621.77 | 76480033 | 566.95 | 33302600 | 673.36 | 43177434 |

DALYs: Disability-adjusted Life Years; YLLs: Years of Life Lost; YLDs: Years Lived with Disability; ASR: Age-standardized DALYs Rate.
