# Supplementary figures and images for: Global trends and projections of high BMI burden and its independent impact on atrial fibrillation and flutter
Source: Int Health. 2025 Feb 4;17(4):552–65. doi: 10.1093/inthealth/ihaf005 (PMC12212217; doi:10.1093/inthealth/ihaf005)

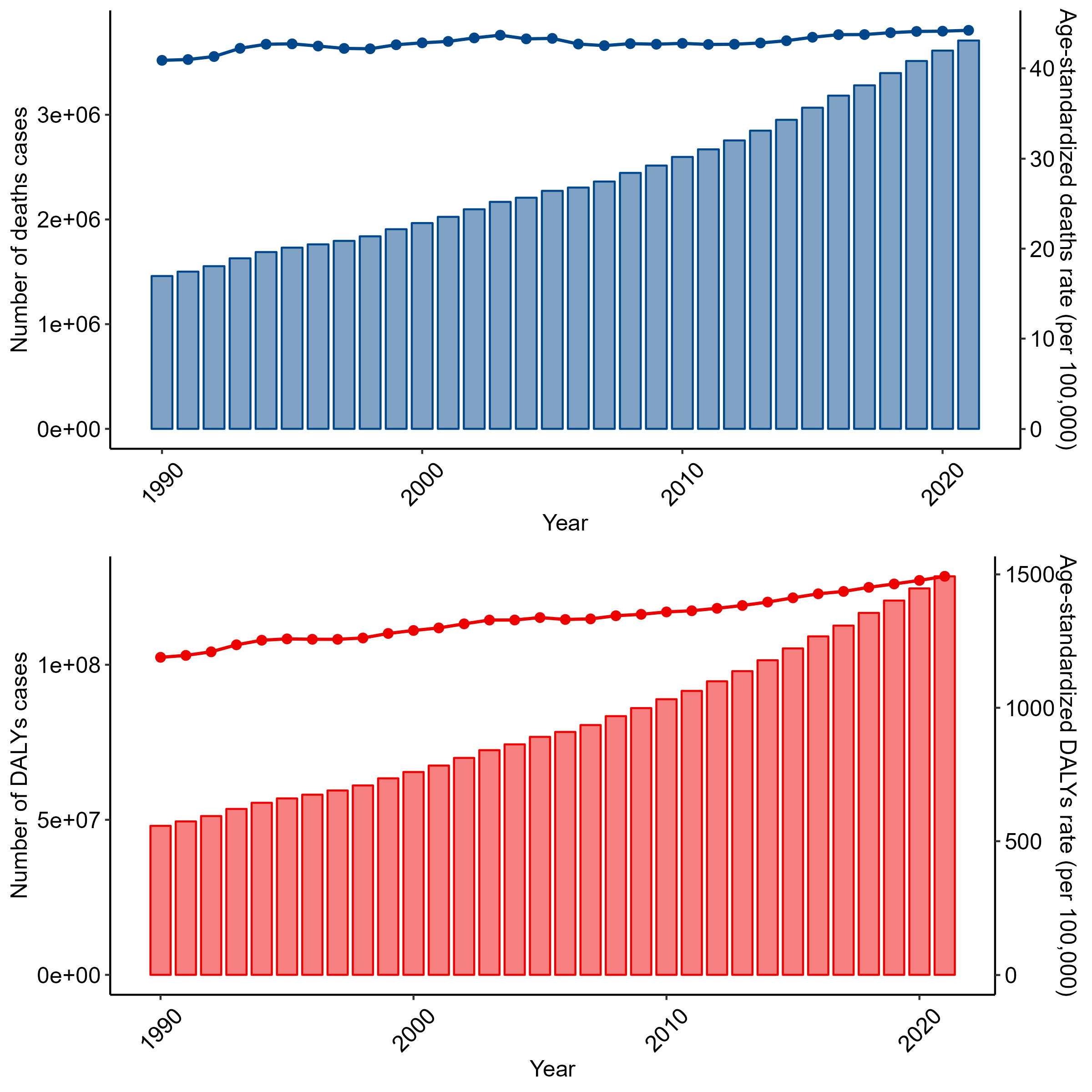

Supplement: ihaf005_Supplemental_Files [file ihaf005_supplemental_files.zip › Supplementary Figure 1.tiff]

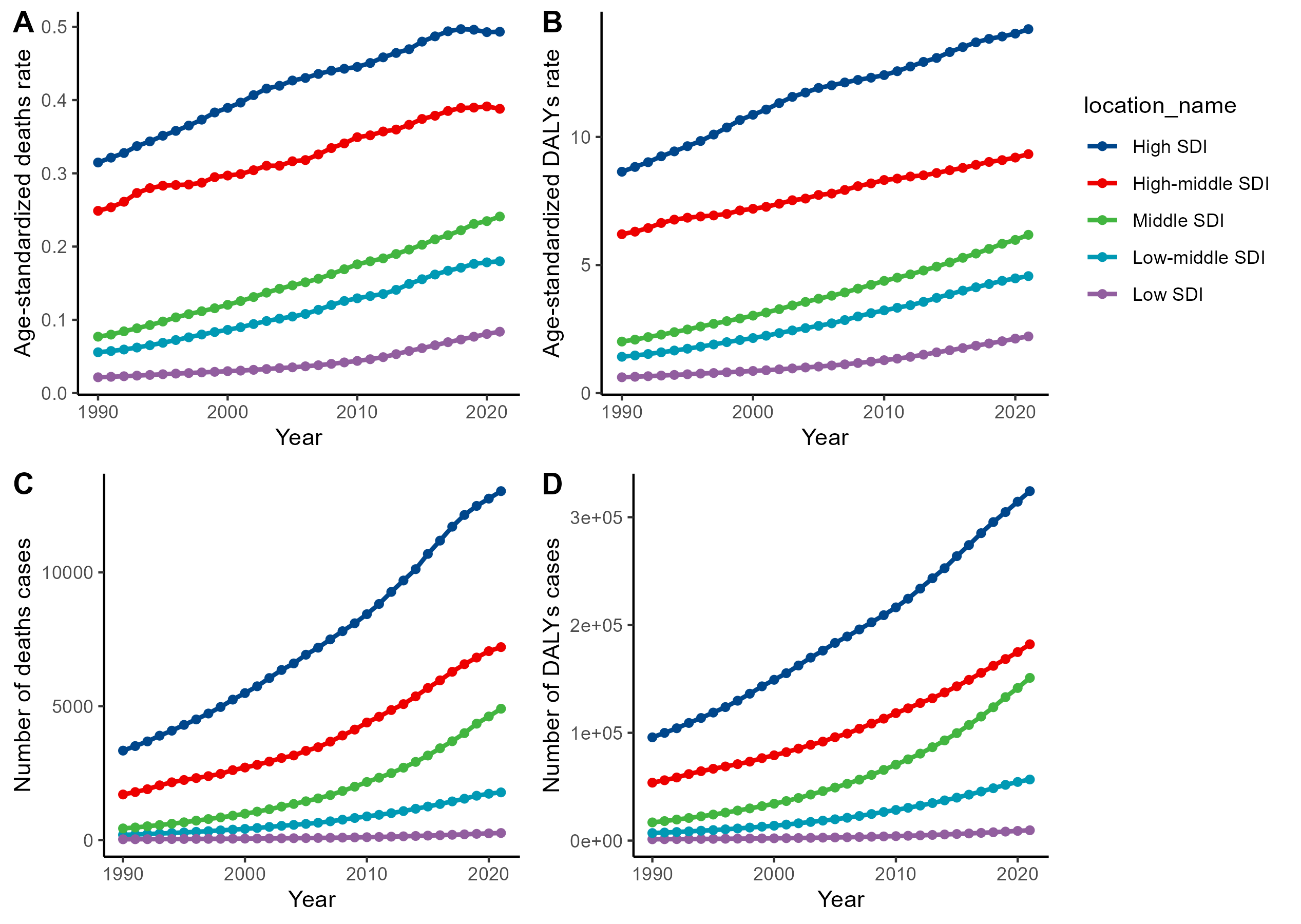

Supplement: ihaf005_Supplemental_Files [file ihaf005_supplemental_files.zip › Supplementary Figure 10.tiff]

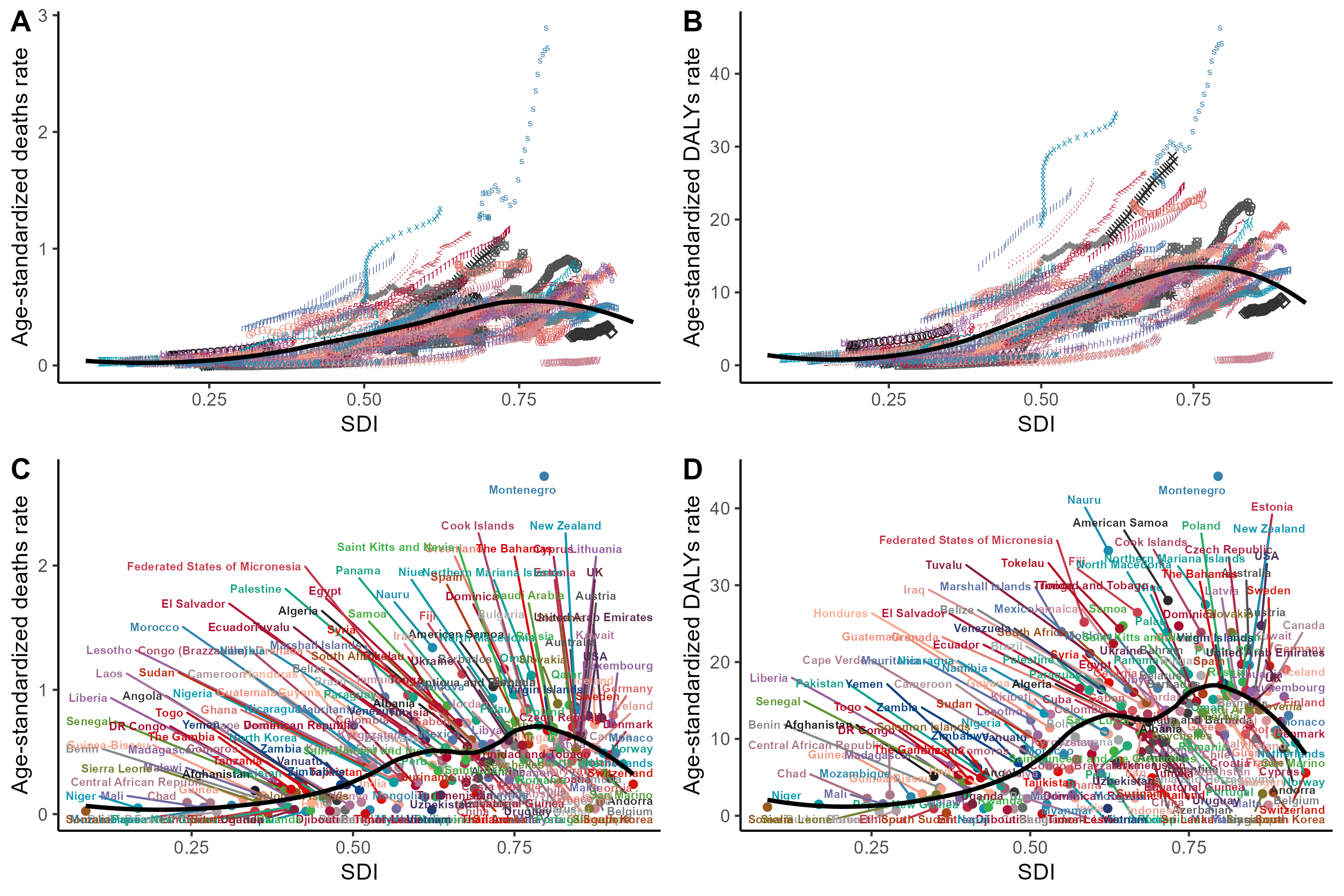

Supplement: ihaf005_Supplemental_Files [file ihaf005_supplemental_files.zip › Supplementary Figure 11.tiff]

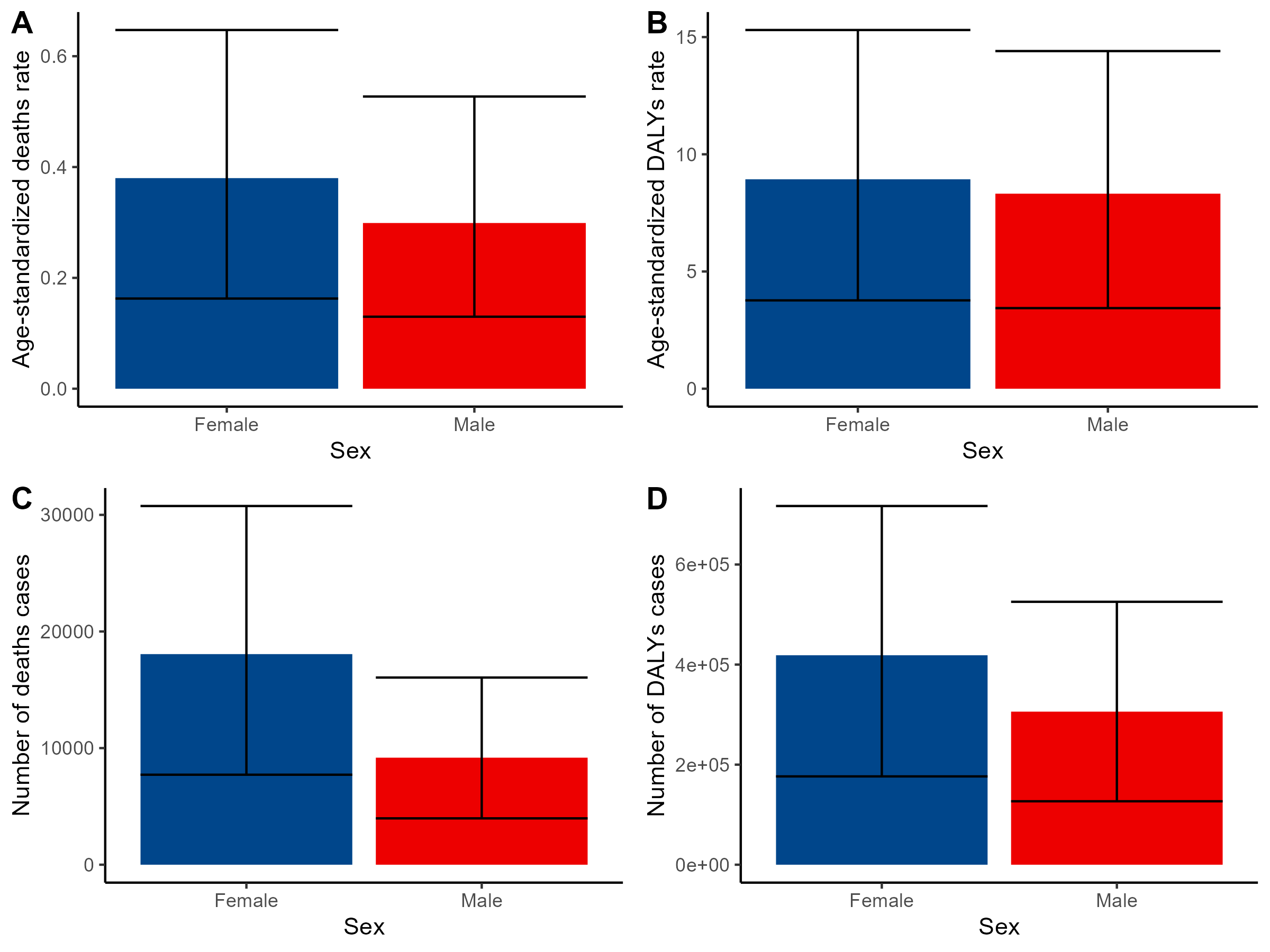

Supplement: ihaf005_Supplemental_Files [file ihaf005_supplemental_files.zip › Supplementary Figure 12.tiff]

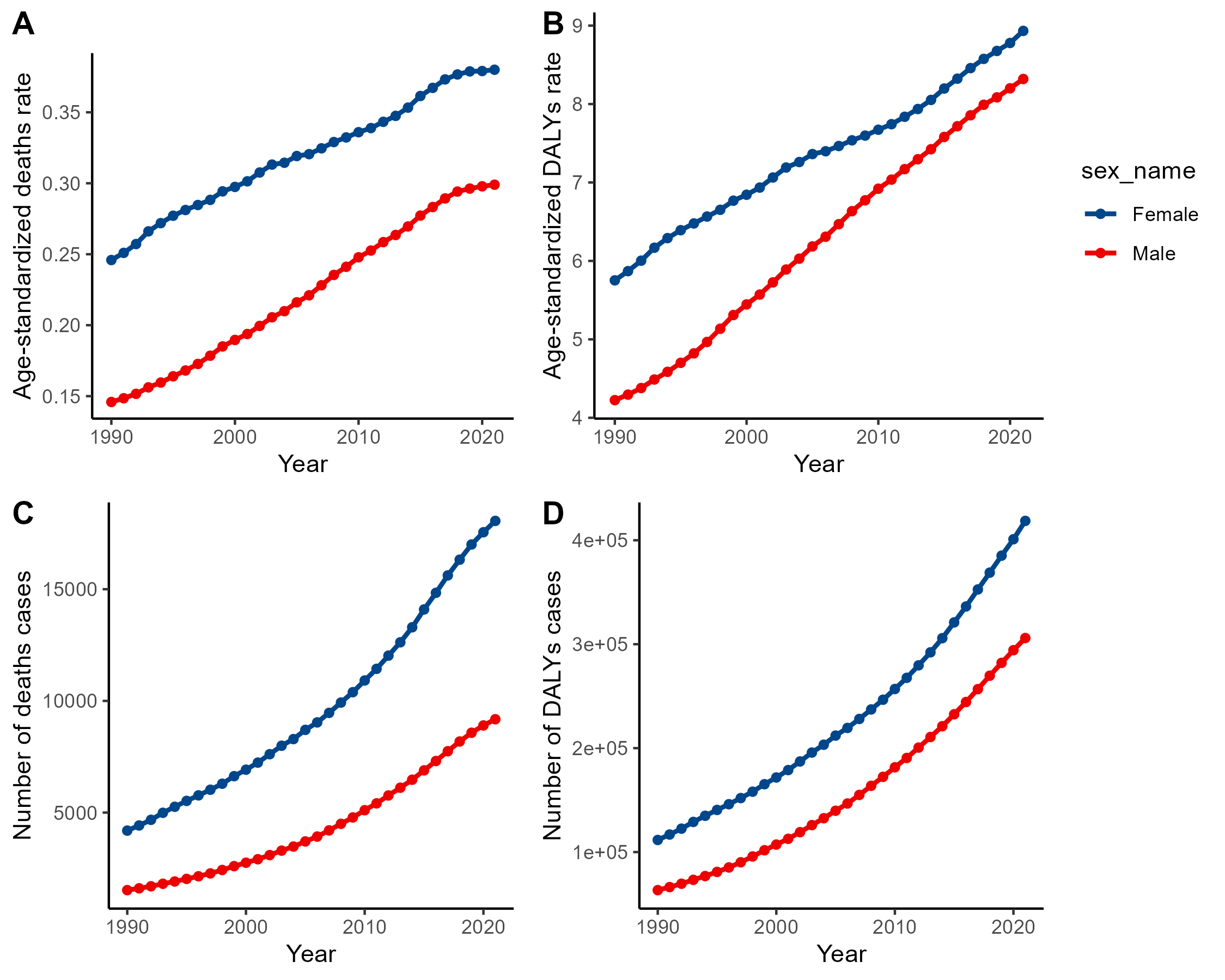

Supplement: ihaf005_Supplemental_Files [file ihaf005_supplemental_files.zip › Supplementary Figure 13.tiff]

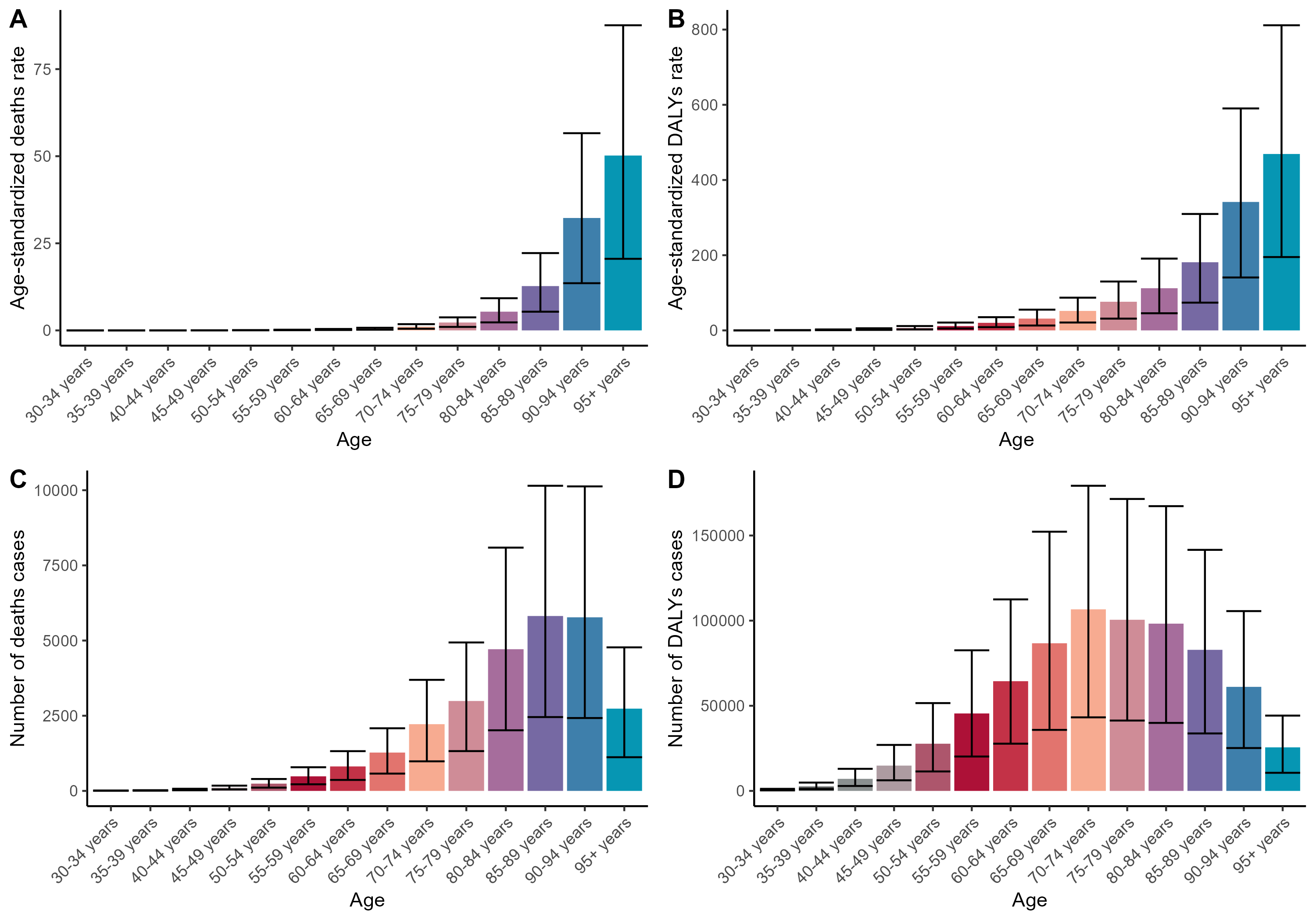

Supplement: ihaf005_Supplemental_Files [file ihaf005_supplemental_files.zip › Supplementary Figure 14.tiff]

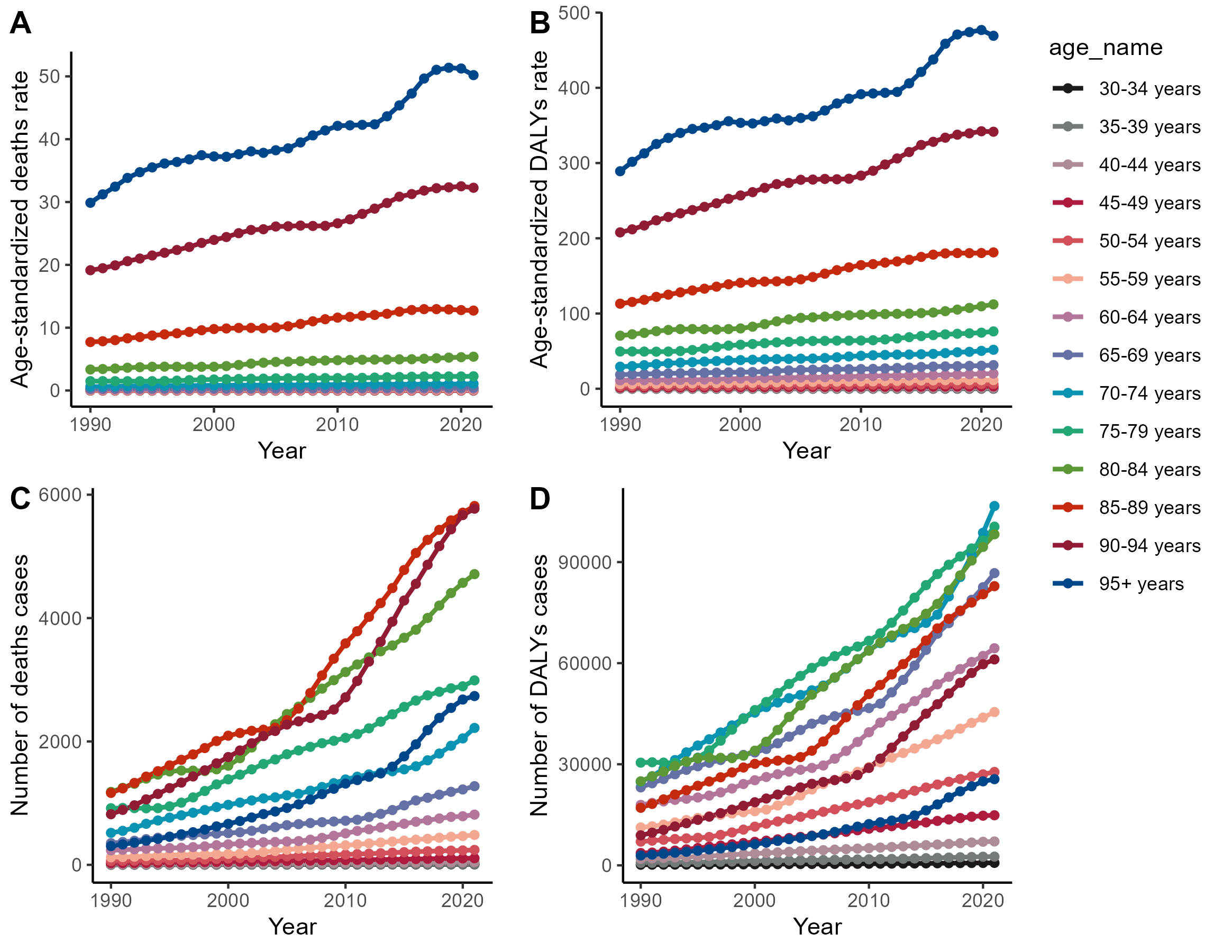

Supplement: ihaf005_Supplemental_Files [file ihaf005_supplemental_files.zip › Supplementary Figure 15.tiff]

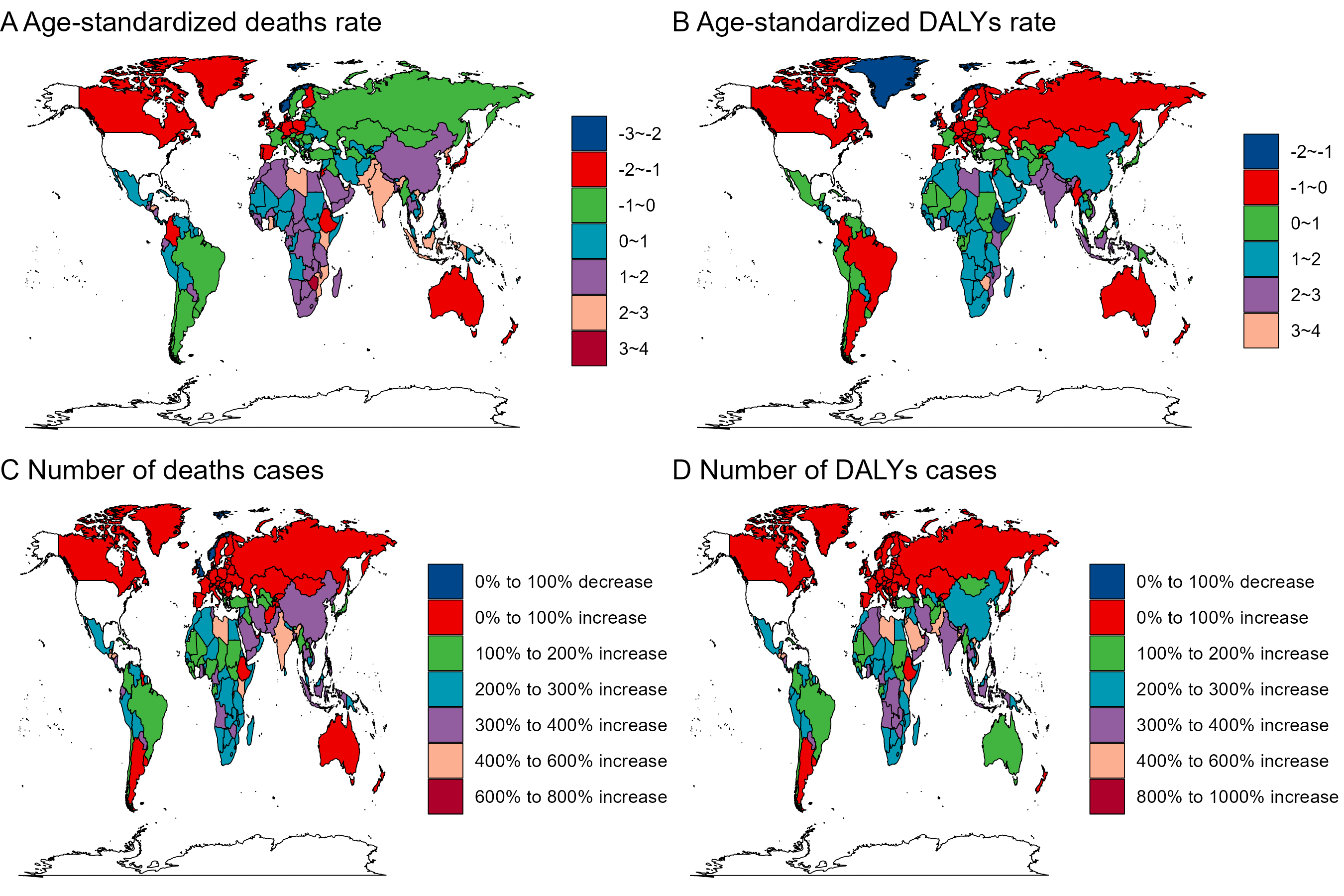

Supplement: ihaf005_Supplemental_Files [file ihaf005_supplemental_files.zip › Supplementary Figure 2.tiff]

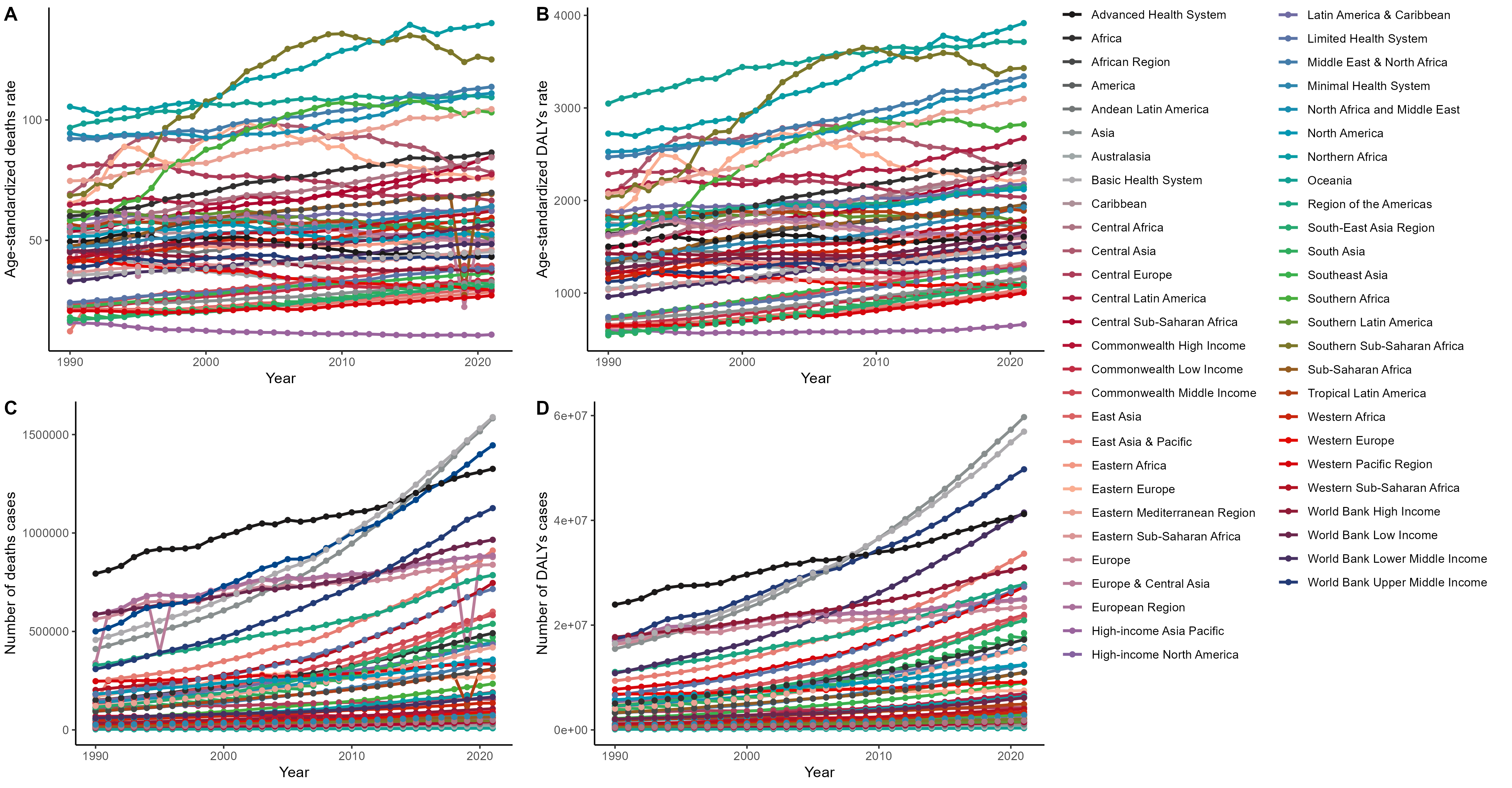

Supplement: ihaf005_Supplemental_Files [file ihaf005_supplemental_files.zip › Supplementary Figure 3.tiff]

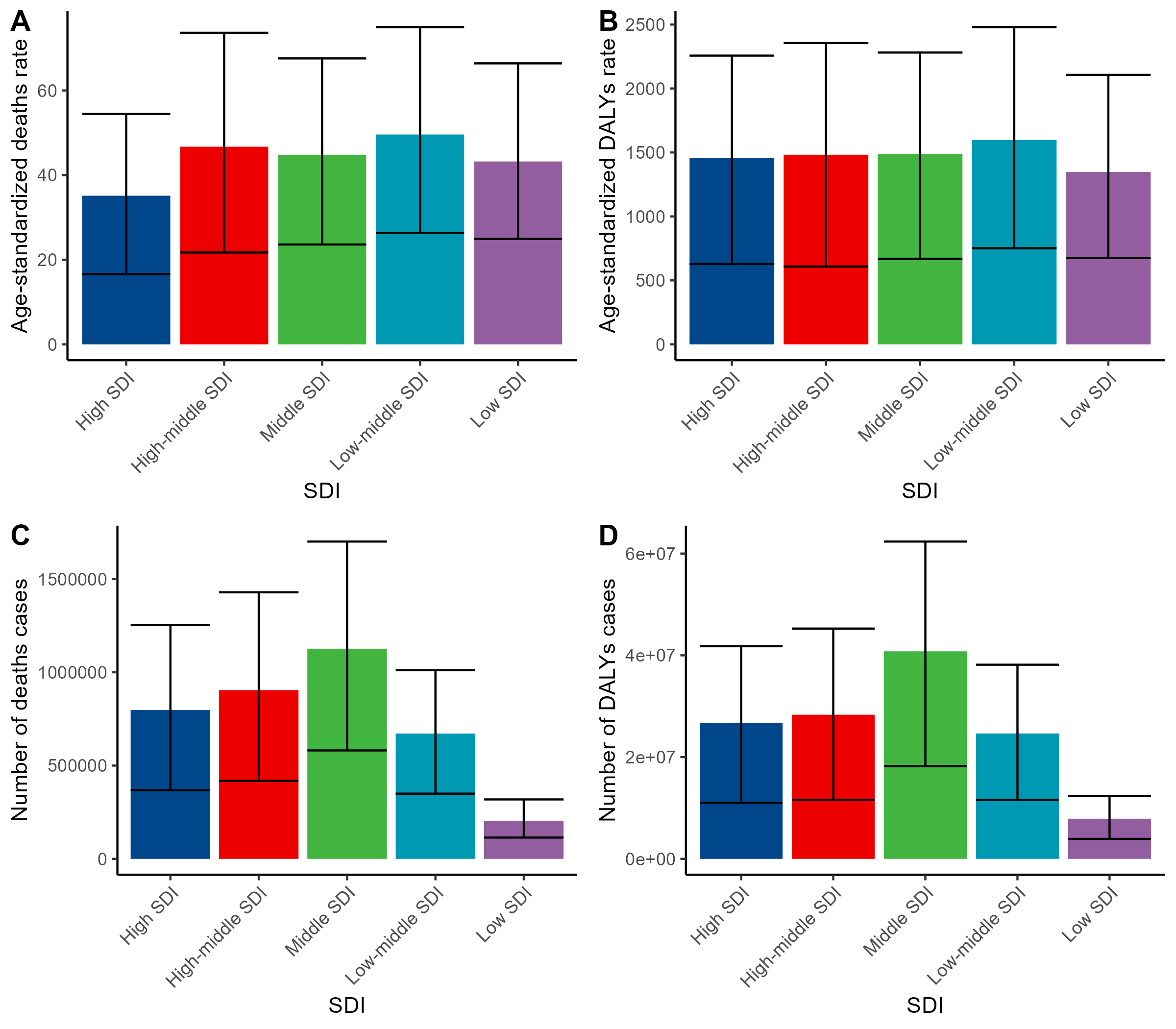

Supplement: ihaf005_Supplemental_Files [file ihaf005_supplemental_files.zip › Supplementary Figure 4.tiff]

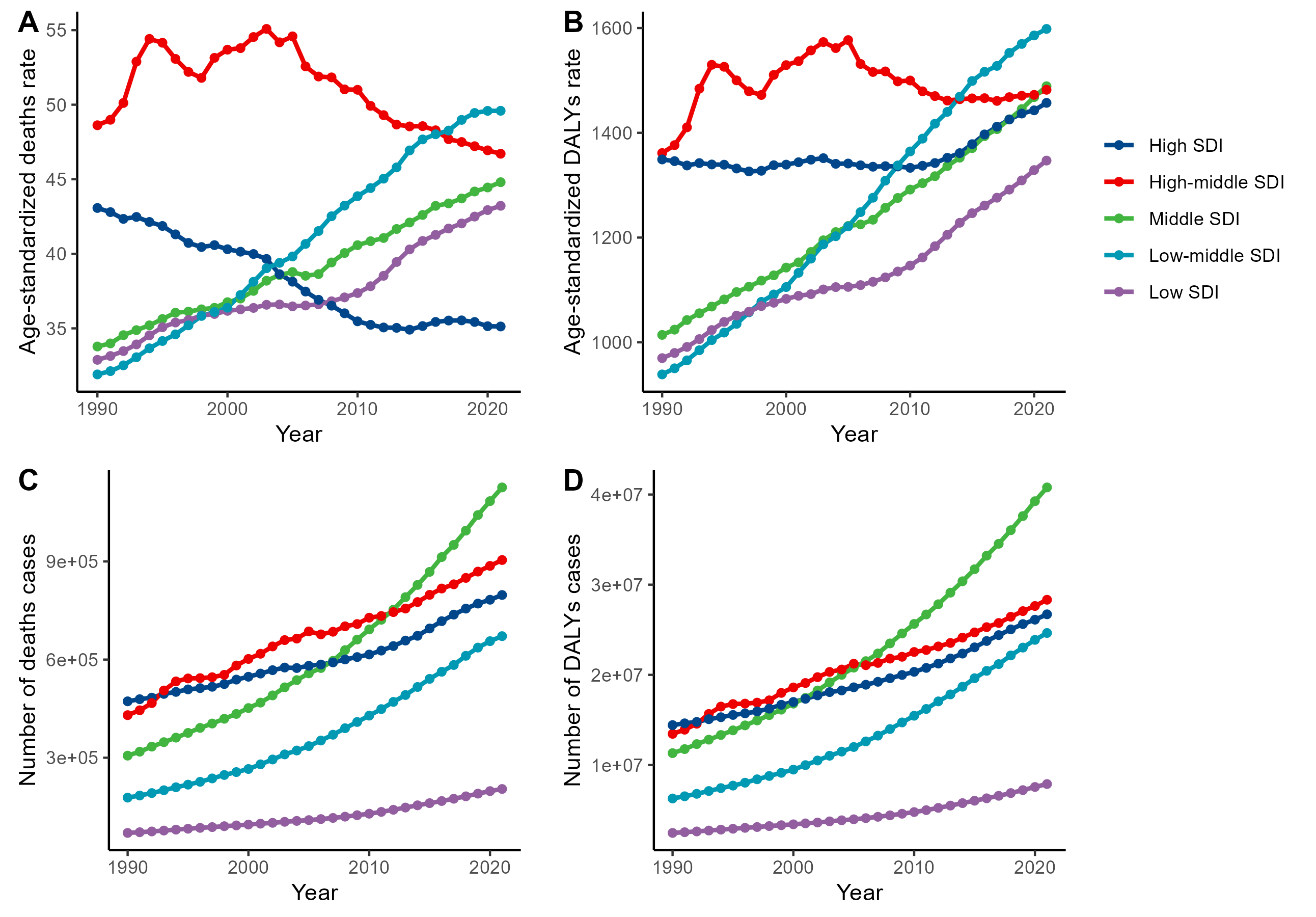

Supplement: ihaf005_Supplemental_Files [file ihaf005_supplemental_files.zip › Supplementary Figure 5.tiff]

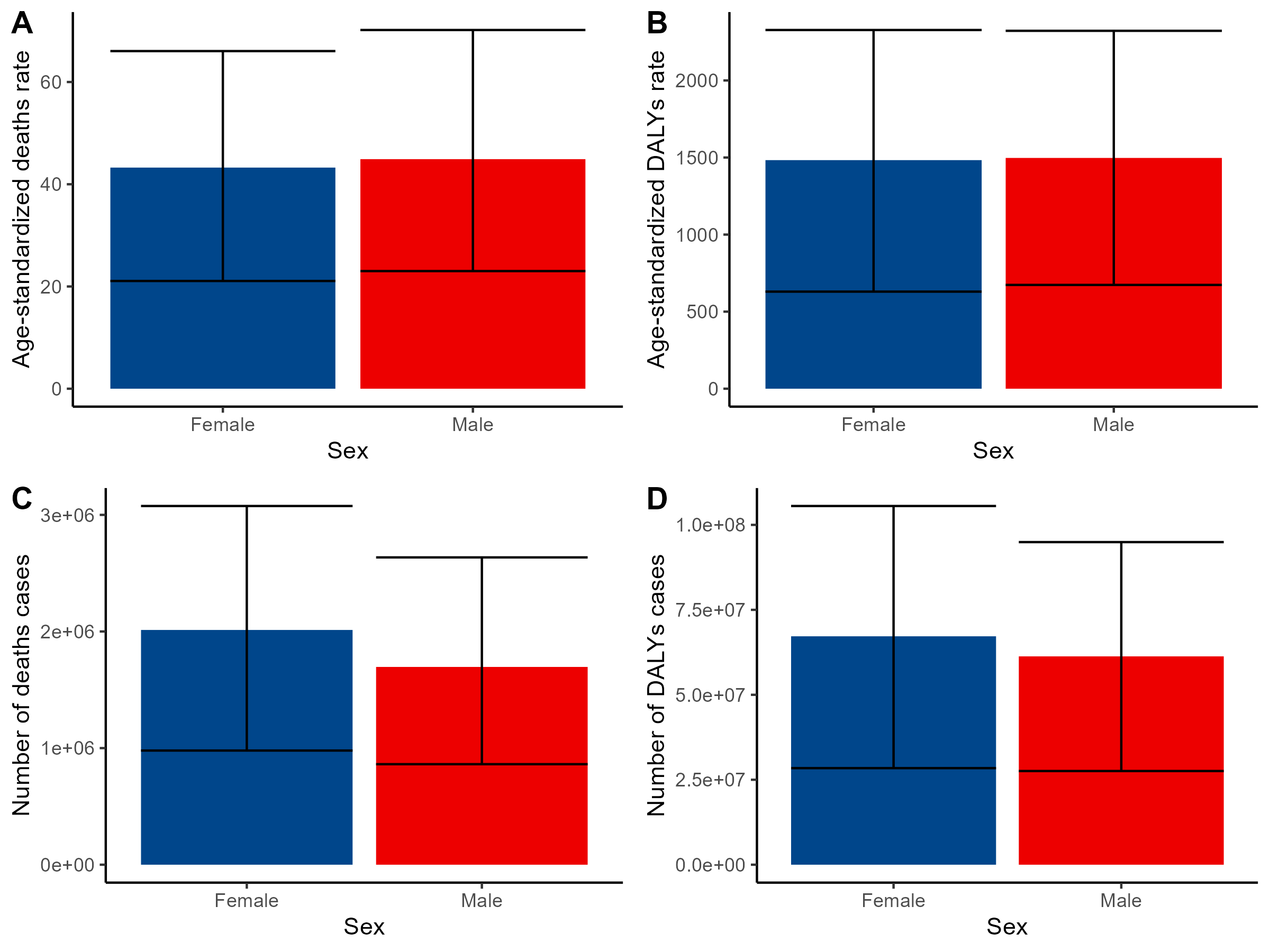

Supplement: ihaf005_Supplemental_Files [file ihaf005_supplemental_files.zip › Supplementary Figure 6.tiff]

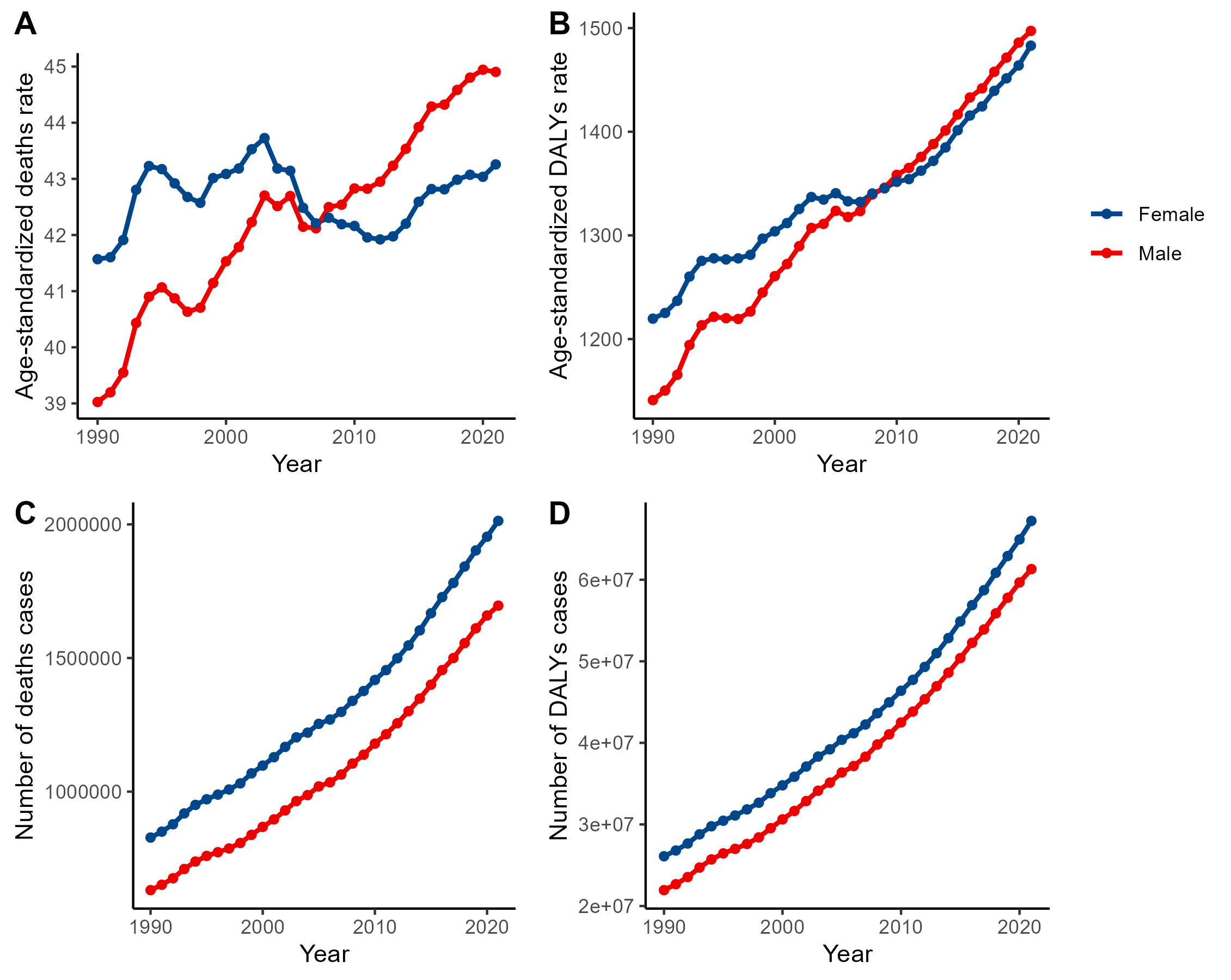

Supplement: ihaf005_Supplemental_Files [file ihaf005_supplemental_files.zip › Supplementary Figure 7.tiff]

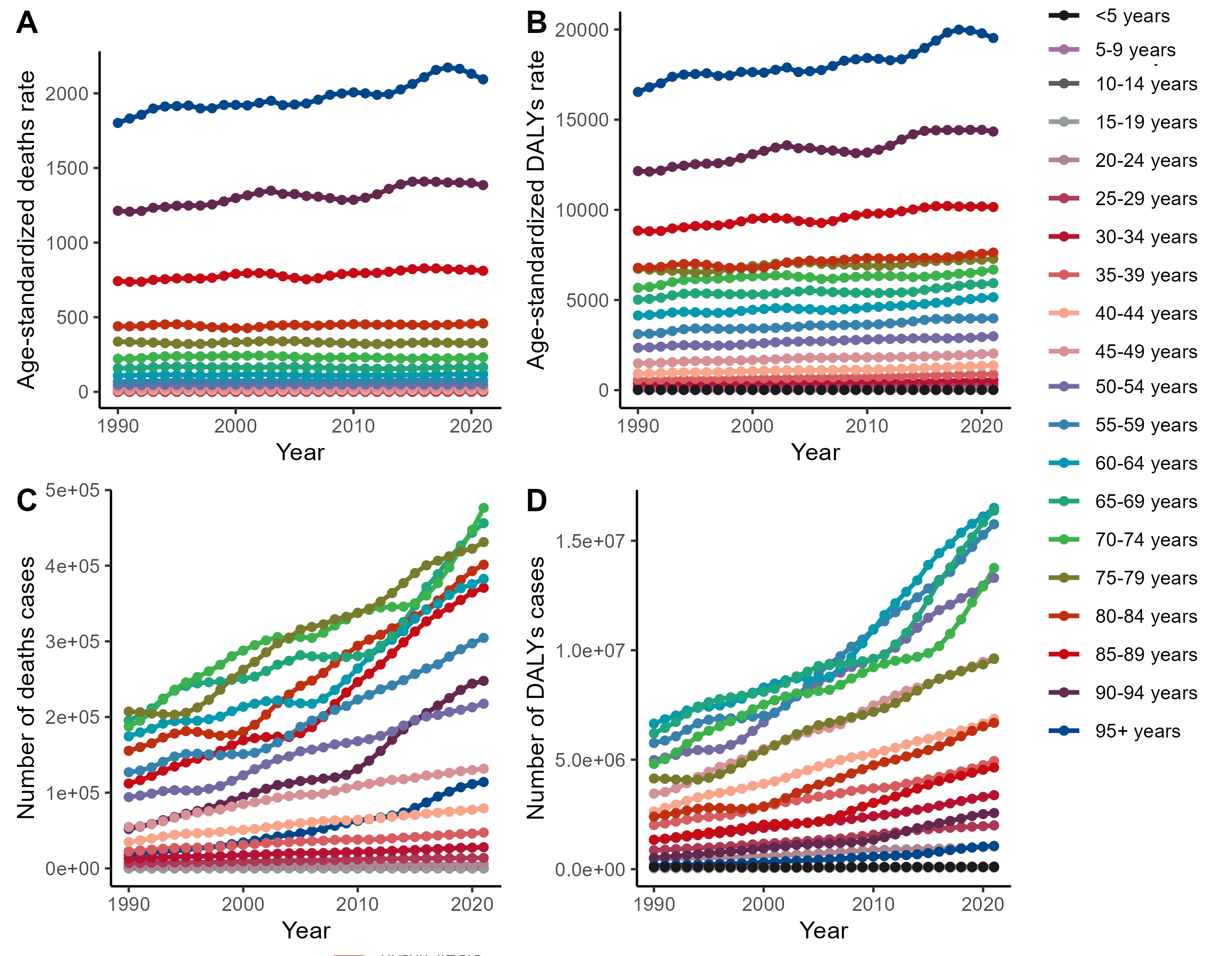

Supplement: ihaf005_Supplemental_Files [file ihaf005_supplemental_files.zip › Supplementary Figure 8.tiff]

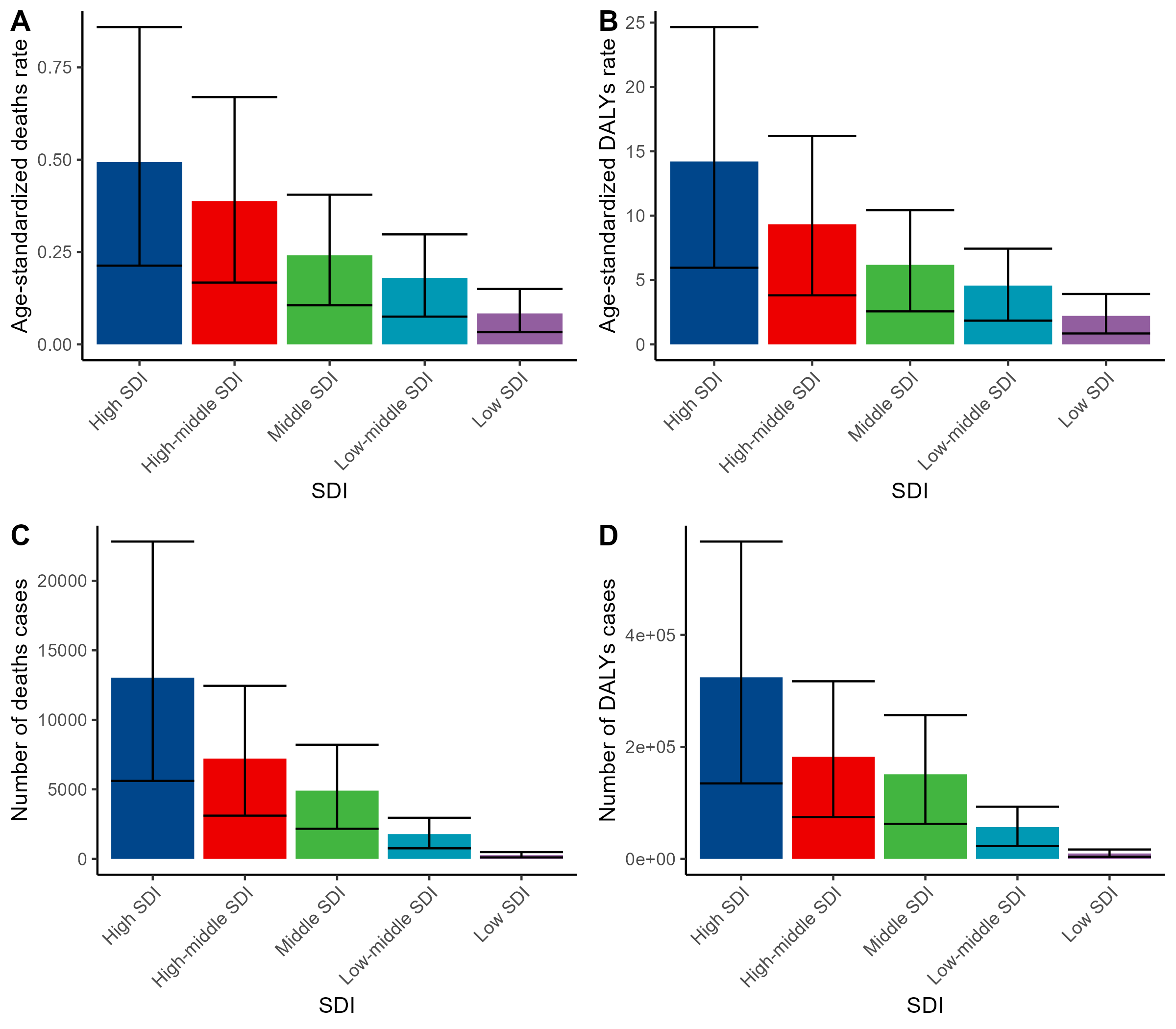

Supplement: ihaf005_Supplemental_Files [file ihaf005_supplemental_files.zip › Supplementary Figure 9.tiff]
